# Bone mineral density loci specific to the skull portray potential pleiotropic effects on craniosynostosis

**DOI:** 10.1038/s42003-023-04869-0

**Published:** 2023-07-04

**Authors:** Carolina Medina-Gomez, Benjamin H. Mullin, Alessandra Chesi, Vid Prijatelj, John P. Kemp, Chen Shochat-Carvalho, Katerina Trajanoska, Carol Wang, Raimo Joro, Tavia E. Evans, Katharina E. Schraut, Ruifang Li-Gao, Tarunveer S. Ahluwalia, M. Carola Zillikens, Kun Zhu, Dennis O. Mook-Kanamori, Daniel S. Evans, Maria Nethander, Maria J. Knol, Gudmar Thorleifsson, Ivana Prokic, Babette Zemel, Linda Broer, Fiona E. McGuigan, Natasja M. van Schoor, Sjur Reppe, Mikolaj A. Pawlak, Stuart H. Ralston, Nathalie van der Velde, Mattias Lorentzon, Kari Stefansson, Hieab H. H. Adams, Scott G. Wilson, M. Arfan Ikram, John P. Walsh, Timo A. Lakka, Kaare M. Gautvik, James F. Wilson, Eric S. Orwoll, Cornelia M. van Duijn, Klaus Bønnelykke, Andre G. Uitterlinden, Unnur Styrkársdóttir, Kristina E. Akesson, Timothy D. Spector, Jonathan H. Tobias, Claes Ohlsson, Janine F. Felix, Hans Bisgaard, Struan F. A. Grant, J. Brent Richards, David M. Evans, Bram van der Eerden, Jeroen van de Peppel, Cheryl Ackert-Bicknell, David Karasik, Erika Kague, Fernando Rivadeneira

**Affiliations:** 1 Department of Internal Medicine, Erasmus MC, University Medical Center Rotterdam, 3000 CA Rotterdam, The Netherlands; 2 Department of Endocrinology and Diabetes, Sir Charles Gairdner Hospital, Nedlands, WA 6009 Australia; 3 School of Biomedical Sciences, University of Western Australia, Nedlands, WA 6009 Australia; 4 Division of Human Genetics, Children’s Hospital of Philadelphia, Philadelphia, PA 19104 USA; 5 Department of Oral and Maxillofacial Surgery, Erasmus MC, University Medical Center Rotterdam, 3000 CA Rotterdam, The Netherlands; 6 Institute for Molecular Bioscience, The University of Queensland, Brisbane, QLD 4072 Australia; 7 The University of Queensland Diamantina Institute, The University of Queensland, Brisbane, QLD 4102 Australia; 8 MRC Integrative Epidemiology Unit, Bristol Medical School, University of Bristol, Bristol, BS8 2BN UK; 9 Azrieli Faculty of Medicine, Bar-Ilan University, Safed, 1311502 Israel; 10 Department of Epidemiology, Erasmus MC, University Medical Center Rotterdam, 3000 CA Rotterdam, The Netherlands; 11 School of Women’s and Infants’ Health, University of Western Australia, Crawley, WA 6009 Australia; 12 Institute of Biomedicine, Physiology, University of Eastern Finland, Kuopio, 70211 Finland; 13 Department of Clinical Genetics, Erasmus MC, University Medical Center Rotterdam, 3000 CA Rotterdam, The Netherlands; 14 Department of Radiology & Nuclear Medicine, Erasmus MC, University Medical Center Rotterdam, 3000 CA Rotterdam, The Netherlands; 15 Centre for Global Health Research, Usher Institute, University of Edinburgh, Edinburgh, EH16 4UX Scotland; 16 Centre for Cardiovascular Sciences, Queen’s Medical Research Institute, University of Edinburgh, Edinburgh, EH8 9AG Scotland; 17 Department of Clinical Epidemiology, Leiden University Medical Centre, 2333 ZA Leiden, The Netherlands; 18 COPSAC, Copenhagen Prospective Studies on Asthma in Childhood, Herlev and Gentofte Hospital, University of Copenhagen, Copenhagen, 2820 Denmark; 19 Steno Diabetes Center Copenhagen, Herlev, 2820 Denmark; 20 The Bioinformatics Center, Department of Biology, University of Copenhagen, Copenhagen, 2200 Denmark; 21 Medical School, University of Western Australia, Perth, WA 6009 Australia; 22 Department of Public Health and Primary Care, Leiden University Medical Centre, 2333 ZA Leiden, The Netherlands; 23 California Pacific Medical Center Research Institute, San Francisco, CA 94107 USA; 24 Bioinformatics Core Facility, Sahlgrenska Academy, University of Gothenburg, 413 90 Gothenburg, Sweden; 25 Center for Bone and Arthritis Research, Institute of Medicine, Sahlgrenska Academy, University of Gothenburg, 413 90 Gothenburg, Sweden; 26 deCODE Genetics/Amgen, Reykjavik, IC-101 Iceland; 27 Department of Pediatrics, Perelman School of Medicine, University of Pennsylvania, Philadelphia, PA 19104 USA; 28 Division of GI, Hepatology, and Nutrition, Children’s Hospital of Philadelphia, Philadelphia, PA 19104 USA; 29 Clinical and Molecular Osteoporosis Research Unit, Department of Clinical Sciences Malmö, Lund University, 205 02 Malmö, Sweden; 30 Department of Epidemiology and Data Science, Amsterdam UMC, 1081 HV Amsterdam, The Netherlands; 31 Department of Plastic and Reconstructive Surgery, Oslo University Hospital, 0372 Oslo, Norway; 32 Department of Medical Biochemistry, Oslo University Hospital, 0372 Oslo, Norway; 33 Unger-Vetlesen Institute, Lovisenberg Diaconal Hospital, 0456 Oslo, Norway; 34 Department of Neurology, Poznan University of Medical Sciences, 61-701 Poznan, Poland; 35 Centre for Genomic and Experimental Medicine, Institute of Genetics and Cancer, University of Edinburgh, Edinburgh, EH4 2XU Scotland; 36 Department of Geriatric Medicine, Amsterdam Public Health Research Institute, Amsterdam UMC, 1105 AZ Amsterdam, The Netherlands; 37 Mary MacKillop Institute for Health Research, Australian Catholic University, Melbourne, VIC 3000 Australia; 38 Latin American Brain Health (BrainLat), Universidad Adolfo Ibáñez, Santiago, Chile; 39 Department of Twin Research & Genetic Epidemiology, King’s College London, London, SE1 7EH UK; 40 Kuopio Research Institute of Exercise Medicine, Kuopio, 70100 Finland; 41 Department of Clinical Physiology and Nuclear Medicine, University of Eastern Finland, Kuopio, 70210 Finland; 42 MRC Human Genetics Unit, Institute of Genetics and Cancer, University of Edinburgh, Edinburgh, EH4 2XU Scotland; 43 Department of Public Health & Preventive Medicine, Oregon Health & Science University, Portland, OR OR97239 USA; 44 Department of Orthopedics Malmö, Skåne University Hospital, S-21428 Malmö, Sweden; 45 Musculoskeletal Research Unit, Translational Health Sciences, Bristol Medical School, Bristol, BS10 5NB UK; 46 Department of Drug Treatment, Sahlgrenska University Hospital, Region Västra Götaland, SE-413 45 Gothenburg, Sweden; 47 The Generation R Study Group, Erasmus MC, University Medical Center Rotterdam, 3000 CA Rotterdam, The Netherlands; 48 Department of Pediatrics, Erasmus MC, University Medical Center Rotterdam, 3000 CA Rotterdam, The Netherlands; 49 Division of Endocrinology, The Children’s Hospital of Philadelphia, Philadelphia, PA 19104 USA; 50 Lady Davis Institute, Jewish General Hospital, Montreal, H3T 1E2 QC Canada; 51 Department of Orthopedics, University of Colorado, Aurora, CO 80045 USA; 52 Marcus Institute for Aging Research, Hebrew SeniorLife, Roslindale, MA 02131 USA; 53 The School of Physiology, Pharmacology and Neuroscience, Biomedical Sciences, University of Bristol, Bristol, BS8 1TD UK

**Keywords:** Genetic markers, Genome-wide association studies

## Abstract

Skull bone mineral density (SK-BMD) provides a suitable trait for the discovery of key genes in bone biology, particularly to intramembranous ossification, not captured at other skeletal sites. We perform a genome-wide association meta-analysis (*n* ~ 43,800) of SK-BMD, identifying 59 loci, collectively explaining 12.5% of the trait variance. Association signals cluster within gene-sets involved in skeletal development and osteoporosis. Among the four novel loci (*ZIC1*, *PRKAR1A*, *AZIN1/ATP6V1C1*, *GLRX3*), there are factors implicated in intramembranous ossification and as we show, inherent to craniosynostosis processes. Functional follow-up in zebrafish confirms the importance of *ZIC1* on cranial suture patterning. Likewise, we observe abnormal cranial bone initiation that culminates in ectopic sutures and reduced BMD in mosaic *atp6v1c1* knockouts. Mosaic *prkar1a* knockouts present asymmetric bone growth and, conversely, elevated BMD. In light of this evidence linking SK-BMD loci to craniofacial abnormalities, our study provides new insight into the pathophysiology, diagnosis and treatment of skeletal diseases.

## Abstract (web summary)

A skull bone mineral density genome-wide association meta-analysis identifies 59 loci and studies in zebrafish evidence the role of *ZIC1*, *ATP6V1C1* and *PRKAR1A* in mineralization of the skull, and of the former two genes in cranial suture patterning.

## Introduction

Bone mineral density (BMD), as measured by Dual X-ray Absorptiometry (DXA), is the primary diagnostic marker for osteoporosis and fracture risk assessment in adults. Large-scale genome-wide association studies (GWAS), using adult BMD measurements of clinically relevant weight-bearing skeletal sites (hip and spine), have been successful in identifying genetic variants that account for a small proportion of the variance in BMD^1, 2^. Hundreds of additional BMD loci have been unveiled using other techniques like heel ultrasound^3,4^ and peripheral quantitative computed tomography (pQCT)^5^.

Skull BMD (SK-BMD) used as a trait in GWAS is favored by its higher heritability (less environmental influence)^6^, yield of identified loci and relevance with osteoporosis outcomes. For instance, a small GWAS (*n* = 9395) comprising pediatric samples identified eight SK-BMD loci, all of which were previously found associated with BMD at other skeletal sites^6^. However, genetic correlation across skeletal sites showed that SK-BMD also holds to some extent, a distinct genetic architecture^6^. In contrast to other skeletal sites, genetic investigations of the skull have the potential to unravel novel genes and pathways key to intramembranous ossification and mechano-sensing. After birth, mechanical strains on the skull are much lower than those exerted by the loading on long bones, e.g., human fibula is exposed to nearly twice the load that the human skull experiences^7^. Calvarial osteocytes are exceptionally mechanosensitive as they preserve their physiological function despite the very low stimuli of mechanical load and muscle traction^7^. Further, the skull has a dual embryonic origin from cranial neural crest cells (CNCC) and paraxial mesoderm, it is made of lamellar bone that ossifies mainly through intramembranous ossification^8^. Intramembranous bones are predominant in the head, forming the cranial vault and face; however, the cranial base grows through means of endochondral ossification^9^, and as such, investigating this skeletal site could capture molecular pathways from both processes.

In this study, we analyzed well-powered GWAS summary data to identify genetic variants associated with SK-BMD with potential pleiotropic effects on craniofacial development and disease. We found genome-wide significant (GWS) association signals mapping to four loci (*ZIC1, PRKAR1A, AZIN1/ATP6V1C1, GLRX3*) not described in previous GWAS of skeletal traits. Functional follow-up with zebrafish models provided robust evidence for the implication of *PRKAR1A, ZIC1* and *ATP6V1C1* in the mineralization of the skull and pointed to a conceivable role of these genes in craniosynostosis.

## Results

### SK-BMD GWAS meta-analysis

**Fig. 1:**
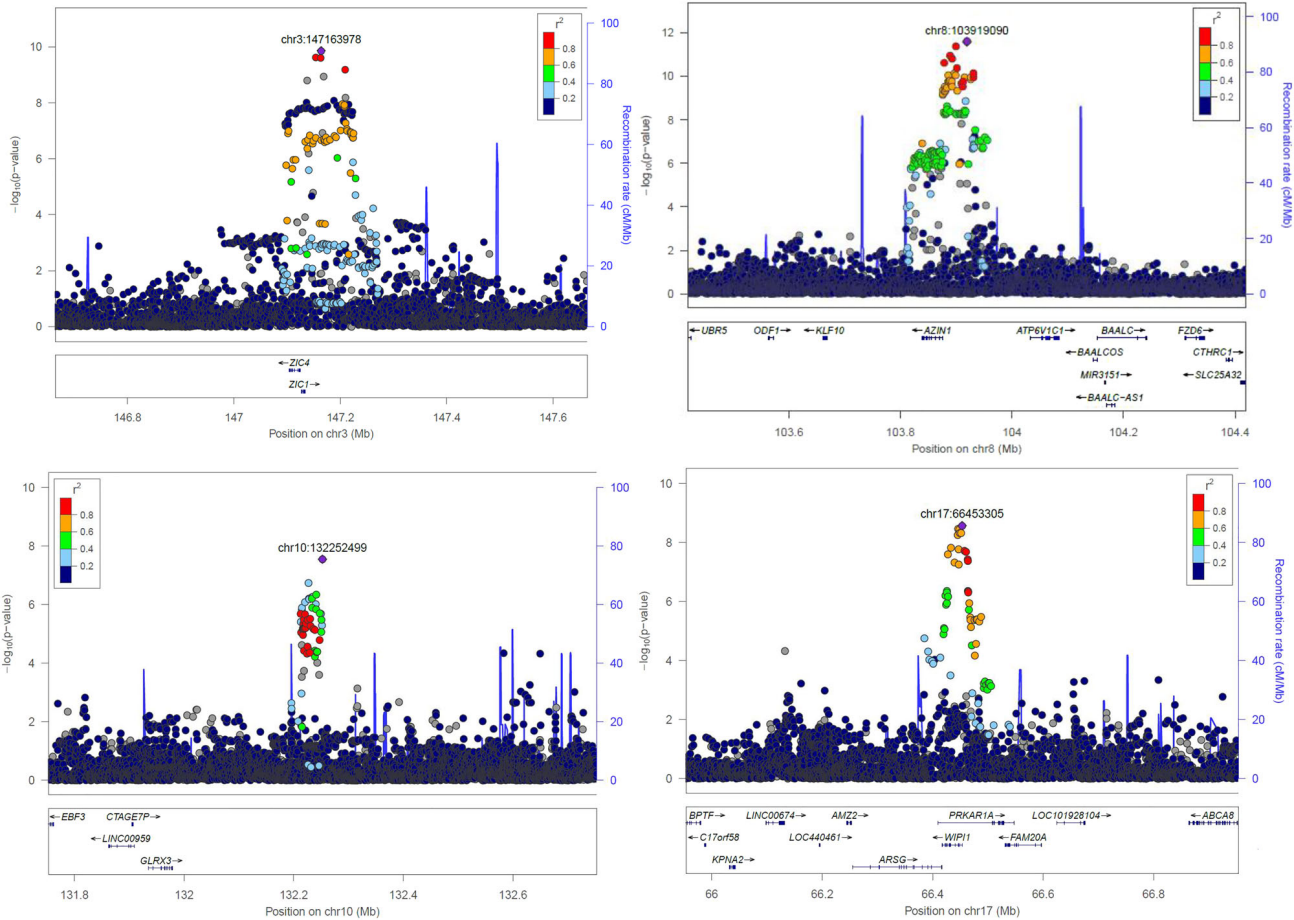
Regional plots for the four novel loci associated with SK-BMD (*P* < 5 × 10^−8^). Circles show GWAS meta-analysis P-values and position of SNPs for the overall meta-analysis (*n* = 43,800). Different colors indicate varying degrees of pair-wise linkage disequilibrium with the top marker (1000 Genomes—CEU population).

We jointly analyzed data from 21 epidemiological studies comprising ~43,800 individuals (Supplementary Data 1–2) and identified 79 independent signals mapping to 59 loci (Supplementary Fig. 1, Supplementary Table 1, Supplementary Data 3) after performing approximate conditional and joint multiple-SNP (COJO) analysis. Independent variants significantly associated with SK-BMD (*P* ≤ 5 × 10^−8^) explained 12.5% of the SK-BMD variance (see Methods). As this estimate was obtained from the same samples used as discovery, it might have been over-estimated due to the winner’s curse effect. Associated variants from 10 loci observed deleterious annotation (Supplementary Table 2). Although we observed inflation in the resulting summary statistics (*λ* = 1.09; see Supplementary Fig. 1), it was not driven by an un-modeled population structure (LD score regression intercept = 1.022). We then performed conditional analysis on variants reported by previous well-powered GWAS of bone traits^1,2,5,6,10–13^, including the UKBB e-BMD survey^4^ (Supplementary Data 4), identifying independent association signals in four loci mapping to 3q24 [*ZIC1/ZIC4*], 8q22.3 [*AZIN1/ATP6V1C1*], 10q26.3 [*GLRX3*], and 17q24.2 [*PRKAR1A/WIPI1*] (Table 1, Fig. 1). Moreover, 13 additional association signals are reported for the first time as associated with DXA-derived BMD traits (Supplementary Fig. 1). Novel independent signals within previously identified BMD loci were also detected at 1p31.3 [*WLS*], 6q22.33 [*CENPW*] and 13q14.11 [*TNFSF11*] (Supplementary Table 3, Supplementary Fig. 1), suggesting the existence of distinct regulatory elements shaping skull-BMD variation.

**Table 1:** Index SNPs of novel loci associated with BMD.

rsID	CHR	BP	Location	Gene	A1	EAF	SK-Effect	*P*	eBMD-Effect	*P*
rs12107945	3	147163978	3q24	*ZIC1*	A	0.30	−0.049	1.37E-10	−2.3E-4	0.73
rs11993347	8	103919090	8q22.3	*ATP6V1C1/AZIN1*	T	0.77	0.059	2.67E-12	0.004	0.025
rs61863293^*^	10	132252499	10q26.3	*GLRX3*	A	0.35	−0.046	2.80E-08	0.002	0.16
rs71378928	17	66453305	17q24.2	*PRKAR1A/WIPI1*	C	0.23	−0.050	2.67E-09	0.003	0.24

^*^Values for eBMD are reported for rs61861957 (r2 = 0.914). Gene names are shown in italic font.

Variants associated with SK-BMD in the meta-analysis (*N* = 43,800) that map outside + /− 500 Kb of known index SNPs of genetic associations with different bone traits. Genomic coordinates are on build 37 of the human genome. Effect sizes and allele frequencies (EAF) are reported for the A1 allele. eBMD statistics are reported from Morris et al.^4^ meta-analysis comprising 426,824 individuals.

### Heritability of SK-BMD and genetic correlation with other traits

SNP-heritability of SK-BMD was estimated to be 0.306 (SE 0.028) using linkage disequilibrium (LD) score regression^14^. The genetic correlation of SK-BMD with BMD measured at other skeletal sites ranged between 0.476 and 0.765, while it was lower with eBMD (*ρ* = 0.35; SE 0.05) and fracture risk (*ρ* = −0.38; SE 0.06) (Supplementary Table 4). Although numerous other anthropometric, immune and neurological traits were tested, only sitting height showed a significant correlation (*ρ* = 0.275; SE 0.058) with SK-BMD at the adjusted significance level (53 traits, *P* ≤ 9.4 x 10^−4^). Despite the track of skull and brain development^15^, and the association of several BMD loci such as *CENPW*, *IDUA*, *ZIC1* with parietal brain volume^16^, we did not find evidence of shared heritability between brain volumes and skull mineralization. Nevertheless, a marginally significant correlation was observed between SK-BMD and infant head circumference^17^ (*ρ* = −0.23; SE 0.07) (Supplementary Table 4).

### Biological and functional annotations of genes in the Skull BMD-associated loci

#### Tissue and cell-specific enrichment of the SK-BMD variants

**Fig. 2:**
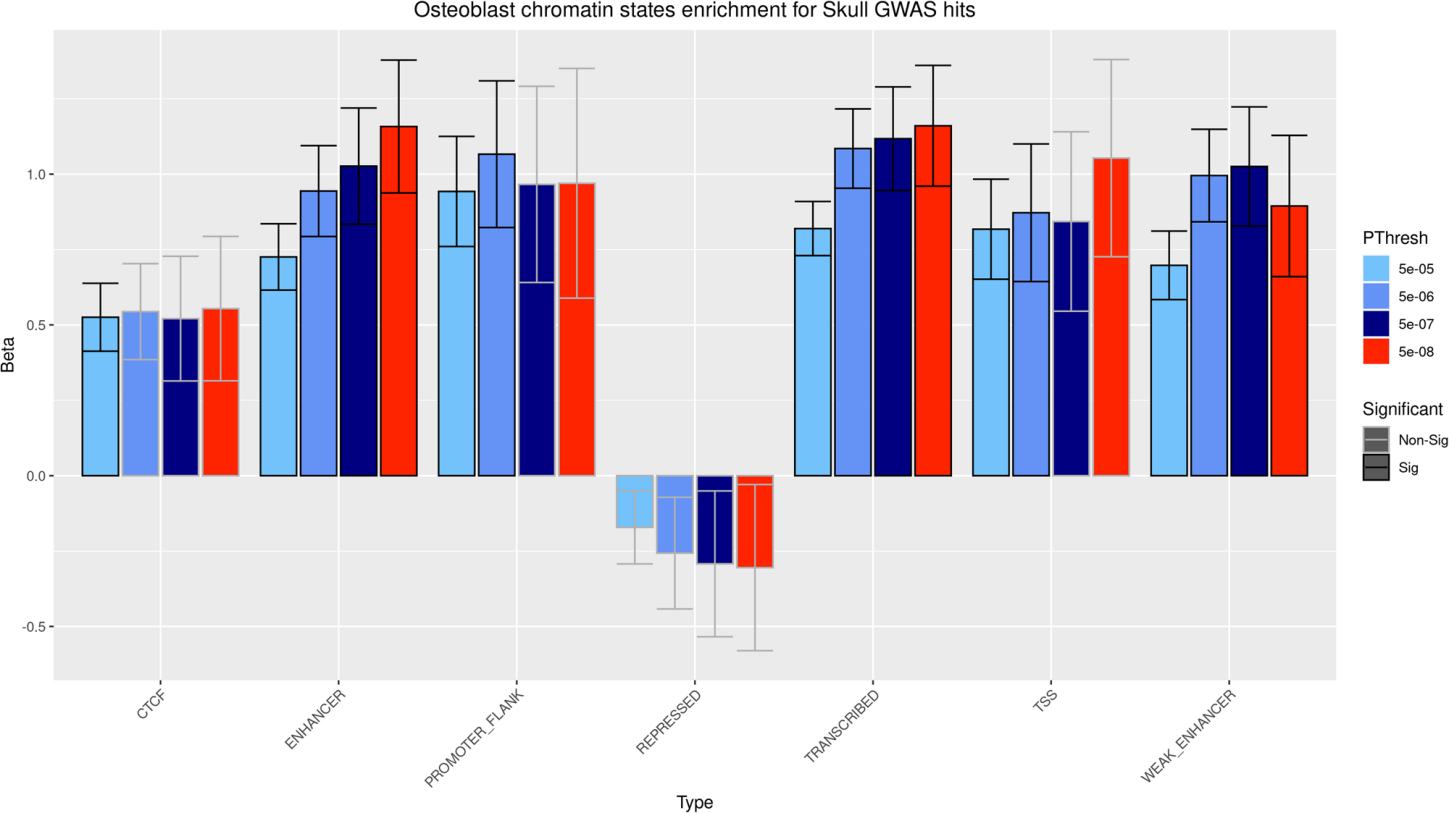
GARFIELD results for chromatin states enrichment analysis in osteoblasts. Enrichment significance was defined at *P* < 4.31 × 10^−4^ (116 effective tests performed). Bar-plot fill colors represent *p*-value GWS threshold of variants included in the analysis. Bar-plot outline colors represent significance of the enrichment. GWS variants were enriched for enhancer, weak enhancer and transcribed osteoblast regions.

**Fig. 3:**
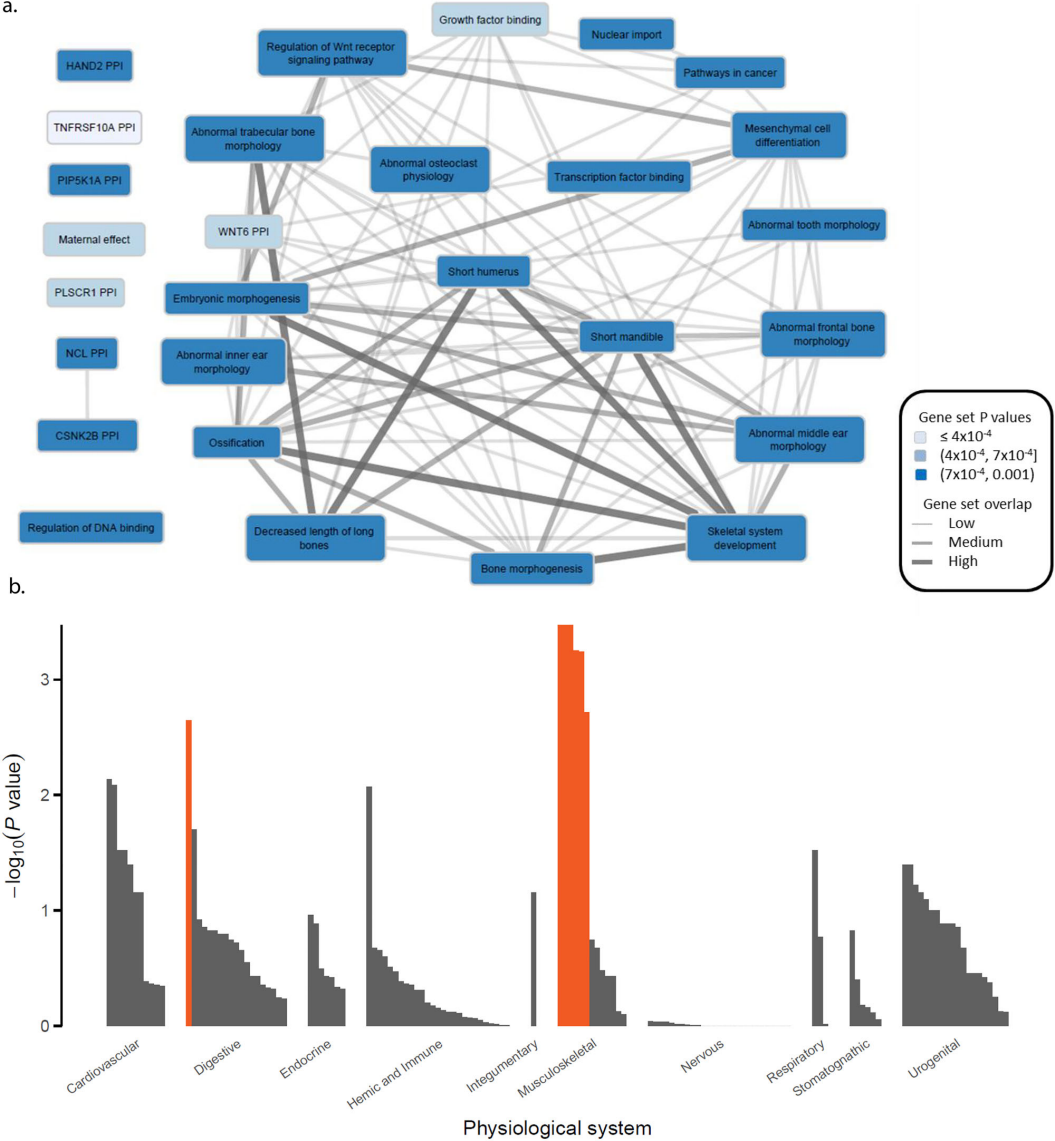
DEPICT results for gene-set and cell/tissue enrichment analyses. **a** Meta gene-sets were defined from similarity clustering of significantly enriched gene sets (FDR < 5%). Each Meta gene-set was named after one of its member gene-sets. The color of the Meta gene-sets represents the *P* value of the member set. Interconnection line width represents the Pearson correlation *ρ* between the gene membership scores for each Meta gene-set (*ρ* < 0.3, no line; 0.3 ≤*ρ* < 0.45, narrow width; 0.45 ≤*ρ* < 0.65, medium width; *ρ* ≥ 0.65, thick width). **b** Bars represent the level of evidence for genes in the associated loci to be expressed in any of the 209 Medical Subject Heading (MeSH) tissue and cell type annotations. Highlighted in orange are these cell/tissue types significantly (FDR < 5%) enriched for the expression of the genes in the associated loci.

SK-BMD GWS variants showed enrichment for enhancer, weak enhancer and transcribed regions in osteoblasts^18^ (see Methods; Fig. 2), in line with the enrichment detected for ATAC-Seq (Assay for Transposase-Accessible Chromatin using sequencing) signatures (Supplementary Data 5). This robust pattern of functional signatures was not observed in the other six cell lines tested (GM12878, H1HESC, HeLa-S3, HepG2, HUVEC, K562). A DEPICT^19^ analysis also yielded consistent results where cells and tissues from the musculoskeletal system presented the highest enrichment of gene expression within the associated loci (see Methods; Supplementary Data 6, Fig. 3b). The twenty-two genes prioritized (FDR < 0.05) were overrepresented in pathways almost exclusively involved in skeletal development and other cranial features (i.e., ear, tooth) (Fig. 3a, Supplementary Tables 5–6).

#### SK-BMD variants role in osteoclast gene expression

We cross-checked the SK-BMD signals against a recently reported osteoclast expression quantitative trait locus (eQTL) dataset^20, 21^. Six loci had eQTLs associated with a False Discovery Rate (FDR) ≤ 5%, including: *ST7L* (1q13.2), *REEP5* (5q22.2), *ING3* (7q31.31), *RIC8A* (11p15.5), and *CDC42* and *LINC00339* (1p36.12). Then, we asked whether the same causal variants may underlie these loci. Applying the Bayesian-based colocalization method^22^ suggested the sharing of causal variants in two loci (*RIC8A* and *REEP5*) (Supplementary Table 7). Further, we used summary Mendelian randomization (SMR)^23^ to determine which loci are more likely to affect SK-BMD by modulating gene expression in blood^24^ or osteoclasts^20,21^. In the blood eQTL data, seven of the fifteen genes identified by the SMR test (P_SMR_ ≤ 0.05/5912 = 8.5 × 10^−6^) showed no evidence of heterogeneity (P_HEIDI_ ≥ 0.05), including: 1p13.2 (*MOV10*); 6p21.1 (*SUPT3H*); 8q22.3 (*AZIN1-AS1*); 12q23.3 (*TMEM263*); 14q24.3 (*EIF2B2* and *MLH3*); 20q12 (*MAFB*) (Supplementary Table 8). Surveying the osteoclast eQTL data identified two loci (P_SMR_ ≤ 0.05/1077 = 4.6 x 10^−5^): *LINC00339* (1p36.12) and *TMEM8A* (16p13.3). Yet, signals in both loci showed high heterogeneity (P_HEIDI_ < 0.05) and were not present in the blood eQTL dataset.

#### Role of SK-BMD variants in the regulation of genes in osteoblasts

In an attempt to map putative causal SNPs to their target effector genes at the SK-BMD GWAS loci, we employed a high-resolution genome-scale, promoter-focused Capture-C based approach coupled with ATAC-seq in human primary mesenchymal stem cell (MSC)-derived osteoblasts^25^. After filtering out interactions between proxy SNPs (r^2^ > 0.4) and promoters not in open chromatin, the following genes were implicated by this approach *C1orf105* [1q24.3]; *TNFRSF11B*/*COLEC10* [8q24.12]; *CTTNBP2NL, MIR4256* and *WNT2B* [1p13.2]; *DMP1* and *SPARCL1* [4q22.1]; *LOC101927839* and *SMG6* [17p13.3]; *ODF1* [8q22.3]; *RUNX2* [6p21.1]; *SMAD9* [13q13.3]; *TCF7L1* [2p11.2]; *FOXD1-AS1* [5q13.2]; and *WNT4* [1p36.12], plus a set of long non-coding RNAs (Supplementary Table 9).

#### Expression of genes annotated from SK-BMD variants in bone tissue and cells

We next examined the expression of 56 protein-coding genes, prioritized from our GWAS either by location or function (see Methods) and having mouse orthologs. Fifty-two of these genes −91% (52/56), including those in the 4 novel loci, were expressed in bone tissue from human hip (iliac crest) biopsies and femoral head fragments (Supplementary Data 7).

Other than *C1orf105, Odf1, Mepe* and *Supt3h*, the additional 52 genes were expressed in murine calvaria osteoblasts (Supplementary Data 7). However, *Mepe* and *Supt3h* were expressed in murine osteoclasts. Moreover, from the 42 genes for which knockdown mouse models are available^26,27^, half showed a bone-relevant phenotype (Supplementary Data 7).

#### Relevance of SK-BMD implicated genes in bone biology

From the large number of associations identified, two main conclusions can be drawn: (1) SK-BMD is a suitable trait for the study of skeletal biology and relevant for the identification of genes and pathways involved in the development of osteoporosis (systemic low BMD), and (2) a few of the SK-BMD loci seem to be specific to the craniofacial skeleton compartment and as such were not detected in previous BMD-GWAS studies from other skeletal sites, despite having much higher statistical power with a sample size up to 10 times larger^4^.

Notwithstanding the wide range of strategies to identify potential genes underlying the associations at the novel loci (i.e., gene expression, molecular pathways, ATAC-seq lookups, eQTLs, mutational evidence and genomic location), the overlap of the lines of evidence was not conclusive. Nonetheless, two of the four novel GWAS signals mapped to genes involved in intramembranous ossification, neural crest development (*PRKAR1A*)^28^, and patterning (*ZIC1*)^29^, with the latter also involved in cranial defects where gain-of-function mutations have been described to cause craniosynostosis^30^, postulating them as strong candidates to underlie the association signal. Moreover, a gene mapping to the 8q22.3 locus, *ATP6V1C1*, codes for a subunit of the V-ATPase complex, which regulates pH by pumping cytosolic protons into intracellular organelles^31^ and plays an essential role in osteoclast-mediated bone resorption^32^. While the gene is not known to be involved in human conditions, mutations in *ATP6V0A3* (*TCIRG1*), another member of the V-ATPAse family, lead to osteopetrosis^33^.

To gain biological insight into the novel loci identified by this SK-BMD effort, we performed gene expression analyses of osteoblast differentiating hMSCs and osteoclast differentiating peripheral blood mononuclear cells (PBMCs) at different time points (Supplementary Fig. 4–6). Basal expression of *PRKAR1A* was high in osteoblast and osteoclasts and tended to reduce within the first day of osteoblast differentiation and during the proliferation, differentiation, and fusion of the osteoclasts to then return to high expression levels when the cells were mature (Supplementary Fig. 4). Conversely, expression of *ATP6V1C1* was upregulated at the onset of the extracellular matrix mineralization and at the start of the PBMC to osteoclast differentiation (Supplementary Fig. 5). The latter expression pattern is expected, given the preponderant role of this gene in the osteoclast proton pump^32^. In contrast with the results from bone biopsies, we did not find detectable expression of *ZIC1* in the surveyed cell lines (Supplementary Fig. 6, Supplementary Data 7), arguably as it is not expected that microarray data would capture the low expression of transcription factors in a given cell^34^.

### Zebrafish studies in SK-BMD and suture patterning

In recent years, zebrafish have emerged as advantageous animal models of skeletal diseases, including osteoporosis and craniosynostosis^35–37^. Zebrafish are particularly suitable to investigate craniofacial development because they permit non-invasive in vivo analysis of skull and sutures during their formation^38^. Therefore, we used zebrafish to understand the potential role of the genes mapping within the novel SK-BMD loci in craniofacial development. We chose *ZIC1*, *ATP6V1C1* and *PRKAR1A* for functional work in zebrafish as they were considered the genes with the greatest biological plausibility for involvement in the SK-BMD association and link to craniofacial outcomes as described above. The association signal in the remaining novel locus mapped ~2.7 Mb upstream of *GLRX3*, a gene involved in iron homeostasis^39^ (Fig. 1). In view of the long distance between the association signal and its closest gene, and the lack of evidence for a regulatory function of the lead variants, we did not select any gene at this locus for functional follow-up.

**Fig. 4:**
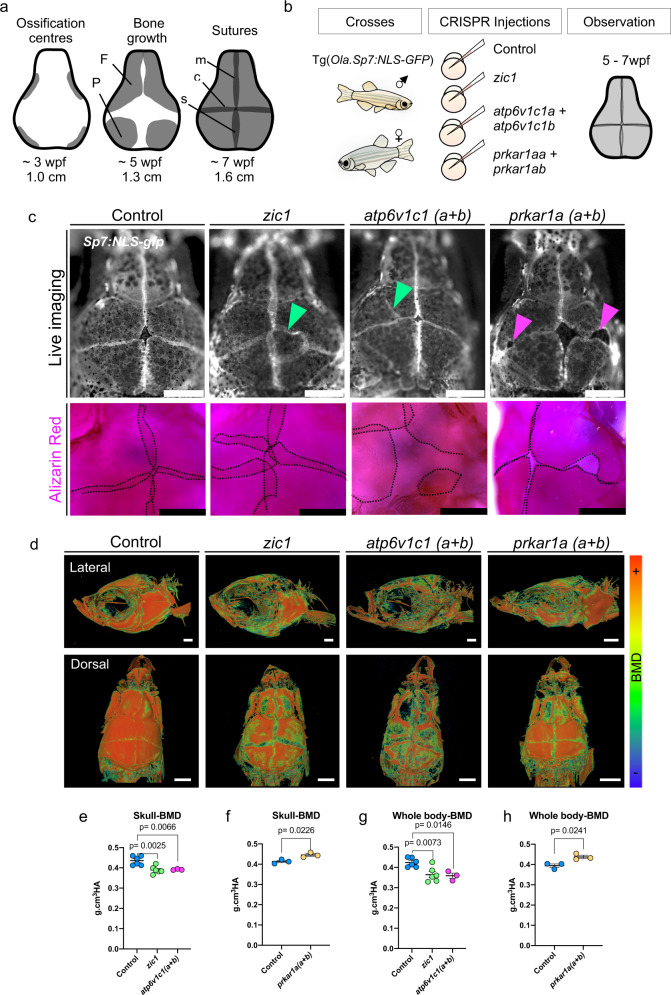
Rapid functional evaluation of novel BMD associated genes in zebrafish identifies a role of novel gene *atp6v1c1* in skull development and a role of *prkar1a* in bone growth. **a** Schematic of skull formation in zebrafish. Four ossification centres (grey) are formed by condensation of osteoblasts in the periphery of the skull at around 3 weeks post-fertilization (wpf). Ossification planes grow towards the centre of the skull forming the frontal (F) and parietal (P) bones, completely covering the brain at 7wpf and forming the metopic (m), coronal (c) and sagittal (s) sutures. **b** Schematics of the zebrafish crispant experiments. Fish carrying osteoblast reporter line Tg(Ola.Sp7:NLS-gfp) were crossed, and embryos at one cell stage were injected with the CRISPR/Cas9 system targeting respective genes. Observations were carried out in vivo during skull/suture formation (5-7wpf). Scale bars = 500 um. **c** Live imaging of skulls and ex vivo Alizarin Red Staining of control, and *zic1, atp6v1c1 (a* + *b)* and *prkar1a (a* + *b)* crispants. Ectopic sutures are indicated with green arrowheads in *zic1* and *atp6v1c1 (a* + *b)* crispants. Sutures were outlined with a dashed line in Alizarin Red pictures. Cavities in the skull are indicated with magenta arrowheads in *prkar1a (a* + *b)* crispant. Scale bars = 500 um. **d** 3D renders micro-computed topographies (µCTs) images of the skull. Images were colour-coded to show BMD variation. **e**–**h** Site-specific BMD measurements of fish of similar standard size. Data are mean ± SD. All *P*-values are indicated. Graphs were generated in Prism 8. **e**, **g** Comparison of *zic1* (*n* = 6), *atp6v1c1 (a* + *b)* (*n* = 3) crispants and controls (*n* = 6). Standard size 1.8 cm. One-way ordinary ANOVA, multiple comparisons test **f**, **h** Comparison of *prkar1a (a* + *b)* crispants (*n* = 3) and controls (*n* = 3). Standard size 1.6 cm. Nonparametric, Two-tailed, *T*-tests.

Given the high efficiency of the CRISPR system in zebrafish, CRISPR-injected fish carrying somatic mutations (crispants, G0s) recapitulate biallelic loss-of-function phenotype^40^. For rapid functional assessment of BMD-associated genes in zebrafish, we monitored the development of the sutures of crispants for each of the studied groups (control *n* = 200, *zic1* *n* = 64, *atp6v1c1 (a* + *b)* *n* = 34, *prkar1a (a* + *b)* *n* = 35) **(**Fig. 4a, b). *zic1* crispants showed abnormal skull development and bone growth (14/64, 22%) with open calvaria (caput membranaceum) or central foramina that culminated in suture mis-patterning (Fig. 4c). These results are consistent with the well-established role of *ZIC1* in suture patterning^30,41^. Similarly, *atp6v1c1(a* + *b)* crispants displayed abnormal skull development (11/34: 32%), with abnormal bone condensation, parietal foramina, uneven bone growth (frontal and parietal) and mis-patterning of the sutures*. prkar1a(a* + *b)* crispants showed slower and asymmetric bone growth, detected by small cavities in the skull during development (10/35), but without ectopic sutures. Ectopic sutures were also detected ex vivo using Alizarin Red (Fig. 4c).

We asked whether the ectopic suture phenotype in crispants was associated with biallelic mutations recapitulating knockout phenotype. For this, we used *zic1* as proof-of-principle. First, a homozygous insertion of a transposon in the *zic1* locus in medaka, that completely abolishes *zic1* expression, results in fish with asymmetrical caudal fin phenotype (Medaka Da mutant)^42^. Asymmetrical caudal fin phenotype was detected in 63% (45/71) zebrafish *zic1* crispants, suggesting a rate for biallelic mutations recapitulating the loss-of-function phenotype in *zic1*. Next, we generated *zic1* homozygous mutants (Supplementary Figs. 7 and 8), and assessed fins and skulls. Skull phenotype (irregular bone edges, abnormal and slow bone growth, ectopic sutures) was only observed in *zic1* mutants with abnormal caudal fin phenotype. Skull phenotype was variable among *zic1* homozygous mutants, like in crispants, and ectopic sutures were only found in 7% of these homozygous mutants. Our results demonstrated that while the ectopic suture phenotype is restricted to biallelic mutations, it is not a highly penetrant phenotype.

Finally, we asked whether crispants displaying skull abnormalities (biallelic mutants recapitulating knockouts) would display changes in BMD. We then performed micro-computed tomography (uCT) and analyzed BMD patterns (Fig. 4d). Comparing fish of similar standard lengths, we detected significant reduction in BMD of both skull (*P* = 0.0025) and whole-body (*P* = 0.007) in *zic1*, and *atp6v1c1(a* + *b)* crispants (*P* = 0.0066 and *P* = 0.015, for skull and whole-body respectively). Conversely, *prkar1a(a* + *b)* crispants showed increased SK-BMD (*P* = 0.02) and whole-body BMD (*P* = 0.024) when compared to wild-type (wt) zebrafish of similar standard lengths (Fig. 4e–h, Supplementary Table 10). These results suggest that genes affecting skull development, to some degree, also regulate BMD of the axial skeleton.

## Discussion

This meta-analysis of SK-BMD comprising up to 43,800 individuals emerges as the first large-scale GWAS in non-pediatric populations focusing specifically on the BMD variation of the skull. SK-BMD constitutes a trait leveraging the study of mechano-sensing and intramembranous ossification features that are more specific to cranial development, but still remaining relevant for the study of the genetics of osteoporosis and fracture risk. The high heritability and less influence of environmental pressures (i.e., skeletal loading) of the skull facilitate the identification of BMD loci harboring genes relevant to skeletal biology. Functional follow-up with zebrafish models provided robust evidence for the implication of *PRKAR1A, ZIC1* and *ATP6V1C1* in the mineralization of the skull. Further, the zebrafish crispant models implicate these three genes in processes encompassed in the pathophysiology of craniosynostosis, with *prkar1a* being involved in calvaria growth and both *zic1* and *atp6v1c1* displaying abnormal suture patterning.

Our fruitful analyses corroborate the relevance of skull BMD for the study of osteoporosis and fracture risk, as shown by the identification of GWAS signals previously discovered in studies up to ten times larger^4^ at other skeletal sites. Traditionally, BMD-related GWAS^1, 2,6,13^ have adjusted for other heritable covariates such as weight and height -in the case of pediatric cohorts- given the impact of these phenotypes either in bone mass or its measurement. Yet, these adjustments could introduce collider bias^43^ and risk of false positives. However, BMD loci unveiled by other GWAS^1,2,6,13^ and our study and by our current study have been robustly replicated in the ultrasound effort ran in the UKBB^4^, which did not adjust for heritable covariates, making less likely the presence of false positives due to a potential collider effect.

Based on GWAS summary statistics, we found a moderately high genetic correlation between skull BMD and BMD at other skeletal sites, describing the genetic component controlling systematic processes of bone development while still other genetic factors present (as denoted by the novel signals) seem to have a predominant role only in the skull bones. Overall, GWS signals mapped to genes displaying enrichment for expression in musculoskeletal-related cells and tissues and were solidly represented in SMAD binding and TGFBR2 PPI subnetwork signaling.

Whereas GWS SNPS showed enrichment in enhancers and transcribed regions on osteoblasts, the identification of the underlying causal gene at the different associated loci was elusive. We attempted gene prioritization using lines of evidence from *in-silico* datasets (i.e., DEPICT, CADD scores), chromatin conformation in osteoblasts, eQTLs in osteoclasts, expression in murine and human bone cell lines and additional evidence from the literature. However, none of the strategies was effective in conclusively identifying potential genes underlying the association in the four novel loci, and in general, the overlap of these evidence lines was not overwhelming. Therefore, follow-up prioritization was based on the suggested function of the genes mapping to the four new loci. Our zebrafish experiments provided strong genetic evidence of the involvement of prioritized genes in the process of skull mineralization. Yet, we cannot guarantee that there are no additional genes underlying the association at specific loci. The evidence resulting from integrating our GWAS results with chromatin annotations and transcriptomic data was scattered across the distinct types of bone cells, overall showing heterogeneous results. Therefore, we cannot exclude that our GWS signals originate from processes stemming from either cells or tissues that play a role in mineralization, but were not integrated into this study.

Gain-of-function mutations in *ZIC1* are known to cause craniosynostosis (premature fusion of the cranial sutures)^29,44^, while loss-of-function mutations in *ZIC1* are associated with alterations in the formation of calvaria foramina and skull ossification defects (caput membranaceum)^41^. Our zebrafish experiments demonstrated the involvement of *zic1* in the regulation of skull bone condensation, growth, and mineralization. *Atp6v1c1* and *prkar1a zebrafish* crispants revealed a similar phenotype to that observed from the *zic1* experiments, including abnormal osteoblast condensation at the ossification centres and delayed bone growth. Although ectopic sutures were detected in *zic1* and *atp6v1c1*, they were not detected in *prkar1a* crispants, what could be the result of an incomplete penetrance of the phenotype. The detection of ectopic sutures in only a small subset of homozygous *zic1* mutants was not surprising. Previous studies on loss-o-function mutation of *sp7* have demonstrated that the development of ectopic sutures varies in position, numbers, and sizes among mutants^38^. Importantly, zebrafish mutants for established craniosynostosis genes (i.e., *FGFR3*^45^ and *TWIST1* and *TCF12*^46^) also displayed abnormal cranial bone growth and eventual ectopic sutures, which reinforce a plausible role of the prioritized genes in the disease pathogenesis.

While our functional studies focused on genes uniquely associated with variation in skull BMD, there are plenty of genes implicated in craniosynostosis (e.g., *EN1*, *RUNX2, SOX6, BMP2, JAG1, LRP5, IDUA*^30,44,47,48^) across the whole set of identified BMD loci (Supplementary Table 1). In craniosynostosis, exacerbation of osteoblast differentiation at the osteogenic fronts of the cranial plates can lead to abnormal extracellular matrix secretion and bone deposition resulting in premature fusion of the sutures^49^. For instance, haploinsufficiency of *RUNX2* causes cleidocranial dysplasia, a condition that displays opened cranial sutures and lack of mineralization (patent fontanels) in the calvaria^50^. Therefore, genes involved in the regulation of osteoblast differentiation may play an important role in suture patency, warranting scrutiny in craniosynostosis patients in whom the definite mutation underlying the condition has not yet been identified. For instance, the pattern of expression of *ATP6V1C1* observed in osteoclast differentiating PBMCs is in line with its described role in osteoclast activation^32^. Yet, opposite to what was described before^32^, we do observe expression of *ATP6V1C1* during osteoblast differentiation, more specifically at the onset of extracellular matrix mineralization. The role of *ATP6V1C1* in extracellular matrix mineralization is further supported by our zebrafish images of skull development, where abnormal condensation and growth of osteoblasts were detected in vivo. Such changes in osteoblast behavior resulted in a lower rate of cranial bone growth during skull formation. Recently, we have expanded the discussion about the genetic overlap between BMD genes and craniosynostosis^37^.

Altogether, this study constitutes the largest genome-wide survey for genes involved in skull BMD variation. We demonstrated that the skull is a skeletal site aiding the identification of genetic factors that remain relevant for the study of osteoporosis and fracture risk. Further, the study of skull BMD allows capturing elements of biological processes, like intramembranous ossification, that cannot be assessed by studies of BMD at other skeletal sites. Also, this GWAS of skull BMD unveiled a link between bone mineralization and the pathogenesis of craniosynostosis, as illustrated by the consequences of *ZIC1* disruption in zebrafish skull mineralization. The potential pleiotropic effects of the identified genes involved in both skull BMD variation and the pathophysiology of craniosynostosis open new avenues for the diagnosis and pharmacological treatment of the disease.

## Methods

### Study populations

This study comprised ~43,800 individuals, taking part in 21 epidemiological studies worldwide (Supplementary Data 1–2). Most of these individuals were adults (>18 years, 75%) from cohorts of European background (85%). Written informed consent was provided by all subjects (or their parents in the case of children), and this study was approved by the corresponding Medical Ethics Committee of each participating study. Skull BMD (g/cm^2^) was measured by DXA in each participating research center following standard manufacturer protocols (Supplementary Data 1). All individuals included in this study had genome-wide array data undergoing quality control and imputed to the 1000 genomes phase 1 version 3 (March 2012) reference panel or the combined 1000 genomes and the UK10K reference panels (Supplementary Data 1).

### SK-BMD GWAS analyses

Individual cohorts generated standardized SK-BMD residuals after adjustment for age, weight, height and genomic principal components, either in sex-combined (family-based studies) or in sex-specific (population-based studies) models then combined for analysis. These standardized residuals were tested for association with genome-wide single nucleotide polymorphisms (SNPs) using an additive model. A centralized quality-control procedure implemented in EasyQC^51^ was applied to individual cohort association summary statistics. Cohort-specific errors in phenotype residual transformation or inflation arising from population stratification, cryptic relatedness and genotype biases were evaluated and corrected when necessary. Moreover, variants with missing information, or nonsensical values (e.g., absolute beta estimates or standard errors >10, association *P*-values >1 or <0; or imputation quality <0; infinite beta estimates or standard errors); minor allele frequency (MAF) less than 0.5%; imputation quality scores <0.4 (Impute info) or <0.3 (Minimac r^2^), were excluded. An inverse-variance meta-analysis applying genomic control was carried out in METAL^52^, surveying 19,211,311 markers present in at least three studies after quality control. We applied the conventional GWS (*P* ≤ 5x10^−8^) threshold for SNP discovery. Conditional analyses were undertaken based on the meta-analysis results employing an iterative strategy as implemented in GCTA^53^, using the Rotterdam Study I (*n* = 6291) dataset as a reference for precise calculation LD between the analyzed markers. We employed this tool to determine: 1) independence of association signals within loci discovered in our study; and 2) independence of the association signals discovered by our meta-analysis from the 570 independent variants, present in our meta-analysis, that have been reported previously in well-powered GWAS of relevant bone traits^1–3,5,6,10–13^. The genetic variance explained (σ^2^_*snp*_=2β^2^xMAFx(1-MAF))^54^ was calculated for each independently associated variant, where β represents the effect size per-SNP in SD units and MAF is the minor allele frequency per variant. The total variance explained by GWS variants corresponds to the summation of these values.

### Shared Genetic architecture of SK-BMD, fracture and other traits

We used LD score regression^14^ (LDSR) as implemented in the LDHub web interface^55^ to rule out that our results were a product of residual population stratification or cryptic relatedness. LDSR was also used to estimate the SNP heritability of SK-BMD. Likewise, we used LDHub to estimate the genetic correlation (ρ_g_) between SK-BMD and relevant traits based on available GWAS summary statistics^14^. In addition, we assessed the genetic correlation between SK-BMD and other relevant traits not present in the LDHub database, i.e., all-type of fracture^56^, handgrip strength^57^ and different lobar brain volumes^16^.

### Search for biological and functional knowledge of the identified association regions

Functional mapping and annotation of genetic associations were performed with FUMA^58^. Also, Combined Annotation Dependent Depletion (CADD) scores for exonic variants were retrieved from this tool as well as annotations to the GWAS catalogue to examine pleiotropic relationships.

Enrichment of GWAS variants for regulatory annotations was tested using the GWAS Analysis of Regulatory or Functional Information Enrichment with LD correction (GARFIELD)^18^. We analyzed our variants for enrichment at variable GWS p-value thresholds (i.e., 5 × 10^−5^, 5 × 10^−6^, 5 ×  10^−7^, 5 × 10^−8^) against chromatin states and histone modifications of osteoblast signatures (as acquired from multiple sources^59,60^) and across other cell lines (GM12878, H1HESC, HeLa-S3, HepG2, HUVEC, K562; as provided by GARFIELD). We tested for enrichment of ATAC-Seq^4^ and DNase I hypersensitive site (DHS) peaks of osteoblast signatures^61^. In total, 116 effective enrichment tests were run across all cell lines and features, including ATAC-Seq and DHS marks. Significance was set at (*P* = 0.05/116, *P* < 4.31 × 10^−4^). To note, data on methylation of the fourth lysine residue at the third histone (H3K4me1) was present only in four cell lines. Also, we used DEPICT^19^ at a GWS threshold to prioritize genes in the associated regions and highlight important pathways influencing skull mineralization. Enriched gene-sets were grouped based on the degree of gene overlap into ‘meta gene-sets’ using affinity propagation clustering^19^. Visualization was carried out in Cytoscape 3.4.

### Osteoclast eQTL analysis

We performed an eQTL analysis of variants based on a recently published dataset^20,21^. A detailed description of the patient recruitment process and laboratory protocols used in this study can be found elsewhere^20^. Briefly, gene expression profiles were generated by performing RNA-Seq on osteoclast-like cells differentiated from PBMCs in-vitro. These cells were isolated from 158 female patients aged 30 to 70 years for whom genome-wide genotype data imputed to the haplotype reference consortium (HRC) panel release 1.1 was also available. The eQTL analysis^20^ was performed on the osteoclast gene expression data normalized using the trimmed mean of M-values method and corrected for total read count by conversion to counts per million using the *edgeR* package in R^62^, and only included variants with a MAF ≥ 5%. Models were adjusted for age, RNA-Seq batch and 10 principal components. Each variant was tested for association with the expression of any gene with a transcription start site that fell within a 1 Mb window (*cis-*eQTLs). Correction for multiple testing was performed using the Benjamini-Yekutieli procedure with a FDR of 5%. We used two different approaches to analyze this data, namely: 1) Co-localization of GWAS/eQTL association signals^22^ and 2) SMR analysis^23^, as we briefly explain below. *Co-localization analysis*: Assessment of co-localization, using a Bayesian framework to calculate posterior probabilities to quantify support for five different hypotheses regarding the presence and sharing of causal variants for eQTLs and SK-BMD, was performed using the *coloc* package in R^22^. *SMR analysis*: This method tests for association between gene expression and a given trait using the most associated eQTL as a genetic instrument. The software performs a SMR test, which uses the top eQTL variant for each gene to identify association signals present in both the GWAS and eQTL datasets. The software also performs a HEIDI test to determine if there is a single causal variant underlying the GWAS and transcriptomic signals. A significant HEIDI test at a particular locus indicates the presence of heterogeneity for the two datasets, indicating the association signals are less likely to be driven by the same causal variant. We followed this approach directly in the osteoclast‐specific eQTL dataset from 158 participants described above^20^, and also in the better-powered eQTL study in whole blood reported by Westra et al.^24^. The genotype data from the osteoclast eQTL cohort was used as the reference panel in this analysis for estimation of LD. The analysis only included genes with at least one eQTL association at *P* ≤ 5 × 10^−8^, with correction for multiple testing performed using the Bonferroni method.

### Osteoblast ATAC-seq and Capture-C

We scrutinized a database of genome-wide interactions of all human promoters in an osteoblast model using ATAC-seq and high-resolution Capture-C recently developed^25^. In short, a custom Agilent SureSelect RNA library targeting *DpnII* restriction fragments overlapping 36,691 promoters of protein-coding, noncoding, antisense, small nuclear (sn)RNA, micro (mi)RNA, small nucleolar (sno)RNA and long intergenic noncoding (linc)RNA genes was designed. Then, genome-wide, promoter-focused high-resolution Capture-C was applied to hMSC-derived osteoblasts. Also, ATAC-seq open chromatin maps from the same samples were generated to determine informative proxy SNPs for each of the SK-BMD loci. The intersection of these two datasets provides an indication of the genes being targeted by the SNPs associated with SK-BMD and thus, more likely to be mediating the association signal. Significant interactions were called using the CHiCAGO pipeline^63^.

### RNAseq of whole human bone tissue

RNAseq of whole human bone tissue was assessed in seventy-one biopsies from female iliac bone donors and subchondral bone fragments from fifty patients undergoing hip replacement surgery due to hip fracture or osteoarthritis which were subjected to transcriptomic analysis. Detailed descriptions of sampling and characteristics of these women part of the Osteogene study can be found elsewhere^64^. For the bone fragment collection, standardized extraction from a 1 cm^2^ area of the caput was performed during surgery and frozen in liquid nitrogen. The frozen bone fragments were then pulverized in a mortar followed by RNA extraction with a Trizol reagent (Life Technologies, Gaithersburg, MD) and further purification using a RNeasy kit (Qiagen). RNA from all the bone samples (biopsies and surgical fragments) were then sequenced in a single batch using TruSeq RNA Library prep kit V2 (Illumina) and single-indexed adapters. Paired-end sequencing with 2 x 50 bp was performed using the Illumina Hiseq2000 platform to obtain at least 6,000,000 reads per library. Transcript level expression values were then created using an in-house pipeline utilizing Picard tools (http://broadinstitute.github.io/picard/), GATK^65^ and featureCounts^66^. Sample-donor annotation concordance was ensured. No library had fewer than 100,000 counts. Expression data was then quantile normalized and genes not expressed in at least 75% of libraries were excluded from analyses.

### RNA expression of human mesenchymal stem cells and peripheral blood mononuclear cells

Human bone marrow-derived mesenchymal stem cells [(hMSC), Lonza Group Ltd., Basel, Switzerland] were seeded in 12-well plates (5 × 10^3^ cells per cm^2^) and differentiated into osteoblasts (using α-Mem pH7.5, 10% heat-inactivated foetal calf serum (FCS), 100 nM Dexamethasone and 10 mM β-glycerophosphate). As mentioned in the datasheet provided by the company, cells were authenticated by FACS analyses for the presence of surface markers CD105, CD166, CD29 and CD44 and the absence of CD14, CD34 and CD45. In addition, osteogenic and adipogenic differentiation was shown by alizarin red S staining, oil-red-O staining and collagen II staining, respectively. Human peripheral blood mononuclear cells (PBMCs)-sorted monocytes, using a CD14 antibody-conjugated magnetic bead system (Miltenyi Biotec, Bergisch Gladbach, Germany) were cultured toward osteoclasts as described before^67^. Cell lines were tested negative for mycoplasma, both by the company and in-house during the culture experiments described in this manuscript. Total RNA was isolated using Trizol (Life Technologies, Carlsbad, CA, USA) after 0, 1, 4, 7, 17 and 21 days of differentiation^68^.

### Mouse-model surveys

Genes prioritized either by location, function DEPICT, eQTL or Capture C analyses were searched in both the Mouse Genome Informatics^26^ (MGI; http://www.informatics.jax.org) and the International Mouse Phenotyping Consortium^27^ (IMPC, https://www.mousephenotype.org/) surveys. Gene expression profiles of candidate genes were examined in primary mouse osteoblasts undergoing differentiation and bone marrow-derived osteoclasts. To study murine osteoblasts, pre-osteoblast-like cells were obtained from neonatal calvaria collected from C57BL/6 J. Next Generation RNA sequencing using an Illumina HiSeq 2000 was used to evaluate the transcriptome every two days from day 2 to 18 days post osteoblast differentiation^13^. Expression of genes in murine osteoclasts was determined using publicly available data obtained using Next-Gen RNA-sequencing applied to bone marrow-derived osteoclasts obtained from 6–8-week-old C57BL/6 mice^69^. All procedures and use of mice for the neonatal osteoblast expression studies were approved by the Jackson Laboratory Animal Care and Use Committee (ACUC), in accordance with NIH guidelines for the care and use of laboratory animals.

### Functional assessment of SK-BMD genes in mutant zebrafish models

All animal experiments in zebrafish were ethically approved by the University of Bristol Animal Welfare and Ethical Review Body (AWERB) and conducted under a UK Home Office project license.

#### Zebrafish CRISPR/Cas9

We used three synthetic guide (g) RNAs (ordered as crispr (cr) RNAs, Sigma) targeting the most plausible orthologs of all three human genes in zebrafish (*zic1; atp6v1c1a* and *atp6v1c1b;* prkar1aa *and prkar1ab)* (Supplementary Table 11). For *zic1*, we used three crRNAs (2 pg), while for the other genes with more than one gene in zebrafish, we concomitantly targeted all orthologs using six crRNAs. crRNAs were incubated with trans-activating (tra) crRNA (10 pg) and GeneArt Platinum Cas9 nuclease (Invitrogen) prior to injections. Injections (1 nl) were performed into 1-cell stage of embryos of the osteoblast reporter lines Tg(*osx:NTR-mCherry*)^70^ or Tg(Ola.*Sp7*:NLS-*gfp*)^71^. Osterix (Osx or Sp7) is a marker of osteoblast maturation. To validate CRISPR efficiency (90%), DNA was extracted from 12 individual larvae injected at 5dpf (days post-fertilization), followed by PCR amplification using FAM-M13F primer and gene-specific primers, with each forward primer containing an M13 tail (Supplementary Table 12). PCR products were submitted for fragment length analysis (ABI 3500)^72^. Controls were injected with Cas9 protein and SygRNA^®^ SpCas9 tracrRNA (10 pg) (Merck). To validate CRISPR efficiency, DNA was extracted at 5dpf (days post-fertilization) from 12 individuals as well as from a pool of 8 injected larvae, followed by PCR amplification using FAM-M13F primer and gene-specific primers, with each forward primer containing an M13 tail (Supplementary Table 12). PCR products underwent fragment length analysis (ABI 3500). As 90% of the 12 individually analyzed fish displayed multiple amplicon peaks, our indel (insertions and deletions) efficiency was 90%. The mosaicism rate was estimated from the pools of 8 fish using the Somatic tissue Activity Test (CRISPR-STAT)^72^. The fold change was calculated by dividing the wt peak between uninjected and injected pools (Supplementary Fig. 7). All the genes targeted in our study showed fold change above 5. *Bone phenotyping in zebrafish: Microscopy and histomorphometry:* Juvenile Tg(*osx:NTR-mCherry*) or Tg(Ola.*Sp7*:NLS-*gfp*) crispants (G0s, injected fish) were briefly anesthetized using tricaine (MS222) followed by live imaging of their skulls using a Leica Fluorescent microscope and LasX software. Ex vivo Alizarin Red staining was performed in two-month fish. Animals were euthanized and fixed in 4% PFA followed by an acid-free bone staining, as previously described^73^. *Zebrafish Micro Computed Tomography (µCT)*: A total of 21 two-month-old fish were fixed in 4% PFA for 7 days, dehydrated in 70% ethanol solution and scanned using a 1172 SkyScan *µ*CT scanner (Bruker, Kontich, Belgium) at pixel-size of 12 *µ*m (scan settings 49 kV, 100 *µ*A, filter Al 0.25 mm). Images were reconstructed using NRecon Software (Bruker). BMD was measured from whole-body and skull-only regions using CTan Software (Bruker), previously calibrated to the phantoms with known mineral density (0.25 and 0.75 g.cm^−3^ hydroxyapatite, Bruker). BMD was compared between groups displaying similar body lengths. *prkar1a* crispants were smaller and therefore were compared with controls matched by size (standard length). A representative skull from each studied group was re-scanned at a higher resolution (pixel size of 3 *µ*m). Amira 6.0 (FEI, Thermo Fisher Scientific) was used to generate 3D volume renders with the same parameters for each group.

### Statistics and reproducibility

Statistical methods have been meticulously described in the methods above.

### Ethics approval and consent to participate

Specific ethics approval and exclusion criteria (where applicable) for each of the studies participating in this meta-analysis can be found in the section cohort description in the Supplementary Material.

### Reporting summary

Further information on research design is available in the Nature Portfolio Reporting Summary linked to this article.

## Supplementary information

Rivadeneira_Peer Review File

Supplementary Information

Description of Additional Supplementary Files

Supplementary Data 1

Supplementary Data 2

Supplementary Data 3

Supplementary Data 4

Supplementary Data 5

Supplementary Data 6

Supplementary Data 7

Reporting Summary

## Data Availability

The significant results of the GWAS meta-analysis generated during this study are included in the supplementary tables of this manuscript. Full GWAS summary data is available on the GEFOS website (www.gefos.org). In the same website, readers will have access to the Osteoclast eQTL data. Data regarding MSC-derived osteoblasts can be accessed in ArrayExpress (https://www.ebi.ac.uk/biostudies/arrayexpress/studies) with the following accession numbers: Capture C: “E-MTAB-6862” and ATAC-seq: “E-MTAB-6834” (MSC-derived osteoblasts). Through the NCBI-GEO readers can have access to both, RNA expression of human mesenchymal stem cells data can be accessed (accession number GSE80614) and gene expression in calvarial osteoblasts from neonatal C57BL/6 J (accession number GSE54461). The RNA-Seq data of the primary bone tissue are publicly available at SRA (accession number: PRJNA764663). All other data are available from the corresponding author (or other sources, as applicable) on reasonable request.

## Code availability

We used publicly available software for analyses included in this study. Details about them including (where applicable) the computer codes are available in the following URLs:

METAL (https://genome.sph.umich.edu/wiki/METAL_Documentation)

GCTA (https://yanglab.westlake.edu.cn/software/gcta/#Overview)

LOCUSZOOM (http://locuszoom.org/)

DEPICT (https://github.com/perslab/depict)

GARFIELD (https://www.ebi.ac.uk/birney-srv/GARFIELD/)

LDSC (https://github.com/bulik/ldsc)

SMR (https://yanglab.westlake.edu.cn/software/smr/#Overview).

Other codes are available on reasonable request.
