# Supplementary material for: Bone mineral density loci specific to the skull portray potential pleiotropic effects on craniosynostosis
Source: Commun Biol. 2023 Jul 4;6:691. doi: 10.1038/s42003-023-04869-0 (PMC10319806; doi:10.1038/s42003-023-04869-0)
Supplement: Supplementary file 2 — Supplementary Information [file 42003_2023_4869_MOESM2_ESM.pdf]

Supplementary Information

Bone Mineral Density Loci Specific to the Skull Portray Potential Pleiotropic Effects On Craniosynostosis

Medina-Gomez et al.

Cohorts short description 2
Supplementary acknowledgments 7
Supplementary tables 13
Supplementary figures 33
Supplementary references 42

Cohorts short description

Avon Longitudinal Study of Parents and their Children (ALSPAC):

The Avon Longitudinal Study of Parents and their Children (ALSPAC) is a longitudinal population-based birth cohort that recruited pregnant women residing in Avon, UK, with an expected delivery date between 1st April 1991 and 31st December 1992. The initial number of pregnancies enrolled was 14,541. When the oldest children were approximately 7 years of age, an attempt was made to bolster the initial sample with eligible cases who had failed to join the study originally. The total sample size for analyses after the age of seven was 15,454 pregnancies, resulting in 15, 589 foetuses. Of these 14,901 were alive at one year of age. The following manuscript contains the results of ALSPAC individuals who were measured on DXA at the age 9 FOCUS group and have GWAS. Ethical approval for the study was obtained from the ALSPAC Ethics and Law Committee and the Local Research Ethics Committees. Consent for biological samples has been collected in accordance with the Human Tissue Act (2004). Informed consent for use of data collected via questionnaires and clinics was obtained from participants following the recommendations of the ALSPAC Ethics and Law Committee at the time. This cohort is described in detail on the website (http://www.alspac.bris.ac.uk) and elsewhere [1-3] and the total body DXA measures and cohort analyzed in the present paper are described in Kemp et al. (2014) [4]. Please note that the study website contains details of all the data that is available through a fully searchable data dictionary and variable search tool (http://www.bris.ac.uk/alspac/researchers/our-data).

Bone Mineral Density in Childhood Study (BMDCS):

The Bone Mineral Density in Childhood Study is an ongoing longitudinal study in which boys and girls aged 6-16 year old were recruited between 2002-2003, and whose DXA measurements are obtained annually at five clinical centers in the United States [5,6].

BPROOF:

B-PROOF is a trial investigating the effect of 2-year supplementation with 400 mcg folic acid and 500 mcg vitamin B12 on fracture incidence in hyperhomoycsteinemic persons aged 65y and older. The study samples have been previously described in detail[7]. All participants gave informed consent and he WU Medical Ethics Committee approved the study protocol, and the Medical Ethics committees of EMC and VUmc gave approval for local feasibility.

Copenhagen Prospective Studies on Asthma (COPSAC) cohort:

The Copenhagen Prospective Studies on Asthma in Childhood is a clinical study. $COPSAC_{2000}$ is a mother child cohort where all the mothers had a history of a doctor's diagnosis of asthma after 7 years of age and thus this is a high risk asthma cohort comprising 411 children. Newborns were enrolled in the first month of life, as previously described in detail[8]. $COPSAC_{2010}$ is a mother child cohort comprising 700 children born to unselected mothers from Denmark as described previously in detail[9,10]. The studies

were conducted in accordance with the guiding principles of the Declaration of Helsinki and was approved by the Local Ethics Committee (COPSAC2000: KF 01-289/96, COPSAC2010: H-B-2008-093), and the Danish Data Protection Agency (COPSAC2000 and COPSAC2010: 2015-41-3696). Both parents provided written informed consent before enrolment. The Ethics Committee for Copenhagen and the Danish Data Protection Agency approved this study.

deCODE genetics BMD study:

The deCODE genetics BMD study is an ongoing population based study of all subjects who have undergone a DEXA-Hologic bone mineral density scan at the Landspitali University Hospital, Reykjavik, Iceland. The study samples have been previously described in detail [11]. All participants gave informed consent and the study was approved by the Data Protection Commission of Iceland and the National Bioethics Committee of Iceland.

ERF:

Erasmus Rucphen Family study (ERF) is a family-based cohort study that includes inhabitants of a genetically isolated community in the South-West of the Netherlands, studied as part of the Genetic Research in Isolated Population (GRIP) program. ERF includes over 3,000 individuals who are living descendants of 22 couples, who had at least six children baptized in the community church, and their spouses. All data were collected between June 2002 and February 2005. The population shows minimal immigration and high inbreeding, therefore frequency of rare alleles is increased in this population. All participants gave informed consent, and the Medical Ethics Committee of the Erasmus University Medical Centre, approved the study.

The Generation R Study:

The Generation R Study is a multiethnic prospective cohort study in which 9,778 pregnant women living in Rotterdam and with delivery date from April 2002 until January 2006 were enrolled. Details of study design and data collection can be found elsewhere [12]. Genotype and imputation of this cohort are described elsewhere [13].

GOOD Study:

The Gothenburg Osteoporosis and Obesity Determinants (GOOD) study was initiated to determine both environmental and genetic factors involved in the regulation of bone and fat mass. The GOOD study is a population-based cohort in which male subjects from between 18 and 20 years of age in the Gothenburg area in Sweden were randomly selected using national population registers and invited to participate in this initiative by phone. From the selected candidates 1,068 agreed to participate providing oral and written informed consent. The GOOD study was approved by the local ethics committee at Gothenburg University [14].

MROS USA:

The Osteoporotic Fractures in Men (MrOS) Study is a multi-center prospective, longitudinal, observational study of risk factors for vertebral and all non-vertebral fractures in older men, and of the sequelae of fractures in men [15,16]. The original specific aims of the study include: (1) to define the skeletal determinants of fracture risk in older men, (2) to define lifestyle and medical factors related to fracture risk, (3) to establish the contribution of fall frequency to fracture risk in older men, (4) to determine to what extent androgen and estrogen concentrations influence fracture risk, (5) to examine the effects of fractures on quality of life, (6) to identify sex differences in the predictors and outcomes of fracture, (7) to collect and store serum, urine and DNA for future analyses as directed by emerging evidence in the fields of aging and skeletal health, and (8) define the extent to which bone mass/fracture risk and prostate diseases are linked. The MrOS Study enrolled 5,994 community dwelling, ambulatory men aged 65 years or older from six communities in the United States (Birmingham, AL; Minneapolis, MN; Palo Alto, CA; Monongahela Valley near Pittsburgh, PA; Portland, OR; and San Diego, CA) between 2000 and 2002. Inclusion criteria were designed to provide a study cohort that is representative of the broad population of older men. The inclusion criteria were: (1) ability to walk without the assistance of another, (2) absence of bilateral hip replacements, (3) ability to provide self-reported data, (4) residence near a clinical site for the duration of the study, (5) absence of a medical condition that (in the judgment of the investigator) would result in imminent death, and (6) ability to understand and sign an informed consent. To qualify as an enrollee, the participant had to provide written informed consent, complete the self-administered questionnaire (SAQ), attend the clinic visit, and complete at least the anthropometric, DXA, and vertebral X-ray procedures. There were no other exclusion criteria. Written informed consent was obtained from all participants, and the Institutional Review Board at each study site approved the study.

Whole body total BMD (g/cm^2) and head BMD (g/cm^2) was measured using dual energy x-ray absorptiometry (DXA) (Hologic, Inc., MA) using Hologic QDR 4500 workstations at the baseline clinic visit. A central quality control lab, certification of DXA operators, and standardized procedures for scanning were used to insure reproducibility of DXA measurements. At baseline, a Hologic whole body phantom was circulated and measured at the 6 clinical sites. The variability across clinics was within acceptable limits, and cross-calibration correction factors were not required.

NEO:

The NEO was designed for extensive phenotyping to investigate pathways that lead to obesity-related diseases. The NEO study is a population-based, prospective cohort study that includes 6,671 individuals aged 45–65 years, with an oversampling of individuals with overweight or obesity. At baseline, information on demography, lifestyle, and medical history have been collected by questionnaires. In addition, samples of 24-h urine, fasting and postprandial blood plasma and serum, and DNA were collected.

OPRA:

The Osteoporosis Risk Assessment Cohort (OPRA) cohort recruited Swedish women aged 75, at which time age-related bone loss is already obvious and fractures prevalent. The study was designed to investigate genetic and lifestyle factors contributing to osteoporosis and fracture risk. Of 1604 women invited between December 1995 and May 1999, 1044 (65%) attended at baseline. No exclusion criteria were applied. All participants answered a detailed questionnaire regarding their general health; BMD and body composition was assessed by DXA. All participants gave written informed consent and the Lund University Ethics Committee approved the study.

ORCADES:

The Orkney Complex Disease Study is an ongoing family-based genetic epidemiology collection in the isolated Scottish archipelago of Orkney. Genetic diversity in this population is decreased compared to Mainland Scotland, consistent with the high levels of endogamy historically. Fasting blood samples were collected and over 300 health-related phenotypes and environmental exposures were measured in each individual. All participants gave informed consent and the study was approved by Research Ethics Committees in Orkney and Aberdeen.

PANIC:

The Physical Activity and Nutrition in Children (PANIC) study is a controlled physical activity and dietary intervention study in a population sample of 506 Finnish children aged 6-8 years at baseline in 2007-2009. Ethical approval was obtained from the Research Ethics Committee of the Hospital District of Northern Savo. All children and their parents gave their written informed consent [17]. (http://www.uef.fi/en/web/physical-activity-and-nutrition-in-children/home)

RAINE:

The Raine (West Australian Pregnancy Cohort) Study is a longitudinal population-based pregnancy cohort study, which recruited 2,900 pregnant women from the public antenatal clinic at King Edward Memorial Hospital and surrounding private clinics in Perth, Western Australia between May 1989 and November 1991 [18]. Of the 2868 live births, 1183 had a whole body DXA at 20 years [19].

Rotterdam Study:

The Rotterdam Study is a prospective cohort study of chronic disabling conditions in Dutch elderly individuals that started in 1990 in Ommoord, a suburb of Rotterdam, among 10,994, men and women aged 55 and over [20].

SOF:

The Study of Osteoporotic Fractures (SOF) is a prospective multicenter study of risk factors for vertebral and non-vertebral fractures [21]. The cohort is comprised of 9,704 community-dwelling women 65 years

old or older recruited from populations-based listings in four U.S. areas: Baltimore, Maryland; Minneapolis, Minnesota; Portland, Oregon; and the Monongahela Valley, Pennsylvania. The SOF participants were followed up every four months by postcard or telephone to ascertain the occurrence of falls, fractures and changes in address. To date, follow-up rates have exceeded 95% for vital status and fractures. All fractures are validated by x-ray reports or, in the case of most hip fractures, a review of pre-operative radiographs. The inclusion criteria were: 1) 65 years or older, (2) ability to walk without the assistance of another, (3) absence of bilateral hip replacements, (4) ability to provide self-reported data, (5) residence near a clinical site for the duration of the study, (6) absence of a medical condition that (in the judgment of the investigator) would result in imminent death, and (7) ability to understand and sign an informed consent.

This study used whole body total BMD (g/cm^2) and head BMD (g/cm^2) measured using dual energy x-ray absorptiometry (DXA) (Hologic, Inc., MA) using Hologic QDR 2000 workstations at the sixth clinic visit. Scans were performed and analyzed at each clinic. Review of scans was done at the UCSF Coordinating Center on random subsets of scans and on problematic scans identified by technicians at the clinic. Some scans were deemed unacceptable and are not included in the data or are set to a special missing value code.

TwinsUK:

The UK Adult Twin Registry (TwinsUK) (www.twinsuk.ac.uk/) was started in 1993 and is comprised of ~12,000 monozygotic and dizygotic twins (83% female) aged 16-85 years recruited by successive media campaigns from all over the UK without selection for any particular disease or trait. The cohort is from Northern European/UK ancestry and has been shown to be representative of singleton populations and the UK population in general (26). All twins received a series of detailed disease and environmental questionnaires and the majority have been assessed in detail clinically at several time points for several hundred phenotypes related to common diseases or intermediate traits. The primary focus of the study has been the genetic basis of healthy aging process and complex diseases, including cardiovascular, metabolic, musculoskeletal, and ophthalmologic disorders.

UK-Biobank Study:

In 2006-2010, the UK Biobank recruited 502,647 individuals aged between 37-76 years (99.5% were 40-69 years) from across the country. Each participant provided information regarding their health and lifestyle using touch screen questionnaires, physical measurements and agreement to have their health followed and they also provided blood, urine and saliva samples for future analysis. UK Biobank has ethical approval from the Northwest Multi-centre Research Ethics Committee (MREC) and informed consent was obtained from all participants.

Supplementary acknowledgments

The authors would like to thank the many colleagues who contributed to collection and phenotypic characterization of the clinical samples, as well as genotyping and analysis of the GWAS data. Full details of acknowledgements are provided below.

ALSPAC Study:

We are extremely grateful to all the families who took part in this study, the midwives for their help in recruiting them, and the whole ALSPAC team, which includes interviewers, computer and laboratory technicians, clerical workers, research scientists, volunteers, managers, receptionists, and nurses. GWAS data was generated by Sample Logistics and Genotyping Facilities at the Wellcome Trust Sanger Institute and LabCorp (Laboratory Corporation of America) using support from 23andMe. The UK Medical Research Council and the Wellcome Trust (Grant ref: 217065/Z/19/Z) and the University of Bristol provide core support for ALSPAC. This publication is the work of the authors, and JPK and DME will serve as guarantors for the contents of this paper. This work is supported by a Medical Research Council program grant (MC_UU_12013/4 to D.M.E). D.M.E is supported by an NHMRC Senior Research Fellowship (APP1137714). J.P.K is funded by a National Health and Medical Research Council (Australia) Investigator grant (GNT1177938)

The Bone Mineral Density in Childhood Study:

BMDCS is extremely grateful to all the families who participated in this study, and the whole team, which includes interviewers, computer and laboratory technicians, clerical workers, research scientists, volunteers, managers, receptionists, and nurses. This work was funded by the National Institute of Child Health and Human Development (NICHD) contracts NO1-HD-1-3228, -3329, -3330, -3331, -3332 and -3333, R01 HD058886 and the Clinical and Translational Research Center (5-MO1-RR-000240 and UL1 RR-026314).

BPROOF:

The authors gratefully thank all study participants, and all co-workers who helped to succeed this trial, especially P.H. in 't Veld, M. Hillen-Tijdink, A. Nicolaas-Merkus, N. Pliester, S. Oliai Araghi, and S. Smits. They also thank Prof. Dr. H.A.P. Pols for obtaining funding. B-PROOF is supported and funded by The Netherlands Organization for Health Research and Development (ZonMw, Grant 6130.0031), The Hague; unrestricted grant from NZO (Dutch Dairy Association), Zoetermeer; NCHA (Netherlands Consortium Healthy Ageing) Leiden/ Rotterdam; Ministry of Economic Affairs, Agriculture and Innovation (project KB-15-004-003), the Hague; Wageningen University, Wageningen; VU University Medical Center, Amsterdam; Erasmus MC, Rotterdam.

CHS:

Cardiovascular Health Study: This CHS research was supported by NHLBI contracts HHSN268201200036C, HHSN268200800007C, N01HC55222, N01HC85079, N01HC85080, N01HC85081, N01HC85082, N01HC85083, N01HC85086; and NHLBI grants U01HL080295, R01HL087652, R01HL105756, R01HL103612, R01HL120393, and R01HL130114 with additional contribution from the National Institute of Neurological Disorders and Stroke (NINDS). Additional support was provided through R01AG023629 from the National Institute on Aging (NIA). A full list of principal CHS investigators and institutions can be found at CHS-NHLBI.org. The provision of genotyping data was supported in part by the National Center for Advancing Translational Sciences, CTSI grant UL1TR000124, and the National Institute of Diabetes and Digestive and Kidney Disease Diabetes Research Center (DRC) grant DK063491 to the Southern California Diabetes Endocrinology Research Center.The content is solely the responsibility of the authors and does not necessarily represent the official views of the National Institutes of Health.

COPSAC:

We express our gratitude to the participants of the COPSAC2000, COPSAC2010 and COPSAC-REGISTRY cohort study for all their support and commitment. We also acknowledge and appreciate the unique efforts of the COPSAC research team.

deCODE:

We thank all the study participants their participation, the staff at deCODE genetics core facilities and the staff at the Research Service Center. deCODE genetics funded the study.

ERF:

We are grateful to all study participants and their relatives, general practitioners and neurologists for their contributions and to P. Veraart for her help in genealogy, J. Vergeer for the supervision of the laboratory work and P. Snijders for his help in data collection. Erasmus Rucphen family study: The ERF study as a part of EUROSPAN (European Special Populations Research Network) was supported by European Commission FP6 STRP grant number 018947 (LSHG-CT-2006-01947) and also received funding from the European Community's Seventh Framework Programme (FP7/2007-2013)/grant agreement HEALTH-F4-2007-201413 by the European Commission under the programme "Quality of Life and Management of the Living Resources" of 5th Framework Programme (no. QLG2-CT-2002-01254). High-throughput analysis of the ERF data was supported by a joint grant from the Netherlands Organization for Scientific Research and the Russian Foundation for Basic Research (NWO-RFBR 047.017.043). Exome sequencing analysis in ERF was supported by the ZonMw grant (project 91111025). Najaf Amin is supported by the Netherlands Brain Foundation (project number F2013(1)-28).

The Generation R Study:

We gratefully acknowledge the contribution of children and parents, general practitioners, hospitals, midwives and pharmacies in Rotterdam. The generation and management of GWAS genotype data for the Generation R Study was done at the Genetic Laboratory of the Department of Internal Medicine,

Erasmus MC, The Netherlands. We thank Pascal Arp, Mila Jhamai, Marijn Verkerk, Lizbeth Herrera and Marjolein Peters for their help in creating, managing and QC of the GWAS database. The musculoskeletal research of the Generation R Study is partly supported by the European Commission grant HEALTH-F2-2008-201865-GEFOS. The general design of Generation R Study is made possible by financial support from Erasmus MC, University Medical Center Rotterdam, Rotterdam, Rotterdam, Erasmus University Rotterdam, the Netherlands Organization for Health Research and Development (ZonMw), the Netherlands Organisation for Scientific Research (NWO), the Ministry of Health, Welfare and Sport and the Ministry of Youth and Families. Additionally, the Netherlands Organization for Health Research and Development supported authors of this manuscript (ZonMw 907.00303, ZonMw 916.10159). F.R is funded by the LEGENDARE ERC Grant (ERC-2020-ADG).

GOOD Study:

Financial support was received from the Swedish Research Council (K2010- 54X-09894-19-3, 2006-3832 and K2010-52X-20229-05-3),the Swedish Foundation for Strategic Research, the ALF/LUA research grant in Gothenburg, the Lundberg Foundation, the Torsten and Ragnar Söderberg's Foundation, the Västra Götaland Foundation, the Göteborg Medical Society, the Novo Nordisk foundation, and the European Commission grant HEALTH-F2-2008-201865-GEFOS.

MROS-USA:

The Osteoporotic Fractures in Men (MrOS) Study is supported by National Institutes of Health funding. The following institutes provide support: The National Institute on Aging (NIA), the National Institute of Arthritis and Musculoskeletal and Skin Diseases (NIAMS), the National Center for Advancing Translational Sciences (NCATS), and NIH Roadmap for Medical Research under the following grant numbers: U01 AG027810, U01 AG042124, U01 AG042139, U01 AG042140, U01 AG042143, U01 AG042145, U01 AG042168, U01 AR066160, and UL1 TR000128. NIAMS provided funding for the MrOS ancillary study ‘Replication of candidate gene associations and bone strength phenotype in MrOS’ under the grant number R01 AR051124 and the MrOS ancillary study ‘GWAS in MrOS and SOF’ under the grant number RC2 AR058973.

NEO:

The authors of the NEO study thank all individuals who participated in the Netherlands Epidemiology in Obesity study, all participating general practitioners for inviting eligible participants and all research nurses for collection of the data. We thank the NEO study group, Pat van Beelen, Petra Noordijk and Ingeborg de Jonge for the coordination, lab and data management of the NEO study. The genotyping in the NEO study was supported by the Centre National de Génotypage (Paris, France), headed by Jean-Francois Deleuze. The NEO study is supported by the participating Departments, the Division and the Board of Directors of the Leiden University Medical Center, and by the Leiden University, Research Profile Area Vascular and Regenerative Medicine. Dennis Mook-Kanamori is supported by Dutch Science Organization (ZonMW-VENI Grant 916.14.023).

OPRA:

This work was supported by grants from the Swedish Research Council (K2015-52X-14691-13-4), Forte (Grant 2007–2125), Greta and Johan Kock Foundation, A. Påhlsson Foundation, A. Osterlund Foundation, H Järnhardt foundation, King Gustav V 80 year fund, King Gustav V and Queen Victoria Foundation, Åke Wiberg Foundation, Thelma Zoegas Foundation, Swedish Rheumatism foundation, Skåne University Hospital Research Fund and the Research and Development Council of Region Skåne, Sweden.

Thanks are extended to the research nurses at the Clinical and Molecular Osteoporosis Research Unit, Malmö; Åsa Almgren for data management, Olle Melander for GWAS genotyping (performed at Lund University, Dept of Clinical Sciences Malmö, Sweden) and to all the women who kindly participated in the study.

ORCADES:

ORCADES was supported by the Chief Scientist Office of the Scottish Government (CZB/4/276, CZB/4/710), the Royal Society, the MRC Human Genetics Unit, Arthritis Research UK and the European Union framework program 6 EUROSPAN project (contract no. LSHG-CT-2006-018947). DNA extractions were performed at the Wellcome Trust Clinical Research Facility in Edinburgh. We would like to acknowledge the invaluable contributions of the research nurses in Orkney, the administrative team in Edinburgh and the people of Orkney.

PANIC:

We are grateful to the parents and children for participating in the PANIC study and to the whole research team for their contribution in carrying out the study. The PANIC study was financially supported by grants from Ministry of Social Affairs and Health of Finland, Ministry of Education and Culture of Finland, Finnish Innovation Fund Sitra, Social Insurance Institution of Finland, Finnish Cultural Foundation, Juha Vainio Foundation, Foundation for Pediatric Research, Paavo Nurmi Foundation, Paulo Foundation, Diabetes Research Foundation, Yrjo Jahnsson Foundation, Finnish Foundation for Cardiovascular Research, State Research Funding from Research Committee of Kuopio University Hospital Catchment Area, Kuopio University Hospital EVO Funding, National Doctoral Programs, and the City of Kuopio.

RAINE:

We thank the Raine Study participants and families for being part of the study, the Raine Study Team for cohort management and data collection. The Raine Study receives core funding support from the University of Western Australia -Faculty of Medicine, Dentistry and Health Sciences, Curtin University, the Raine Medical Research Foundation, the Women and Infants Research Foundation, Telethon Kids Institute and Edith Cowan University. The study was funded by the National Health and Medical Research Council of Australia [grant numbers 572613, 403981 and 003209], the Canadian Institutes of Health Research [grant number MOP-82893] and the Lions Eye Institute in WA. This work was supported by resources provided by the Pawsey Supercomputing Centre with funding from the Australian Government and the Government of Western Australia.

Rotterdam Study:

The generation and management of GWAS genotype data for the Rotterdam Study (RS I, RS II, RS III) was executed by the Human Genotyping Facility of the Genetic Laboratory of the Department of Internal Medicine, Erasmus MC, Rotterdam, The Netherlands. The GWAS datasets are supported by the Netherlands Organisation of Scientific Research NWO Investments (nr. 175.010.2005.011, 911-03-012), the Genetic Laboratory of the Department of Internal Medicine, Erasmus MC, the Research Institute for Diseases in the Elderly (014-93-015; RIDE2), the Netherlands Genomics Initiative (NGI)/Netherlands Organisation for Scientific Research (NWO) Netherlands Consortium for Healthy Aging (NCHA), project nr. 050-060-810. We thank Pascal Arp, Mila Jhamai, Marijn Verkerk, Lizbeth Herrera and Marjolein Peters, PhD, and Carolina Medina-Gomez, MSc, for their help in creating the GWAS database, and Karol Estrada, PhD, Yurii Aulchenko, PhD, and Carolina Medina-Gomez, PhD, for the creation and analysis of imputed data. Dr. Fernando Rivadeneira received an additional grant from the Netherlands Organization for Health Research and Development ZonMw VIDI 016.136.367. The Rotterdam Study is funded by Erasmus Medical Center and Erasmus University, Rotterdam, Netherlands Organization for the Health Research and Development (ZonMw), the Research Institute for Diseases in the Elderly (RIDE), the Ministry of Education, Culture and Science, the Ministry for Health, Welfare and Sports, the European Commission (DG XII), and the Municipality of Rotterdam. The authors are very grateful to the study participants, the staff from the Rotterdam Study (particularly L. Buist and J.H. van den Boogert) and the participating general practitioners and pharmacists.

SOF:

The Study of Osteoporotic Fractures (SOF) is supported by National Institutes of Health funding. The National Institute on Aging (NIA) provides support under the following grant numbers: R01 AG005407, R01 AR35582, R01 AR35583, R01 AR35584, R01 AG005394, R01 AG027574, and R01 AG027576. The National Institute of Arthritis and Musculoskeletal and Skin Diseases (NIAMS) provides funding for the SOF ancillary study 'GWAS in MrOS and SOF' under the grant number RC2AR058973.

Twins UK:

We gratefully acknowledge the help and support of the twin volunteers. TwinsUK is funded by the Wellcome Trust, Medical Research Council, European Union, the National Institute for Health Research (NIHR)-funded BioResource, Clinical Research Facility and Biomedical Research Centre based at Guy's and St Thomas' NHS Foundation Trust in partnership with King's College London. TDS is holder of an ERC Advanced Principal Investigator award. SNP Genotyping was performed by The Wellcome Trust Sanger Institute and National Eye Institute via NIH/CIDR for TwinsUK. The study also received support from the Australian National Health and Medical Research Council (Project Grant 1048216) and The Pawsey Supercomputing Centre (with Funding from the Australian Government and the Government of Western Australia; PG 16/0162, PG 17/director2025).

Functional Group:

C. Ackert-Bicknell is supported by the National Institute of Health /National Institute of Arthritis Musculoskeletal and Skin Diseases grant number AR060981. E. Krague is supported by Versus Arthritis, grant #21937.

Supplementary tables

Supplementary Table 1. Independent SNPs associated with SK-BMD. Genomic coordinates are on build 37 of the human genome. Craniosynostosis (CS). Beta coefficients and allele frequencies (EAF) are reported for the A1 allele. Jsufix refers to the summary statistics in the join analysis fitting all SNPs together. Var. exp.= 2xbeta^{2}xEAFx(1-EAF) [22]. SK=skull BMD , assessed in this study; LS=lumbar spine BMD (Zheng et al.[23]); FN=Femoral Neck BMD (Zheng et al. [23]); eBMD = heel BMD (Morris et al. [24]).

Marker Description								Skull BMD						FN-BMD		LS-BMD		e-BMD	
CHR	BP	locus	CS-gene present	closest_gene	rsnumber	A1	EAF	beta	P	betaJ	PJ	locus	var. exp.	beta_FN	P_FN	beta_LS	P_LS	beta_eBMD	P_eBMD
1	22451845	1p36.12	no	WNT4	rs7526484	T	0.24	0.075	1.47E-19	0.057	1.89E-11	1	0.0021	0.026	0.004	0.018	0.094	0.022	3.00E-25
1	22484575	1p36.12	no	WNT4	rs3971300	T	0.71	0.109	5.70E-43	0.101	3.26E-36	1	0.0048	0.041	3.27E-06	0.039	1.44E-04	0.039	4.10E-88
1	22692315	1p36.12	no	ZBTB40	rs12728589	C	0.18	0.088	1.30E-21	0.091	7.14E-23	1	0.0023	0.082	3.04E-16	0.090	3.41E-14	0.061	5.80E-150
1	68634642	1p31.3	no	WLS	rs12132335	A	0.14	-0.059	5.50E-09	-0.074	4.62E-13	2	0.0008	-0.029	0.008	-0.003	0.796	-0.006	0.035
1	68656697	1p31.3	no	WLS	rs2566752	T	0.62	-0.063	2.67E-18	-0.072	3.50E-22	2	0.0019	-0.062	3.65E-15	-0.083	1.49E-19	-0.036	1.30E-78
1	110475552	1p13.3	no	CSF1	rs12132533	A	0.80	0.049	1.88E-08	0.048	2.82E-08	3	0.0008	0.022	0.017	0.022	0.041	0.008	0.007
1	113147607	1p13.2	no	ST7L	rs55652172**	A	0.20	0.050	1.34E-08	0.050	1.85E-08	4	0.0008	0.041	1.33E-05	0.034	0.002	0.032	4.60E-46
1	163875519	1q23.3	no	LOC100422212	rs7550321**	A	0.44	-0.042	1.30E-09	-0.042	1.67E-09	5	0.0009	-0.005	0.534	-0.030	0.001	-0.018	6.20E-24
1	172122809	1q24.3	no	DNM3	rs495590	C	0.54	0.042	7.52E-09	0.041	8.40E-09	6	0.0009	0.025	0.001	0.017	0.062	0.020	1.50E-29
1	220129357	1q41	no	SLC30A10	rs2795312	T	0.65	0.050	3.06E-10	0.050	2.73E-10	7	0.0011	0.017	0.033	0.013	0.153	0.015	1.60E-16
2	40630678	2p22.1	no	SLC8A1	rs10490046	A	0.77	0.064	1.20E-14	0.064	1.07E-14	8	0.0015	0.021	0.025	0.015	0.162	0.029	2.00E-40
2	85498783	2q14.2	no	TCF7L1	rs11675489	A	0.54	0.040	1.63E-08	0.040	1.56E-08	9	0.0008	0.015	0.050	0.023	0.011	0.013	6.20E-12
2	119104991	2p11.2	no	INSIG2	rs72962911	A	0.86	-0.059	7.26E-09	-0.058	1.26E-08	10	0.0008	0.007	0.558	-0.047	3.68E-04	-0.048	1.10E-73
2	119621910	2p11.2	yes	EN1	rs11679232	T	0.28	0.055	2.02E-10	0.054	2.95E-10	10	0.0012	0.002	0.910	0.035	0.067	0.048	4.20E-129
3	41138172	3p22.1	no	CTNNB1	rs428510	T	0.56	0.046	8.56E-11	0.046	1.13E-10	11	0.0010	0.061	2.44E-15	0.055	8.02E-10	0.046	3.50E-146
3	147163978	3q24	yes	ZIC1	rs12107945*	A	0.30	-0.049	1.37E-10	-0.049	1.20E-10	12	0.0010	-0.001	0.903	-0.016	0.097	-2.39E-04	0.730
3	156692207	3q25.31	no	LEKR1	rs74394007	A	0.86	0.062	1.59E-09	0.062	1.69E-09	13	0.0009	0.034	0.003	0.043	0.001	0.046	8.90E-63
4	994414	4p16.3	yes	IDUA	rs3755955	A	0.16	-0.062	1.26E-10	-0.062	1.27E-10	14	0.0010	-0.044	2.87E-05	-0.049	5.05E-05	-0.068	1.90E-167
4	88831249	4q22.1	no	MEPE	rs11934731	A	0.68	-0.069	3.87E-20	-0.069	3.02E-20	15	0.0021	-0.035	1.97E-05	-0.048	5.02E-07	-0.036	6.10E-74
5	72618265	5q13.2	no	FOXD1	rs11948073**	A	0.53	-0.048	3.86E-11	-0.048	4.28E-11	16	0.0011	0.000	0.999	-0.022	0.014	-0.015	1.40E-17
5	112188456	5q22.2	no	REEP5	rs454968	T	0.36	0.047	1.66E-10	0.047	1.53E-10	17	0.0010	0.010	0.235	0.006	0.507	0.013	1.20E-11
5	122860170	5q23.2	no	CSNK1G3	rs9327301	A	0.26	-0.061	4.47E-14	-0.061	4.32E-14	18	0.0014	-0.025	0.005	-0.009	0.357	-0.024	1.80E-30
6	6939532	6p25.1	no	RREB1	rs2490298**	A	0.61	0.042	7.12E-09	0.042	8.85E-09	19	0.0008	-0.006	0.476	-0.005	0.611	0.004	0.011
6	44943982	6p21.1	yes	SUPT3H	rs17423748	T	0.72	0.056	3.00E-12	0.056	2.99E-12	20	0.0013	0.004	0.670	0.028	0.004	0.012	6.60E-08
6	53617914	6p12.1	no	LRRC1	rs10456165**	A	0.22	0.057	3.45E-11	0.057	3.69E-11	21	0.0011	0.006	0.528	0.023	0.034	-0.008	9.00E-05
6	74458737	6q13	no	CD109	rs9447004**	A	0.50	0.039	4.61E-08	0.039	4.49E-08	22	0.0008	0.002	0.765	0.008	0.375	0.024	2.10E-42
6	127047776	6q22.33	no	CENPW	rs1101560	A	0.26	0.063	5.76E-15	0.068	7.91E-17	23	0.0015	0.007	0.420	0.011	0.301	-0.019	6.00E-23
6	127178839	6q22.33	no	CENPW	rs113622822	T	0.04	0.139	2.61E-11	0.133	4.49E-10	23	0.0014	0.037	0.113	0.033	0.222	0.034	8.00E-10
6	127425630	6q22.33	no	RSPO3	rs1963689	T	0.74	-0.069	1.81E-17	-0.055	3.69E-11	23	0.0018	-0.023	0.009	-0.027	0.008	-0.050	4.00E-130
6	130348257	6q23.1	no	L3MBTL3	rs75191738**	T	0.14	-0.059	5.80E-08	-0.061	1.30E-08	24	0.0008	0.021	0.055	-0.003	0.825	0.022	2.50E-19
6	133366810	6q23.2	no	LINC00326	rs13204469	A	0.68	0.109	2.02E-46	0.087	9.63E-26	25	0.0051	-0.023	0.006	-0.020	0.037	-0.038	4.50E-85
6	133641473	6q23.2	no	EYA4	rs11759873	A	0.65	0.089	5.85E-33	0.056	4.42E-12	25	0.0036	0.005	0.492	-0.009	0.324	-0.036	4.00E-83
6	151874122	6q25.1	no	CCDC170	rs9478217	A	0.43	-0.049	3.23E-11	-0.049	2.85E-11	26	0.0012	-0.053	1.23E-11	-0.056	8.03E-10	-0.071	7.0E-328

Marker Description								Skull BMD						FN-BMD		LS-BMD		e-BMD	
CHR	BP	locus	CS-gene present	closest_gene	rsnumber	A1	EAF	beta	P	betaJ	PJ	locus	var. exp.	beta_FN	P_FN	beta_LS	P_LS	beta_eBMD	P_eBMD
7	96121636	7q21.3	no	C7orf76	rs4383904	T	0.33	-0.046	1.91E-09	-0.046	2.00E-09	27	0.0009	-0.063	4.6E-15	-0.059	6.45E-10	-0.045	3.80E-132
7	120790559	7q31.31	no	CPED1	rs56335989	T	0.56	-0.059	4.44E-16	-0.051	1.54E-08	28	0.0017	-0.017	0.032	-0.009	0.316	0.052	1.10E-178
7	120873438	7q31.31	no	CPED1	rs6965195	A	0.37	-0.095	1.17E-37	-0.099	2.12E-27	28	0.0043	-0.016	0.048	-0.018	0.056	0.067	9.70E-291
7	120969769	7q31.31	no	WNT16	rs2908004	A	0.44	0.115	3.07E-57	0.080	3.72E-16	28	0.0065	0.045	1.71E-09	0.039	7.390E-06	0.095	2.10E-592
7	120977734	7q31.31	no	WNT16	rs55963900	A	0.27	0.130	1.95E-59	0.110	7.63E-25	28	0.0066	0.053	3.7E-10	0.073	2.09E-13	0.162	6.30E-1408
8	103919090	8q22.3	no	ATP6v1C1/AZI	rs11993347*	T	0.77	0.059	2.67E-12	0.059	2.47E-12	29	0.0012	0.003	0.721	0.028	0.007	0.004	0.025
8	109396911	8q23.1	no	EMC2	rs10108399**	T	0.35	0.044	3.78E-09	0.044	3.60E-09	30	0.0009	0.003	0.677	0.014	0.139	0.010	6.70E-09
8	120008587	8q24.12	no	COLEC10	rs7842942	T	0.42	0.079	5.36E-28	0.079	3.84E-28	31	0.0031	0.059	5.31E-14	0.080	7.62E-19	0.011	3.00E-10
10	54427825	10q21.1	no	MBL2	rs1373004	T	0.12	-0.092	6.42E-17	-0.092	8.73E-17	32	0.0018	-0.045	2.81E-04	-0.056	1.10E-04	-0.129	2.6E-433
10	132252499	10q26.3	no	GLRX3	rs61863293*#	A	0.35	-0.046	2.80E-08	-0.046	3.24E-08	33	0.0010	-0.004	0.637	-0.013	0.221	0.002	0.160
11	259575	11p15.5	no	PSMD13	rs56257551	T	0.11	0.078	4.47E-10	0.078	4.70E-10	34	0.0012	0.032	0.016	0.035	0.023	-0.001	0.650
11	15903307	11p15.2	yes	SOX6	rs1440705	A	0.29	0.049	9.11E-10	0.049	8.42E-10	35	0.0010	0.022	0.009	0.029	0.004	0.012	1.80E-08
11	27280398	11p14.1	no	CCDC34	rs1351176	T	0.23	-0.035	3.50E-05	0.059	3.19E-09	36	0.0004	0.006	0.477	-0.015	0.154	-0.018	3.80E-16
11	27322158	11p14.1	no	CCDC34	rs10742173	C	0.60	-0.097	7.78E-41	-0.110	6.60E-38	36	0.0045	-0.014	0.079	-0.020	0.030	-0.045	7.30E-133
11	27367731	11p14.1	no	CCDC34	rs11029963	T	0.66	-0.026	0.0003961	-0.057	7.05E-13	36	0.0003	-0.022	0.007	-0.017	0.065	-0.014	1.60E-14
11	27463564	11p14.1	no	LGR4	rs11030009	A	0.49	0.122	9.32E-66	0.111	1.40E-52	36	0.0074	0.008	0.330	0.030	9.71E-04	0.021	1.50E-33
11	35083633	11p13	no	CD44	rs2553773	C	0.43	-0.041	1.07E-08	-0.044	4.23E-10	37	0.0008	-0.015	0.054	-0.015	0.101	-0.028	3.20E-54
11	46815633	11p11.2	no	LRP4	rs4506602	T	0.51	-0.058	2.22E-16	-0.058	3.26E-16	38	0.0017	-0.027	4.58E-04	-0.030	0.001	-0.021	1.80E-33
11	68218290	11q13.2	yes	LRP5	rs11228240	T	0.26	-0.104	5.39E-36	-0.104	3.70E-36	39	0.0042	-0.043	7.22E-07	-0.075	1.58E-13	-0.043	6.60E-101
11	121917163	11q23.3	no	BLID	rs1944461	T	0.60	0.039	3.03E-08	0.039	3.14E-08	40	0.0007	0.014	0.087	0.023	0.014	0.010	3.60E-08
12	28716044	12p11.22	no	CCDC91	rs10843202**	T	0.24	-0.064	1.71E-14	-0.064	1.89E-14	41	0.0015	0.026	0.004	-0.006	0.590	0.022	9.60E-26
12	49224108	12q13.12	yes	DDX23	rs11551274	C	0.03	-0.173	4.41E-17	-0.404	3.51E-35	42	0.0018	-0.019	0.392	-0.093	4.50E-04	-0.095	9.10E-76
12	49248340	12q13.12	yes	ADCY6	rs143348825	A	0.02	-0.037	0.1828	0.392	3.53E-19	42	4.63E-05	0.008	0.788	-0.049	0.143	-0.047	8.00E-13
12	49277680	12q13.12	yes	ADCY6	rs77105837	A	0.94	0.048	0.001873	0.089	3.30E-08	42	0.0002	0.002	0.923	-0.042	0.026	-0.011	0.003
12	49385679	12q13.12	yes	WNT1	rs10875906	T	0.27	0.046	3.23E-08	0.069	4.29E-15	42	0.0008	0.021	0.016	0.046	1.10E-05	0.027	3.80E-40
12	90412755	12q21.33	no	GALNT4	rs4842705	A	0.58	-0.048	1.79E-11	-0.049	8.98E-12	43	0.0011	-0.024	0.002	-0.025	0.006	-0.048	4.50E-156
12	93982332	12q22	no	SOCS2	rs7977788**	A	0.24	0.045	4.78E-08	0.046	2.46E-08	44	0.0007	0.004	0.682	0.002	0.829	0.011	1.70E-07
12	107373522	12q23.3	no	MTERFD3	rs7302653	C	0.51	0.048	1.91E-11	0.048	1.71E-11	45	0.0011	0.026	0.001	0.027	0.002	-0.012	1.50E-10
13	37473386	13q13.3	no	SMAD9	rs493248	T	0.23	0.050	1.27E-09	0.050	9.65E-10	46	0.0009	0.011	0.222	0.022	0.038	0.017	1.00E-15
13	43148546	13q14.11	no	TNFSF11	rs138818878	C	0.96	-0.121	1.87E-08	-0.122	1.33E-08	47	0.0011	-0.085	1.85E-04	-0.145	3.17E-08	-0.058	1.60E-25
16	392318	16p13.3	yes	AXIN1	rs8047501	A	0.49	0.060	2.71E-16	0.060	3.04E-16	48	0.0018	0.051	1.11E-10	0.050	5.24E-08	0.023	2.00E-37
16	87182052	16q24.2	no	C16orf95	rs1984529	A	0.44	-0.044	1.13E-09	-0.044	1.31E-09	49	0.0010	-0.003	0.650	-0.010	0.284	-0.006	1.10E-04
17	837381	17p13.3	no	NXN	rs11650468**	T	0.59	-0.048	2.25E-09	-0.048	1.99E-09	50	0.0011	-0.001	0.878	0.009	0.347	-0.003	0.130
17	2068932	17p11.2	no	SMG6	rs4790881	A	0.70	0.043	2.45E-08	0.043	2.02E-08	51	0.0008	0.050	2.92E-09	0.035	3.41E-04	0.052	2.40E-153
17	41862277	17q21.31	no	C17orf105	rs34814687	A	0.08	0.100	5.66E-13	0.100	6.15E-13	52	0.0014	0.085	1.75E-08	0.092	1.55E-07	0.071	5.20E-97
17	63305939	17q24.1	no	RGS9	rs12941447	A	0.12	-0.066	5.94E-09	-0.068	2.57E-09	53	0.0009	-0.027	0.028	-0.019	0.180	-0.026	3.70E-19
17	63820304	17q24.1	no	CEP112	rs12601958	A	0.69	-0.060	2.06E-15	-0.059	9.52E-15	53	0.0015	-0.008	0.357	-0.022	0.022	-0.010	1.80E-06
17	66453305	17q24.2	no	PRKAR1A	rs71378928*	C	0.23	-0.050	2.67E-09	-0.051	1.71E-09	54	0.0009	0.007	0.434	-0.022	0.040	0.003	0.240
17	67672730	17q24.3	no	MAP2K6	rs2214207**	T	0.60	0.038	1.97E-07	0.041	1.70E-08	55	0.0007	-0.015	0.055	0.000	0.978	0.005	0.043
17	68060451	17q24.3	no	KCNJ16	rs72856781**	A	0.66	-0.049	4.74E-11	-0.049	3.80E-11	56	0.0011	0.008	0.303	0.008	0.392	0.008	1.80E-04
17	68217231	17q24.3	no	KCNJ2	rs236529**	A	0.41	0.049	3.79E-12	0.049	7.69E-12	56	0.0012	-0.005	0.559	0.012	0.203	-0.002	0.210
18	60059716	18q21.33	no	TNFRSF11A	rs6567278	T	0.56	0.048	1.30E-11	0.048	1.55E-11	56	0.0011	0.016	0.036	0.041	7.31E-06	0.018	4.00E-23
20	10640306	20p12.2	yes	JAG1	rs6040061	A	0.49	-0.044	2.17E-10	-0.044	2.52E-10	57	0.0010	-0.030	9.52E-05	-0.052	5.94E-09	-0.031	5.00E-69
20	39088536	20q12	no	MAFB	rs6029124##	T	0.28	0.047	1.60E-08	0.047	1.34E-08	58	0.0009	0.006	0.518	0.029	0.003	0.007	9.60E-05
21	36970350	21q22.12	no	RUNX1	rs9976876	T	0.46	-0.042	7.21E-09	-0.042	8.32E-09	59	0.0009	-0.016	0.041	-0.019	0.031	-0.019	6.50E-26

* Reported for the first time in relation to a bone phenotype

**Reported for the first time in a DXA-Derived site-specific BMD phenotype

Values for e-BMD are reported for rs61861957 (r^2=0.914)

Values for e-BMD are reported for rs6029123 (r^2=0.971)

Supplementary Table 2. Genome-wide significant SNPs with functional deleterious annotation. Genomic coordinates are on build 37 of the human genome. Beta coefficients and allele frequencies (EAF) are reported for the A1 allele. Annotation and CADD scores are extracted from FUMA results. In bold, SNPs with CADD score over 20.

CHR	BP	SNP	A1	A2	EAF	beta	P	Gene	Annotation	CADD scores
4	994414	rs3755955	a	g	0.16	-0.062	1.26E-10	IDUA	nonsynonymous SNV	0.09
4	995868	rs114806891	t	c	0.07	0.091	1.57E-09	IDUA	stopgain	16.19
4	995919	rs6830825	c	g	0.16	-0.057	2.93E-09	IDUA	nonsynonymous SNV	1.504
4	995997	rs6811373	a	g	0.84	0.058	2.70E-09	IDUA	nonsynonymous SNV	4.145
4	996012	rs6831021	c	g	0.16	-0.058	2.69E-09	IDUA	nonsynonymous SNV	0.324
4	996165	rs6831280	a	g	0.16	-0.060	8.81E-10	IDUA	nonsynonymous SNV	0.05
4	996690	rs73066479	a	g	0.16	-0.056	1.63E-08	IDUA	nonsynonymous SNV	2.646
4	1019011	rs4647932	t	c	0.06	0.085	1.05E-08	FGFRL1	nonsynonymous SNV	10.84
7	120776097	rs41281692	c	g	0.55	-0.058	1.60E-15	CPED1	nonsynonymous SNV	1.623
**7**	**120876835**	**rs35793694**	**a**	**g**	**0.92**	**-0.124**	**7.34E-20**	**CPED1**	**nonsynonymous SNV**	**22.9**
**7**	**120969769**	**rs2908004**	**a**	**g**	**0.44**	**0.115**	**3.07E-57**	**WNT16**	**nonsynonymous SNV**	**20.8**
7	120979089	rs2707466	t	c	0.44	0.114	9.24E-57	WNT16	nonsynonymous SNV	16.85
8	119964052	rs2073618	c	g	0.46	-0.064	4.20E-19	TNFRSF11B	nonsynonymous SNV	15.99
**11**	**27390144**	**rs34717439**	**a**	**g**	**0.03**	**-0.133**	**5.22E-11**	**LGR4**	**nonsynonymous SNV**	**23.4**
**11**	**27679916**	**rs6265**	**t**	**c**	**0.19**	**0.052**	**7.16E-09**	**BDNF**	**nonsynonymous SNV**	**24.1**
11	46387868	rs1317826	a	g	0.70	-0.047	3.54E-09	DGKZ	nonsynonymous SNV	1.042
**11**	**46893108**	**rs2306029**	**t**	**c**	**0.46**	**0.055**	**2.36E-14**	**LRP4**	**nonsynonymous SNV**	**21.7**
11	46898771	rs6485702	t	c	0.35	0.055	2.47E-13	LRP4	nonsynonymous SNV	7.073
**11**	**68174189**	**rs4988321**	**a**	**g**	**0.04**	**-0.168**	**6.84E-17**	**LRP5**	**nonsynonymous SNV**	**23.2**
**11**	**68201295**	**rs3736228**	**t**	**c**	**0.15**	**-0.118**	**6.62E-30**	**LRP5**	**nonsynonymous SNV**	**20.9**
12	28605426	rs10771427	a	g	0.77	0.060	1.30E-12	CCDC91	nonsynonymous SNV	9.094
**12**	**49168798**	**rs3730071**	**a**	**c**	**0.03**	**-0.155**	**8.42E-15**	**ADCY6**	**nonsynonymous SNV**	**23.2**
12	49224108	rs11551274	c	g	0.03	-0.173	4.41E-17	DDX23	nonsynonymous SNV	0.768
**12**	**49399132**	**rs1126930**	**c**	**g**	**0.03**	**0.139**	**1.68E-10**	**PRKAG1**	**nonsynonymous SNV**	**22.7**
13	43148546	rs138818878	c	g	0.96	-0.121	1.87E-08	TNFSF11	nonsynonymous SNV	17.61
17	66449122	rs883541	a	g	0.77	0.049	4.70E-09	WIPI1	nonsynonymous SNV	15.93

Supplementary Table 3. SK-BMD Genome-wide significant SNPs conditionally independent from variants previously reported as associated with bone traits. Beta coefficients and allele frequency (EAF) are reported for the A1 allele and gene referring to the closest gene to the particular variant. Highlighted in green background the four novel loci.

Chr	SNP	bp	EA	EAF	b	se	p	n	bC	bC_se	pC	Gene	Loci
1	1:68634642	68634642	A	0.1377	-0.0588	0.0101	5.50E-09	47950.7	-0.0775	0.0092	2.66E-17	WLS	1p31.3
3	3:147103892	147103892	A	0.063	-0.0805	0.0144	2.46E-08	47451.1	-0.0794	0.0144	3.42E-08	ZIC4	3q24
3	3:147107186	147107186	T	0.9369	0.0805	0.0144	2.29E-08	47380.9	0.0793	0.0144	3.53E-08	ZIC4	3q24
3	3:147115775	147115775	T	0.9364	0.0801	0.0144	2.38E-08	47033.6	0.0791	0.0144	3.91E-08	ZIC4	3q24
3	3:147119629	147119629	T	0.0631	-0.0811	0.0144	1.82E-08	47380.4	-0.0801	0.0144	2.63E-08	ZIC4	3q24
3	3:147119740	147119740	T	0.0632	-0.0811	0.0144	1.83E-08	47310.4	-0.0801	0.0144	2.63E-08	ZIC1	3q24
3	3:147121650	147121650	T	0.0632	-0.0814	0.0144	1.64E-08	47310.2	-0.0804	0.0144	2.33E-08	ZIC1	3q24
3	3:147124889	147124889	A	0.0632	-0.0813	0.0144	1.69E-08	47310.3	-0.0803	0.0144	2.37E-08	ZIC1	3q24
3	3:147129882	147129882	A	0.0632	-0.0816	0.0144	1.48E-08	47310.1	-0.0806	0.0144	2.10E-08	ZIC1	3q24
3	3:147134598	147134598	T	0.9372	0.0816	0.0144	1.54E-08	47591.3	0.0805	0.0144	2.23E-08	ZIC1	3q24
3	3:147136172	147136172	A	0.0628	-0.0817	0.0144	1.48E-08	47591.2	-0.0805	0.0144	2.22E-08	ZIC1	3q24
3	3:147136624	147136624	T	0.0624	-0.0833	0.0145	9.79E-09	47216.7	-0.0820	0.0145	1.50E-08	ZIC1	3q24
3	3:147137705:ID	147137705	D	0.0624	-0.1069	0.0177	1.54E-09	31673.3	-0.1057	0.0177	2.35E-09	ZIC1	3q24
3	3:147139626	147139626	T	0.9372	0.0819	0.0144	1.40E-08	47591	0.0807	0.0144	2.01E-08	ZIC1	3q24
3	3:147139741	147139741	T	0.0628	-0.0817	0.0144	1.51E-08	47591.2	-0.0805	0.0144	2.18E-08	ZIC1	3q24
3	3:147145745	147145745	T	0.9373	0.0816	0.0145	1.71E-08	47007	0.0804	0.0145	2.89E-08	ZIC1	3q24
3	3:147151946	147151946	A	0.0595	-0.0836	0.0149	1.96E-08	46751.6	-0.0822	0.0149	3.35E-08	ZIC1	3q24
3	3:147152103	147152103	A	0.06	-0.0833	0.0148	1.98E-08	47015.6	-0.0819	0.0148	3.04E-08	ZIC1	3q24
3	3:147153885	147153885	T	0.7046	0.0483	0.0076	2.38E-10	48305.2	0.0483	0.0076	2.15E-10	ZIC1	3q24
3	3:147157704	147157704	A	0.9399	0.0837	0.0149	1.79E-08	46314.1	0.0823	0.0149	3.29E-08	ZIC1	3q24
3	3:147159244	147159244	A	0.9374	0.0818	0.0145	1.77E-08	47076.9	0.0805	0.0145	2.77E-08	ZIC1	3q24
3	3:147163128	147163128	T	0.2893	-0.0492	0.0078	2.40E-10	46423.9	-0.0488	0.0078	4.11E-10	ZIC1	3q24
3	3:147163978	147163978	A	0.297	-0.0489	0.0076	1.37E-10	48153.2	-0.0489	0.0076	1.30E-10	ZIC1	3q24
3	3:147167274	147167274	A	0.0595	-0.0844	0.0149	1.62E-08	46751	-0.0830	0.0149	2.46E-08	ZIC1	3q24
3	3:147168680:ID	147168680	D	0.0616	-0.1111	0.0182	1.12E-09	30317.8	-0.1098	0.0182	1.59E-09	ZIC1	3q24
3	3:147169704	147169704	C	0.9405	0.0847	0.015	1.46E-08	46129.5	0.0833	0.0150	2.71E-08	ZIC1	3q24
3	3:147173184	147173184	A	0.9373	0.0828	0.0146	1.34E-08	46364.4	0.0815	0.0146	2.31E-08	ZIC1	3q24
3	3:147175718	147175718	T	0.9399	0.0849	0.0149	1.27E-08	46313.1	0.0836	0.0149	2.01E-08	ZIC1	3q24
3	3:147187915	147187915	A	0.9392	0.0858	0.0149	8.03E-09	45813	0.0846	0.0149	1.32E-08	ZIC1	3q24
3	3:147193692	147193692	A	0.937	0.083	0.0146	1.24E-08	46158	0.0818	0.0146	2.06E-08	ZIC1	3q24
3	3:147198407	147198407	T	0.0602	-0.0842	0.015	1.78E-08	45627.1	-0.0831	0.0150	3.01E-08	ZIC1	3q24
3	3:147203215	147203215	T	0.9373	0.0824	0.0146	1.79E-08	46364.7	0.0812	0.0146	2.58E-08	ZIC1	3q24
3	3:147203820	147203820	A	0.6344	0.0416	0.0073	1.10E-08	46991.8	0.0419	0.0073	9.25E-09	ZIC1	3q24
3	3:147204553	147204553	A	0.0629	-0.0824	0.0146	1.85E-08	46227	-0.0812	0.0146	2.57E-08	ZIC1	3q24
3	3:147207242	147207242	T	0.9373	0.0817	0.0147	2.42E-08	45736.5	0.0805	0.0147	4.21E-08	ZIC1	3q24
3	3:147207565	147207565	T	0.3663	-0.0413	0.0073	1.32E-08	46954.2	-0.0416	0.0073	1.23E-08	ZIC1	3q24
3	3:147207613	147207613	T	0.3662	-0.0415	0.0073	1.18E-08	46959.3	-0.0418	0.0073	1.04E-08	ZIC1	3q24
3	3:147207883	147207883	T	0.9373	0.0817	0.0147	2.44E-08	45736.5	0.0805	0.0147	4.21E-08	ZIC1	3q24
3	3:147208921	147208921	T	0.9393	0.0842	0.0151	2.35E-08	44677.5	0.0829	0.0151	3.89E-08	ZIC1	3q24
3	3:147209179	147209179	T	0.7029	0.0475	0.0077	6.36E-10	46903.7	0.0476	0.0077	6.41E-10	ZIC1	3q24
3	3:147209795:ID	147209795	D	0.0624	-0.1045	0.018	6.29E-09	30627.9	-0.1033	0.0180	9.39E-09	ZIC1	3q24
3	3:147212674	147212674	C	0.0631	-0.0819	0.0146	2.03E-08	46090.6	-0.0808	0.0146	3.09E-08	ZIC1	3q24
3	3:147213259	147213259	A	0.9369	0.0819	0.0146	2.05E-08	46090.6	0.0808	0.0146	3.09E-08	ZIC1	3q24
3	3:147213365	147213365	T	0.9369	0.0819	0.0146	2.05E-08	46090.6	0.0808	0.0146	3.09E-08	ZIC1	3q24
3	3:147215263	147215263	A	0.9369	0.0821	0.0146	2.11E-08	46090.5	0.0807	0.0146	3.20E-08	ZIC1	3q24
3	3:147215392	147215392	A	0.9285	0.0796	0.0142	2.18E-08	43386.9	0.0780	0.0142	3.88E-08	ZIC1	3q24
3	3:147215426	147215426	A	0.0633	-0.0836	0.0146	1.13E-08	45953.4	-0.0822	0.0146	1.77E-08	ZIC1	3q24
3	3:147216849	147216849	A	0.9369	0.0822	0.0147	2.08E-08	45465.5	0.0808	0.0147	3.81E-08	ZIC1	3q24
3	3:147217113	147217113	T	0.9363	0.0816	0.0146	2.40E-08	45685.7	0.0802	0.0146	3.88E-08	ZIC1	3q24
3	3:147217814	147217814	T	0.0631	-0.0823	0.0147	2.00E-08	45465.4	-0.0809	0.0147	3.67E-08	ZIC1	3q24
3	3:147221641	147221641	A	0.9371	0.0818	0.0147	2.76E-08	45600.7	0.0806	0.0147	4.09E-08	ZIC1	3q24
3	3:147222983	147222983	C	0.937	0.0825	0.0147	2.09E-08	45532.6	0.0813	0.0147	3.12E-08	ZIC1	3q24
6	6:126678268	126678268	T	0.4721	-0.0509	0.0077	3.41E-11	39290.6	-0.0322	0.0053	1.43E-09	CENPW	6q22.33
6	6:126815604	126815604	A	0.4589	-0.0435	0.0076	1.04E-08	40491.6	-0.0285	0.0050	9.62E-09	CENPW	6q22.33

Chr	SNP	bp	EA	EAF	b	se	p	n	bC	bC_se	pC	Gene	Loci
8	8:103876183	103876183	A	0.1831	-0.0564	0.0092	7.80E-10	45872.1	-0.0560	0.0092	1.18E-09	AZIN1	8q22.3
8	8:103876325	103876325	A	0.183	-0.0567	0.0092	6.48E-10	45891.2	-0.0563	0.0092	9.67E-10	AZIN1	8q22.3
8	8:103876780	103876780	T	0.1826	-0.0573	0.0092	4.44E-10	45968.5	-0.0569	0.0092	6.47E-10	AZIN1	8q22.3
8	8:103877434	103877434	A	0.3653	-0.0432	0.0074	4.79E-09	45743.8	-0.0428	0.0074	7.39E-09	AZIN1	8q22.3
8	8:103878469	103878469	A	0.7746	0.0566	0.0085	2.46E-11	46030.9	0.0563	0.0085	3.68E-11	AZIN1	8q22.3
8	8:103879532	103879532	A	0.1827	-0.0578	0.0092	3.12E-10	45948.2	-0.0573	0.0092	4.80E-10	AZIN1	8q22.3
8	8:103879686	103879686	C	0.8159	0.0567	0.0091	5.33E-10	46687.7	0.0561	0.0091	6.89E-10	AZIN1	8q22.3
8	8:103879797	103879797	C	0.3678	-0.0427	0.0073	6.29E-09	46871.4	-0.0423	0.0073	7.09E-09	AZIN1	8q22.3
8	8:103880307	103880307	A	0.8169	0.0569	0.0091	4.69E-10	46885.1	0.0564	0.0091	5.68E-10	AZIN1	8q22.3
8	8:103881011	103881011	C	0.6263	0.043	0.0073	4.56E-09	46565.3	0.0426	0.0073	5.36E-09	AZIN1	8q22.3
8	8:103881433	103881433	T	0.1828	-0.0575	0.0092	3.87E-10	45929.1	-0.0570	0.0092	5.89E-10	AZIN1	8q22.3
8	8:103881570	103881570	A	0.6264	0.0428	0.0073	5.46E-09	46570.7	0.0424	0.0073	6.31E-09	AZIN1	8q22.3
8	8:103882228	103882228	C	0.3574	-0.0432	0.0074	5.26E-09	46180.5	-0.0428	0.0074	7.37E-09	AZIN1	8q22.3
8	8:103882658	103882658	T	0.3738	-0.043	0.0073	4.78E-09	46560.3	-0.0426	0.0073	5.20E-09	AZIN1	8q22.3
8	8:103883316	103883316	A	0.357	-0.0441	0.0074	2.54E-09	46202	-0.0437	0.0074	3.62E-09	AZIN1	8q22.3
8	8:103883630	103883630	A	0.1972	-0.0562	0.009	3.33E-10	45286	-0.0558	0.0090	5.57E-10	AZIN1	8q22.3
8	8:103883865	103883865	A	0.6423	0.0436	0.0074	3.61E-09	46162.6	0.0432	0.0074	5.34E-09	AZIN1	8q22.3
8	8:103885884	103885884	A	0.1875	-0.0589	0.0091	9.45E-11	46029.3	-0.0584	0.0091	1.41E-10	AZIN1	8q22.3
8	8:103886116	103886116	T	0.1891	-0.0575	0.009	1.75E-10	46753.5	-0.0570	0.0090	2.42E-10	AZIN1	8q22.3
8	8:103886397	103886397	A	0.8109	0.0575	0.009	1.74E-10	46753.5	0.0570	0.0090	2.42E-10	AZIN1	8q22.3
8	8:103887097	103887097	T	0.189	-0.0575	0.009	1.72E-10	46772.5	-0.0570	0.0090	2.42E-10	AZIN1	8q22.3
8	8:103888931	103888931	C	0.1891	-0.0575	0.009	1.71E-10	46753.5	-0.0570	0.0090	2.42E-10	AZIN1	8q22.3
8	8:103889048	103889048	A	0.7531	0.0557	0.0082	1.17E-11	46441.3	0.0554	0.0082	1.48E-11	AZIN1	8q22.3
8	8:103889803	103889803	T	0.6259	0.0431	0.0073	4.19E-09	46545.1	0.0427	0.0073	4.90E-09	AZIN1	8q22.3
8	8:103890807	103890807	T	0.8165	0.0577	0.0091	2.59E-10	46804.7	0.0573	0.0091	3.17E-10	AZIN1	8q22.3
8	8:103891944	103891944	A	0.8161	0.0582	0.0091	1.83E-10	46725	0.0578	0.0091	2.22E-10	AZIN1	8q22.3
8	8:103892644	103892644	T	0.1837	-0.0574	0.0091	3.15E-10	46765.6	-0.0570	0.0091	3.92E-10	AZIN1	8q22.3
8	8:103892855	103892855	C	0.7725	0.0568	0.0084	1.59E-11	46824.7	0.0564	0.0084	1.85E-11	AZIN1	8q22.3
8	8:103896967	103896967	C	0.1859	-0.0592	0.0091	7.66E-11	46334.1	-0.0587	0.0091	1.12E-10	AZIN1	8q22.3
8	8:103898531	103898531	A	0.627	0.0429	0.0073	5.32E-09	46600.8	0.0425	0.0073	5.77E-09	AZIN1	8q22.3
8	8:103898631	103898631	T	0.8142	0.0589	0.0091	9.94E-11	46353.8	0.0584	0.0091	1.39E-10	AZIN1	8q22.3
8	8:103899282	103899282	A	0.7703	0.0579	0.0084	4.56E-12	46506.6	0.0575	0.0084	7.73E-12	AZIN1	8q22.3
8	8:103900885	103900885	A	0.3727	-0.0428	0.0073	5.85E-09	46616.2	-0.0424	0.0073	6.22E-09	AZIN1	8q22.3
8	8:103901234	103901234	T	0.2291	-0.0581	0.0088	4.49E-11	42452.6	-0.0577	0.0088	5.52E-11	AZIN1	8q22.3
8	8:103901908	103901908	A	0.7202	0.0491	0.0079	4.99E-10	46176.3	0.0486	0.0079	7.47E-10	AZIN1	8q22.3
8	8:103904753	103904753	T	0.6263	0.0429	0.0073	5.32E-09	46565.5	0.0425	0.0073	5.61E-09	AZIN1	8q22.3
8	8:103908028	103908028	T	0.6271	0.0429	0.0073	5.10E-09	46605.9	0.0425	0.0073	5.69E-09	AZIN1	8q22.3
8	8:103908161	103908161	T	0.6271	0.0427	0.0073	6.03E-09	46606.2	0.0423	0.0073	6.70E-09	AZIN1	8q22.3
8	8:103909436:ID	103909436	D	0.184	-0.063	0.0111	1.52E-08	31386.5	-0.0625	0.0111	1.80E-08	AZIN1	8q22.3
8	8:103909612	103909612	A	0.7233	0.0501	0.0079	2.25E-10	46492.2	0.0497	0.0079	3.27E-10	AZIN1	8q22.3
8	8:103909907	103909907	T	0.6279	0.0429	0.0074	5.16E-09	45394.4	0.0425	0.0074	9.19E-09	AZIN1	8q22.3
8	8:103911835	103911835	A	0.2767	-0.0497	0.0079	3.03E-10	46492.8	-0.0493	0.0079	4.53E-10	AZIN1	8q22.3
8	8:103912414	103912414	A	0.6271	0.0428	0.0073	5.78E-09	46606	0.0424	0.0073	6.19E-09	AZIN1	8q22.3
8	8:103912514	103912514	T	0.2755	-0.0506	0.0079	1.81E-10	46616.6	-0.0502	0.0079	2.17E-10	AZIN1	8q22.3
8	8:103914366	103914366	A	0.7668	0.0487	0.0084	6.00E-09	46031.1	0.0482	0.0084	9.70E-09	AZIN1	8q22.3
8	8:103914490	103914490	A	0.6357	0.0432	0.0074	4.58E-09	45797.3	0.0428	0.0074	7.38E-09	AZIN1	8q22.3
8	8:103914604	103914604	A	0.8142	0.0586	0.0091	1.22E-10	46354.2	0.0582	0.0091	1.67E-10	AZIN1	8q22.3
8	8:103914746	103914746	A	0.3742	-0.0432	0.0073	3.94E-09	46539.9	-0.0428	0.0073	4.41E-09	AZIN1	8q22.3
8	8:103916392	103916392	T	0.8831	0.0673	0.0111	1.48E-09	45658.1	0.0674	0.0111	1.28E-09	AZIN1	8q22.3
8	8:103916654	103916654	A	0.627	0.0432	0.0073	4.24E-09	46600.3	0.0428	0.0073	4.46E-09	AZIN1	8q22.3
8	8:103919090	103919090	T	0.7716	0.0588	0.0084	2.67E-12	46691.2	0.0584	0.0084	3.55E-12	AZIN1	8q22.3
8	8:103926036	103926036	A	0.1858	-0.0583	0.0091	1.46E-10	46354.6	-0.0579	0.0091	2.05E-10	AZIN1	8q22.3
8	8:103926197	103926197	T	0.1862	-0.0586	0.0091	1.20E-10	46277.3	-0.0582	0.0091	1.65E-10	AZIN1	8q22.3
8	8:103930583	103930583	A	0.7638	0.054	0.0084	1.15E-10	45616.9	0.0538	0.0084	1.56E-10	AZIN1	8q22.3
8	8:103930817	103930817	C	0.7651	0.0545	0.0084	7.93E-11	45790.8	0.0543	0.0084	1.05E-10	AZIN1	8q22.3
8	8:103934176	103934176	T	0.7989	0.0493	0.0089	3.12E-08	45641.6	0.0494	0.0089	2.89E-08	AZIN1	8q22.3
10	10:132252499	132252499	A	0.3486	-0.0459	0.0083	2.80E-08	37123.6	-0.0469	0.0083	1.55E-08	GLRX3	10q26.3

Chr	SNP	bp	EA	EAF	b	se	p	n	bC	bC_se	pC	Gene	Loci
13	13:51100845	51100845	T	0.0388	0.0856	0.0182	2.69E-06	47026.7	0.1010	0.0177	1.13E-08	TNFSF11	13q14.11
13	13:51163995	51163995	T	0.0663	0.0682	0.0141	1.28E-06	47202.3	0.0804	0.0137	3.85E-09	TNFSF11	13q14.11
17	17:836233	836233	T	0.6486	-0.0434	0.0079	4.21E-08	40830.5	-0.0434	0.0079	4.01E-08	NXN	17p13.3
17	17:837260	837260	A	0.4129	-0.0497	0.0087	1.05E-08	31644.5	-0.0501	0.0087	8.47E-09	NXN	17p13.3
17	17:837381	837381	T	0.5932	-0.0478	0.008	2.25E-09	37598	-0.0479	0.0080	2.23E-09	NXN	17p13.3
17	17:837408	837408	A	0.3653	0.0464	0.0082	1.45E-08	37249.5	0.0463	0.0082	1.60E-08	NXN	17p13.3
17	17:837617	837617	T	0.505	-0.048	0.0083	8.18E-09	33717.7	-0.0482	0.0083	6.14E-09	NXN	17p13.3
17	17:837680	837680	T	0.5778	-0.0512	0.0087	3.84E-09	31443.4	-0.0513	0.0087	3.65E-09	NXN	17p13.3
17	17:66433299	66433299	A	0.2283	-0.0479	0.0085	1.45E-08	45629.2	-0.0463	0.0085	4.66E-08	ARSG	17q24.2
17	17:66445469	66445469	C	0.7693	0.0486	0.0083	5.51E-09	47503.3	0.0471	0.0083	1.19E-08	ARSG	17q24.2
17	17:66446136	66446136	A	0.769	0.0491	0.0083	3.43E-09	47459.4	0.0476	0.0083	8.46E-09	ARSG	17q24.2
17	17:66447073	66447073	T	0.2876	-0.0441	0.0078	1.63E-08	46594.8	-0.0435	0.0078	2.28E-08	PRKAR1A	17q24.2
17	17:66448490	66448490	A	0.232	-0.049	0.0083	3.47E-09	47316.4	-0.0475	0.0083	9.11E-09	PRKAR1A	17q24.2
17	17:66449122	66449122	A	0.7694	0.0488	0.0083	4.70E-09	47517.5	0.0473	0.0083	1.05E-08	PRKAR1A	17q24.2
17	17:66451708	66451708	A	0.2306	-0.0487	0.0083	4.68E-09	47517.6	-0.0472	0.0083	1.15E-08	PRKAR1A	17q24.2
17	17:66453305	66453305	C	0.2289	-0.0503	0.0085	2.67E-09	45541.7	-0.0485	0.0085	9.93E-09	WIPI1	17q24.2
17	17:68060451	68060451	A	0.6552	-0.0489	0.0074	4.74E-11	46938.4	-0.0473	0.0074	1.50E-10	KCNJ6	17q24.3
17	17:68064408	68064408	T	0.3457	0.0486	0.0074	4.42E-11	46881.1	0.0470	0.0074	1.94E-10	KCNJ6	17q24.3
17	17:68075324	68075324	T	0.6551	-0.0478	0.0074	9.70E-11	46933.9	-0.0463	0.0074	3.77E-10	KCNJ6	17q24.3
17	17:68090207	68090207	T	0.3499	0.0488	0.0074	4.51E-11	46617	0.0474	0.0074	1.36E-10	KCNJ6	17q24.3
17	17:68206588	68206588	T	0.5137	0.0387	0.007	3.42E-08	47451.2	0.0395	0.0070	1.55E-08	KCNJ2	17q24.3
17	17:68214336	68214336	A	0.2563	0.0454	0.0081	2.08E-08	46443.7	0.0469	0.0081	6.62E-09	KCNJ2	17q24.3
17	17:68215574	68215574	C	0.6999	-0.0431	0.0077	1.76E-08	46640.4	-0.0445	0.0077	6.65E-09	KCNJ2	17q24.3
17	17:68216128	68216128	A	0.2729	0.0465	0.0078	2.94E-09	48109.3	0.0479	0.0078	7.67E-10	KCNJ2	17q24.3
17	17:68217231	68217231	A	0.4146	0.0493	0.0071	3.79E-12	47456.7	0.0499	0.0071	1.90E-12	KCNJ2	17q24.3
17	17:68217471	68217471	T	0.7412	-0.0462	0.008	8.14E-09	47309.8	-0.0477	0.0080	2.30E-09	KCNJ2	17q24.3
17	17:68217860	68217860	T	0.4183	0.0466	0.0072	7.71E-11	46035.2	0.0476	0.0072	3.34E-11	KCNJ2	17q24.3
17	17:68219001	68219001	T	0.6028	-0.048	0.0072	2.40E-11	46781.8	-0.0487	0.0072	1.29E-11	KCNJ2	17q24.3
17	17:68219033	68219033	A	0.2544	0.0467	0.008	5.65E-09	47843.7	0.0482	0.0080	1.58E-09	KCNJ2	17q24.3
17	17:68220218	68220218	A	0.396	0.0479	0.0072	3.68E-11	46830.6	0.0486	0.0072	1.31E-11	KCNJ2	17q24.3
17	17:68220226	68220226	T	0.6053	-0.0482	0.0072	2.36E-11	46883.4	-0.0491	0.0072	8.56E-12	KCNJ2	17q24.3
17	17:68220870	68220870	T	0.3908	0.0479	0.0072	3.09E-11	47048.9	0.0488	0.0072	1.18E-11	KCNJ2	17q24.3
17	17:68222505	68222505	C	0.2993	0.0435	0.0077	1.39E-08	46711.1	0.0449	0.0077	5.01E-09	KCNJ2	17q24.3
17	17:68231252	68231252	A	0.7462	-0.0452	0.008	1.68E-08	47920.4	-0.0464	0.0080	6.15E-09	KCNJ2	17q24.3
17	17:68235925	68235925	T	0.7431	-0.0453	0.008	1.59E-08	47539.3	-0.0463	0.0080	6.40E-09	KCNJ2	17q24.3
17	17:68237256	68237256	T	0.7456	-0.045	0.008	2.11E-08	47846.1	-0.0461	0.0080	7.68E-09	KCNJ2	17q24.3
17	17:68242546	68242546	A	0.2529	0.0446	0.008	2.32E-08	48034	0.0456	0.0080	1.12E-08	KCNJ2	17q24.3

Supplementary Table 4. Genetic correlation of SK-BMD with different traits. The genetic correlation was calculated based on the summary statistics of the only-Europeans meta-analysis in LD-Hub using its current datasets.

Trait	rg	se	P	PMID
eBMD	0.347	0.0459	4.03E-14	30598549
LS-BMD	0.604	0.0577	1.19E-25	26367794
FN-BMD	0.4761	0.061	5.88E-15	26367794
Fracture	-0.38	0.06	1.17E-10	30158200
Forearm-BMD	0.7651	0.2149	1.76E-06	26367794
Sit. height R	0.2753	0.0576	1.76E-06	25865494
Infant HC	0.2282	0.0703	0.0012	22504419
IBD	-0.103	0.0351	0.0033	26192919
Ulcerative colitis	-0.1089	0.0404	0.007	26192919
Hand grip	0.08	0.03	0.0098	29313844
App-leanmass	0.13	0.05	0.0108	28724990
PGC cross-disorder analysis	0.1177	0.05	0.0185	23453885
Body fat	-0.0873	0.041	0.0334	26833246
Schizophrenia	0.0572	0.0304	0.0595	25056061
Bipolar disorder	0.0871	0.0499	0.0808	21926972
Extreme height	-0.0652	0.039	0.0941	23563607
Difference in height between childhood and adulthood; age 8	-0.1005	0.0655	0.1253	23449627
Crohns disease	-0.0584	0.0398	0.1418	26192919
Eczema	0.0953	0.0668	0.1539	26482879
Rheumatoid Arthritis	-0.0593	0.0427	0.1645	24390342
Waist circumference	-0.0412	0.0304	0.1761	25673412
Primary biliary cirrhosis	0.0841	0.0623	0.1774	26394269
Child birth length	-0.0885	0.0665	0.183	25281659
Child birth weight	-0.0762	0.0573	0.1832	23202124
Overweight	0.0413	0.0311	0.1839	23563607
Difference in height between adolescence and adulthood; age	-0.0941	0.0709	0.1848	23449627
Body mass index	0.0363	0.0281	0.1972	20935630
Childhood obesity	0.0605	0.0494	0.2209	22484627
Obesity class 1	0.0333	0.0292	0.2541	23563607
Obesity class 3	-0.0608	0.0558	0.2765	23563607
Subjective well being	0.0453	0.0449	0.3129	27089181
Waist-to-hip ratio	-0.0316	0.033	0.3369	25673412
Lung cancer (all)	-0.0477	0.0532	0.3698	24880342
Depressive symptoms	0.0371	0.0421	0.3783	27089181
Extreme bmi	0.0363	0.0449	0.4196	23563607
Height_2010	-0.0223	0.0305	0.4646	20881960
Hip circumference	-0.0249	0.0347	0.4735	25673412

Trait	rg	se	P	PMID
Systemic lupus erythematosus	0.0367	0.0537	0.4942	26502338
Mean Caudate	-0.0279	0.0602	0.6431	25607358
Birth weight	-0.014	0.0347	0.687	27680694
Major depressive disorder	0.0224	0.0627	0.7211	22472876
Lung cancer	-0.0152	0.0521	0.7704	27488534
Mean Putamen	0.014	0.0583	0.8103	25607358
Height; Females at age 10 and males at age 12	-0.0131	0.0548	0.8114	23449627
Obesity class 2	-0.0089	0.0377	0.8133	23563607
Anorexia Nervosa	-0.008	0.0387	0.8361	24514567
Celiac disease	0.0071	0.0682	0.9167	20190752
Asthma	0.0064	0.0665	0.9234	17611496
Extreme waist-to-hip ratio	0.0064	0.068	0.9254	23563607
Brain frontal lobe volume	-0.0007	0.0647	0.9909	31396565
Brain temporal lobe volume	0.0621	0.0733	0.3969	31396565
Brain parietal lobe volume	-0.1103	0.0712	0.1212	31396565
Brain occipital lobe volume	0.0826	0.0638	0.196	31396565

Supplementary Table 5. DEPICT Gene prioritization (FDR<5%). Based on the genome-wide significant SNPs in the skull BMD GWAS (N=43,800). Top cis-eQTLs based on Westra et al.[25]. FDR= False Discovery Rate

Locus	Chromosome and position	Gene ID	Gene	P value	Gene closest to lead SNP	Top cis eQTL SNP	FDR
1p13.2	chr1:113009163-113258099	ENSG00000155363	MOV10	2.80E-03	FALSE	rs3748656	<0.05
1q24.3	chr1:171810621-172387606	ENSG00000230630	DNM3OS	2.17E-03	TRUE	-	<0.05
1q24.3	chr1:171810621-172387606	ENSG00000207949	MIR214	3.75E-03	FALSE	-	<0.05
1p36.12	chr1:22351681-22470462	ENSG00000162552	WNT4	2.85E-03	TRUE	rs6688182	<0.05
2q14.2	chr2:119599766-119605254	ENSG00000163064	EN1	1.01E-04	TRUE	-	<=0.01
2p11.2	chr2:85360533-85537511	ENSG00000152284	TCF7L1	3.95E-03	FALSE	-	<0.05
4q22.1	chr4:88742550-88767969	ENSG00000152595	MEPE	8.63E-06	TRUE	-	<=0.01
6q22.33	chr6:127439749-127518910	ENSG00000146374	RSPO3	2.68E-04	TRUE	-	<0.05
6p21.1	chr6:44777054-45518818	ENSG00000124813	RUNX2	2.54E-07	TRUE	rs2772395	<=0.01
6q13	chr6:7107830-7252213	ENSG00000124782	RREB1	4.56E-03	TRUE	rs4960298;rs2714341; rs6907319	<0.05
8q24.12	chr8:119935796-120118821	ENSG00000164761	TNFRSF11B	3.03E-04	TRUE	rs1389541	<0.05
11p14.1	chr11:27352374-27899195	ENSG00000109881	CCDC34	1.24E-03	TRUE	rs3781677	<0.05
11p14-p13	chr11:27352374-27899195	ENSG00000245573	BDNF-AS1	1.83E-03	TRUE	-	<0.05
11p11.2	chr11:46299212-47664175	ENSG00000157613	CREB3L1	3.29E-03	FALSE	-	<0.05
11q13.2	chr11:68080077-68382802	ENSG00000162337	LRP5	3.27E-04	FALSE	rs10750821	<0.05
12q13.12	chr12:49372398-49375459	ENSG00000125084	WNT1	5.43E-03	TRUE	-	<0.05
12q13.12	chr12:49388932-49463808	ENSG00000167548	MLL2	3.76E-03	FALSE	-	<0.05
12q13.12	chr12:49672836-49692465	ENSG00000257960	-	8.52E-04	FALSE	-	<0.05
12q21.33	chr12:90501516-90506066	ENSG00000257194	-	1.19E-03	TRUE	-	<0.05
17q21.31	chr17:41831103-41940994	ENSG00000167941	SOST	4.19E-04	FALSE	rs1731902	<0.05
20p12.2	chr20:10618332-10654608	ENSG00000101384	JAG1	6.50E-04	TRUE	rs8708	<0.05
20q12	chr20:39314488-39317880	ENSG00000204103	MAFB	3.21E-04	TRUE	rs2902941	<0.05

Supplementary Table 6. DEPICT Gene-set enrichment analysis (FDR<5%). These 194 gene-sets were further clustered in 28 'metagene-sets' shown in Figure 3a.

Original gene set ID	Original gene set description	P value	FDR	cluster
MP:0000164	abnormal cartilage development	3.88E-09	<0.01	MP:0000164
MP:0002113	abnormal skeleton development	1.02E-08	<0.01	MP:0002113
MP:0002115	abnormal limb bone morphology	8.09E-08	<0.01	MP:0002115
MP:0003723	abnormal long bone morphology	1.41E-07	<0.01	MP:0000164
MP:0000445	short snout	3.12E-07	<0.01	MP:0000445
GO:0016055	Wnt receptor signaling pathway	4.91E-07	<0.01	GO:0016055
MP:0004686	decreased length of long bones	7.35E-07	<0.01	MP:0002113
MP:0000088	short mandible	8.07E-07	<0.01	MP:0000445
GO:0060070	canonical Wnt receptor signaling pathway	9.14E-07	<0.01	GO:0016055
MP:0000435	shortened head	1.42E-06	<0.01	MP:0000445
GO:0030111	regulation of Wnt receptor signaling pathway	1.55E-06	<0.01	GO:0016055
MP:0002114	abnormal axial skeleton morphology	1.59E-06	<0.01	MP:0002115
MP:0000150	abnormal rib morphology	1.66E-06	<0.01	MP:0002115
MP:0002427	disproportionate dwarf	1.70E-06	<0.01	MP:0002113
MP:0000063	decreased bone mineral density	5.86E-06	<0.01	MP:0000063
MP:0002111	abnormal tail morphology	6.50E-06	<0.01	MP:0002111
MP:0000097	short maxilla	8.02E-06	<0.01	MP:0000445
GO:0060349	bone morphogenesis	1.05E-05	<0.01	MP:0002111
GO:0060350	endochondral bone morphogenesis	1.21E-05	<0.01	MP:0002111
MP:0005006	abnormal osteoblast physiology	1.28E-05	<0.01	MP:0000063
MP:0003935	abnormal craniofacial development	1.52E-05	<0.01	MP:0003935
GO:0001837	epithelial to mesenchymal transition	1.64E-05	<0.01	GO:0001837
MP:0003056	abnormal hyoid bone morphology	1.73E-05	<0.01	MP:0000445
GO:0001503	ossification	1.84E-05	<0.01	GO:0001503
GO:0001501	skeletal system development	2.17E-05	<0.01	MP:0002115
MP:0004989	decreased osteoblast cell number	2.35E-05	<0.01	MP:0000063
ENSG00000143398	PIP5K1A PPI subnetwork	2.45E-05	<0.01	ENSG00000143398
MP:0010029	abnormal basicranium morphology	2.63E-05	<0.01	MP:0002115
GO:0034504	protein localization to nucleus	2.74E-05	<0.01	GO:0034504
MP:0000137	abnormal vertebrae morphology	3.27E-05	<0.01	MP:0002115
MP:0000440	domed cranium	3.38E-05	<0.01	MP:0002113
ENSG00000175387	SMAD2 PPI subnetwork	3.76E-05	<0.01	ENSG00000175387
MP:0000266	abnormal heart morphology	3.86E-05	<0.01	MP:0000266
MP:0000026	abnormal inner ear morphology	4.16E-05	<0.05	MP:0000026
GO:0048598	embryonic morphogenesis	4.24E-05	<0.05	GO:0048598
MP:0000060	delayed bone ossification	4.90E-05	<0.05	GO:0001503
ENSG00000106571	GLI3 PPI subnetwork	5.01E-05	<0.05	ENSG00000175387
GO:0001649	osteoblast differentiation	5.07E-05	<0.05	GO:0001503

Original gene set ID	Original gene set description	P value	FDR	cluster
MP:0000107	abnormal frontal bone morphology	5.35E-05	<0.05	MP:0003935
MP:0008271	abnormal bone ossification	5.59E-05	<0.05	MP:0000063
MP:0005508	abnormal skeleton morphology	5.69E-05	<0.05	MP:0002115
MP:0003408	increased width of hypertrophic chondrocyte zone	5.92E-05	<0.05	MP:0000063
GO:0060348	bone development	6.02E-05	<0.05	MP:0002111
KEGG_WNT_SIGNALING	KEGG_WNT_SIGNALING_PATHWAY	6.04E-05	<0.05	GO:0016055
MP:0000135	decreased compact bone thickness	6.21E-05	<0.05	MP:0000063
MP:0000165	abnormal long bone hypertrophic chondrocyte zone	6.28E-05	<0.05	MP:0002113
GO:0060828	regulation of canonical Wnt receptor signaling pathway	6.68E-05	<0.05	GO:0016055
GO:0006606	protein import into nucleus	6.99E-05	<0.05	GO:0034504
MP:0000455	abnormal maxilla morphology	7.17E-05	<0.05	MP:0000445
MP:0000166	abnormal chondrocyte morphology	7.41E-05	<0.05	MP:0002113
MP:0003419	delayed endochondral bone ossification	7.63E-05	<0.05	MP:0000445
MP:0000550	abnormal forelimb morphology	7.81E-05	<0.05	MP:0000445
ENSG00000179950	PUF60 PPI subnetwork	7.84E-05	<0.05	ENSG00000179950
GO:0002062	chondrocyte differentiation	8.13E-05	<0.05	MP:0002115
MP:0000443	abnormal snout morphology	9.00E-05	<0.05	MP:0003935
ENSG00000167085	PHB PPI subnetwork	9.50E-05	<0.05	ENSG00000179950
MP:0004462	small basisphenoid bone	1.02E-04	<0.05	MP:0000445
GO:0010770	positive regulation of cell morphogenesis involved in differentiation	1.03E-04	<0.05	GO:0001837
GO:0051170	nuclear import	1.03E-04	<0.05	GO:0034504
ENSG00000135547	HEY2 PPI subnetwork	1.18E-04	<0.05	ENSG00000135547
MP:0003662	abnormal long bone epiphyseal plate proliferative zone	1.22E-04	<0.05	MP:0002113
MP:0000428	abnormal craniofacial morphology	1.23E-04	<0.05	GO:0048598
ENSG00000120659	TNFSF11 PPI subnetwork	1.30E-04	<0.05	MP:0000266
MP:0002657	chondrodystrophy	1.32E-04	<0.05	MP:0002113
MP:0004986	abnormal osteoblast morphology	1.35E-04	<0.05	MP:0000063
MP:0006395	abnormal epiphyseal plate morphology	1.36E-04	<0.05	MP:0002113
MP:0005352	small cranium	1.44E-04	<0.05	MP:0000164
MP:0002896	abnormal bone mineralization	1.48E-04	<0.05	MP:0000063
GO:0051098	regulation of binding	1.48E-04	<0.05	GO:0051098
ENSG00000137693	YAP1 PPI subnetwork	1.49E-04	<0.05	MP:0000266
GO:0051216	cartilage development	1.67E-04	<0.05	MP:0002115
ENSG00000037965	HOXC8 PPI subnetwork	1.74E-04	<0.05	MP:0003935
MP:0003055	abnormal long bone epiphyseal plate morphology	1.81E-04	<0.05	MP:0002113
MP:0000049	abnormal middle ear morphology	1.98E-04	<0.05	MP:0000049
MP:0004359	short ulna	2.17E-04	<0.05	MP:0000164
MP:0000914	exencephaly	2.41E-04	<0.05	GO:0048598
MP:0000039	abnormal otic capsule morphology	2.42E-04	<0.05	MP:0000049
MP:0008396	abnormal osteoclast differentiation	2.77E-04	<0.05	MP:0008396
GO:0046822	regulation of nucleocytoplasmic transport	2.78E-04	<0.05	GO:0034504

Original gene set ID	Original gene set description	P value	FDR	cluster
MP:0000130	abnormal trabecular bone morphology	2.79E-04	<0.05	MP:0000063
ENSG00000206300	ENSG00000206300 PPI subnetwork	2.85E-04	<0.05	ENSG00000206300
ENSG00000204435	CSNK2B PPI subnetwork	2.85E-04	<0.05	ENSG00000206300
ENSG00000206406	CSNK2B PPI subnetwork	2.85E-04	<0.05	ENSG00000206300
MP:0004355	short radius	3.26E-04	<0.05	MP:0000164
GO:0048568	embryonic organ development	3.26E-04	<0.05	GO:0048598
MP:0002100	abnormal tooth morphology	3.31E-04	<0.05	MP:0002100
MP:0000438	abnormal cranium morphology	3.42E-04	<0.05	MP:0000445
ENSG00000070770	CSNK2A2 PPI subnetwork	3.54E-04	<0.05	ENSG00000206300
MP:0000547	short limbs	3.56E-04	<0.05	MP:0002113
MP:0002764	short tibia	3.76E-04	<0.05	MP:0000164
MP:0004249	abnormal crista ampullaris morphology	3.77E-04	<0.05	MP:0000026
GO:0019838	growth factor binding	3.80E-04	<0.05	GO:0019838
MP:0003795	abnormal bone structure	3.95E-04	<0.05	MP:0000063
GO:0000785	chromatin	3.99E-04	<0.05	ENSG00000175387
MP:0004351	short humerus	4.03E-04	<0.05	MP:0000164
GO:0042475	odontogenesis of dentin-containing tooth	4.05E-04	<0.05	MP:0002100
MP:0002116	abnormal craniofacial bone morphology	4.08E-04	<0.05	MP:0000445
ENSG00000115844	DLX2 PPI subnetwork	4.09E-04	<0.05	MP:0003935
GO:0090101	negative regulation of transmembrane receptor protein serine/threonine kinase signaling pathway	4.12E-04	<0.05	GO:0001837
MP:0008395	abnormal osteoblast differentiation	4.32E-04	<0.05	MP:0000063
GO:0030178	negative regulation of Wnt receptor signaling pathway	4.33E-04	<0.05	GO:0016055
MP:0000131	abnormal long bone epiphysis morphology	4.35E-04	<0.05	MP:0002113
GO:0001958	endochondral ossification	4.42E-04	<0.05	MP:0002111
ENSG00000167258	CDK12 PPI subnetwork	4.44E-04	<0.05	ENSG00000206300
MP:0000133	abnormal long bone metaphysis morphology	4.64E-04	<0.05	MP:0002113
MP:0002544	brachydactyly	4.74E-04	<0.05	MP:0002115
ENSG00000138685	FGF2 PPI subnetwork	4.75E-04	<0.05	GO:0019838
MP:0009887	abnormal palatal shelf fusion at midline	4.82E-04	<0.05	MP:0000445
GO:0003170	heart valve development	4.87E-04	<0.05	ENSG00000135547
GO:0060562	epithelial tube morphogenesis	4.92E-04	<0.05	GO:0048598
MP:0003054	spina bifida	4.92E-04	<0.05	GO:0048598
GO:0048729	tissue morphogenesis	4.99E-04	<0.05	GO:0048598
MP:0003109	short femur	5.03E-04	<0.05	MP:0000164
ENSG00000005339	CREBBP PPI subnetwork	5.06E-04	<0.05	ENSG00000175387
MP:0003718	maternal effect	5.08E-04	<0.05	MP:0003718
ENSG00000070814	TCOF1 PPI subnetwork	5.11E-04	<0.05	ENSG00000179950
ENSG00000112062	MAPK14 PPI subnetwork	5.42E-04	<0.05	ENSG00000175387
GO:0051090	regulation of sequence-specific DNA binding transcription factor activity	5.46E-04	<0.05	ENSG00000175387
MP:0008525	decreased cranium height	5.52E-04	<0.05	MP:0000445
MP:0001891	hydroencephaly	5.60E-04	<0.05	GO:0048598
MP:0002237	abnormal nasal cavity morphology	5.64E-04	<0.05	MP:0000445

Original gene set ID	Original gene set description	P value	FDR	cluster
GO:0008134	transcription factor binding	5.76E-04	<0.05	ENSG00000175387
MP:0000109	abnormal parietal bone morphology	5.88E-04	<0.05	MP:0003935
GO:0048705	skeletal system morphogenesis	6.01E-04	<0.05	MP:0002115
MP:0005296	abnormal humerus morphology	6.11E-04	<0.05	MP:0000164
GO:0005667	transcription factor complex	6.21E-04	<0.05	ENSG00000175387
MP:0004704	short vertebral column	6.28E-04	<0.05	MP:0000164
GO:0048762	mesenchymal cell differentiation	6.33E-04	<0.05	GO:0001837
GO:0030278	regulation of ossification	6.37E-04	<0.05	GO:0001503
MP:0005157	holoprosencephaly	6.39E-04	<0.05	GO:0048598
GO:0033365	protein localization to organelle	6.41E-04	<0.05	GO:0034504
GO:0014031	mesenchymal cell development	6.43E-04	<0.05	GO:0001837
GO:0048634	regulation of muscle organ development	6.53E-04	<0.05	GO:0001837
GO:0045597	positive regulation of cell differentiation	6.61E-04	<0.05	GO:0048598
MP:0005105	abnormal middle ear ossicle morphology	6.77E-04	<0.05	MP:0000049
MP:0005358	abnormal incisor morphology	6.79E-04	<0.05	MP:0002100
MP:0008023	abnormal styloid process morphology	6.89E-04	<0.05	MP:0000049
GO:0030282	bone mineralization	6.90E-04	<0.05	GO:0001503
REACTOME_SHC:MEDIA	REACTOME_SHC:MEDIATED_CASCADE	6.96E-04	<0.05	MP:0000266
MP:0003560	osteoarthritis	7.07E-04	<0.05	MP:0000063
GO:0051130	positive regulation of cellular component organization	7.12E-04	<0.05	MP:0000266
ENSG00000188313	PLSCR1 PPI subnetwork	7.12E-04	<0.05	ENSG00000188313
MP:0006398	increased long bone epiphyseal plate size	7.12E-04	<0.05	MP:0000063
MP:0002908	delayed wound healing	7.19E-04	<0.05	MP:0000063
ENSG00000111186	WNT5B PPI subnetwork	7.27E-04	<0.05	ENSG00000111186
MP:0008947	increased neuron number	7.27E-04	<0.05	MP:0002100
ENSG00000105329	TGFB1 PPI subnetwork	7.35E-04	<0.05	GO:0019838
GO:0019901	protein kinase binding	7.53E-04	<0.05	ENSG00000175387
GO:0009968	negative regulation of signal transduction	7.54E-04	<0.05	GO:0016055
MP:0001614	abnormal blood vessel morphology	7.71E-04	<0.05	GO:0019838
MP:0000556	abnormal hindlimb morphology	7.75E-04	<0.05	MP:0000445
ENSG00000181789	COPG PPI subnetwork	7.89E-04	<0.05	ENSG00000179950
MP:0000458	abnormal mandible morphology	8.55E-04	<0.05	MP:0000445
MP:0011101	partial prenatal lethality	8.61E-04	<0.05	MP:0000266
MP:0000036	absent semicircular canals	8.62E-04	<0.05	MP:0000026
MP:0000163	abnormal cartilage morphology	8.77E-04	<0.05	MP:0000445
GO:0048562	embryonic organ morphogenesis	8.80E-04	<0.05	GO:0048598
GO:0005520	insulin-like growth factor binding	9.06E-04	<0.05	GO:0019838
MP:0000559	abnormal femur morphology	9.12E-04	<0.05	MP:0000063
ENSG00000147202	DIAPH2 PPI subnetwork	9.46E-04	<0.05	ENSG00000175387
GO:0033157	regulation of intracellular protein transport	9.47E-04	<0.05	GO:0034504
MP:0004136	abnormal tongue muscle morphology	9.52E-04	<0.05	MP:0000049

Original gene set ID	Original gene set description	P value	FDR	cluster
GO:0017038	protein import	9.62E-04	<0.05	GO:0034504
ENSG00000185721	DRG1 PPI subnetwork	9.75E-04	<0.05	ENSG00000179950
GO:0051101	regulation of DNA binding	9.75E-04	<0.05	GO:0051098
MP:0008151	increased diameter of long bones	9.79E-04	<0.05	MP:0002113
ENSG00000115808	STRN PPI subnetwork	9.92E-04	<0.05	ENSG00000179950
GO:0035108	limb morphogenesis	1.00E-03	<0.05	GO:0048598
GO:0035107	appendage morphogenesis	1.00E-03	<0.05	GO:0048598
GO:0043392	negative regulation of DNA binding	1.00E-03	<0.05	GO:0051098
MP:0003148	decreased cochlear coiling	1.01E-03	<0.05	MP:0000026
ENSG00000134245	WNT2B PPI subnetwork	1.03E-03	<0.05	ENSG00000111186
GO:0060021	palate development	1.04E-03	<0.05	MP:0002115
MP:0001541	abnormal osteoclast physiology	1.06E-03	<0.05	MP:0008396
ENSG00000164107	HAND2 PPI subnetwork	1.08E-03	<0.05	ENSG00000135547
MP:0001698	decreased embryo size	1.08E-03	<0.05	ENSG00000175387
GO:0006913	nucleocytoplasmic transport	1.10E-03	<0.05	GO:0034504
KEGG_PATHWAYS_IN_C	KEGG_PATHWAYS_IN_CANCER	1.11E-03	<0.05	MP:0000266
ENSG00000104689	TNFRSF10A PPI subnetwork	1.12E-03	<0.05	ENSG00000104689
ENSG00000115053	NCL PPI subnetwork	1.12E-03	<0.05	ENSG00000179950
ENSG00000170315	UBB PPI subnetwork	1.14E-03	<0.05	ENSG00000179950
MP:0004993	decreased bone resorption	1.18E-03	<0.05	MP:0008396
ENSG00000101266	CSNK2A1 PPI subnetwork	1.19E-03	<0.05	ENSG00000206300
GO:0060485	mesenchyme development	1.20E-03	<0.05	GO:0001837
MP:0001394	circling	1.21E-03	<0.05	MP:0000026
MP:0000079	abnormal basioccipital bone morphology	1.22E-03	<0.05	MP:0002115
ENSG00000139219	COL2A1 PPI subnetwork	1.22E-03	<0.05	MP:0000063
KEGG_TGF_BETA_SIGNA	KEGG_TGF_BETA_SIGNALING_PATHWAY	1.22E-03	<0.05	GO:0001503
GO:0000118	histone deacetylase complex	1.24E-03	<0.05	ENSG00000175387
GO:0051169	nuclear transport	1.25E-03	<0.05	GO:0034504
ENSG00000115596	WNT6 PPI subnetwork	1.26E-03	<0.05	ENSG00000111186
MP:0005587	abnormal Meckel's cartilage morphology	1.27E-03	<0.05	MP:0000445
MP:0006030	abnormal otic vesicle development	1.27E-03	<0.05	MP:0000026
MP:0000454	abnormal jaw morphology	1.27E-03	<0.05	MP:0000445

Supplementary Table 7. eQTL and Colocalization of signals associated with Skull BMD and Osteoclast expression. Showing only results for markers where eQTL analysis FDR<0.05. P=P-value eQTL analysis; PP_H3=Posterior probability that two SNPs in high linkage disequilibrium are underlying the association with osteoclast gene expression and skull BMD variation; PP_H4=Posterior probability that a single SNP is underlying the association with osteoclast gene expression and skull BMD variation. eQTLs with PP4 greater than 50% are written in bold font.

			**eQTL analysis**				**Colocalization analysis**	
Locus	SNP	Gene	Distance	P	Slope	FDR	PP_H3	PP_H4
1p13.2	rs55652172	ST7L	-15841	5.30E-09	0.922	3.33E-07	99.90%	1.62x10-3%
1p36.12	rs7526484	LINC00339	100821	6.29E-06	-0.652	3.68E-04	99.99%	1.95x10-8%
1p36.12	rs7526484	CDC42	72725	0.0015	-0.360	0.0432	65.60%	5.11%
5q22.2	**rs454968**	**REEP5**	**-69781**	**5.68E-04**	**-0.358**	**0.0166**	**24.20%**	**62.70%**
7q31.31	rs2908004	ING3	378966	0.0033	0.365	0.0486	19.70%	5.55%
11p15.5	**rs56257551**	**RIC8A**	**52064**	**1.26E-04**	**-0.749**	**0.0151**	**12.50%**	**52.50%**

Supplementary Table 8. SMR analysis for eQTL and signals associated with Skull BMD. Showing only results for markers where eQTL analysis P<5x10-8. Frequency [Freq] and effect size [beta] are given for A1. P=P.value; nsnps=Number of SNPs. Markers with HEIDI results P>0.05, supporting the hypothesis that a unique SNP is underlying the eQTL and Skull-BMD association are written in bold font

Locus	Gene	topSNP	A1	A2	Freq	beta_GWAS	P_GWAS	beta_eQTL	P_eQTL	beta_SMR	P_SMR	P_HEIDI	nsnp_HEIDI	Tissue/Cell
1p36.12	CDC42	rs2268177	T	A	0.155	-0.095	7.43E-25	0.847	1.09E-298	-0.112	2.66E-23	3.27E-22	20	Blood
1p13.2	**MOV10**	**rs3748656**	**T**	**C**	**0.143**	**0.046**	**1.13E-07**	**0.248**	**2.89E-26**	**0.186**	**2.05E-06**	**0.221**	**15**	Blood
6p21.1	**SUPT3H**	**rs9369523**	**C**	**T**	**0.404**	**0.039**	**1.35E-07**	**-0.237**	**6.18E-34**	**-0.164**	**1.48E-06**	**0.129**	**20**	Blood
7q31.31	CPED1	rs1949803	G	T	0.478	-0.053	4.69E-13	0.271	5.67E-46	-0.193	1.37E-10	7.59E-21	20	Blood
8q22.3	AZIN1	rs1062315	T	A	0.320	-0.041	1.32E-07	-0.320	1.06E-54	0.127	6.25E-07	0.009	20	Blood
8q22.3	**AZIN1-AS1**	**rs1055376**	**A**	**G**	**0.410**	**-0.043**	**4.79E-09**	**-0.207**	**4.90E-25**	**0.209**	**3.72E-07**	**0.209**	**20**	Blood
11p15.5	SCGB1C1	rs11602954	A	G	0.180	0.045	5.18E-07	0.419	6.28E-77	0.107	1.38E-06	0.032	20	Blood
11p15.5	BET1L	rs505404	G	T	0.196	0.045	5.74E-08	0.193	3.52E-17	0.231	4.71E-06	0.001	19	Blood
11q13.3	TESMIN	rs2513272	C	T	0.298	-0.042	9.60E-08	-0.225	1.66E-26	0.186	1.69E-06	3.88E-05	20	Blood
11q13.3	CPT1A	rs3136547	C	G	0.283	-0.042	6.18E-08	-0.257	4.19E-34	0.165	7.27E-07	2.66E-06	20	Blood
12q23.3	**TMEM263**	**rs2888893**	**C**	**T**	**0.460**	**-0.045**	**1.49E-10**	**0.151**	**4.98E-15**	**-0.301**	**7.34E-07**	**0.263**	**20**	Blood
14q24.3	**EIF2B2**	**rs175016**	**T**	**C**	**0.441**	**-0.036**	**6.02E-07**	**-0.622**	**1.78E-278**	**0.058**	**7.89E-07**	**0.877**	**20**	Blood
14q24.3	**MLH3**	**rs13712**	**T**	**C**	**0.441**	**-0.036**	**5.53E-07**	**-0.285**	**8.73E-51**	**0.126**	**2.23E-06**	**0.860**	**20**	Blood
16p13.3	DECR2	rs1698231	C	T	0.503	-0.034	3.96E-06	0.405	1.57E-103	-0.084	6.99E-06	0.008	20	Blood
20q12	**MAFB**	**rs2902941**	**G**	**A**	**0.360**	**0.039**	**2.40E-07**	**0.185**	**8.30E-20**	**0.213**	**6.89E-06**	**0.141**	**20**	Blood
1p36.12	LINC00339	rs2501289	C	G	0.332	0.052	1.05E-11	-1.072	3.35E-22	-0.049	2.56E-08	1.47E-10	20	Osteoclast
16p13.3	TMEM8A	rs8064205	G	A	0.460	0.040	2.63E-08	-0.731	1.34E-10	-0.055	2.72E-05	1.63E-08	20	Osteoclast

Supplementary Table 9. Capture C interaction data from hMSC-derived osteoblasts. Cells were obtained from 3 independent donors. Significant calls from CHiCAGO with CHiCAGO score >=5 are reported for the Skull BMD GWAS loci , using open proxies with r^2>=0.4.

LOCUS	SENTINEL_SNP	PROXY_SNP_(R2)	SNP_POSITION	SNP_FRAGMENT	IMPLICATED_GENE	BAIT	DISTANCE_SNP_BAIT	NUM_READS	CHiCAGO_SCORE	RESOLUTION
CCDC91	rs10843202	rs35098487 (0.56)	chr12:28343976	chr12:28342813-28345036	TCONS_00021090	chr12:287	380543	6	5.54	4frag
CFAP221	rs11679232	rs62159869 (0.89)	chr2:119613117	chr2:119612492-119613570	TCONS_l2_00014855	chr2:1189	647079	7	6.27	4frag
CFAP221	rs11679232	rs62159869 (0.89)	chr2:119613117	chr2:119612492-119613570	TCONS_00003829	chr2:1189	628957	5	7.12	4frag
DEPTOR	rs7842942	rs13277230 (0.70)	chr8:119922993	chr8:119921858-119925117	TNFRSF11B/COLEC10	chr8:1200	155622	18	10.66	4frag
DNM3	rs495590	rs562932 (0.70)	chr1:172150960	chr1:172149458-172152278	C1orf105	chr1:1723	237005	6	5.18	4frag
FAM13A	rs11934731	rs13127257 (0.41)	chr4:88765516	chr4:88764918-88766746	SPARCL1	chr4:8844	312853	7	7.83	4frag
FAM13A	rs11934731	rs13127257 (0.41)	chr4:88765516	chr4:88764918-88766746	DMP1	chr4:8857	193743	12	10.93	4frag
KMT5B	rs11228240	rs901823 (0.56)	chr11:68205578	chr11:68203432-68207950	TCONS_00019360	chr11:680	155210	5	5.66	4frag
LRIG2	rs55652172	rs12122980 (0.94)	chr1:113157181	chr1:113156543-113157221	CTTNBP2NL	chr1:1129	217085	4	6.19	1frag
LRIG2	rs55652172	rs12122980 (0.94)	chr1:113157181	chr1:113156413-113157724	MIR4256	chr1:1130	152439	6	7.03	4frag
LRIG2	rs55652172	rs2279499 (0.79)	chr1:113247563	chr1:113246392-113247910	CTTNBP2NL	chr1:1129	307467	4	6.62	1frag
LRIG2	rs55652172	rs2279499 (0.79)	chr1:113247563	chr1:113246392-113247910	WNT2B	chr1:1130	237706	3	5.26	1frag
MAT2A	rs11675489	rs6547609 (0.40)	chr2:85512675	chr2:85512519-85512877	TCF7L1	chr2:8535	151755	5	9.23	1frag
POSTN	rs493248	rs17217404 (0.99)	chr13:37466215	chr13:37465828-37466760	SMAD9	chr13:374	27217	6	5.13	1frag
RIMS2	rs11993347	rs2916559 (0.43)	chr8:103827084	chr8:103825579-103827745	ODF1	chr8:1035	262404	6	5.86	4frag
SMG6	rs4790881	rs35401268 (0.87)	chr17:2026417	chr17:2026042-2027041	SMG6	chr17:199	34079	6	6.12	1frag
SMG6	rs4790881	rs35401268 (0.87)	chr17:2026417	chr17:2023679-2027041	LOC101927839	chr17:213	107825	13	18.09	4frag
SMG6	rs4790881	rs148183711 (0.79)	chr17:2118506	chr17:2117462-2118920	LOC101927839	chr17:213	15736	16	6.84	4frag
SUPT3H	rs17423748	rs3799973 (0.42)	chr6:44823727	chr6:44822963-44824144	RUNX2	chr6:4529	471540	5	6.35	1frag
ZBTB40	rs7526484	rs34446016 (0.42)	chr1:22458637	chr1:22458035-22458666	WNT4	chr1:2246	9977	10	5.46	1frag
ZBTB40	rs7526484	rs742358 (0.45)	chr1:22459170	chr1:22458667-22459843	WNT4	chr1:2246	9444	10	5.23	1frag
ZNF366	rs11948073	rs10805874 (0.49), rs12517173 (0.87)	chr5:72607981, chr5:72607398	chr5:72606661-72610093	TCONS_00009420, TCONS_00009421	chr5:7258	20069; 19486	23	6.94	4frag
ZNF366	rs11948073	rs10805874 (0.49), rs12517173 (0.87)	chr5:72607981, chr5:72607398	chr5:72607278-72609451	TCONS_00009423 (FOXD1-AS)	chr5:7274	133923; 134506	6	8.92	1frag
ZNF366	rs11948073	rs368996568 (1.00)	chr5:72618031	chr5:72617174-72618214	TCONS_00009423 (FOXD1-AS)	chr5:7274	123873	4	5.23	1frag

Supplementary Table 10. Site-specific BMD measurements of fish of similar standard size. Values of whole body and skull BMD for *zic1* (n=6), *atp6v1c1 (a+b)* (n=3) crispants and controls (n=6) and *prkar1a (a+b)* crispants (n=3) and controls (n=3). Values are reported as g/cm^3. Data source for Figure 4e-h.

control	zic1	atp6v1c1		Whole body BMD	
0.44907	0.38539	0.3805			
0.45064	0.4255	0.33498			
0.42857	0.35351	0.36065			
0.40161	0.32893				
0.41412	0.36018				
0.40098	0.33301				

control	prkar1a			Whole body BMD	
0.37673	0.45409				
0.40349	0.43239				
0.40252	0.42593				

control	zic1	atp6v1c1		Skull BMD	
0.4593	0.39927	0.3953			
0.45927	0.42016	0.39008			
0.44611	0.38208	0.38937			
0.41279	0.38376				
0.42119	0.38795				
0.41218	0.36538				

control	prkar1a			Skull BMD	
0.4033	0.45777				
0.41266	0.43499				
0.42349	0.44271				

Supplementary Table 11. CRISPR/CAs9 targeting sequences

Gene/ gRNA name	Target Sequence 5'-3' (without PAM)	PAM	Exon Targeted	Position danRer11/zebrafish
zic1_cr1	GGGAAGAGTGCGGCCGGGAA	GGG	1	chr24:4972675-4972697
zic1_cr2	TGGGAAGAGTGCGGCCGGGA	AGG	1	chr24:4972676-4972698
zic1_cr3	GATTTTCAGATTTTCCGATC	GGG	1	chr24:4972552-4972574
atp6v1C1a_cr1	GAGTCTGTTGACCAGGAGTT	TGG	7	chr16:52757882-52757904
atp6v1C1a_cr2	AGACGACTTTGTGCTGGATT	CGG	7	chr16:52757921-52757943
atp6v1C1a_cr3	AGTGGTACCAAAGTAAGTGA	CGG	7	chr16:52757966-52757988
atp6v1C1b_cr4	TACATTACCCGGTTCCAGT	GGG	5	chr19:32953948
atp6v1C1b_cr5	GCTGATATAGTCAAGAAGG	AGG	5	chr19:32954332
atp6v1C1b_cr6	ATACATTACCCGGTTCCAG	TGG	5	chr19:32953947
prkar1a_a_cr1	AGTCTCTGAGGTAAGCCAT	GGG	3	chr3:36268843
prkar1a_a_cr2	AGTGCAGAGGTCTACACTG	AGG	3	chr3:36268573
prkar1a_a_cr3	CCGTCGGCCTTTAACCACA	GGG	3	chr3:36268610
prkar1a_b_ cr4	GCGACCAGCATTGGCGAGGG	CGG	7	chr12:2881146-2881168
prkar1a_b_ cr5	GGTCTTGGCTCTGACGGTGG	CGG	7	chr12:2881210-2881232
prkar1a_b_ cr6	GGAGAGCTGGCGTTAATCTA	CGG	7	chr12:2881176-2881198

Supplementary Table 12.Primers used for fragment length analysis

gene and gRNAs	M13+F	R	Amplicon sequence	amplicon
zic1_exon1	TGTAAAACGACGGCCAGTTATGCACGAGCTTGTGACCC	ACCTCTTCATTAAAAACTAGGAATGAA	>chr24:4972471-4972771 301bp TATGCACGAGCTTGTGACCC<br>ACCTCTTCATTAAAAACTAGGAATGAA<br>TATGCACGAGCTTGTGACCCatctgactgtagagcacgttgggggaccag<br>agcagtcgaatcacatttgcttttgggaagagtgcggccgggaagggaaa<br>ccgtttaaagcaaaatataaacttgtgaatcatatcagagttcacacggg<br>agagaaaccgtttccgtgtccattccccggctgtggaaaagtatttgccc<br>gatcggaaaatctgaaaatccacaaaagaacacacacaggtaagaaaatc<br>gttattttcatctctaaatacacgTTCATTCCTAGTTTTTAATGAAGAGGT	301
atp6v1C1_a	TGTAAAACGACGGCCAGTACACACTGTGAGCATGTGCA	AATGTGAGAGCTGGTGGTCC	>chr16:52757779-52758081 303bp ACACACTGTGAGCATGTGCA<br>AATGTGAGAGCTGGTGGTCC<br>ACACACTGTGAGCATGTGCAtaagagcaattcaggagttgtgtgactgtc<br>ttcaaatattttcagacttgttcatttttttgtgttgaggaatccgtcac<br>ttactttggtaccactacaagtaaagtaatgaggtactccgaatccagca<br>caaagtcgtctttcttgacaatatcagccaaactcctggtcaacagactc<br>cctctgatgacaacacaaaaccacacgttagttgattggaaaaggctatg<br>tccatacaagtgtctctcaagtacgtttcgggaGGACCACCAGCTCTCACATT	303
atp6v1C1_b	TGTAAAACGACGGCCAGTAGAGTCCAACACAAAATCCTCCT	CCGGTTCCAGTGGGATATGG	>chr19:32953956-32954370 415bp AGAGTCCAACACAAAATCCTCCT<br>CCGGTTCCAGTGGGATATGGAGAGTCCAACACAAAATCCTCCTtcttgactatatcagc<br>caaactcctagtcaaaagacttcctctggtgggcaagacatgttattactcgatatcattt<br>aaaacatcattaaatgttgcaagtaaactgaaaatataaaaaagtcacac<br>agacttacacattctttctctccaggctctgcaggctgccctttaggcta<br>ttataggctgaagctctggctttaagatcattgtcaatctgagtcacttg<br>ctaataagagaagcaaagcatgaaatgtaatgaatgaaagatcaatctcc<br>acgtgtattataaacactgataacgattttaacatgcctaaccttagaca<br>tgatctctgagatgctcttaagggactgttttatgggatacttggCCATATCCCACTGGAACCGG	
prkar1a_a	TGTAAAACGACGGCCAGTGCGTCCTCCTCAGTGTAGAC	GAAGCACAACATCCAGCAGC	>chr3:36268545+36268910 366bp<br>GCGTCCTCCTCAGTGTAGACGAAGCACAACATCCAGCAGCGCGTCCTCCTCAGTG<br>TAGACctctgcactgatggcaccacggcgccgtcg<br>gcctttaaccacagggttcataggaggagagacctcatcttcccgagaat<br>ctgagcgagagctggacttctgctggctcaccatctgtttagcctcctcc<br>tgattgacataaacaccattcgtggaagtgagacttactttttatgagta<br>tagaaaatgtgtaagtgttcaagcccatgcggttttcctctgaagaacac<br>aactgaccaactcaagcttctcgaagtagtctctgaggtaagccatgggt<br>ctttcaggcctggcggtgcacagctgcacaatgcagtccttcagcaGCTG<br>CTGGATGTTGTGCTTC	
prkar1a_b	TGTAAAACGACGGCCAGTCCAAAGTCCAGTTTCACTCAGG	ACCTCAAATGTTCACGCGAC	>chr12:2881060+2881402 343bp CCAAAGTCCAGTTTCACTCAGG<br>ACCTCAAATGTTCACGCGACCCAAAGTCCAGTTTCA<br>CTCAGGattaagtcagattacaccagtgagagat<br>gatgtttgtgcacaggtgtatgtgaacaatgagtgggcgaccagcattgg<br>cgagggcggcagttttggagagctggcgttaatctacggcaccccgagag<br>ccgccaccgtcagagccaagaccaacgtgaagctgtggggcatcgacaga<br>gacagctataggagaatactgatggtacaacatcacctcaacacacacac<br>acgcacactacactttaatgctgtgttcacaccagacgcggaacgcgcgg<br>ataaatcgcgctattcgtaaataGTCGCGTGAACATTTGAGGT	343

Supplementary figures

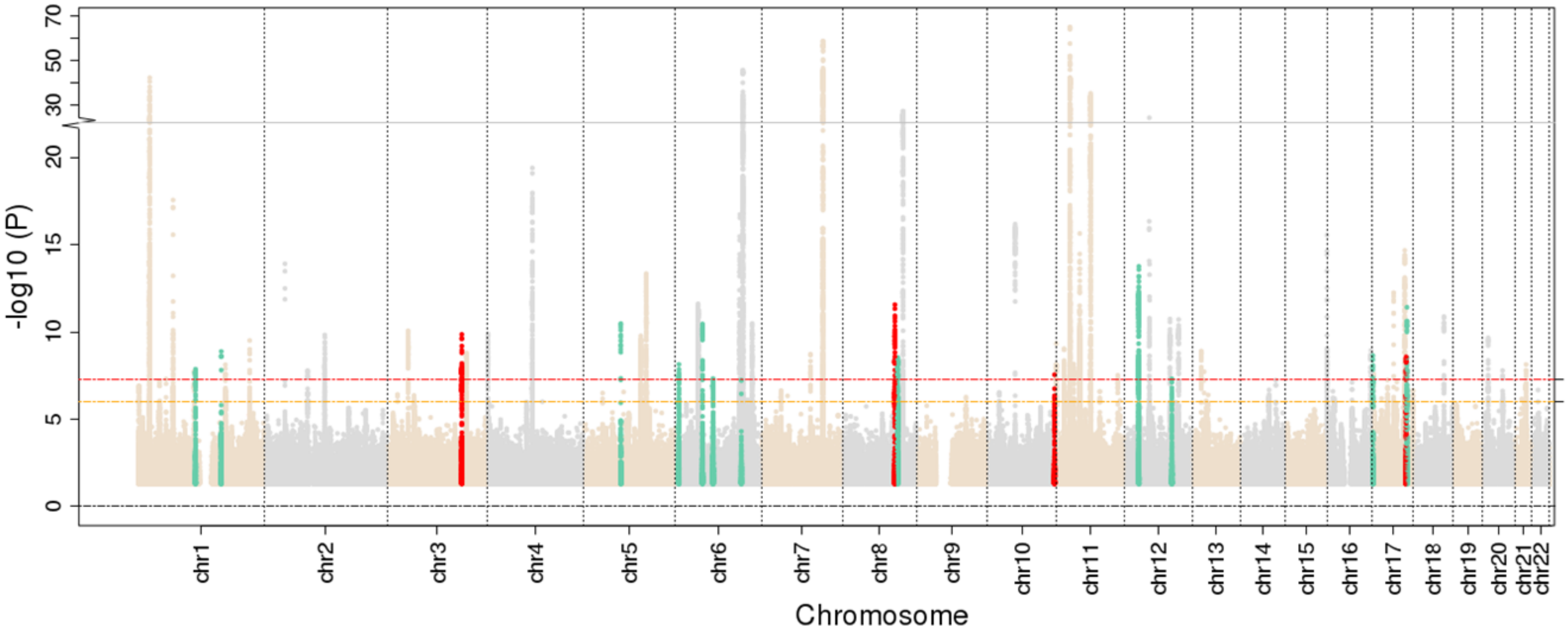

Supplementary Figure 1. Manhattan Plot SK-BMD Meta-analysis. Association signals independent from those previously reported for GWAS for DXA/pQCT efforts are presented in aquamarine. BMD association signals in loci not previously reported (so also independent from UKBB ultrasound efforts) are represented by red dots

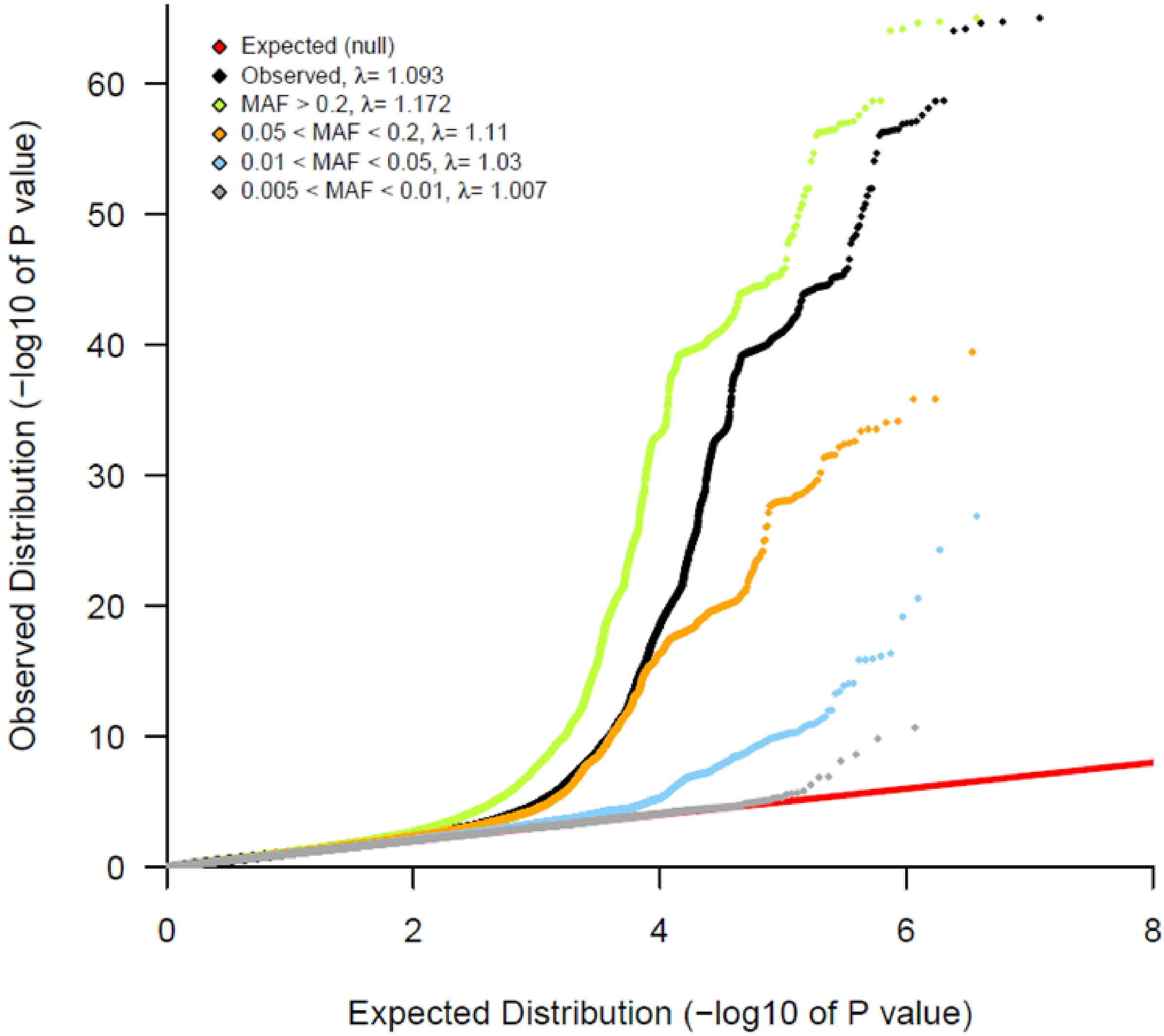

Supplementary Figure 2. QQ-Plot for the genome-wide association study of SK-BMD. This plot show the inflation of the test statistics per bin of minor allele frequency

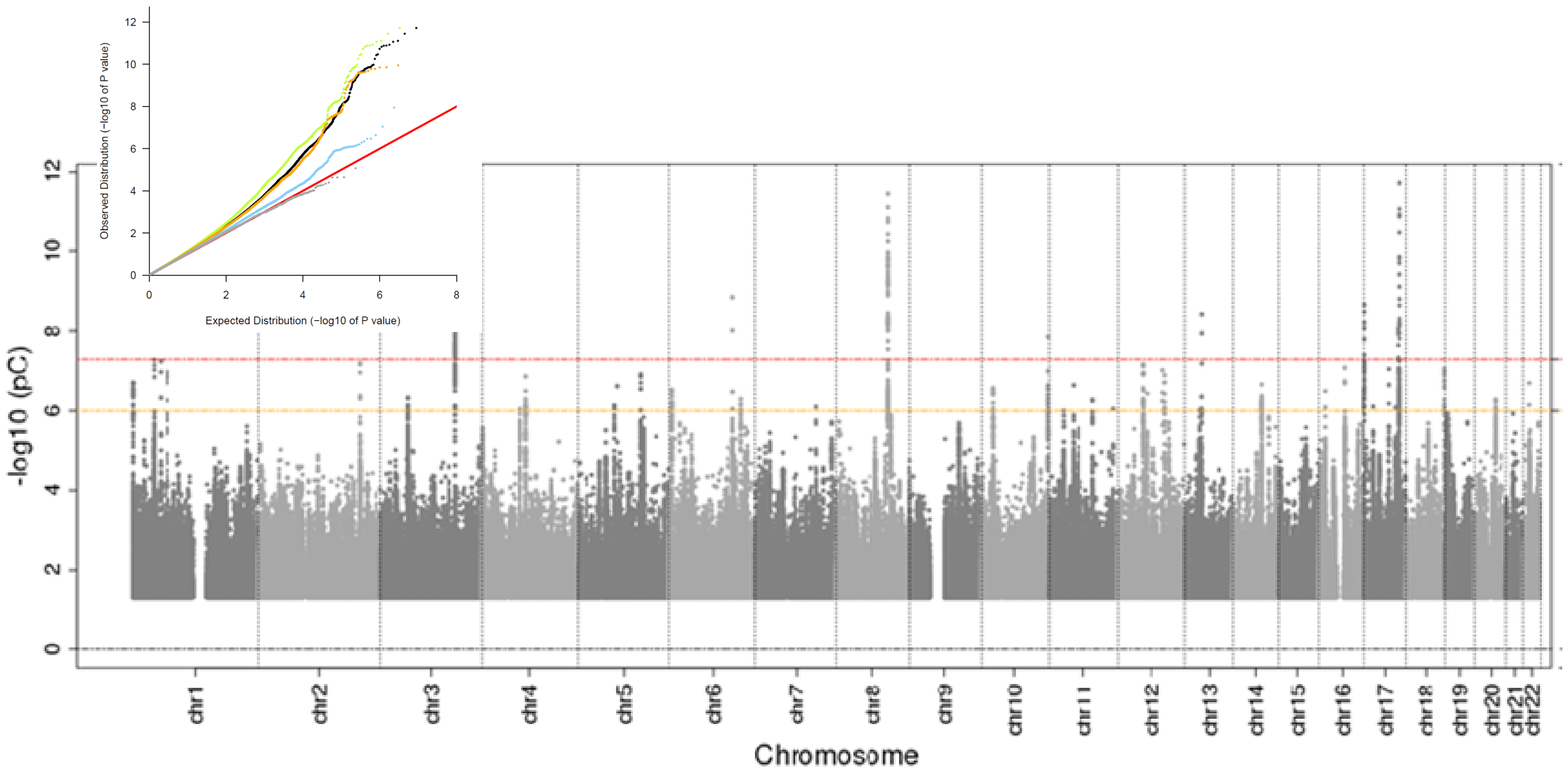

Supplementary Figure 3. Manhattan and QQ-plots of association statistics (-log10(P values)) for SK-BMD meta-analysis after conditional analysis on known independent variants. Each dot represents a SNP and the x-axis indicates its chromosomal position (Build 37 NCBI). Dashed horizontal red and yellow lines mark the GWS threshold ($P<5x10^{-8}$) and suggestive threshold ($P<1x10^{-6}$), respectively. Top: QQ-plot showing the inflation of the test statistics per bin of minor allele frequency

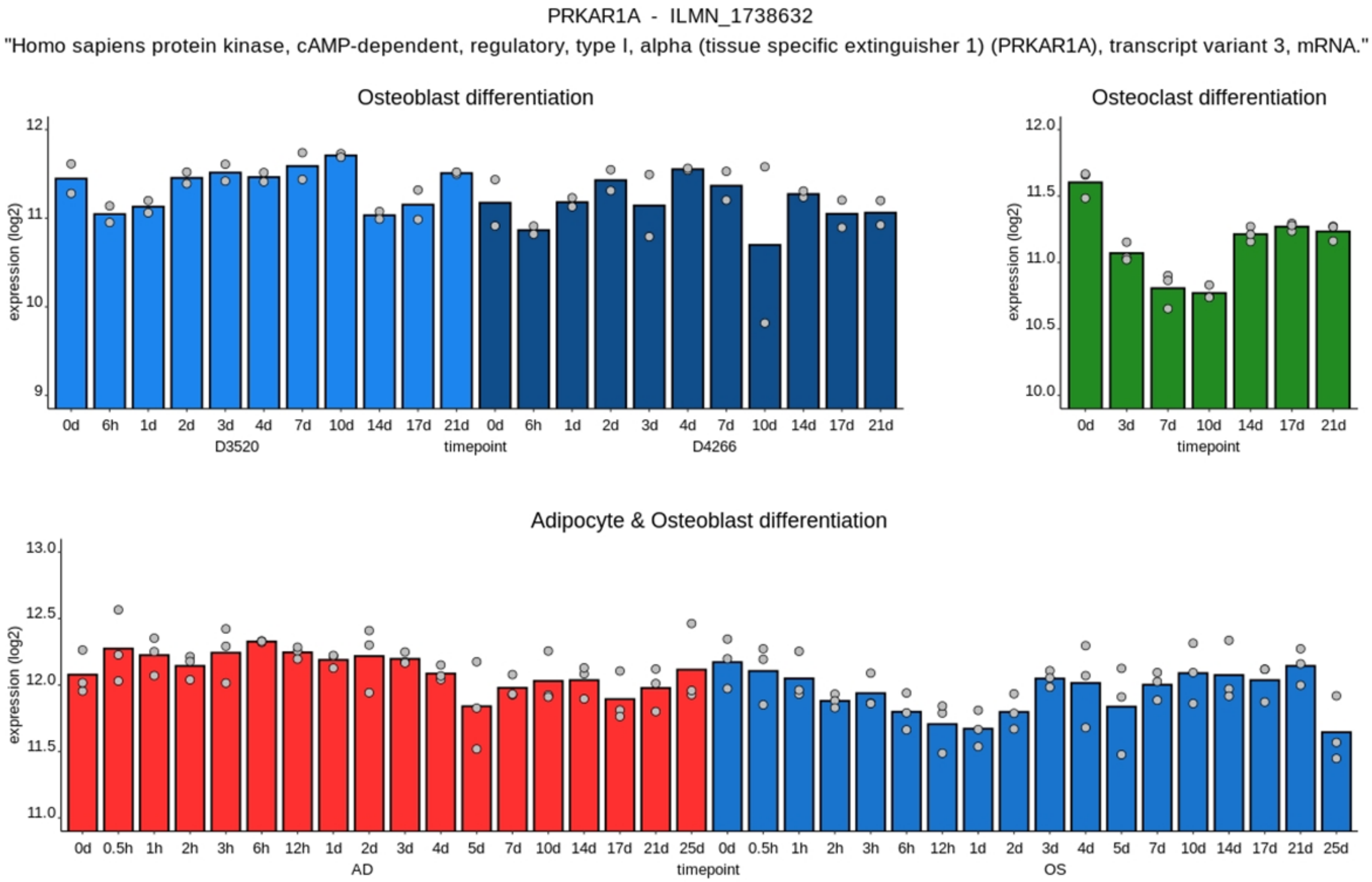

Supplementary Figure 4. Longitudinal assessment of *PRKAR1A* expression in human cells. Upper left) Osteoblast differentiation Bone marrow derived human MSCs from 2 different donors (two replicates per time point) differentiated into Osteoblasts. **Upper right**) **Osteoclast differentiation** human PBMCs differentiated into Osteoclast from 2 different donors. **Bottom) Adipocyte and Osteoblast differentiation** of Bone marrow derive human MSCs from a single donor (three replicates per time point) .

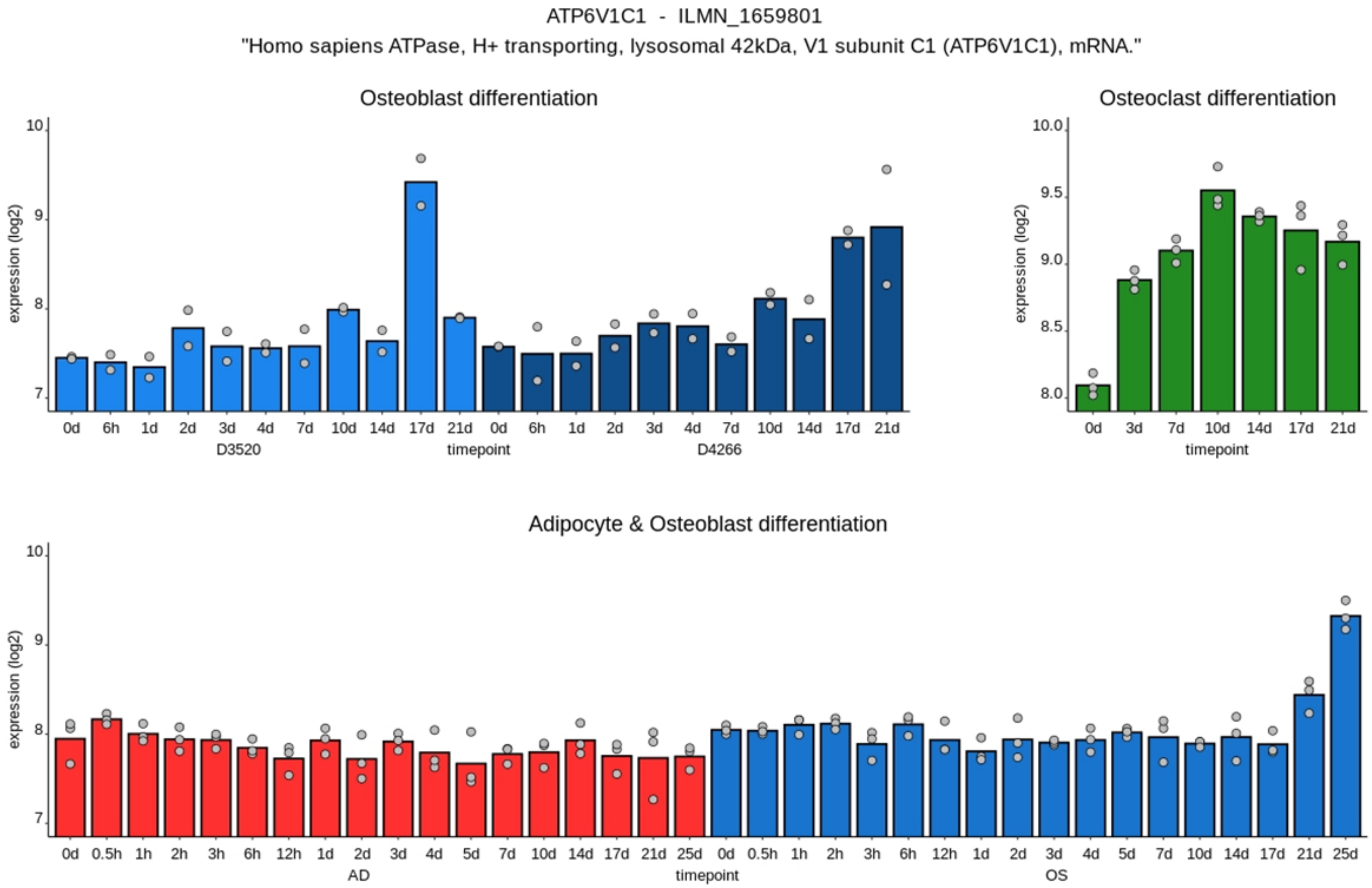

Supplementary Figure 5. Longitudinal assessment of *ATP6V1C1* expression in human cells. Upper left) Osteoblast differentiation Bone marrow derived human MSCs from 2 different donors (two replicates per time point) differentiated into Osteoblasts. **Upper right) Osteoclast differentiation** human PBMCs differentiated into Osteoclast from 2 different donors. **Bottom) Adipocyte and Osteoblast differentiation** of Bone marrow derive human MSCs from a single donor (three replicates per time point).

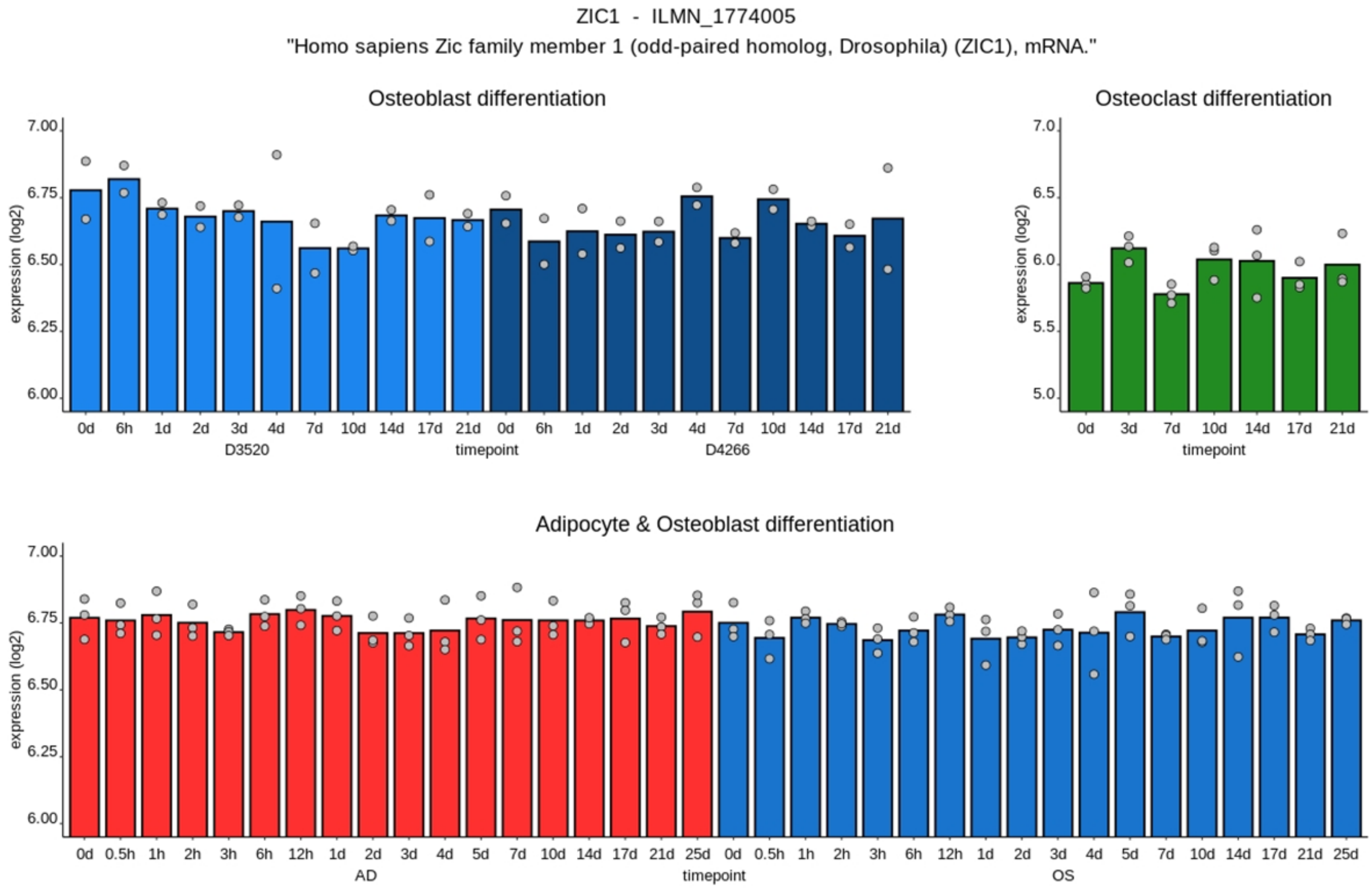

Supplementary Figure 6. Longitudinal assessment of *ZIC1* expression in human cells. Upper left) Osteoblast differentiation Bone marrow derived human MSCs from 2 different donors (two replicates per time point) differentiated into Osteoblasts. **Upper right**) **Osteoclast differentiation** human PBMCs differentiated into Osteoclast from 2 different donors. **Bottom) Adipocyte and Osteoblast differentiation** of Bone marrow derive human MSCs from a single donor (three replicates per time point) .

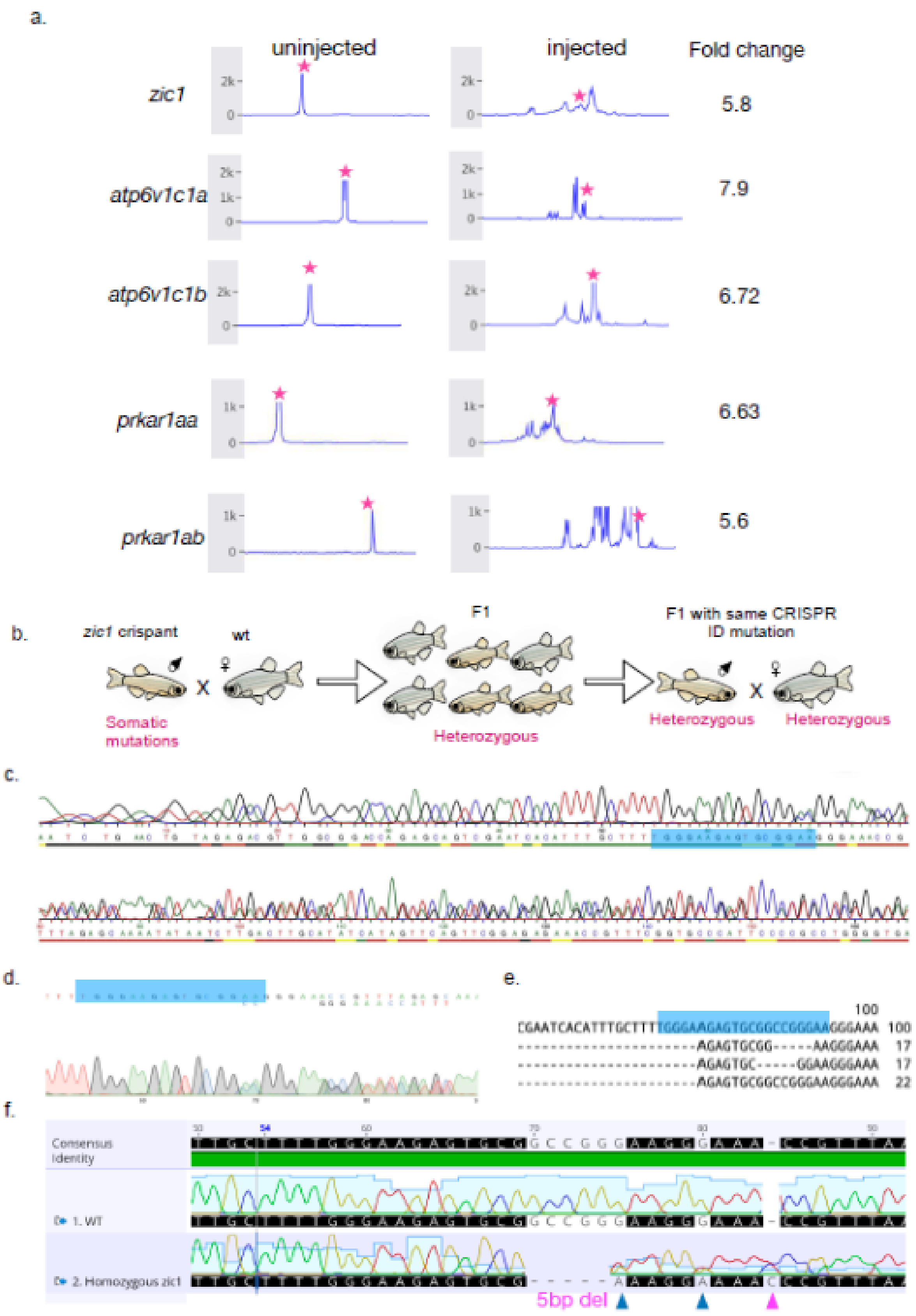
a.
uninjected
injected
Fold change
zic1
5.8
atp6v1c1a
7.9
atp6v1c1b
6.72
prkar1aa
6.63
prkar1ab
5.6
b.
zic1 crispant
wt
F1
F1 with same CRISPR ID mutation
Somatic mutations
Heterozygous
Heterozygous
Heterozygous
c.
d.
e.
CGAATCACATTTGCTTTTGGGAAGAGTGCGGCCGGGAAGGGAAA 100
AGAGTGCGG-----AAGGGAAA 17
AGAGTGC-----GGAAGGGAAA 17
AGAGTGCGGCCGGGAAGGGAAA 22
f.
Consensus
Identity
1. WT
2. Homozygous zic1
5bp del

Supplementary Figure 7. Analysis of the mutagenesis efficiency in crispants and the generation of zic1 homozygous mutants. a) Evaluation of CRISPR efficiency. Amplification peaks for uninjected and injected pools (n=8) are shown for each gene. Red stars show the wt peak. Fold change was calculated by dividing the wt peak between uninjected and injected pools. **b) Generation of zic1 homozygous mutants.** zic1 crispants were crossed to wt to generate F1 (carrying different types of heterozygous mutations). F1 was genotyped using Sanger sequencing and analysed using CRISPR-ID. A pair of F1 carrying the same mutation were crossed to generate F2. **c) Sanger sequencing of F1 fish.** Blue square highlights a region targeted by two gRNAS. d and e) CRISPR-ID analysis showing the alignment between a reference control and F1. A pair of fish with the same mutation were crossed to generate zic1 homozygous mutants (F2). **e) CRISPR-ID analysis of one F1 sequence** (fish with asymmetrical caudal fin), predicting 5bp deletions. **f) Sequence alignment (Geneious) of a wt and a zic1 homozygous mutant.** Note the confirmation of a deletion of 5bp, two mismatches (blue arrowheads) and an insertion of a base pair (magenta arrowhead). These mutations lead to truncation of the zic1 protein, reducing protein length from 442aa to 283aa**.**

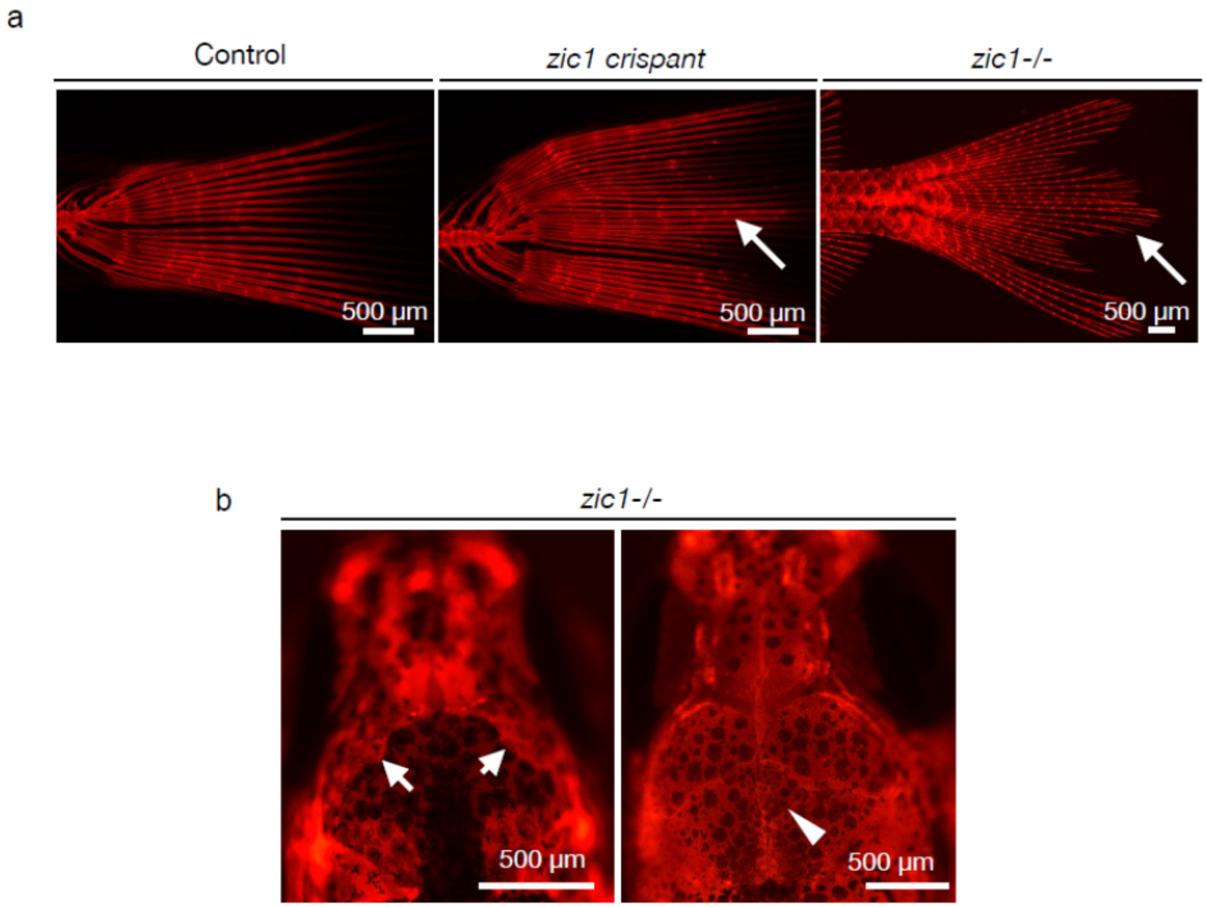

Supplementary Figure 8. Zebrafish zic1 homozygous mutants show similar phenotypes as crispants. a) Asymmetrical caudal fin phenotype was found among crispants, a persistent trait in homozygous mutants. Asymmetrical caudal fin phenotype was detected in 13/55 (24%) zebrafish. **b) *zic1* homozygous mutants phenotype.** *Zic1* homozygous mutants showed a delay in skull formation, with uneven bone growth (arrows), that can culminate in ectopic suture (arrowhead), like in crispants. While skull phenotype was not detected in any zebrafish with normal fins, all zebrafish with abnormal caudal fins showed irregular cranial bone growth. Scale bars are indicated in each figure.

Supplementary references

1. Boyd, A. *et al.* Cohort Profile: the 'children of the 90s'--the index offspring of the Avon Longitudinal Study of Parents and Children. *Int J Epidemiol* **42**, 111-27 (2013).
2. Fraser, A. *et al.* Cohort Profile: the Avon Longitudinal Study of Parents and Children: ALSPAC mothers cohort. *Int J Epidemiol* **42**, 97-110 (2013).
3. Northstone, K. *et al.* The Avon Longitudinal Study of Parents and Children (ALSPAC): an update on the enrolled sample of index children in 2019 [version 1; peer review: 2 approved]. *Wellcome Open Research* **4**(2019).
4. Kemp, J.P. *et al.* Phenotypic dissection of bone mineral density reveals skeletal site specificity and facilitates the identification of novel loci in the genetic regulation of bone mass attainment. *PLoS Genet* **10**, e1004423 (2014).
5. Kalkwarf, H.J. *et al.* The bone mineral density in childhood study: bone mineral content and density according to age, sex, and race. *J Clin Endocrinol Metab* **92**, 2087-99 (2007).
6. Zemel, B.S. *et al.* Revised reference curves for bone mineral content and areal bone mineral density according to age and sex for black and non-black children: results of the bone mineral density in childhood study. *J Clin Endocrinol Metab* **96**, 3160-9 (2011).
7. van Wijngaarden, J.P. *et al.* Rationale and design of the B-PROOF study, a randomized controlled trial on the effect of supplemental intake of vitamin B12 and folic acid on fracture incidence. *BMC Geriatr* **11**, 80 (2011).
8. Bisgaard, H. The Copenhagen Prospective Study on Asthma in Childhood (COPSAC): design, rationale, and baseline data from a longitudinal birth cohort study. *Ann Allergy Asthma Immunol* **93**, 381-9 (2004).
9. Ahluwalia, T.S. *et al.* FUT2-ABO epistasis increases the risk of early childhood asthma and Streptococcus pneumoniae respiratory illnesses. *Nat Commun* **11**, 6398 (2020).
10. Bisgaard, H. *et al.* Deep phenotyping of the unselected COPSAC2010 birth cohort study. *Clin Exp Allergy* **43**, 1384-94 (2013).
11. Styrkarsdottir, U. *et al.* Nonsense mutation in the LGR4 gene is associated with several human diseases and other traits. *Nature* **497**, 517-20 (2013).
12. Kooijman, M.N. *et al.* The Generation R Study: design and cohort update 2017. *Eur J Epidemiol* **31**, 1243-1264 (2016).
13. Medina-Gomez, C. *et al.* Challenges in conducting genome-wide association studies in highly admixed multi-ethnic populations: the Generation R Study. *Eur J Epidemiol* **30**, 317-30 (2015).
14. Lorentzon, M., Mellstrom, D. & Ohlsson, C. Age of attainment of peak bone mass is site specific in Swedish men--The GOOD study. *J Bone Miner Res* **20**, 1223-7 (2005).
15. Orwoll, E. *et al.* Design and baseline characteristics of the osteoporotic fractures in men (MrOS) study--a large observational study of the determinants of fracture in older men. *Contemp Clin Trials* **26**, 569-85 (2005).
16. Blank, J.B. *et al.* Overview of recruitment for the osteoporotic fractures in men study (MrOS). *Contemp Clin Trials* **26**, 557-68 (2005).

17. Viitasalo, A. *et al.* The effects of a 2-year individualized and family-based lifestyle intervention on physical activity, sedentary behavior and diet in children. *Prev Med* **87**, 81-8 (2016).
18. Newnham, J.P., Evans, S.F., Michael, C.A., Stanley, F.J. & Landau, L.I. Effects of frequent ultrasound during pregnancy: a randomised controlled trial. *Lancet* **342**, 887-91 (1993).
19. Straker, L. *et al.* Cohort Profile: The Western Australian Pregnancy Cohort (Raine) Study-Generation 2. *Int J Epidemiol* (2017).
20. Hofman, A. *et al.* The Rotterdam Study: 2016 objectives and design update. *Eur J Epidemiol* **30**, 661-708 (2015).
21. Cummings, S.R. *et al.* Appendicular bone density and age predict hip fracture in women. The Study of Osteoporotic Fractures Research Group. *JAMA* **263**, 665-8 (1990).
22. Yang, J. *et al.* Conditional and joint multiple-SNP analysis of GWAS summary statistics identifies additional variants influencing complex traits. *Nature Genetics* **44**, 369-U170 (2012).
23. Zheng, H.F. *et al.* Whole-genome sequencing identifies EN1 as a determinant of bone density and fracture. *Nature* **526**, 112-7 (2015).
24. Morris, J.A. *et al.* An atlas of genetic influences on osteoporosis in humans and mice. *Nat Genet* **51**, 258-266 (2019).
25. Westra, H.J. *et al.* Systematic identification of trans eQTLs as putative drivers of known disease associations. *Nat Genet* **45**, 1238-1243 (2013).
