# Supplementary material for: Bone mineral density loci specific to the skull portray potential pleiotropic effects on craniosynostosis
Source: Commun Biol. 2023 Jul 4;6:691. doi: 10.1038/s42003-023-04869-0 (PMC10319806; doi:10.1038/s42003-023-04869-0)
Supplement: Supplementary file 3 — Description of Additional Supplementary Files [file 42003_2023_4869_MOESM3_ESM.pdf]

Description of Additional Supplementary Files

File name: Supplementary Data 1

Description: Genotyping, Phenotyping and analysis description per cohort

File name: Supplementary Data 2

Description: Participant descriptions per cohort

File name: Supplementary Data 3

Description: Regional plots for all GWS loci

File name: Supplementary Data 4

Description: Lookup of all known independent markers associated with bone phenotypes.

File name: Supplementary Data 5

Description: GARFIELD analyses, source data for Figure 2

File name: Supplementary Data 6

Description: DEPICT enrichment analyses, source data for Figure 3b.

File name: Supplementary Data 7

Description: Relevance of SK-BMD implicated genes across mice and human datasets.
