# Supplementary material for: Bone mineral density loci specific to the skull portray potential pleiotropic effects on craniosynostosis
Source: Commun Biol. 2023 Jul 4;6:691. doi: 10.1038/s42003-023-04869-0 (PMC10319806; doi:10.1038/s42003-023-04869-0)
Supplement: Supplementary file 6 — Supplementary Data 3 [file 42003_2023_4869_MOESM6_ESM.zip › loci/chr10_131752499-132752499.pdf]

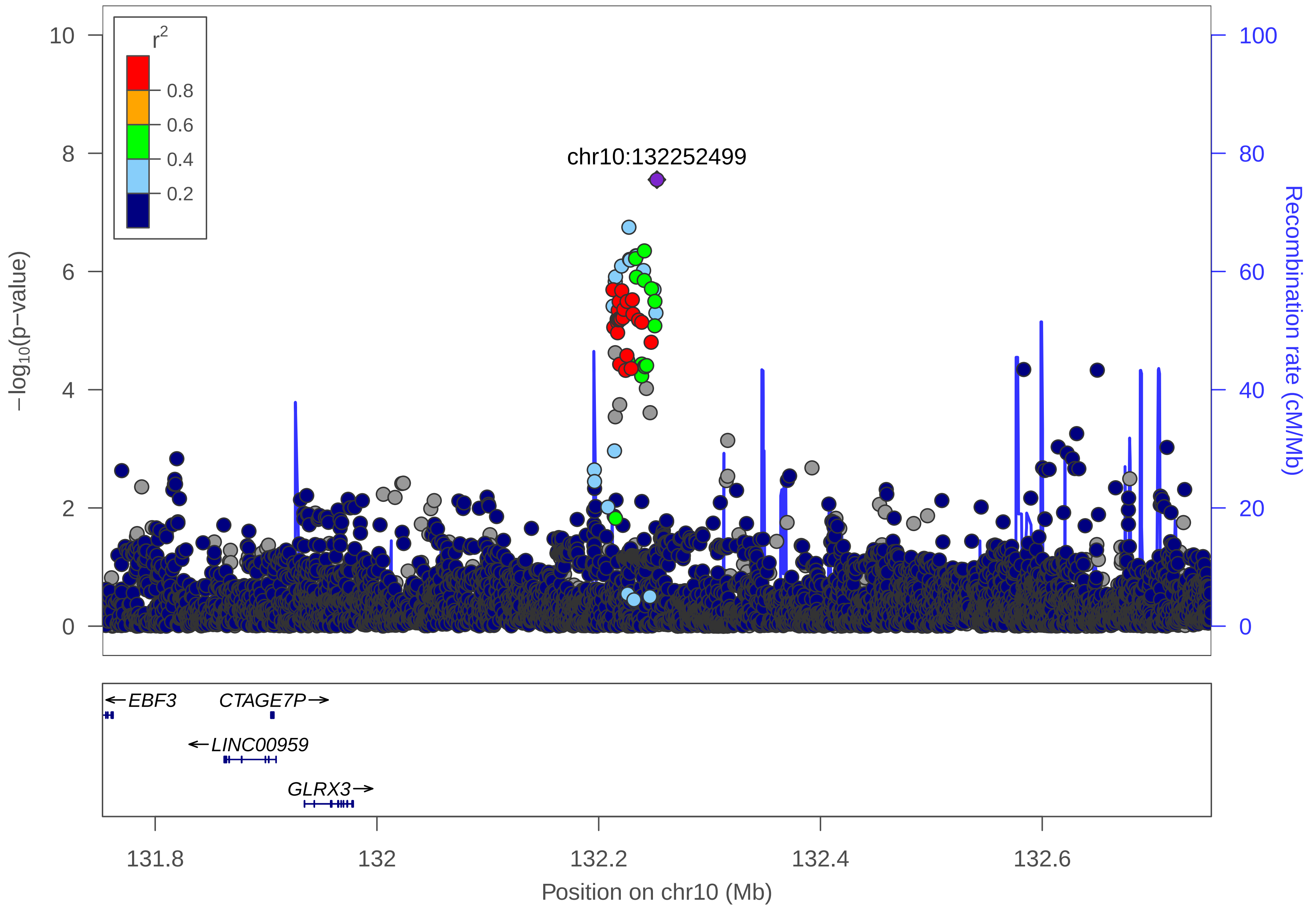

r2
0.8
0.6
0.4
0.2
chr10:132252499
−log10(p−value)
Recombination rate (cM/Mb)
0
2
4
6
8
10
0
20
40
60
80
100
EBF3
CTAGE7P
LINC00959
GLRX3
131.8
132
132.2
132.4
132.6
Position on chr10 (Mb)

| | |
|---|---|
| date: | Wed Aug 1 12:50:15 2018 |
| build: | hg19 |
| display range: | chr10:131752499−132752499 [131752499−132752499] |
| hilite range: | 0 − 0 [ 0 − 0 ] |
| reference SNP: | chr10:132252499 |
| number of SNPs plotted: | 6292 |
| min P−value: | 2.8E−8 [chr10:132252499] |
| max P−value: | 1E0 [chr10:132639807] |

Make more plots at http://csg.sph.umich.edu/locuszoom/
