# Supplementary material for: Bone mineral density loci specific to the skull portray potential pleiotropic effects on craniosynostosis
Source: Commun Biol. 2023 Jul 4;6:691. doi: 10.1038/s42003-023-04869-0 (PMC10319806; doi:10.1038/s42003-023-04869-0)
Supplement: Supplementary file 6 — Supplementary Data 3 [file 42003_2023_4869_MOESM6_ESM.zip › loci/chr10_53927825-54927825.pdf]

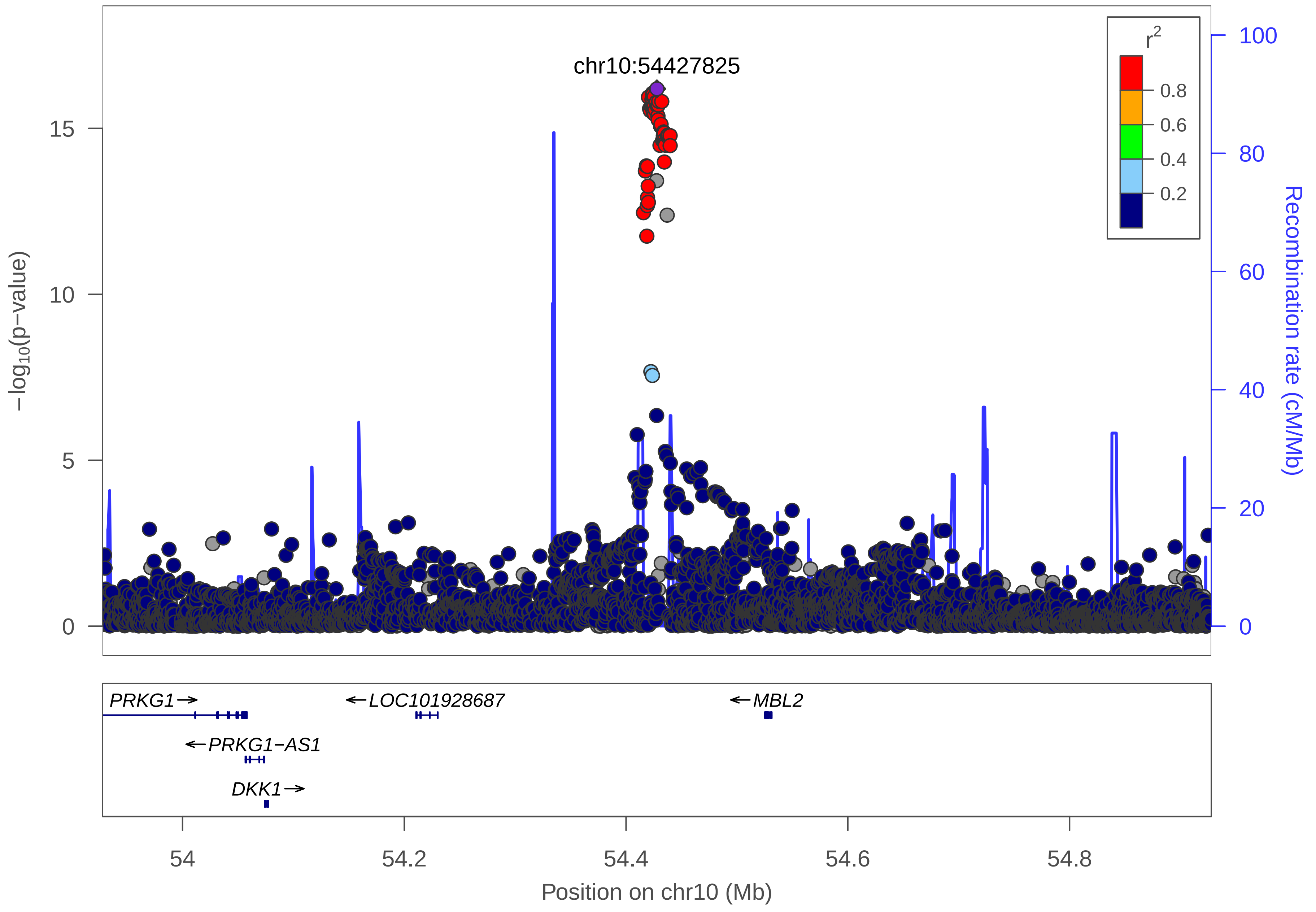

chr10:54427825
r2
0.8
0.6
0.4
0.2
15
10
5
0
−log10(p−value)
100
80
60
40
20
0
Recombination rate (cM/Mb)
PRKG1→
←LOC101928687
←MBL2
←PRKG1−AS1
DKK1→
54
54.2
54.4
54.6
54.8
Position on chr10 (Mb)

date:	Wed Aug 1 12:49:38 2018
build:	hg19
display range:	chr10:53927825−54927825 [53927825−54927825]
hilite range:	0 − 0 [0 − 0]
reference SNP:	chr10:54427825
number of SNPs plotted:	4853
min P−value:	6.42E−17 [chr10:54427825]
max P−value:	10E−1 [chr10:54049721]

Make more plots at http://csg.sph.umich.edu/locuszoom/
