# Supplementary material for: Bone mineral density loci specific to the skull portray potential pleiotropic effects on craniosynostosis
Source: Commun Biol. 2023 Jul 4;6:691. doi: 10.1038/s42003-023-04869-0 (PMC10319806; doi:10.1038/s42003-023-04869-0)
Supplement: Supplementary file 6 — Supplementary Data 3 [file 42003_2023_4869_MOESM6_ESM.zip › loci/chr11_1-759575.pdf]

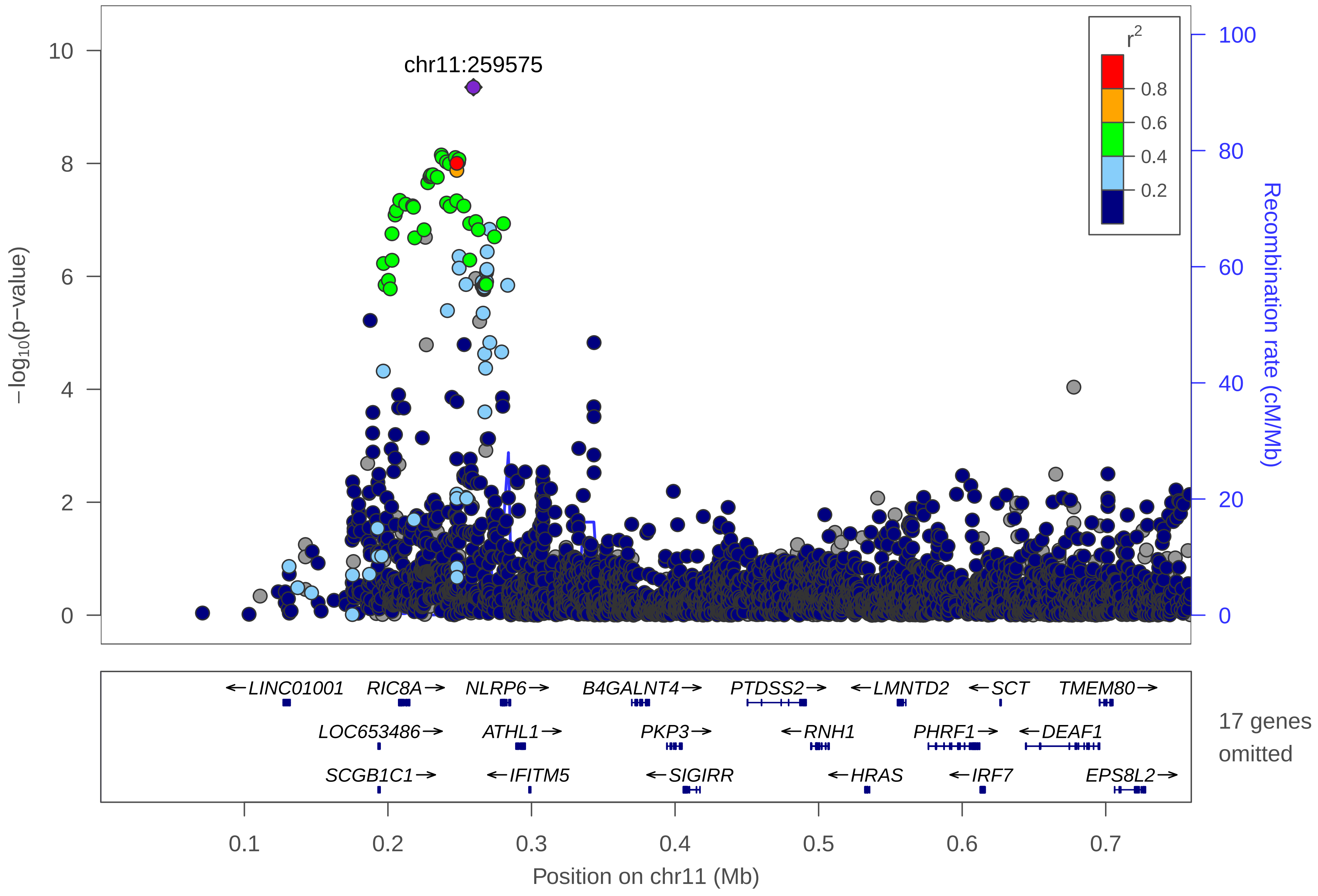
chr11:259575
r2
0.8
0.6
0.4
0.2
−log10(p−value)
Recombination rate (cM/Mb)
LINC01001
RIC8A
NLRP6
B4GALNT4
PTDSS2
LMNTD2
SCT
TMEM80
LOC653486
ATHL1
PKP3
RNH1
PHRF1
DEAF1
SCGB1C1
IFITM5
SIGIRR
HRAS
IRF7
EPS8L2
17 genes omitted
Position on chr11 (Mb)

date:	Wed Aug 1 12:50:52 2018
build:	hg19
display range:	chr11:1−759575 [1−759575]
hilite range:	0 − 0 [0 − 0]
reference SNP:	chr11:259575
number of SNPs plotted:	3653
min P−value:	4.47E−10 [chr11:259575]
max P−value:	10E−1 [chr11:660597]
omitted Genes:	ODF3, BET1L, MIR6743
omitted Genes:	SIRT3, PSMD13, IFITM2
omitted Genes:	IFITM1, IFITM3, ANO9
omitted Genes:	LRRC56, RASSF7, MIR210HG
omitted Genes:	MIR210, LOC143666, CDHR5
omitted Genes:	DRD4, TALDO1

Make more plots at http://csg.sph.umich.edu/locuszoom/
