# Supplementary material for: Bone mineral density loci specific to the skull portray potential pleiotropic effects on craniosynostosis
Source: Commun Biol. 2023 Jul 4;6:691. doi: 10.1038/s42003-023-04869-0 (PMC10319806; doi:10.1038/s42003-023-04869-0)
Supplement: Supplementary file 6 — Supplementary Data 3 [file 42003_2023_4869_MOESM6_ESM.zip › loci/chr11_121417163-122417163.pdf]

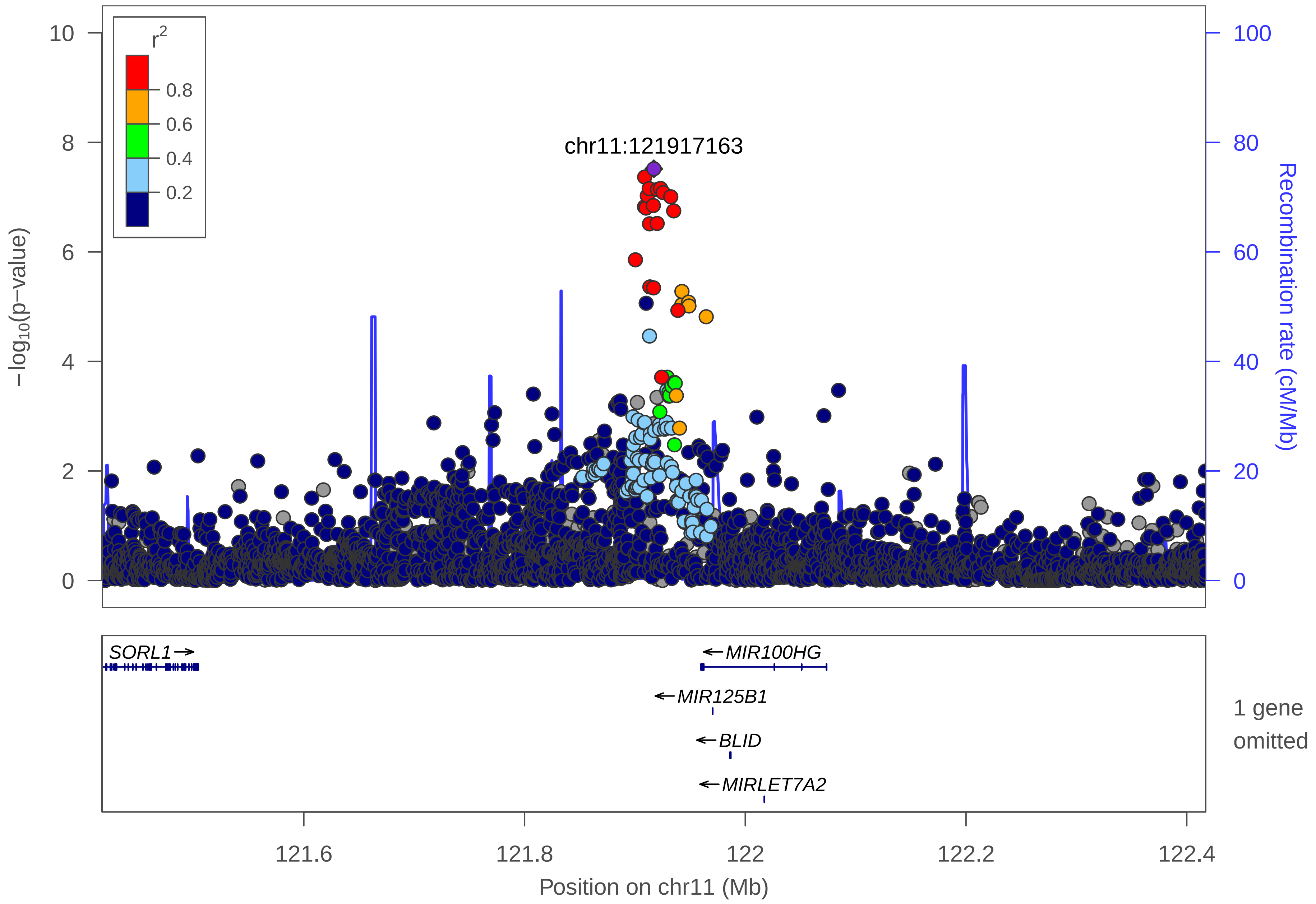

r2
0.8
0.6
0.4
0.2
chr11:121917163
10
8
6
4
2
0
100
80
60
40
20
0
−log10(p−value)
Recombination rate (cM/Mb)
SORL1
MIR100HG
MIR125B1
BLID
MIRLET7A2
1 gene omitted
121.6
121.8
122
122.2
122.4
Position on chr11 (Mb)

date:	Wed Aug 1 12:56:13 2018
build:	hg19
display range:	chr11:121417163−122417163 [121417163−122417163]
hilite range:	0 − 0 [0 − 0]
reference SNP:	chr11:121917163
number of SNPs plotted:	3418
min P−value:	3.03E−8 [chr11:121917163]
max P−value:	10E−1 [chr11:121501755]
omitted Genes:	MIR100

Make more plots at http://csg.sph.umich.edu/locuszoom/
