# Supplementary material for: Bone mineral density loci specific to the skull portray potential pleiotropic effects on craniosynostosis
Source: Commun Biol. 2023 Jul 4;6:691. doi: 10.1038/s42003-023-04869-0 (PMC10319806; doi:10.1038/s42003-023-04869-0)
Supplement: Supplementary file 6 — Supplementary Data 3 [file 42003_2023_4869_MOESM6_ESM.zip › loci/chr11_15603307-16903307.pdf]

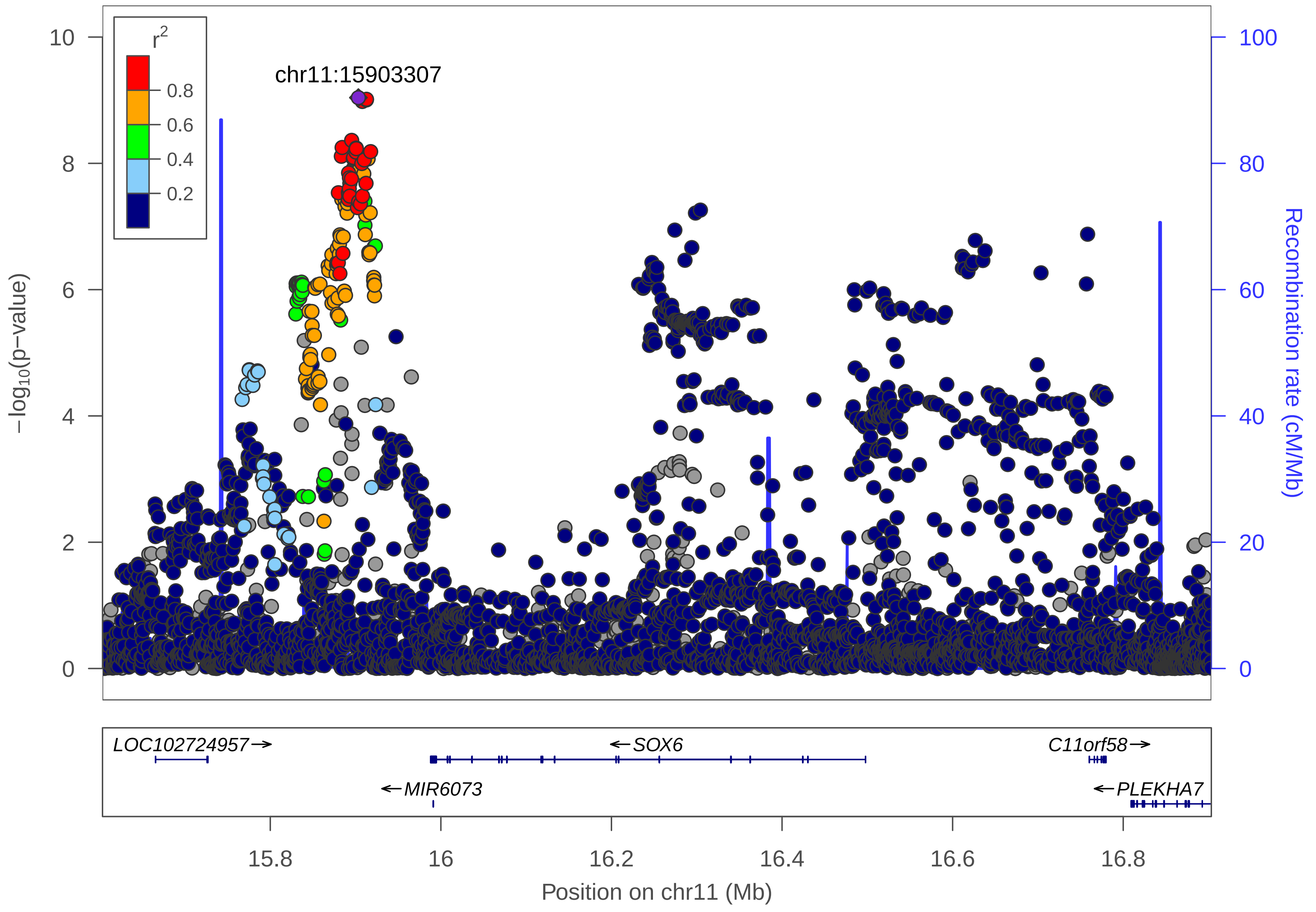

r2
0.8
0.6
0.4
0.2
chr11:15903307
−log10(p−value)
Recombination rate (cM/Mb)
10
8
6
4
2
0
100
80
60
40
20
0
LOC102724957→
←SOX6
C11orf58→
←MIR6073
←PLEKHA7
15.8
16
16.2
16.4
16.6
16.8
Position on chr11 (Mb)

date:	Wed Aug 1 15:09:18 2018
build:	hg19
display range:	chr11:15603307−16903307 [15603307−16903307]
hilite range:	0 − 0 [0 − 0]
reference SNP:	chr11:15903307
number of SNPs plotted:	4106
min P−value:	9.11E−10 [chr11:15903307]
max P−value:	10E−1 [chr11:15885932]

Make more plots at http://csg.sph.umich.edu/locuszoom/
