# Supplementary material for: Bone mineral density loci specific to the skull portray potential pleiotropic effects on craniosynostosis
Source: Commun Biol. 2023 Jul 4;6:691. doi: 10.1038/s42003-023-04869-0 (PMC10319806; doi:10.1038/s42003-023-04869-0)
Supplement: Supplementary file 6 — Supplementary Data 3 [file 42003_2023_4869_MOESM6_ESM.zip › loci/chr11_26963564-27963564.pdf]

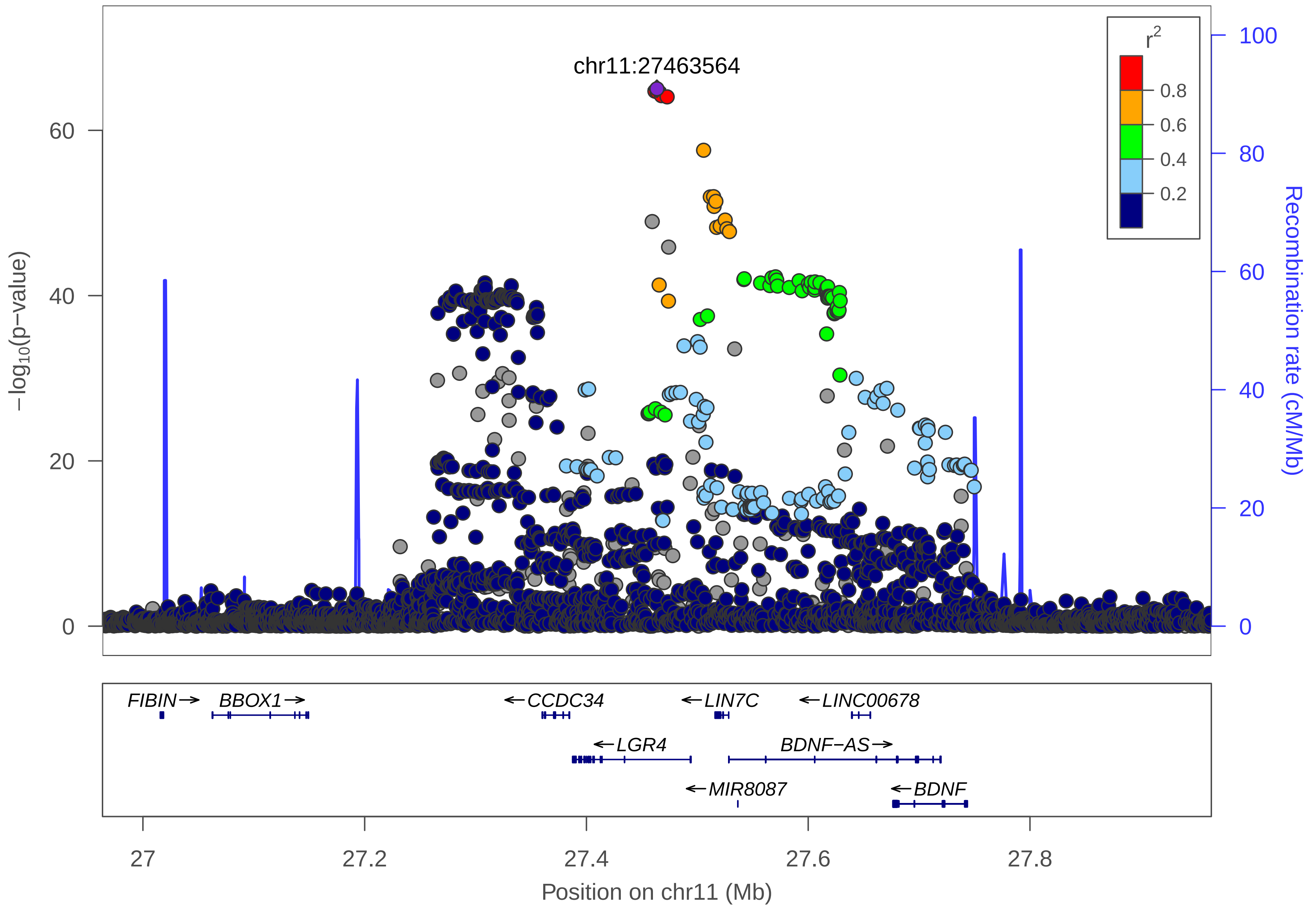

chr11:27463564
r2
0.8
0.6
0.4
0.2
−log10(p−value)
0
20
40
60
Recombination rate (cM/Mb)
0
20
40
60
80
100
FIBIN→
BBOX1→
←CCDC34
←LIN7C
←LINC00678
←LGR4
BDNF−AS→
←MIR8087
←BDNF
27
27.2
27.4
27.6
27.8
Position on chr11 (Mb)

date:	Wed Aug 1 12:53:50 2018
build:	hg19
display range:	chr11:26963564−27963564 [26963564−27963564]
hilite range:	0 − 0 [0 − 0]
reference SNP:	chr11:27463564
number of SNPs plotted:	3593
min P−value:	9.32E−66 [chr11:27463564]
max P−value:	9.99E−1 [chr11:27048328]

Make more plots at http://csg.sph.umich.edu/locuszoom/
