# Supplementary material for: Bone mineral density loci specific to the skull portray potential pleiotropic effects on craniosynostosis
Source: Commun Biol. 2023 Jul 4;6:691. doi: 10.1038/s42003-023-04869-0 (PMC10319806; doi:10.1038/s42003-023-04869-0)
Supplement: Supplementary file 6 — Supplementary Data 3 [file 42003_2023_4869_MOESM6_ESM.zip › loci/chr11_34583633-35583633.pdf]

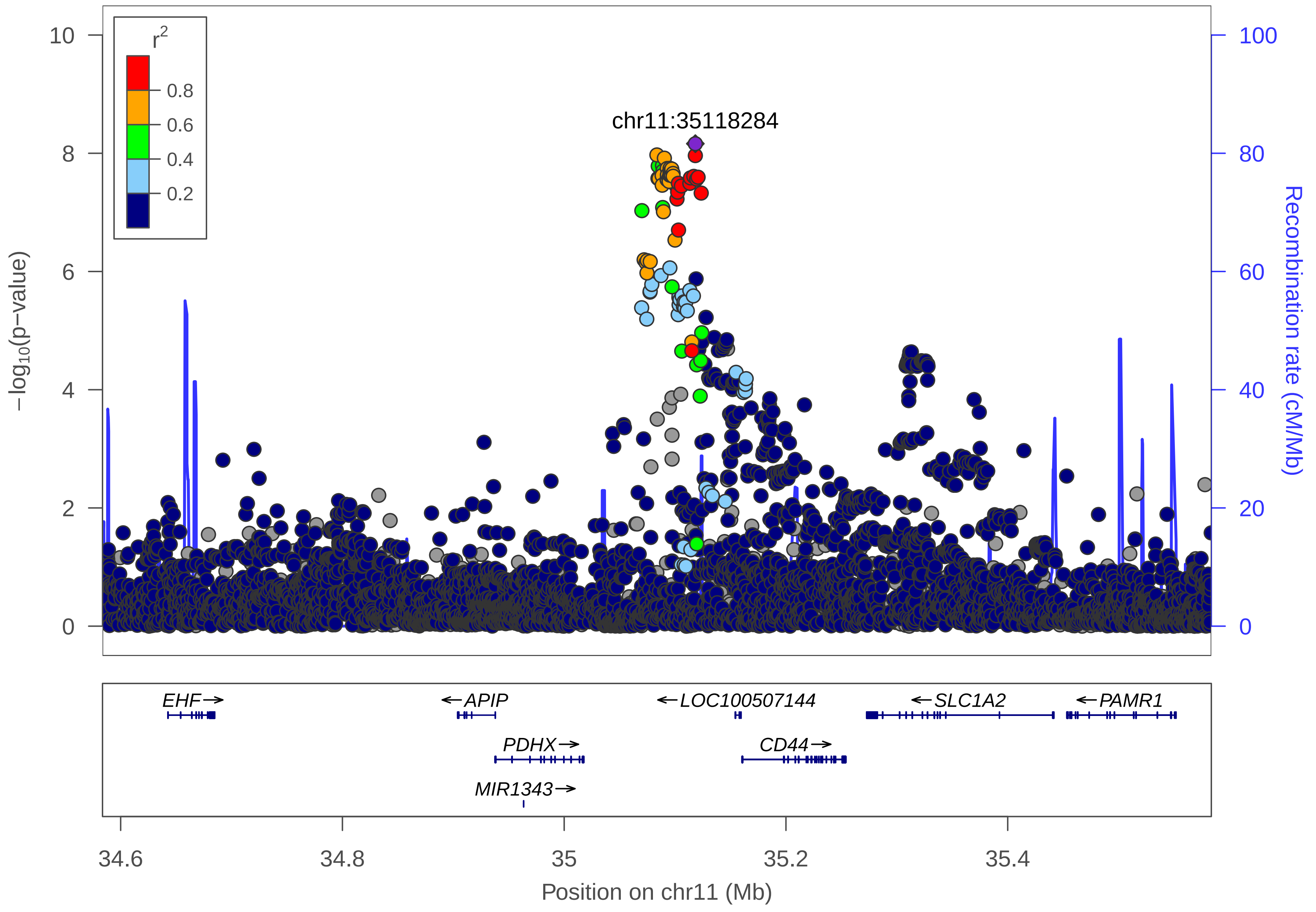

r2
0.8
0.6
0.4
0.2
chr11:35118284
−log10(p−value)
Recombination rate (cM/Mb)
10
8
6
4
2
0
100
80
60
40
20
0
EHF→
←APIP
←LOC100507144
←SLC1A2
←PAMR1
PDHX→
CD44→
MIR1343→
34.6
34.8
35
35.2
35.4
Position on chr11 (Mb)

date:	Wed Aug 1 12:54:26 2018
build:	hg19
display range:	chr11:34583633−35583633 [34583633−35583633]
hilite range:	0 − 0 [0 − 0]
reference SNP:	chr11:35118284
number of SNPs plotted:	4913
min P−value:	6.88E−9 [chr11:35118284]
max P−value:	1E0 [chr11:35568339]

Make more plots at http://csg.sph.umich.edu/locuszoom/
