# Supplementary material for: Bone mineral density loci specific to the skull portray potential pleiotropic effects on craniosynostosis
Source: Commun Biol. 2023 Jul 4;6:691. doi: 10.1038/s42003-023-04869-0 (PMC10319806; doi:10.1038/s42003-023-04869-0)
Supplement: Supplementary file 6 — Supplementary Data 3 [file 42003_2023_4869_MOESM6_ESM.zip › loci/chr11_46115633-47615633.pdf]

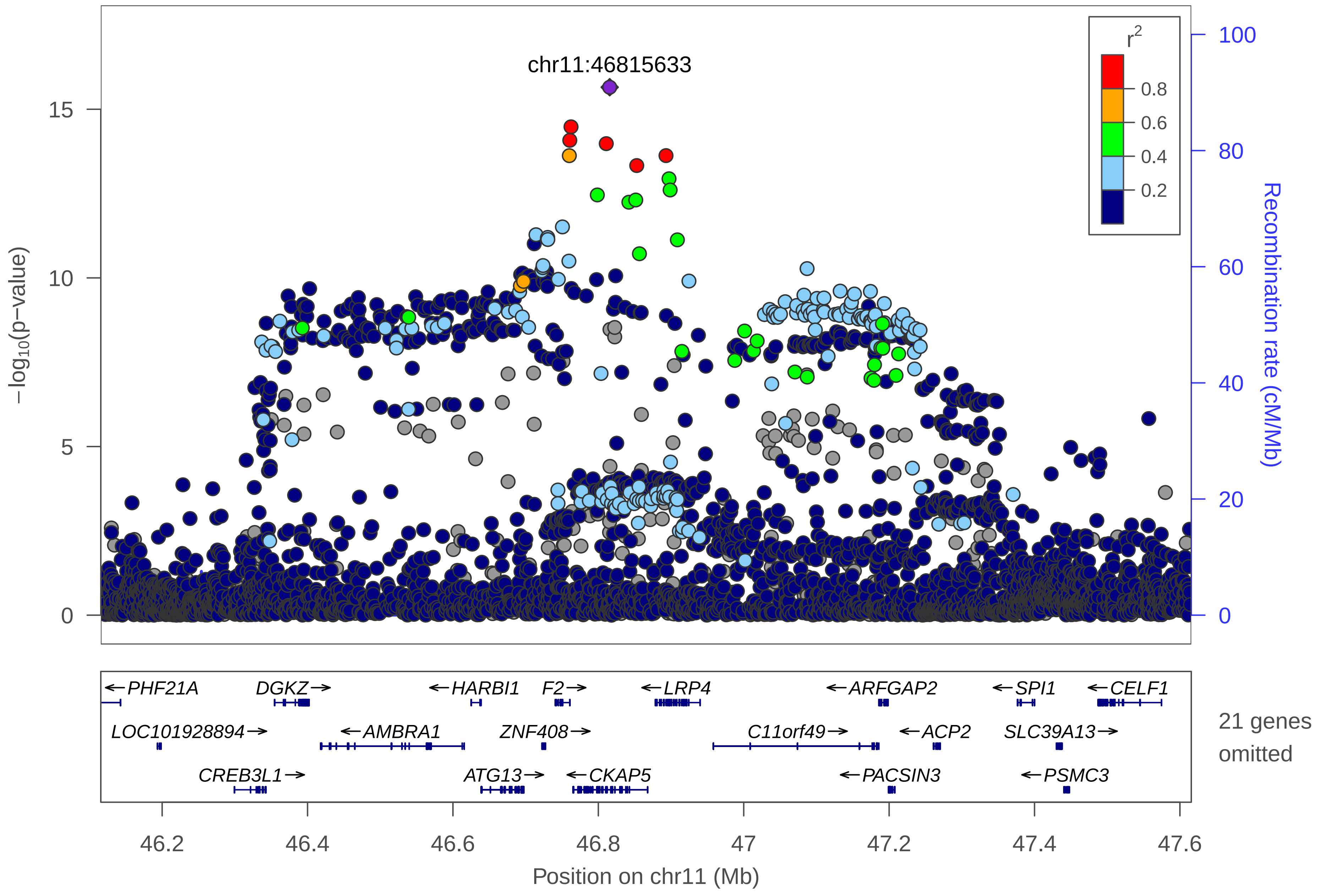
chr11:46815633
r²
0.8
0.6
0.4
0.2
−log10(p−value)
15
10
5
0
Recombination rate (cM/Mb)
100
80
60
40
20
0
PHF21A
DGKZ
HARBI1
F2
LRP4
ARFGAP2
SPI1
CELF1
LOC101928894
AMBRA1
ZNF408
C11orf49
ACP2
SLC39A13
CREB3L1
ATG13
CKAP5
PACSIN3
PSMC3
21 genes omitted
46.2
46.4
46.6
46.8
47
47.2
47.4
47.6
Position on chr11 (Mb)

date:	Wed Aug 1 15:17:35 2018
build:	hg19
display range:	chr11:46115633−47615633 [46115633−47615633]
hilite range:	0 − 0 [0 − 0]
reference SNP:	chr11:46815633
number of SNPs plotted:	3800
min P−value:	2.22E−16 [chr11:46815633]
max P−value:	9.99E−1 [chr11:46818081]
omitted Genes:	MIR4688, MDK, CHRM4
omitted Genes:	MIR3160−1, MIR3160−2, ARHGAP1
omitted Genes:	MIR5582, SNORD67, LRP4−AS1
omitted Genes:	MIR6745, DDB2, NR1H3
omitted Genes:	MADD, LOC101928943, MYBPC3
omitted Genes:	RAPSN, PTPMT1, KBTBD4
omitted Genes:	NDUFS3, FAM180B, C1QTNF4

Make more plots at http://csg.sph.umich.edu/locuszoom/
