# Supplementary material for: Bone mineral density loci specific to the skull portray potential pleiotropic effects on craniosynostosis
Source: Commun Biol. 2023 Jul 4;6:691. doi: 10.1038/s42003-023-04869-0 (PMC10319806; doi:10.1038/s42003-023-04869-0)
Supplement: Supplementary file 6 — Supplementary Data 3 [file 42003_2023_4869_MOESM6_ESM.zip › loci/chr11_67718290-68718290.pdf]

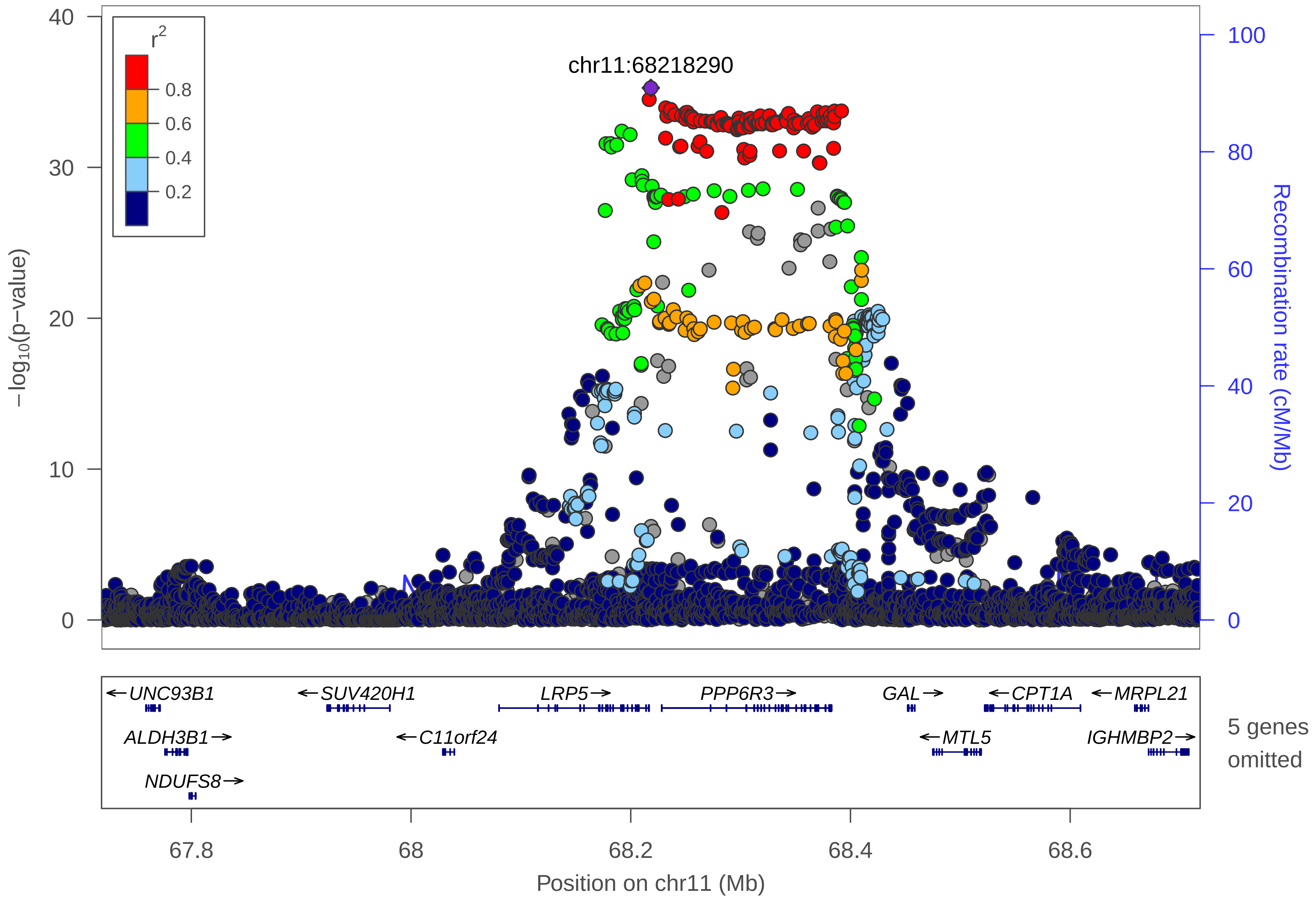

r2
0.8
0.6
0.4
0.2
chr11:68218290
−log10(p−value)
Recombination rate (cM/Mb)
0
10
20
30
40
100
80
60
40
20
0
UNC93B1
SUV420H1
LRP5
PPP6R3
GAL
CPT1A
MRPL21
ALDH3B1
C11orf24
MTL5
IGHMBP2
NDUFS8
5 genes omitted
67.8
68
68.2
68.4
68.6
Position on chr11 (Mb)

date:	Wed Aug 1 12:55:38 2018
build:	hg19
display range:	chr11:67718290−68718290 [67718290−68718290]
hilite range:	0 − 0 [0 − 0]
reference SNP:	chr11:68218290
number of SNPs plotted:	3683
min P−value:	5.39E−36 [chr11:68218290]
max P−value:	1E0 [chr11:67975088]
omitted Genes:	MIR7113, MIR4691, TCIRG1
omitted Genes:	MIR6753, CHKA

Make more plots at http://csg.sph.umich.edu/locuszoom/
