# Supplementary material for: Bone mineral density loci specific to the skull portray potential pleiotropic effects on craniosynostosis
Source: Commun Biol. 2023 Jul 4;6:691. doi: 10.1038/s42003-023-04869-0 (PMC10319806; doi:10.1038/s42003-023-04869-0)
Supplement: Supplementary file 6 — Supplementary Data 3 [file 42003_2023_4869_MOESM6_ESM.zip › loci/chr12_106873522-107873522.pdf]

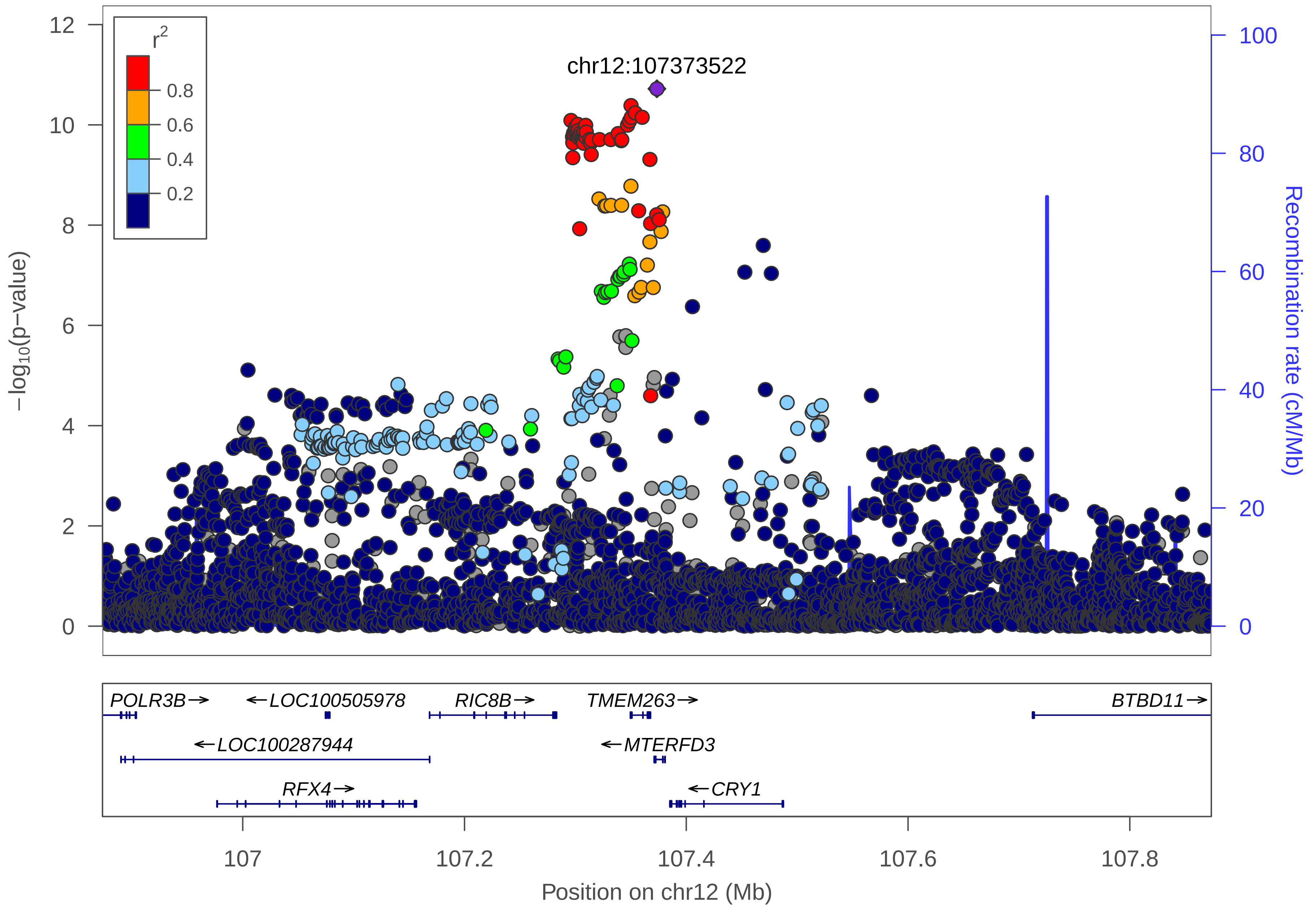

chr12:107373522
r2
0.8
0.6
0.4
0.2
−log10(p−value)
Recombination rate (cM/Mb)
POLR3B→
←LOC100505978
RIC8B→
TMEM263→
BTBD11→
←LOC100287944
←MTERFD3
RFX4→
←CRY1
107
107.2
107.4
107.6
107.8
Position on chr12 (Mb)

date:	Wed Aug 1 13:01:05 2018
build:	hg19
display range:	chr12:106873522−107873522 [106873522−107873522]
hilite range:	0 − 0 [0 − 0]
reference SNP:	chr12:107373522
number of SNPs plotted:	3701
min P−value:	1.91E−11 [chr12:107373522]
max P−value:	9.99E−1 [chr12:107864848]

Make more plots at http://csg.sph.umich.edu/locuszoom/
