# Supplementary material for: Bone mineral density loci specific to the skull portray potential pleiotropic effects on craniosynostosis
Source: Commun Biol. 2023 Jul 4;6:691. doi: 10.1038/s42003-023-04869-0 (PMC10319806; doi:10.1038/s42003-023-04869-0)
Supplement: Supplementary file 6 — Supplementary Data 3 [file 42003_2023_4869_MOESM6_ESM.zip › loci/chr12_28006044-29006044.pdf]

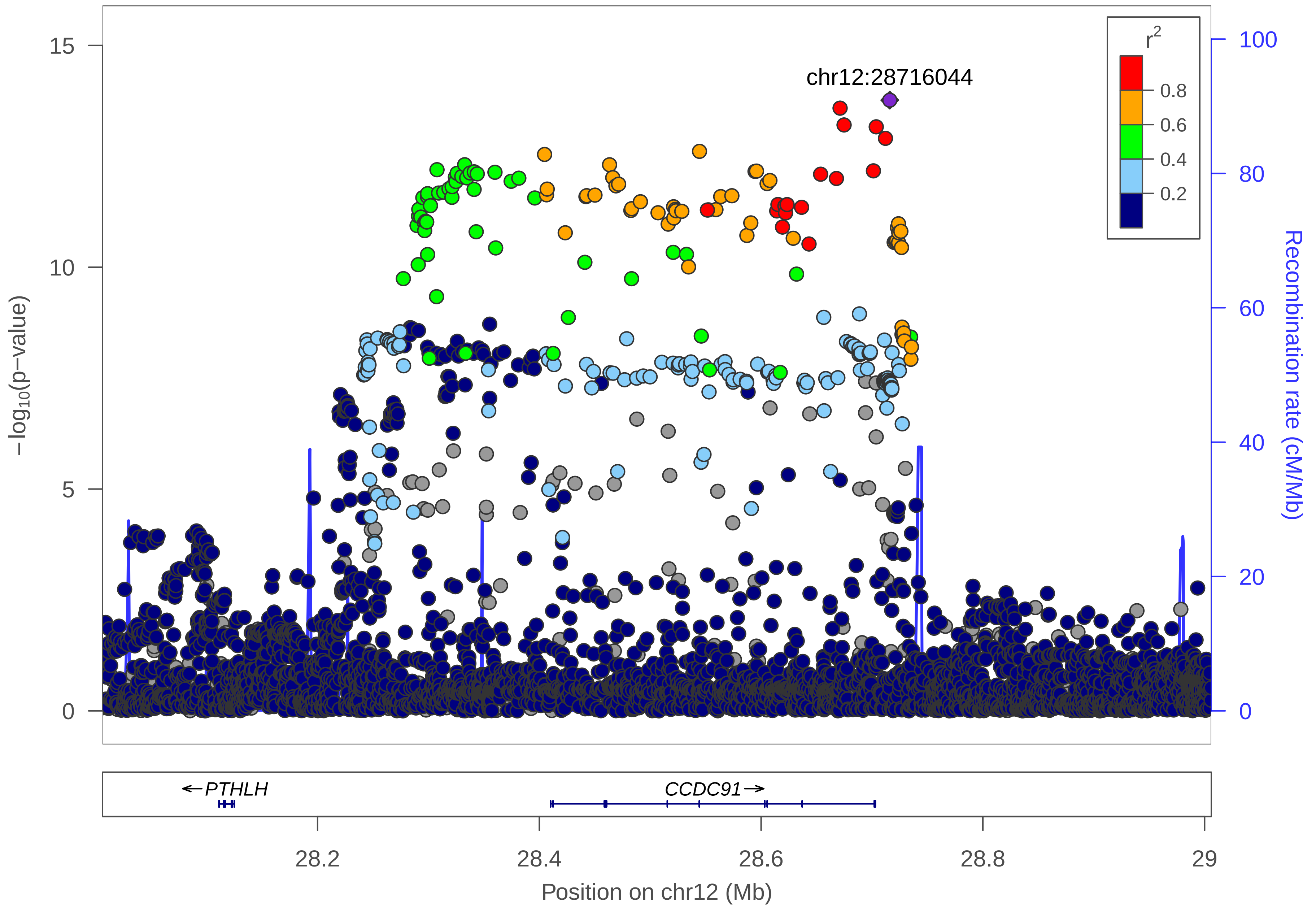

chr12:28716044
r2
0.8
0.6
0.4
0.2
15
10
5
0
100
80
60
40
20
0
−log10(p−value)
Recombination rate (cM/Mb)
←PTHLH
CCDC91→
28.2
28.4
28.6
28.8
29
Position on chr12 (Mb)

date:	Thu Aug 2 16:41:01 2018
build:	hg19
display range:	chr12:28006044−29006044 [28006044−29006044]
hilite range:	0 − 0 [0 − 0]
reference SNP:	chr12:28716044
number of SNPs plotted:	4289
min P−value:	1.71E−14 [chr12:28716044]
max P−value:	10E−1 [chr12:28507879]

Make more plots at http://csg.sph.umich.edu/locuszoom/
