# Supplementary material for: Bone mineral density loci specific to the skull portray potential pleiotropic effects on craniosynostosis
Source: Commun Biol. 2023 Jul 4;6:691. doi: 10.1038/s42003-023-04869-0 (PMC10319806; doi:10.1038/s42003-023-04869-0)
Supplement: Supplementary file 6 — Supplementary Data 3 [file 42003_2023_4869_MOESM6_ESM.zip › loci/chr12_49000000-50000000.pdf]

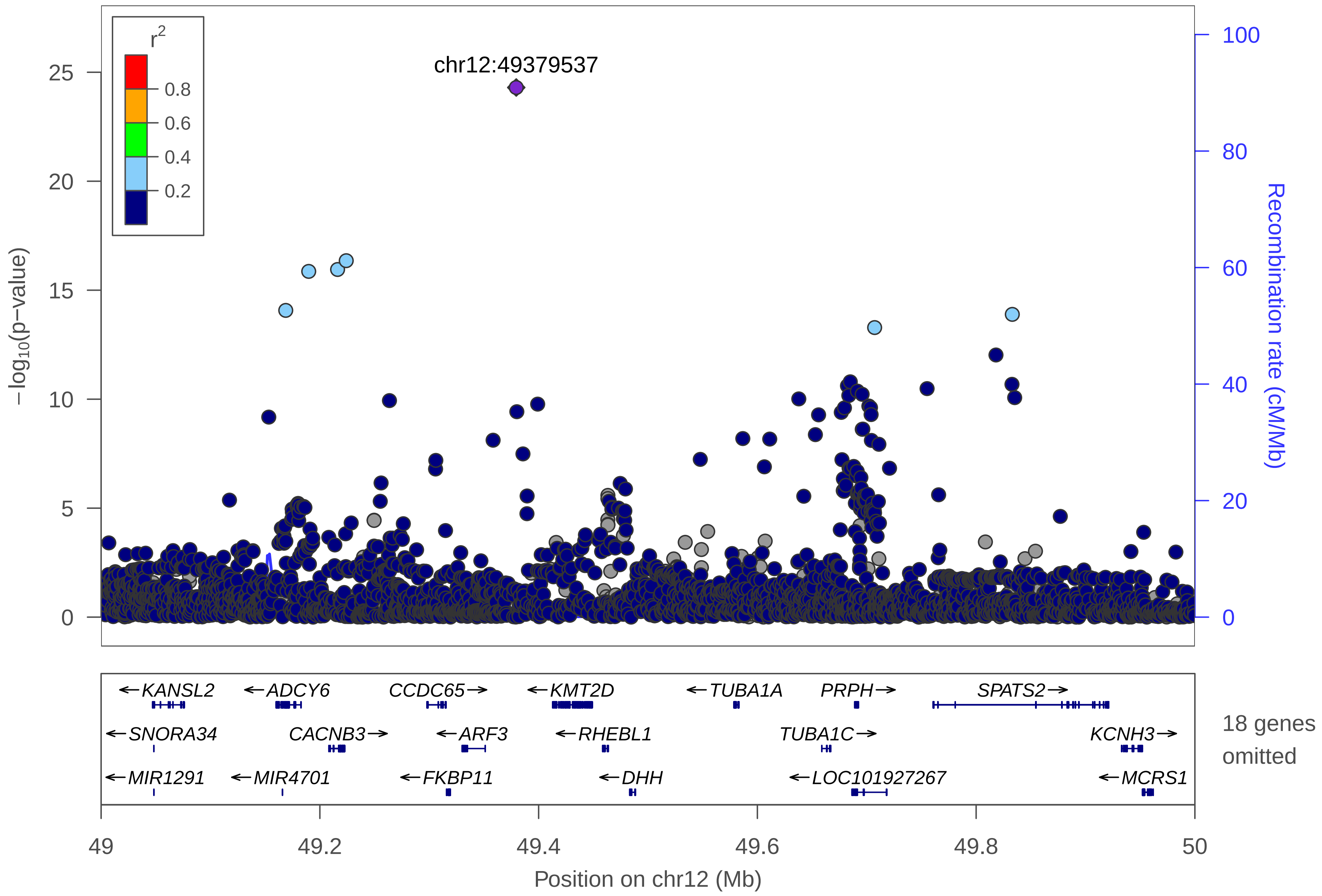

chr12:49379537
r2
0.8
0.6
0.4
0.2
−log10(p−value)
25
20
15
10
5
0
100
80
60
40
20
0
Recombination rate (cM/Mb)
←KANSL2
←ADCY6
CCDC65→
←KMT2D
←TUBA1A
PRPH→
SPATS2→
←SNORA34
CACNB3→
←ARF3
←RHEBL1
TUBA1C→
KCNH3→
←MIR1291
←MIR4701
←FKBP11
←DHH
←LOC101927267
←MCRS1
18 genes omitted
49
49.2
49.4
49.6
49.8
50
Position on chr12 (Mb)

date:	Wed Aug 1 15:25:35 2018
build:	hg19
display range:	chr12:49000000−50000000 [4.9e+07−5e+07]
hilite range:	0 − 0 [0 − 0]
reference SNP:	chr12:49379537
number of SNPs plotted:	3137
min P−value:	5.01E−25 [chr12:49379537]
max P−value:	9.97E−1 [chr12:49747498]
omitted Genes:	SNORA2A, SNORA2B, CCNT1
omitted Genes:	LINC00935, LOC100506125, DDX23
omitted Genes:	RND1, WNT10B, WNT1
omitted Genes:	DDN, PRKAG1, LMBR1L
omitted Genes:	TUBA1B, TROAP, C1QL4
omitted Genes:	DNAJC22, LOC100335030, FAM186B

Make more plots at http://csg.sph.umich.edu/locuszoom/
