# Supplementary material for: Bone mineral density loci specific to the skull portray potential pleiotropic effects on craniosynostosis
Source: Commun Biol. 2023 Jul 4;6:691. doi: 10.1038/s42003-023-04869-0 (PMC10319806; doi:10.1038/s42003-023-04869-0)
Supplement: Supplementary file 6 — Supplementary Data 3 [file 42003_2023_4869_MOESM6_ESM.zip › loci/chr12_89912755-90912755.pdf]

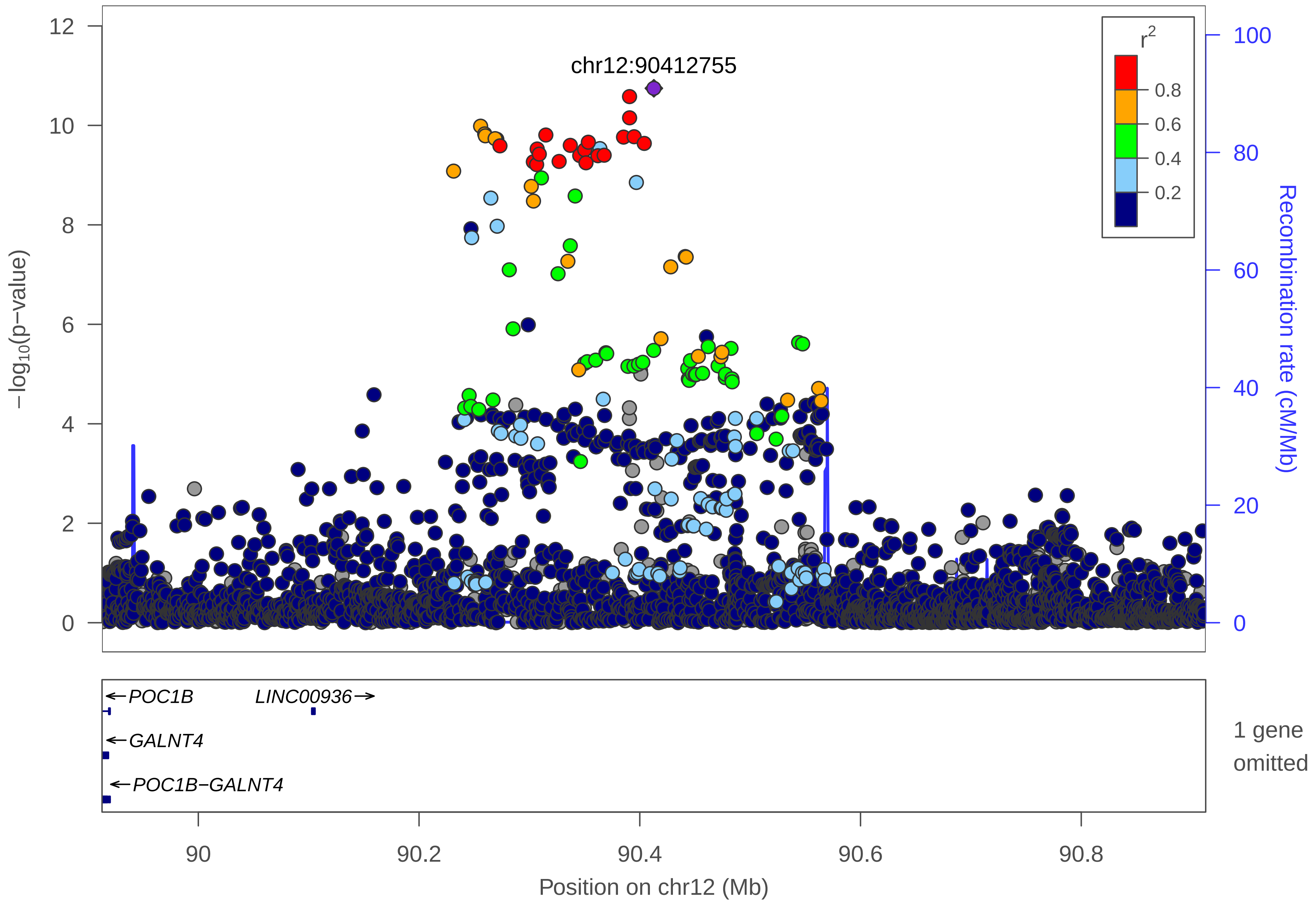

chr12:90412755
r2
0.8
0.6
0.4
0.2
−log10(p−value)
Recombination rate (cM/Mb)
12
10
8
6
4
2
0
100
80
60
40
20
0
←POC1B
LINC00936→
←GALNT4
←POC1B−GALNT4
1 gene omitted
90
90.2
90.4
90.6
90.8
Position on chr12 (Mb)

date:	Wed Aug 1 12:59:53 2018
build:	hg19
display range:	chr12:89912755−90912755 [89912755−90912755]
hilite range:	0 − 0 [0 − 0]
reference SNP:	chr12:90412755
number of SNPs plotted:	3252
min P−value:	1.79E−11 [chr12:90412755]
max P−value:	10E−1 [chr12:90806603]
omitted Genes:	ATP2B1

Make more plots at http://csg.sph.umich.edu/locuszoom/
