# Supplementary material for: Bone mineral density loci specific to the skull portray potential pleiotropic effects on craniosynostosis
Source: Commun Biol. 2023 Jul 4;6:691. doi: 10.1038/s42003-023-04869-0 (PMC10319806; doi:10.1038/s42003-023-04869-0)
Supplement: Supplementary file 6 — Supplementary Data 3 [file 42003_2023_4869_MOESM6_ESM.zip › loci/chr12_93482332-94482332.pdf]

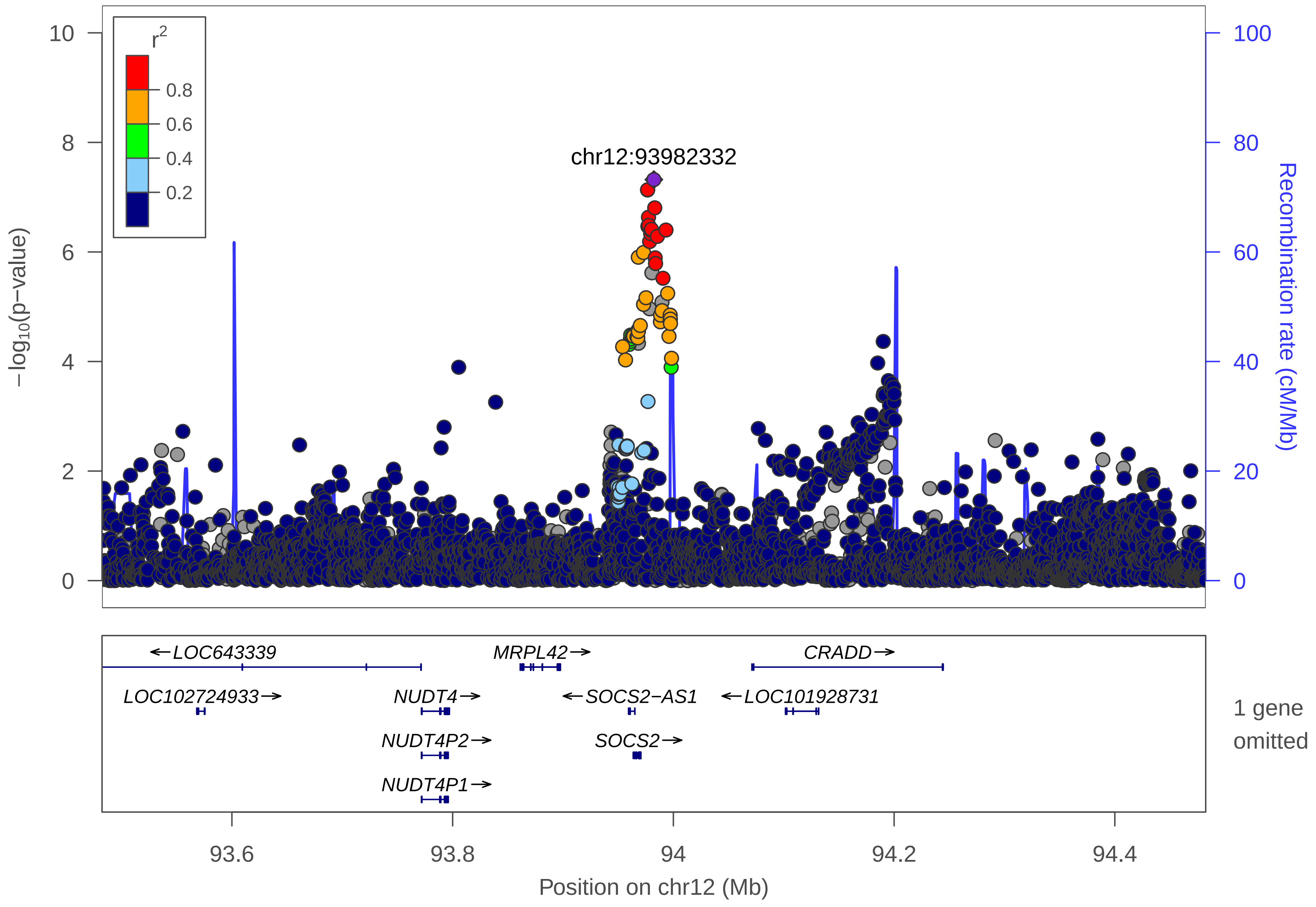

r2
0.8
0.6
0.4
0.2
chr12:93982332
−log10(p−value)
Recombination rate (cM/Mb)
10
8
6
4
2
0
100
80
60
40
20
0
←LOC643339
MRPL42→
CRADD→
LOC102724933→
NUDT4→
←SOCS2−AS1
←LOC101928731
NUDT4P2→
SOCS2→
NUDT4P1→
1 gene
omitted
93.6
93.8
94
94.2
94.4
Position on chr12 (Mb)

date:	Wed Aug 1 13:00:30 2018
build:	hg19
display range:	chr12:93482332−94482332 [93482332−94482332]
hilite range:	0 − 0 [0 − 0]
reference SNP:	chr12:93982332
number of SNPs plotted:	4401
min P−value:	4.78E−8 [chr12:93982332]
max P−value:	10E−1 [chr12:93494216]
omitted Genes:	UBE2N

Make more plots at http://csg.sph.umich.edu/locuszoom/
