# Supplementary material for: Bone mineral density loci specific to the skull portray potential pleiotropic effects on craniosynostosis
Source: Commun Biol. 2023 Jul 4;6:691. doi: 10.1038/s42003-023-04869-0 (PMC10319806; doi:10.1038/s42003-023-04869-0)
Supplement: Supplementary file 6 — Supplementary Data 3 [file 42003_2023_4869_MOESM6_ESM.zip › loci/chr13_36973386-37973386.pdf]

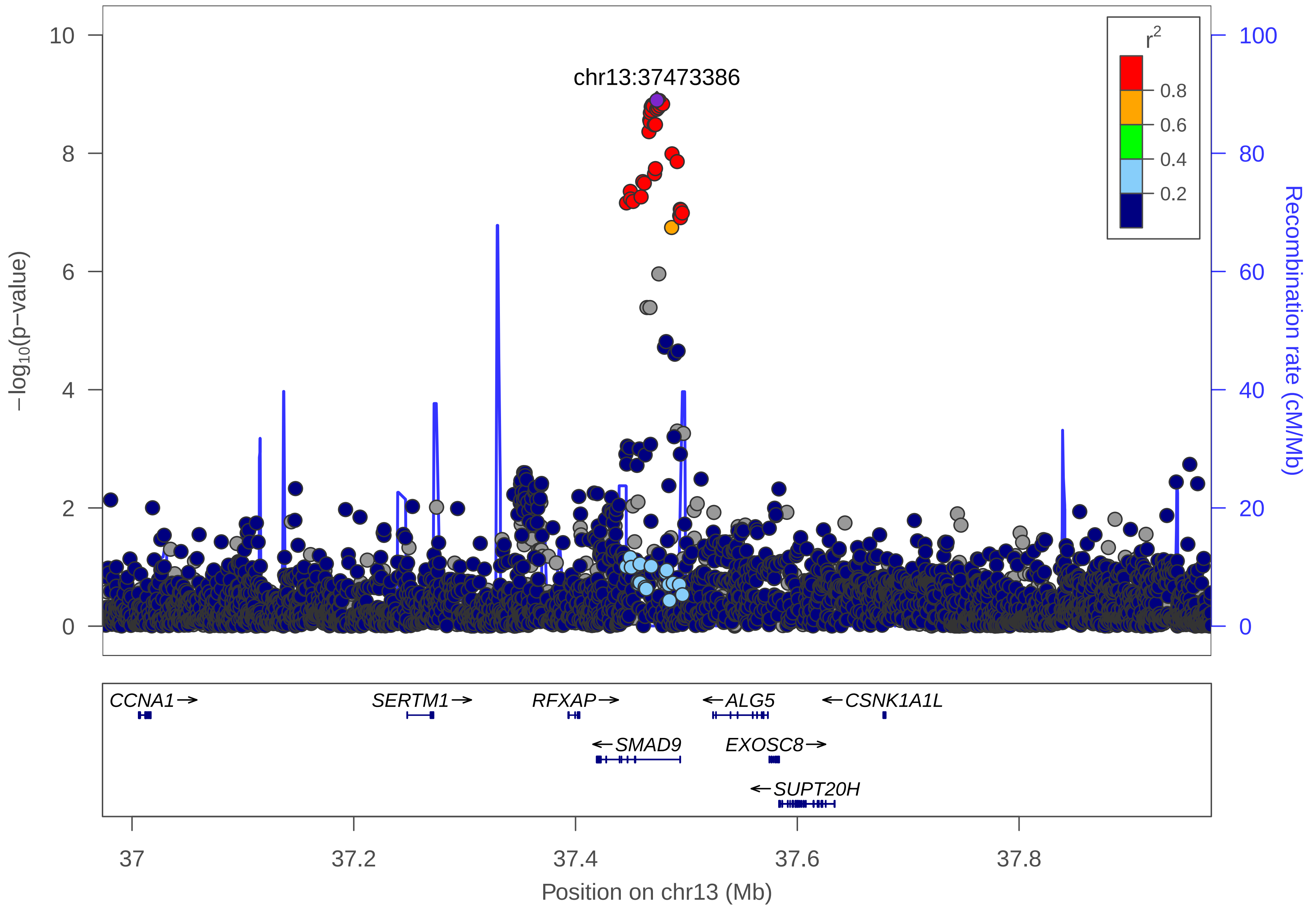

chr13:37473386
r2
0.8
0.6
0.4
0.2
10
8
6
4
2
0
−log10(p−value)
100
80
60
40
20
0
Recombination rate (cM/Mb)
CCNA1→
SERTM1→
RFXAP→
←ALG5
←CSNK1A1L
←SMAD9
EXOSC8→
←SUPT20H
37
37.2
37.4
37.6
37.8
Position on chr13 (Mb)

date:	Wed Aug 1 13:01:41 2018
build:	hg19
display range:	chr13:36973386−37973386 [36973386−37973386]
hilite range:	0 − 0 [0 − 0]
reference SNP:	chr13:37473386
number of SNPs plotted:	4045
min P−value:	1.27E−9 [chr13:37473386]
max P−value:	10E−1 [chr13:37911059]

Make more plots at http://csg.sph.umich.edu/locuszoom/
