# Supplementary material for: Bone mineral density loci specific to the skull portray potential pleiotropic effects on craniosynostosis
Source: Commun Biol. 2023 Jul 4;6:691. doi: 10.1038/s42003-023-04869-0 (PMC10319806; doi:10.1038/s42003-023-04869-0)
Supplement: Supplementary file 6 — Supplementary Data 3 [file 42003_2023_4869_MOESM6_ESM.zip › loci/chr13_42648546-43648546.pdf]

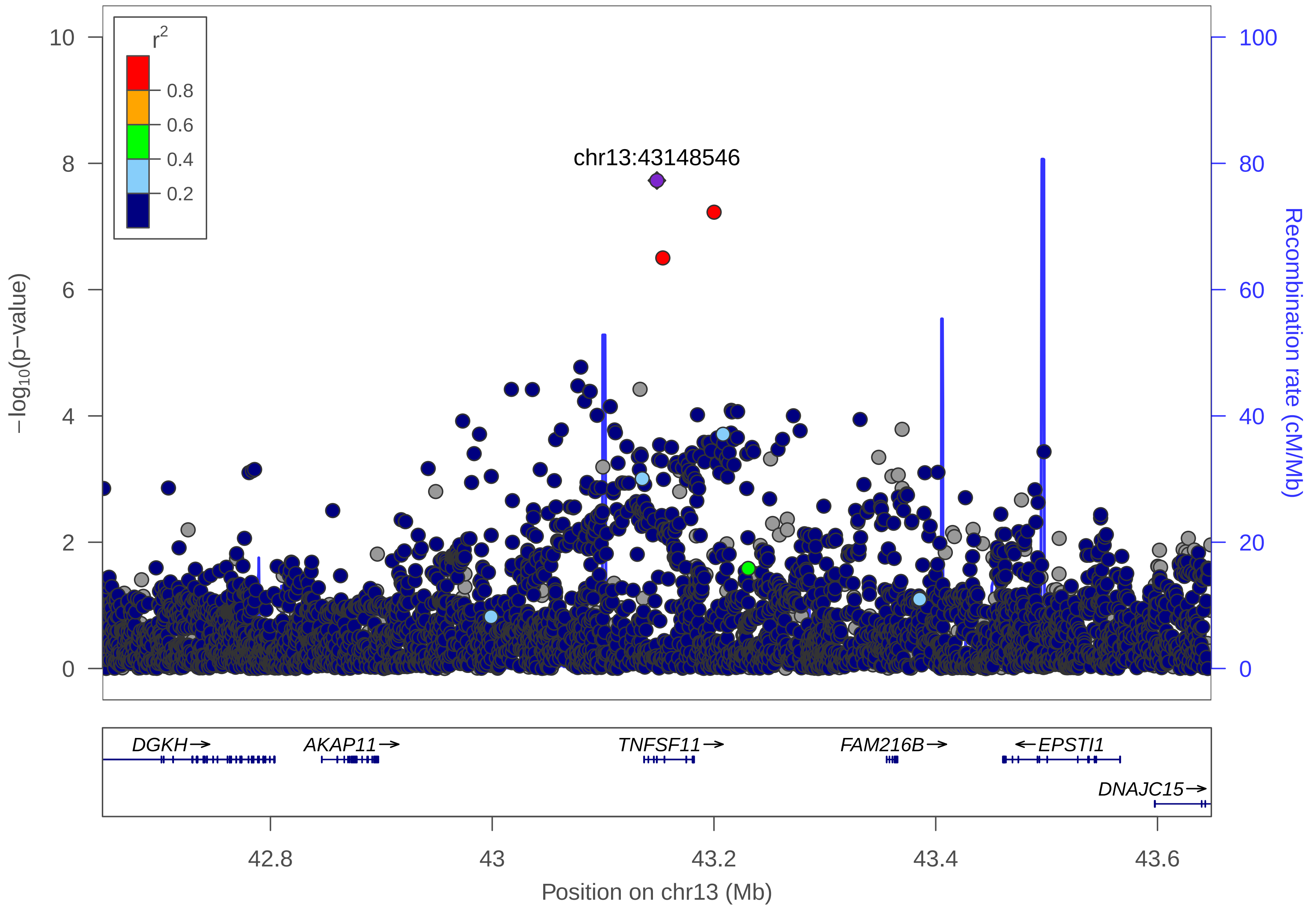

r2
0.8
0.6
0.4
0.2
chr13:43148546
−log10(p−value)
Recombination rate (cM/Mb)
10
8
6
4
2
0
100
80
60
40
20
0
DGKH→
AKAP11→
TNFSF11→
FAM216B→
←EPSTI1
DNAJC15→
42.8
43
43.2
43.4
43.6
Position on chr13 (Mb)

date:	Wed Aug 1 13:02:18 2018
build:	hg19
display range:	chr13:42648546−43648546 [42648546−43648546]
hilite range:	0 − 0 [0 − 0]
reference SNP:	chr13:43148546
number of SNPs plotted:	4552
min P−value:	1.87E−8 [chr13:43148546]
max P−value:	10E−1 [chr13:42902126]

Make more plots at http://csg.sph.umich.edu/locuszoom/
