# Supplementary material for: Bone mineral density loci specific to the skull portray potential pleiotropic effects on craniosynostosis
Source: Commun Biol. 2023 Jul 4;6:691. doi: 10.1038/s42003-023-04869-0 (PMC10319806; doi:10.1038/s42003-023-04869-0)
Supplement: Supplementary file 6 — Supplementary Data 3 [file 42003_2023_4869_MOESM6_ESM.zip › loci/chr16_1-892318.pdf]

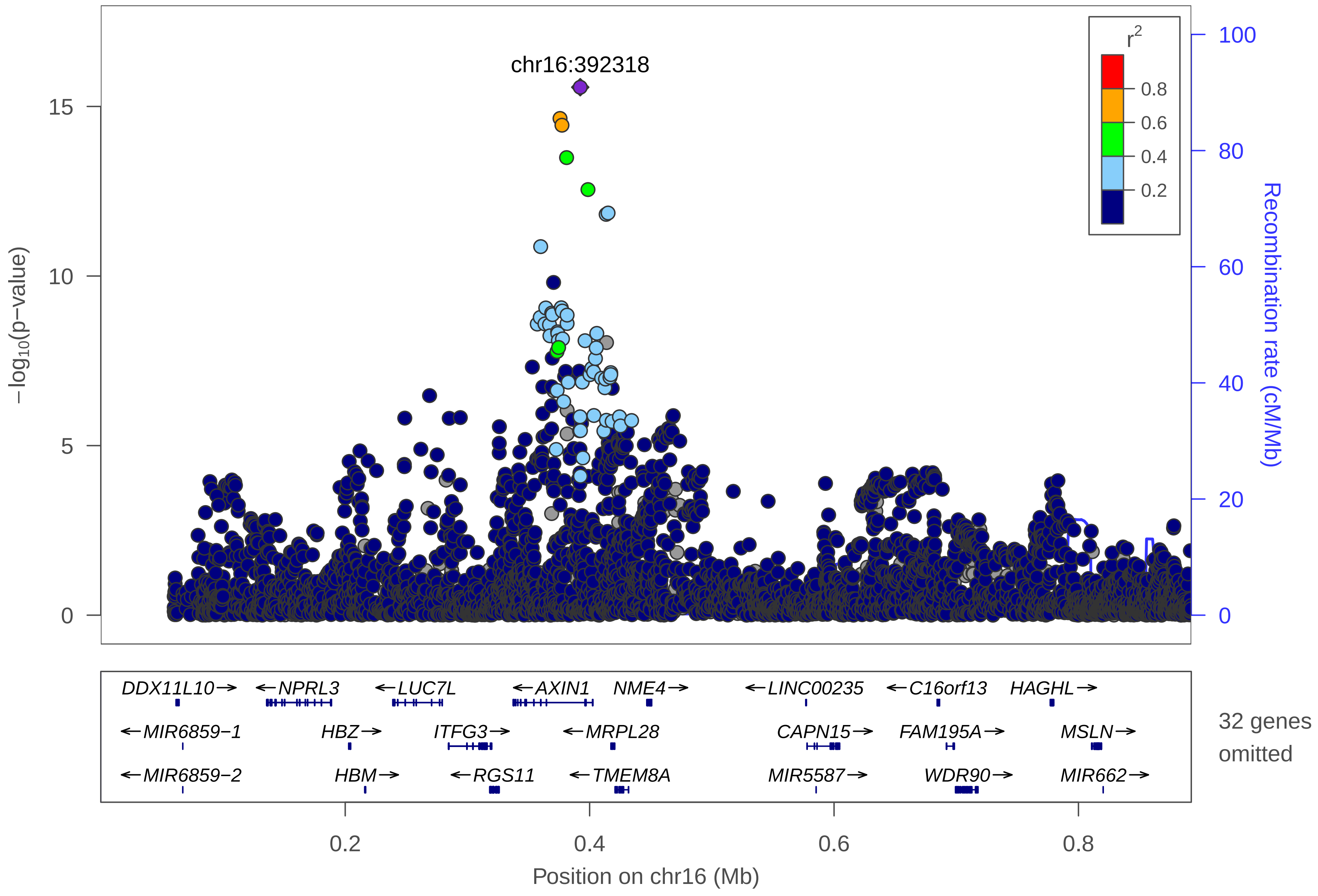

chr16:392318
−log10(p−value)
Recombination rate (cM/Mb)
r2
0.8
0.6
0.4
0.2
0
5
10
15
0
20
40
60
80
100
DDX11L10→
←NPRL3
←LUC7L
←AXIN1
NME4→
←LINC00235
←C16orf13
HAGHL→
←MIR6859−1
HBZ→
ITFG3→
←MRPL28
CAPN15→
FAM195A→
MSLN→
←MIR6859−2
HBM→
←RGS11
←TMEM8A
MIR5587→
WDR90→
MIR662→
32 genes omitted
0.2
0.4
0.6
0.8
Position on chr16 (Mb)

date:	Wed Aug 1 13:02:55 2018
build:	hg19
display range:	chr16:1−892318 [1−892318]
hilite range:	0 − 0 [0 − 0]
reference SNP:	chr16:392318
number of SNPs plotted:	4354
min P−value:	2.71E−16 [chr16:392318]
max P−value:	10E−1 [chr16:807347]
omitted Genes:	POLR3K, SNRNP25, RHBDF1
omitted Genes:	MPG, HBA2, HBA1
omitted Genes:	HBQ1, ARHGDIG, PDIA2
omitted Genes:	LOC100134368, DECR2, RAB11FIP3
omitted Genes:	MIR3176, PRR35, NHLRC4
omitted Genes:	PIGQ, RAB40C, WFIKKN1
omitted Genes:	RHOT2, RHBDL1, STUB1
omitted Genes:	JMJD8, WDR24, FBXL16
omitted Genes:	METRN, FAM173A, CCDC78
omitted Genes:	NARFL, RPUSD1, CHTF18
omitted Genes:	GNG13, PRR25

Make more plots at http://csg.sph.umich.edu/locuszoom/
