# Supplementary material for: Bone mineral density loci specific to the skull portray potential pleiotropic effects on craniosynostosis
Source: Commun Biol. 2023 Jul 4;6:691. doi: 10.1038/s42003-023-04869-0 (PMC10319806; doi:10.1038/s42003-023-04869-0)
Supplement: Supplementary file 6 — Supplementary Data 3 [file 42003_2023_4869_MOESM6_ESM.zip › loci/chr16_86682052-87682052.pdf]

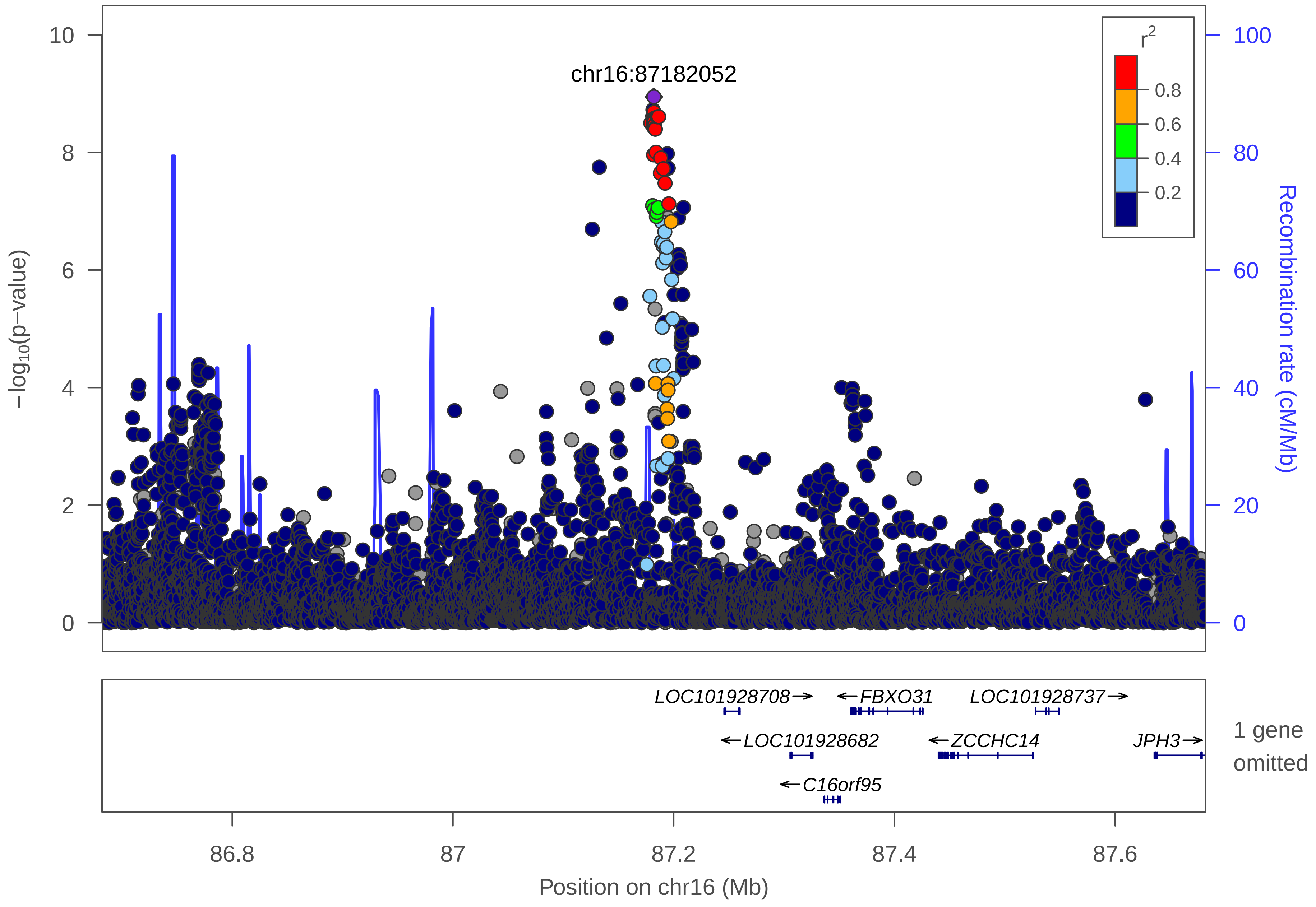
chr16:87182052
r2
0.8
0.6
0.4
0.2
−log10(p−value)
Recombination rate (cM/Mb)
0
2
4
6
8
10
0
20
40
60
80
100
LOC101928708→
←FBXO31
LOC101928737→
←LOC101928682
←ZCCHC14
JPH3→
←C16orf95
1 gene omitted
86.8
87
87.2
87.4
87.6
Position on chr16 (Mb)

date:	Wed Aug 1 13:03:33 2018
build:	hg19
display range:	chr16:86682052−87682052 [86682052−87682052]
hilite range:	0 − 0 [0 − 0]
reference SNP:	chr16:87182052
number of SNPs plotted:	6553
min P−value:	1.13E−9 [chr16:87182052]
max P−value:	1E0 [chr16:87643802]
omitted Genes:	MAP1LC3B

Make more plots at http://csg.sph.umich.edu/locuszoom/
