# Supplementary material for: Bone mineral density loci specific to the skull portray potential pleiotropic effects on craniosynostosis
Source: Commun Biol. 2023 Jul 4;6:691. doi: 10.1038/s42003-023-04869-0 (PMC10319806; doi:10.1038/s42003-023-04869-0)
Supplement: Supplementary file 6 — Supplementary Data 3 [file 42003_2023_4869_MOESM6_ESM.zip › loci/chr17_1568932-2568932.pdf]

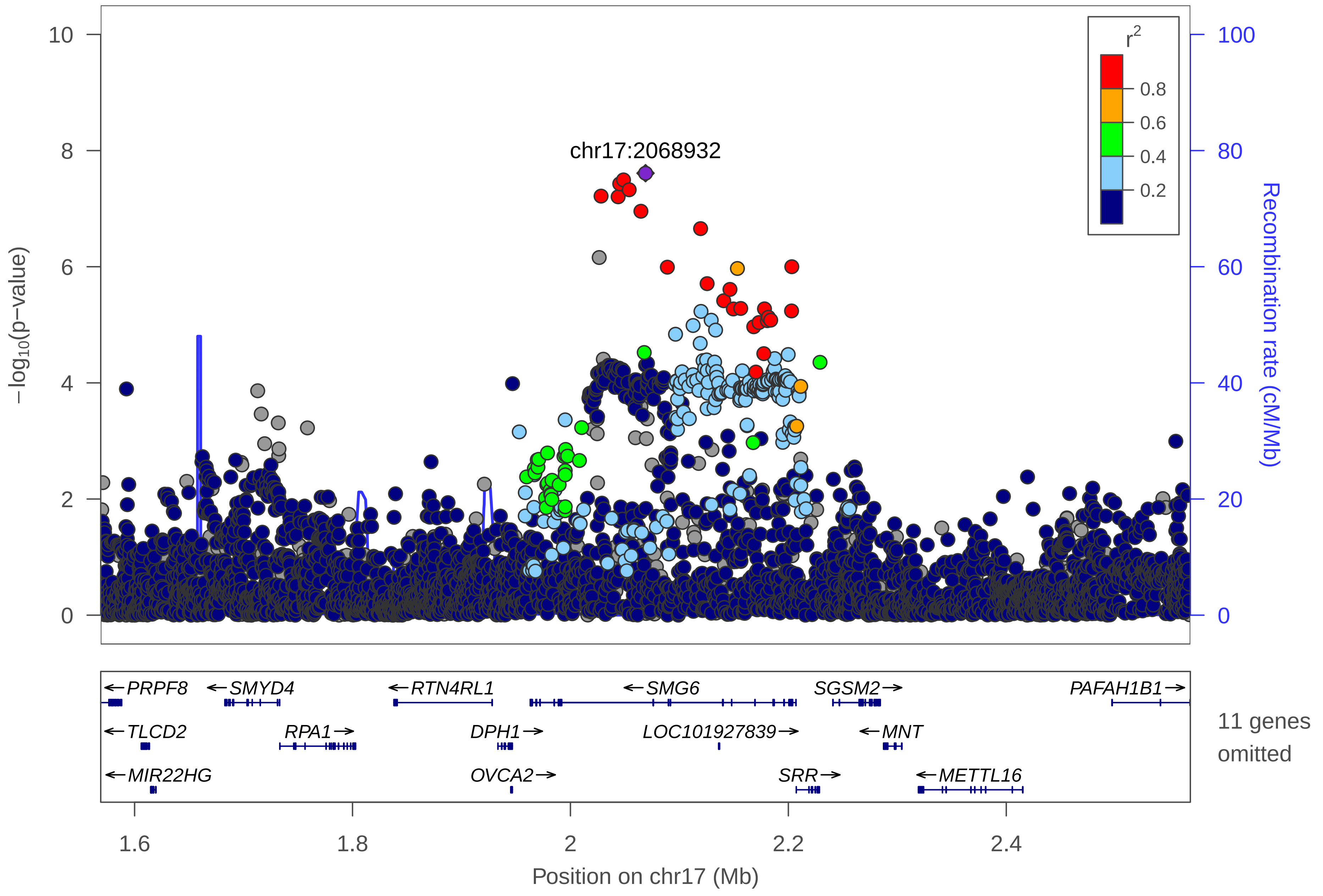

chr17:2068932
r2
0.8
0.6
0.4
0.2
−log10(p−value)
0
2
4
6
8
10
Recombination rate (cM/Mb)
0
20
40
60
80
100
PRPF8
SMYD4
RTN4RL1
SMG6
SGSM2
PAFAH1B1
TLCD2
RPA1
DPH1
LOC101927839
MNT
MIR22HG
OVCA2
SRR
METTL16
11 genes omitted
1.6
1.8
2
2.2
2.4
Position on chr17 (Mb)

date:	Wed Aug 1 13:04:47 2018
build:	hg19
display range:	chr17:1568932−2568932 [1568932−2568932]
hilite range:	0 − 0 [0 − 0]
reference SNP:	chr17:2068932
number of SNPs plotted:	4384
min P−value:	2.45E−8 [chr17:2068932]
max P−value:	9.99E−1 [chr17:1747450]
omitted Genes:	MIR22, WDR81, SERPINF2
omitted Genes:	SERPINF1, MIR132, MIR212
omitted Genes:	HIC1, TSR1, SNORD91B
omitted Genes:	SNORD91A, LOC284009

Make more plots at http://csg.sph.umich.edu/locuszoom/
