# Supplementary material for: Bone mineral density loci specific to the skull portray potential pleiotropic effects on craniosynostosis
Source: Commun Biol. 2023 Jul 4;6:691. doi: 10.1038/s42003-023-04869-0 (PMC10319806; doi:10.1038/s42003-023-04869-0)
Supplement: Supplementary file 6 — Supplementary Data 3 [file 42003_2023_4869_MOESM6_ESM.zip › loci/chr17_337381-1337381.pdf]

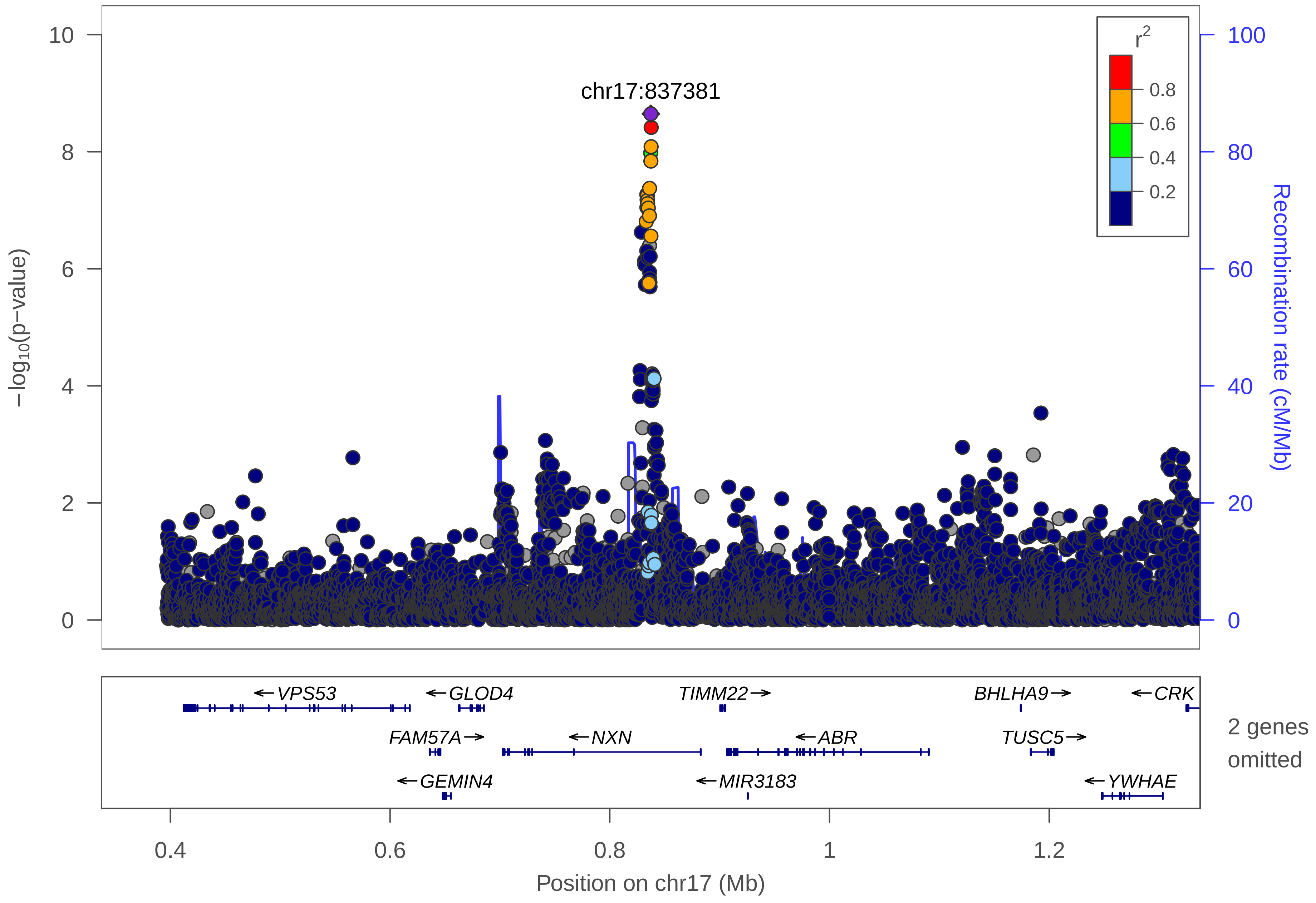
chr17:837381
r2
0.8
0.6
0.4
0.2
−log10(p−value)
Recombination rate (cM/Mb)
0
2
4
6
8
10
0
20
40
60
80
100
VPS53
GLOD4
TIMM22
BHLHA9
CRK
FAM57A
NXN
ABR
TUSC5
GEMIN4
MIR3183
YWHAE
2 genes omitted
0.4
0.6
0.8
1
1.2
Position on chr17 (Mb)

date:	Wed Aug 1 13:04:10 2018
build:	hg19
display range:	chr17:337381−1337381 [337381−1337381]
hilite range:	0 − 0 [0 − 0]
reference SNP:	chr17:837381
number of SNPs plotted:	6314
min P−value:	2.25E−9 [chr17:837381]
max P−value:	10E−1 [chr17:1100963]
omitted Genes:	DBIL5P, RNMTL1

Make more plots at http://csg.sph.umich.edu/locuszoom/
