# Supplementary material for: Bone mineral density loci specific to the skull portray potential pleiotropic effects on craniosynostosis
Source: Commun Biol. 2023 Jul 4;6:691. doi: 10.1038/s42003-023-04869-0 (PMC10319806; doi:10.1038/s42003-023-04869-0)
Supplement: Supplementary file 6 — Supplementary Data 3 [file 42003_2023_4869_MOESM6_ESM.zip › loci/chr17_41362277-42362277.pdf]

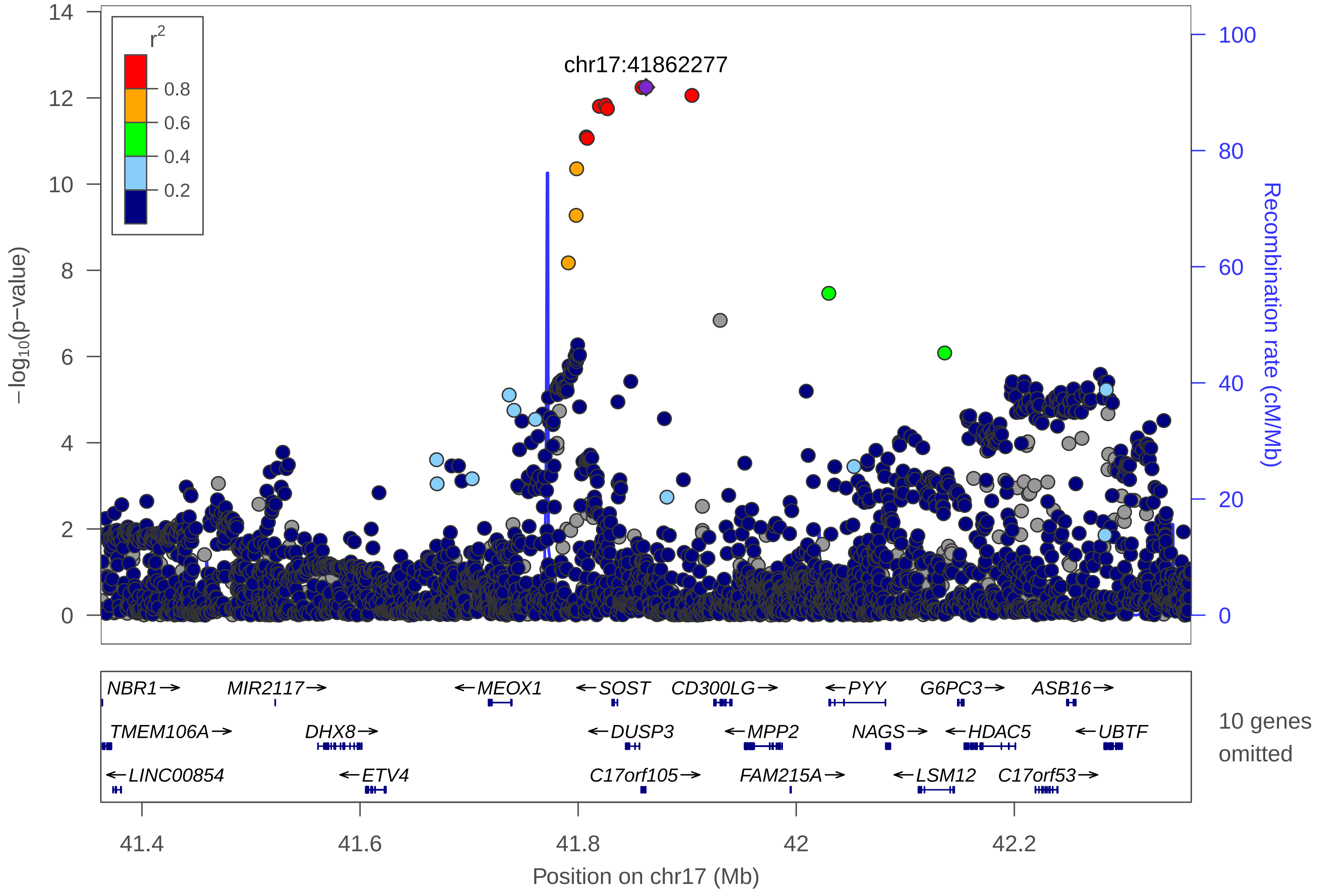

r2
0.8
0.6
0.4
0.2
chr17:41862277
−log10(p−value)
Recombination rate (cM/Mb)
14
12
10
8
6
4
2
0
100
80
60
40
20
0
NBR1→
MIR2117→
←MEOX1
←SOST
CD300LG→
←PYY
G6PC3→
ASB16→
TMEM106A→
DHX8→
←DUSP3
←MPP2
NAGS→
←HDAC5
←UBTF
←LINC00854
←ETV4
C17orf105→
FAM215A→
←LSM12
C17orf53→
10 genes omitted
41.4
41.6
41.8
42
42.2
Position on chr17 (Mb)

date:	Wed Aug 1 13:05:25 2018
build:	hg19
display range:	chr17:41362277−42362277 [41362277−42362277]
hilite range:	0 − 0 [0 − 0]
reference SNP:	chr17:41862277
number of SNPs plotted:	3387
min P−value:	5.66E−13 [chr17:41862277]
max P−value:	10E−1 [chr17:41645063]
omitted Genes:	LINC00910, ARL4D, MPP3
omitted Genes:	PPY, TMEM101, ASB16−AS1
omitted Genes:	TMUB2, ATXN7L3, MIR6782
omitted Genes:	SLC4A1

Make more plots at http://csg.sph.umich.edu/locuszoom/
