# Supplementary material for: Bone mineral density loci specific to the skull portray potential pleiotropic effects on craniosynostosis
Source: Commun Biol. 2023 Jul 4;6:691. doi: 10.1038/s42003-023-04869-0 (PMC10319806; doi:10.1038/s42003-023-04869-0)
Supplement: Supplementary file 6 — Supplementary Data 3 [file 42003_2023_4869_MOESM6_ESM.zip › loci/chr17_63150304-64150304.pdf]

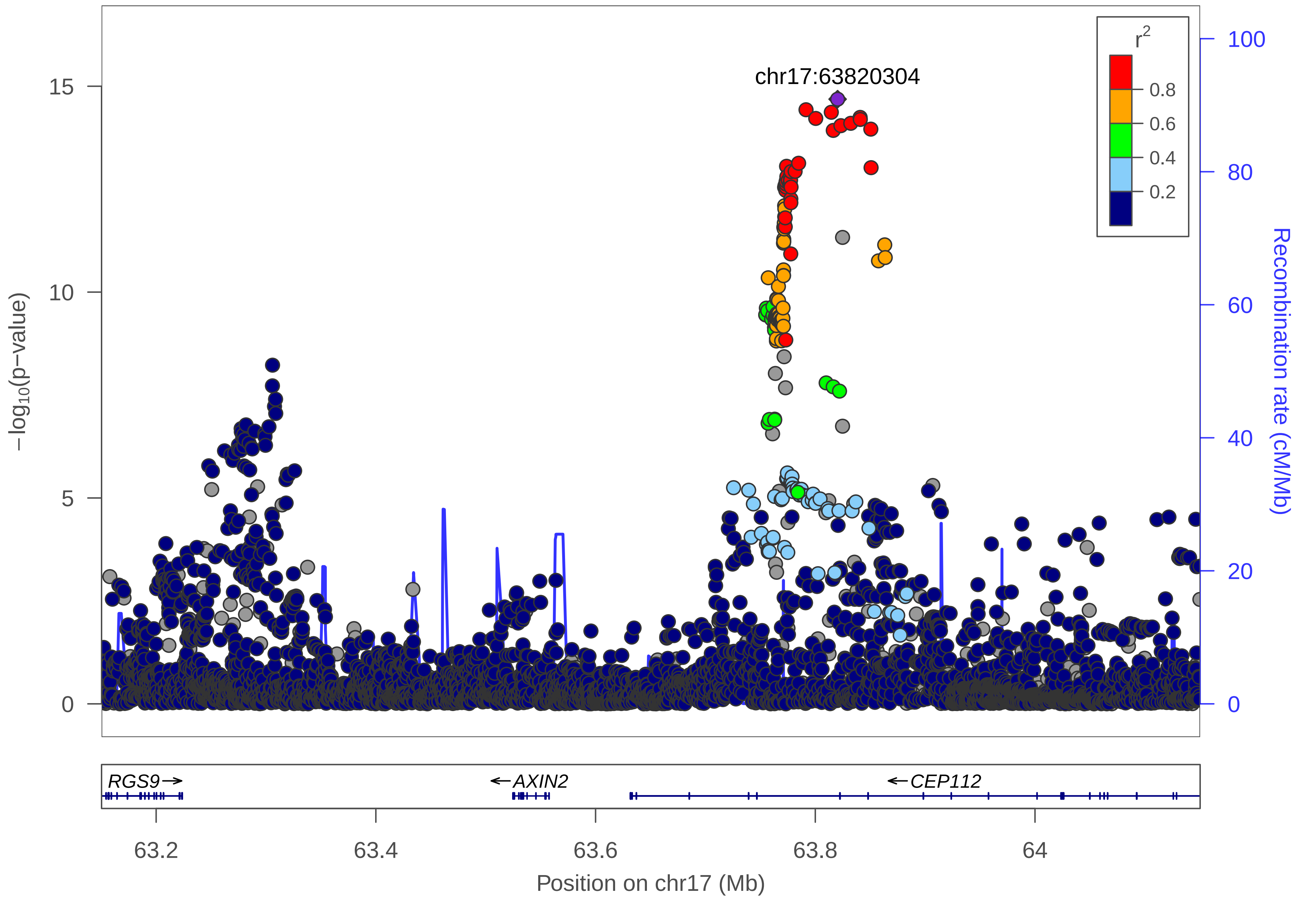

chr17:63820304
r2
0.8
0.6
0.4
0.2
15
10
5
0
−log10(p−value)
100
80
60
40
20
0
Recombination rate (cM/Mb)
RGS9→
←AXIN2
←CEP112
63.2
63.4
63.6
63.8
64
Position on chr17 (Mb)

date:	Wed Aug 1 15:32:33 2018
build:	hg19
display range:	chr17:63150304−64150304 [63150304−64150304]
hilite range:	0 − 0 [0 − 0]
reference SNP:	chr17:63820304
number of SNPs plotted:	4886
min P−value:	2.06E−15 [chr17:63820304]
max P−value:	1E0 [chr17:63610163]

Make more plots at http://csg.sph.umich.edu/locuszoom/
