# Supplementary material for: Bone mineral density loci specific to the skull portray potential pleiotropic effects on craniosynostosis
Source: Commun Biol. 2023 Jul 4;6:691. doi: 10.1038/s42003-023-04869-0 (PMC10319806; doi:10.1038/s42003-023-04869-0)
Supplement: Supplementary file 6 — Supplementary Data 3 [file 42003_2023_4869_MOESM6_ESM.zip › loci/chr17_65953305-66953305.pdf]

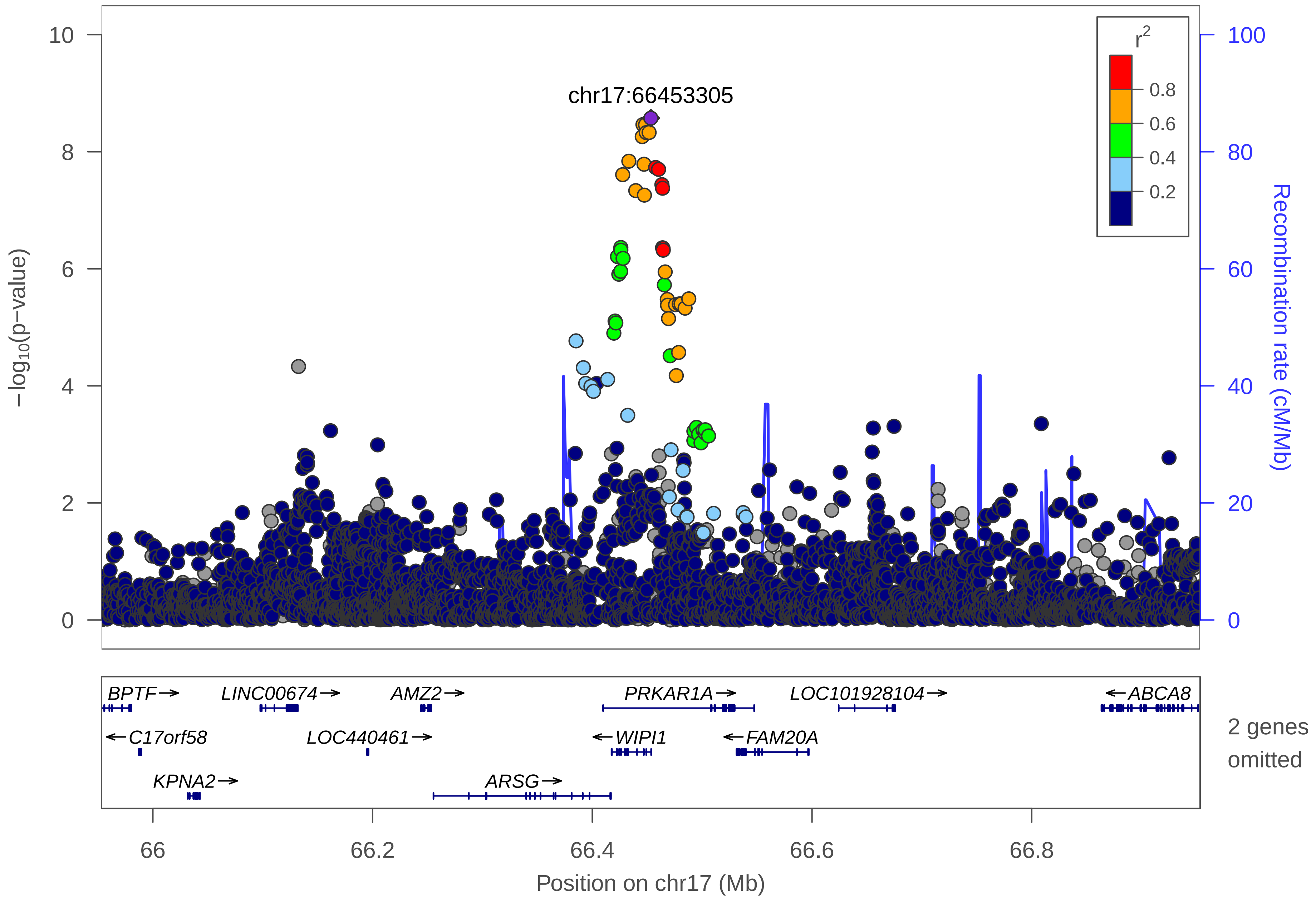

chr17:66453305
r2
0.8
0.6
0.4
0.2
−log10(p−value)
0
2
4
6
8
10
Recombination rate (cM/Mb)
0
20
40
60
80
100
BPTF→
LINC00674→
AMZ2→
PRKAR1A→
LOC101928104→
←ABCA8
←C17orf58
LOC440461→
←WIPI1
←FAM20A
KPNA2→
ARSG→
2 genes omitted
66
66.2
66.4
66.6
66.8
Position on chr17 (Mb)

date:	Wed Aug 1 13:07:17 2018
build:	hg19
display range:	chr17:65953305−66953305 [65953305−66953305]
hilite range:	0 − 0 [0 − 0]
reference SNP:	chr17:66453305
number of SNPs plotted:	4459
min P−value:	2.66E−9 [chr17:66453305]
max P−value:	10E−1 [chr17:66330485]
omitted Genes:	SLC16A6, MIR635

Make more plots at http://csg.sph.umich.edu/locuszoom/
