# Supplementary material for: Bone mineral density loci specific to the skull portray potential pleiotropic effects on craniosynostosis
Source: Commun Biol. 2023 Jul 4;6:691. doi: 10.1038/s42003-023-04869-0 (PMC10319806; doi:10.1038/s42003-023-04869-0)
Supplement: Supplementary file 6 — Supplementary Data 3 [file 42003_2023_4869_MOESM6_ESM.zip › loci/chr17_67560451-68560451.pdf]

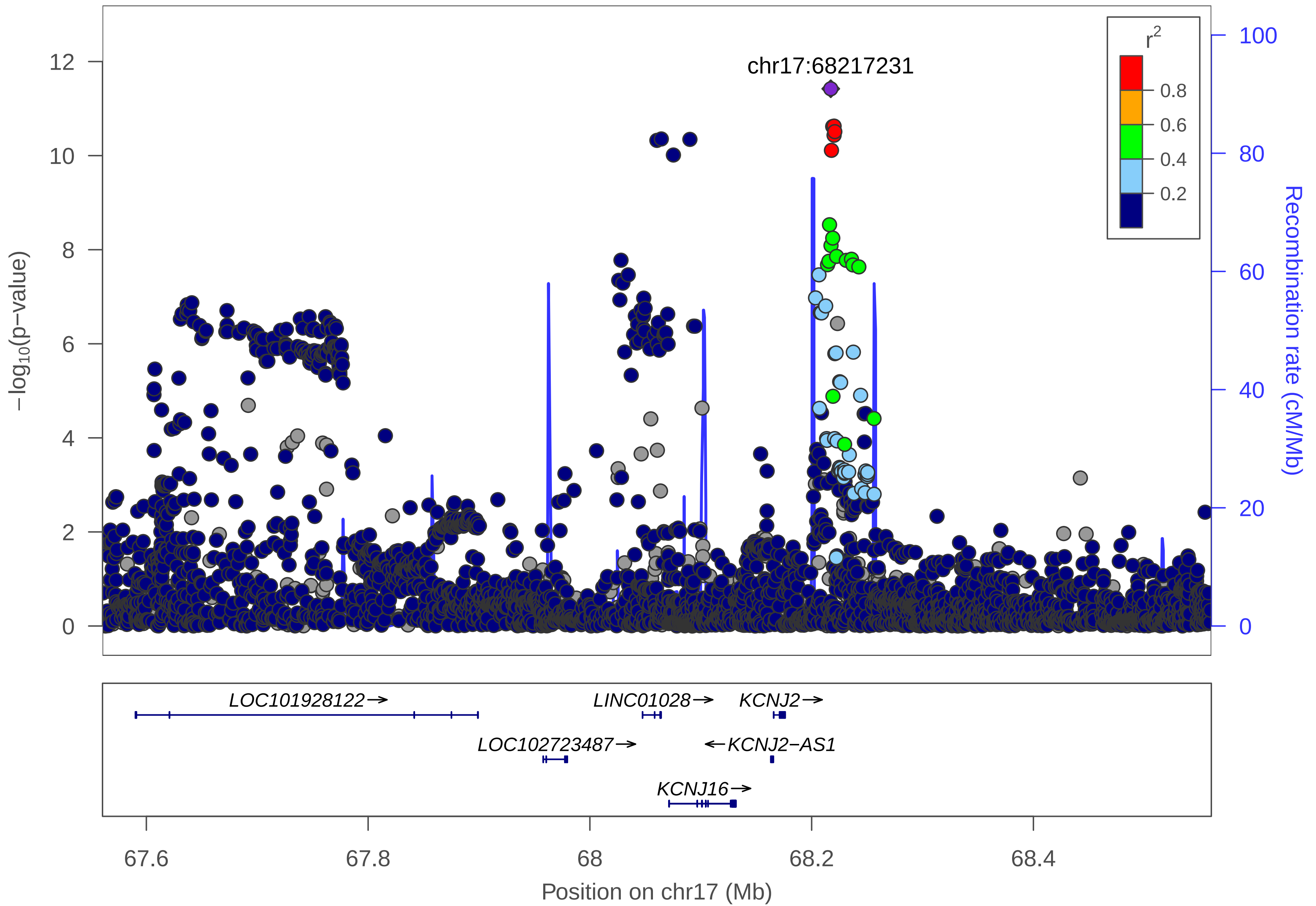

chr17:68217231
r2
0.8
0.6
0.4
0.2
−log10(p−value)
Recombination rate (cM/Mb)
0
2
4
6
8
10
12
0
20
40
60
80
100
LOC101928122→
LINC01028→
KCNJ2→
LOC102723487→
←KCNJ2−AS1
KCNJ16→
67.6
67.8
68
68.2
68.4
Position on chr17 (Mb)

date:	Wed Aug 1 13:08:30 2018
build:	hg19
display range:	chr17:67560451−68560451 [67560451−68560451]
hilite range:	0 − 0 [0 − 0]
reference SNP:	chr17:68217231
number of SNPs plotted:	3717
min P−value:	3.79E−12 [chr17:68217231]
max P−value:	9.99E−1 [chr17:68303074]

Make more plots at http://csg.sph.umich.edu/locuszoom/
