# Supplementary material for: Bone mineral density loci specific to the skull portray potential pleiotropic effects on craniosynostosis
Source: Commun Biol. 2023 Jul 4;6:691. doi: 10.1038/s42003-023-04869-0 (PMC10319806; doi:10.1038/s42003-023-04869-0)
Supplement: Supplementary file 6 — Supplementary Data 3 [file 42003_2023_4869_MOESM6_ESM.zip › loci/chr18_59559716-60559716.pdf]

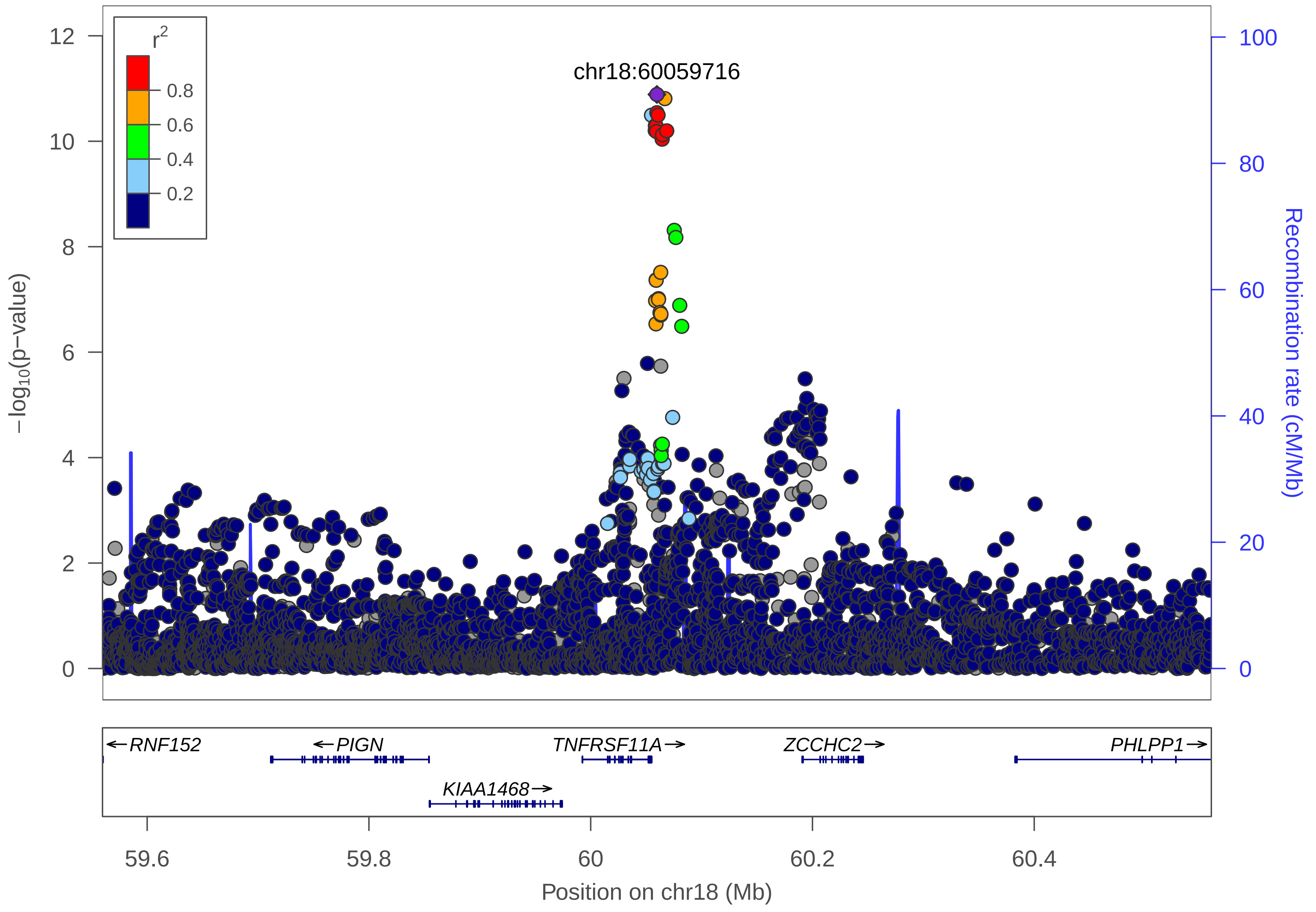

r2
0.8
0.6
0.4
0.2
chr18:60059716
12
10
8
6
4
2
0
−log10(p−value)
100
80
60
40
20
0
Recombination rate (cM/Mb)
←RNF152
←PIGN
TNFRSF11A→
ZCCHC2→
PHLPP1→
KIAA1468→
59.6
59.8
60
60.2
60.4
Position on chr18 (Mb)

date:	Wed Aug 1 13:09:44 2018
build:	hg19
display range:	chr18:59559716−60559716 [59559716−60559716]
hilite range:	0 − 0 [0 − 0]
reference SNP:	chr18:60059716
number of SNPs plotted:	4494
min P−value:	1.3E−11 [chr18:60059716]
max P−value:	10E−1 [chr18:59699902]

Make more plots at http://csg.sph.umich.edu/locuszoom/
