# Supplementary material for: Bone mineral density loci specific to the skull portray potential pleiotropic effects on craniosynostosis
Source: Commun Biol. 2023 Jul 4;6:691. doi: 10.1038/s42003-023-04869-0 (PMC10319806; doi:10.1038/s42003-023-04869-0)
Supplement: Supplementary file 6 — Supplementary Data 3 [file 42003_2023_4869_MOESM6_ESM.zip › loci/chr1_109975552-110975552.pdf]

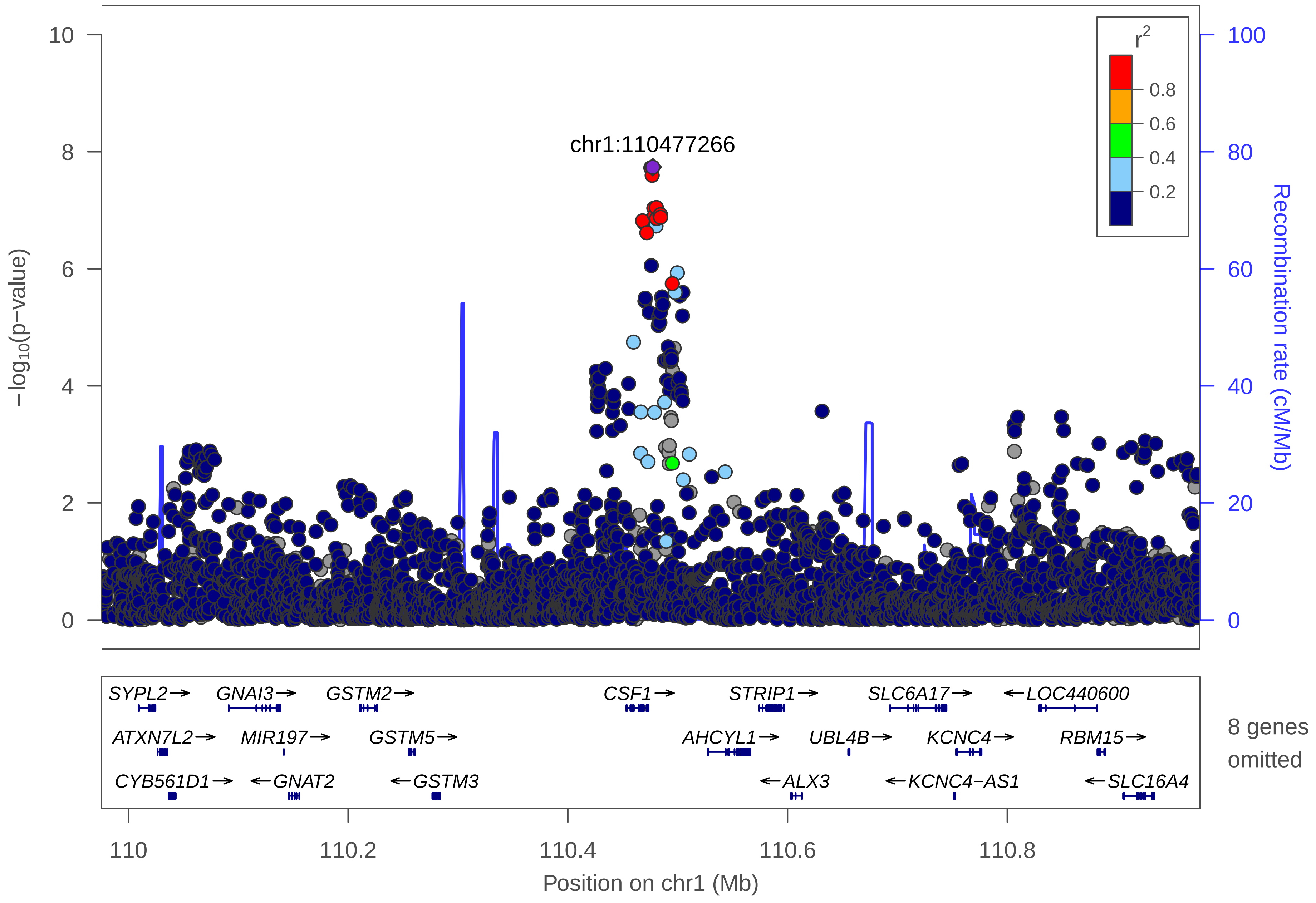

chr1:110477266
r2
0.8
0.6
0.4
0.2
10
8
6
4
2
0
100
80
60
40
20
0
−log10(p−value)
Recombination rate (cM/Mb)
SYPL2→
GNAI3→
GSTM2→
CSF1→
STRIP1→
SLC6A17→
←LOC440600
ATXN7L2→
MIR197→
GSTM5→
AHCYL1→
UBL4B→
KCNC4→
RBM15→
CYB561D1→
←GNAT2
←GSTM3
←ALX3
←KCNC4−AS1
←SLC16A4
8 genes omitted
110
110.2
110.4
110.6
110.8
Position on chr1 (Mb)

| | |
|---|---|
| date: | Wed Aug 1 12:28:29 2018 |
| build: | hg19 |
| display range: | chr1:109975552−110975552 [109975552−110975552] |
| hilite range: | 0 − 0 [ 0 − 0 ] |
| reference SNP: | chr1:110477266 |
| number of SNPs plotted: | 3783 |
| min P−value: | 1.83E−8 [chr1:110477266] |
| max P−value: | 10E−1 [chr1:110272599] |
| omitted Genes: | AMIGO1, GPR61, AMPD2 |
| omitted Genes: | GSTM4, GSTM1, EPS8L3 |
| omitted Genes: | LAMTOR5, LAMTOR5−AS1 |

Make more plots at http://csg.sph.umich.edu/locuszoom/
