# Supplementary material for: Bone mineral density loci specific to the skull portray potential pleiotropic effects on craniosynostosis
Source: Commun Biol. 2023 Jul 4;6:691. doi: 10.1038/s42003-023-04869-0 (PMC10319806; doi:10.1038/s42003-023-04869-0)
Supplement: Supplementary file 6 — Supplementary Data 3 [file 42003_2023_4869_MOESM6_ESM.zip › loci/chr1_112647607-113647607.pdf]

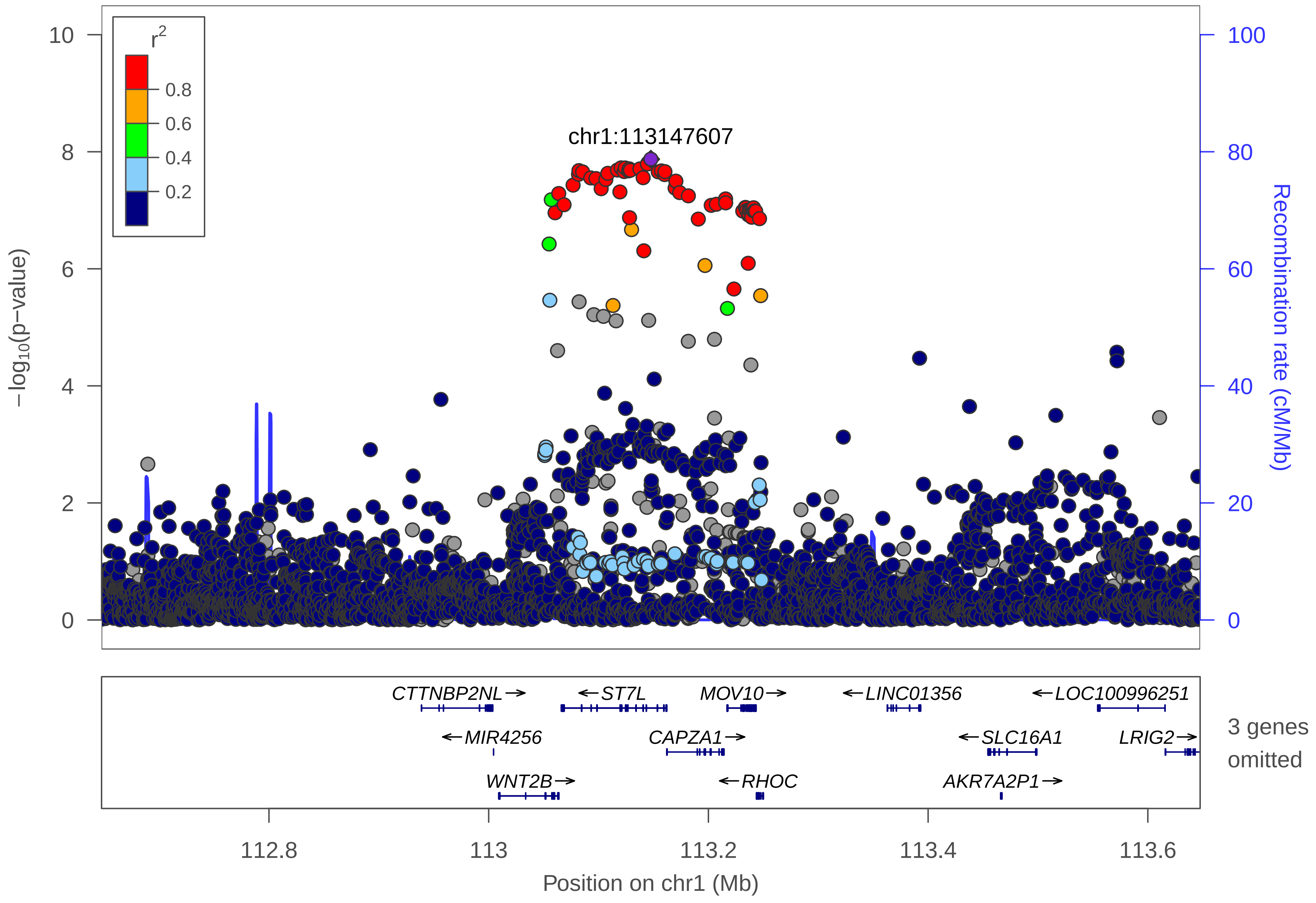

r²
0.8
0.6
0.4
0.2
chr1:113147607
10
8
6
4
2
0
−log10(p−value)
100
80
60
40
20
0
Recombination rate (cM/Mb)
CTTNBP2NL→
←ST7L
MOV10→
←LINC01356
←LOC100996251
←MIR4256
CAPZA1→
←SLC16A1
LRIG2→
WNT2B→
←RHOC
AKR7A2P1→
3 genes omitted
112.8
113
113.2
113.4
113.6
Position on chr1 (Mb)

date:	Wed Aug 1 12:29:04 2018
build:	hg19
display range:	chr1:112647607−113647607 [112647607−113647607]
hilite range:	0 − 0 [0 − 0]
reference SNP:	chr1:113147607
number of SNPs plotted:	3624
min P−value:	1.34E−8 [chr1:113147607]
max P−value:	10E−1 [chr1:112926937]
omitted Genes:	PPM1J, FAM19A3, SLC16A1−AS1

Make more plots at http://csg.sph.umich.edu/locuszoom/
