# Supplementary material for: Bone mineral density loci specific to the skull portray potential pleiotropic effects on craniosynostosis
Source: Commun Biol. 2023 Jul 4;6:691. doi: 10.1038/s42003-023-04869-0 (PMC10319806; doi:10.1038/s42003-023-04869-0)
Supplement: Supplementary file 6 — Supplementary Data 3 [file 42003_2023_4869_MOESM6_ESM.zip › loci/chr1_163375519-164375519.pdf]

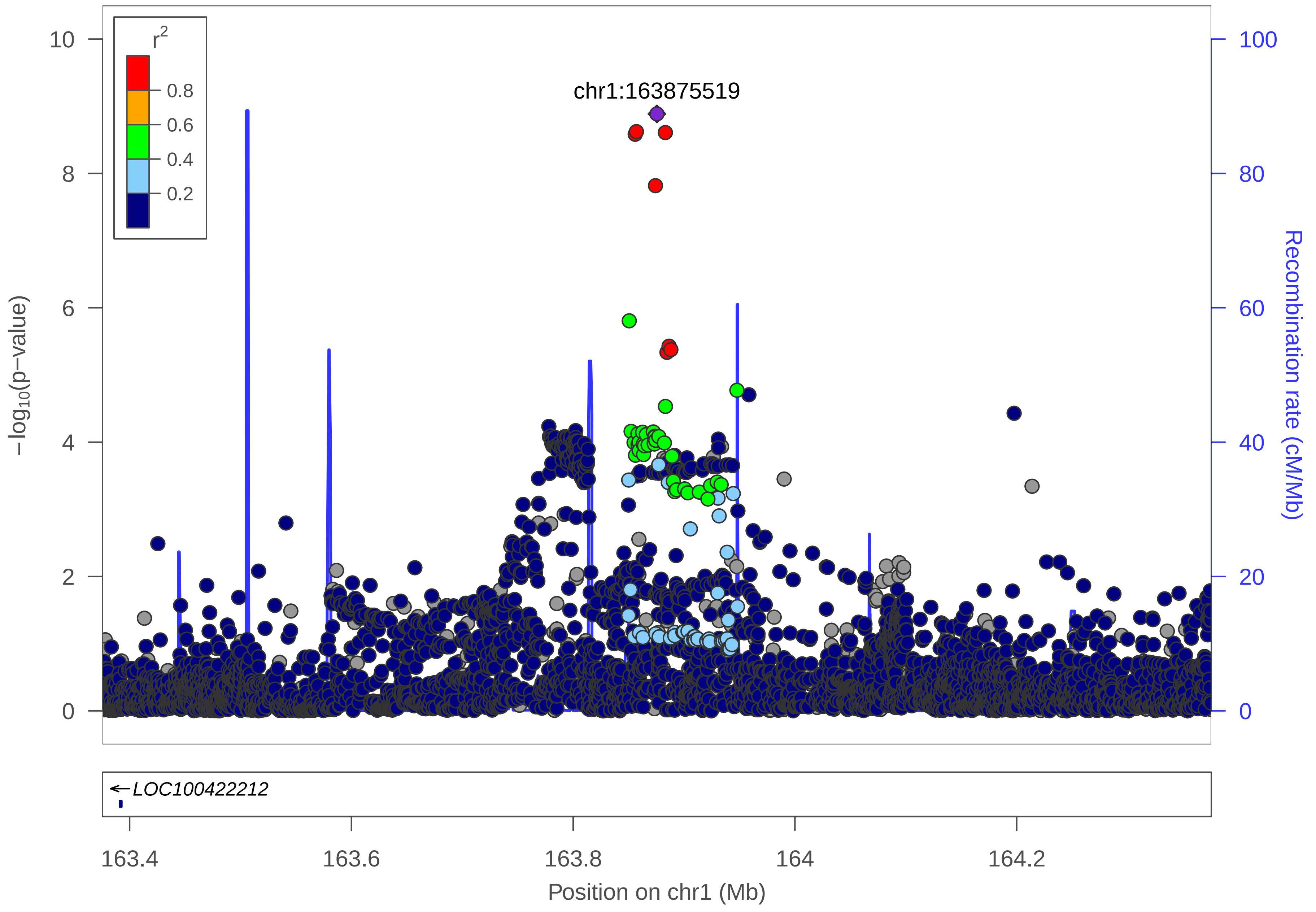

r2
0.8
0.6
0.4
0.2
chr1:163875519
10
8
6
4
2
0
−log10(p−value)
100
80
60
40
20
0
Recombination rate (cM/Mb)
←LOC100422212
163.4
163.6
163.8
164
164.2
Position on chr1 (Mb)

date:	Wed Aug 1 12:29:38 2018
build:	hg19
display range:	chr1:163375519−164375519 [163375519−164375519]
hilite range:	0 − 0 [0 − 0]
reference SNP:	chr1:163875519
number of SNPs plotted:	4378
min P−value:	1.3E−9 [chr1:163875519]
max P−value:	9.99E−1 [chr1:163556749]

Make more plots at http://csg.sph.umich.edu/locuszoom/
