# Supplementary material for: Bone mineral density loci specific to the skull portray potential pleiotropic effects on craniosynostosis
Source: Commun Biol. 2023 Jul 4;6:691. doi: 10.1038/s42003-023-04869-0 (PMC10319806; doi:10.1038/s42003-023-04869-0)
Supplement: Supplementary file 6 — Supplementary Data 3 [file 42003_2023_4869_MOESM6_ESM.zip › loci/chr1_171622809-172622809.pdf]

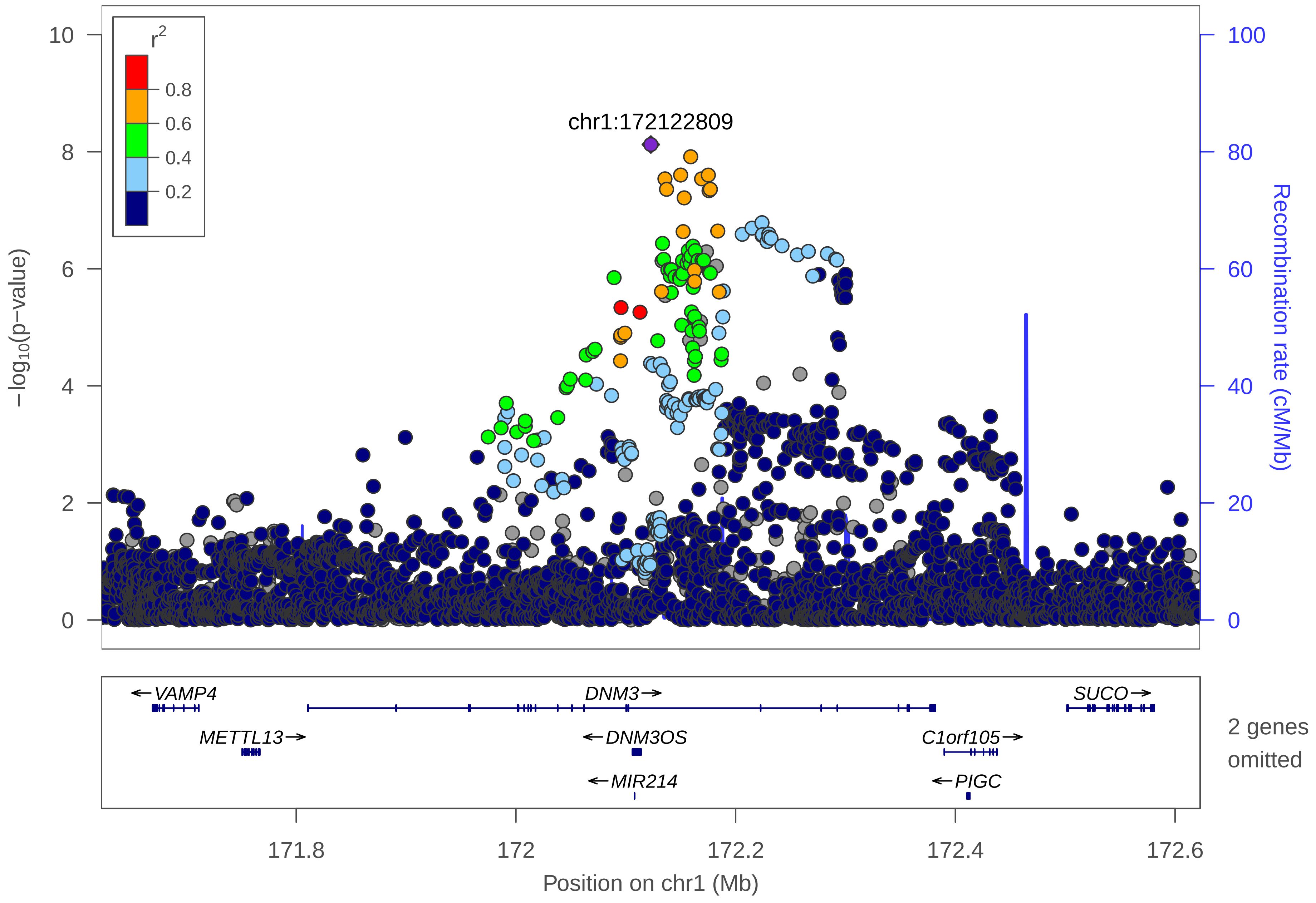
chr1:172122809
r2
0.8
0.6
0.4
0.2
−log10(p−value)
Recombination rate (cM/Mb)
10
8
6
4
2
0
100
80
60
40
20
0
VAMP4
DNM3
SUCO
METTL13
DNM3OS
C1orf105
MIR214
PIGC
2 genes omitted
171.8
172
172.2
172.4
172.6
Position on chr1 (Mb)

date:	Wed Aug 1 12:30:12 2018
build:	hg19
display range:	chr1:171622809−172622809 [171622809−172622809]
hilite range:	0 − 0 [0 − 0]
reference SNP:	chr1:172122809
number of SNPs plotted:	3799
min P−value:	7.52E−9 [chr1:172122809]
max P−value:	9.99E−1 [chr1:171693357]
omitted Genes:	MIR3120, MIR199A2

Make more plots at http://csg.sph.umich.edu/locuszoom/
