# Supplementary material for: Bone mineral density loci specific to the skull portray potential pleiotropic effects on craniosynostosis
Source: Commun Biol. 2023 Jul 4;6:691. doi: 10.1038/s42003-023-04869-0 (PMC10319806; doi:10.1038/s42003-023-04869-0)
Supplement: Supplementary file 6 — Supplementary Data 3 [file 42003_2023_4869_MOESM6_ESM.zip › loci/chr1_21951845-22951845.pdf]

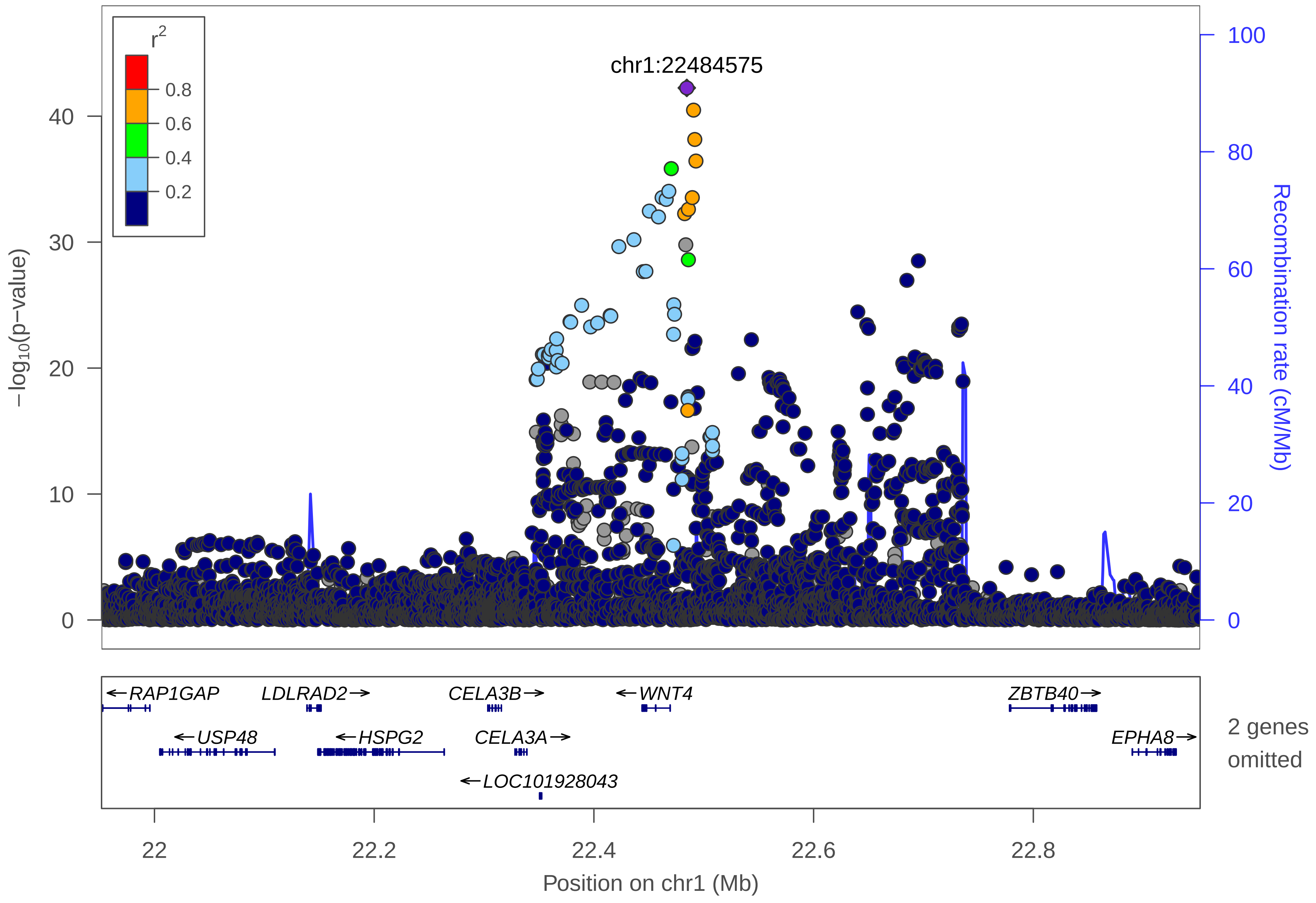

r2
0.8
0.6
0.4
0.2
chr1:22484575
−log10(p−value)
0
10
20
30
40
Recombination rate (cM/Mb)
0
20
40
60
80
100
RAP1GAP
LDLRAD2
CELA3B
WNT4
ZBTB40
USP48
HSPG2
CELA3A
EPHA8
LOC101928043
2 genes omitted
22
22.2
22.4
22.6
22.8
Position on chr1 (Mb)

date:	Wed Aug 1 12:25:35 2018
build:	hg19
display range:	chr1:21951845−22951845 [21951845−22951845]
hilite range:	0 − 0 [0 − 0]
reference SNP:	chr1:22484575
number of SNPs plotted:	5165
min P−value:	5.7E−43 [chr1:22484575]
max P−value:	10E−1 [chr1:22308498]
omitted Genes:	LINC00339, CDC42

Make more plots at http://csg.sph.umich.edu/locuszoom/
