# Supplementary material for: Bone mineral density loci specific to the skull portray potential pleiotropic effects on craniosynostosis
Source: Commun Biol. 2023 Jul 4;6:691. doi: 10.1038/s42003-023-04869-0 (PMC10319806; doi:10.1038/s42003-023-04869-0)
Supplement: Supplementary file 6 — Supplementary Data 3 [file 42003_2023_4869_MOESM6_ESM.zip › loci/chr1_219629357-220629357.pdf]

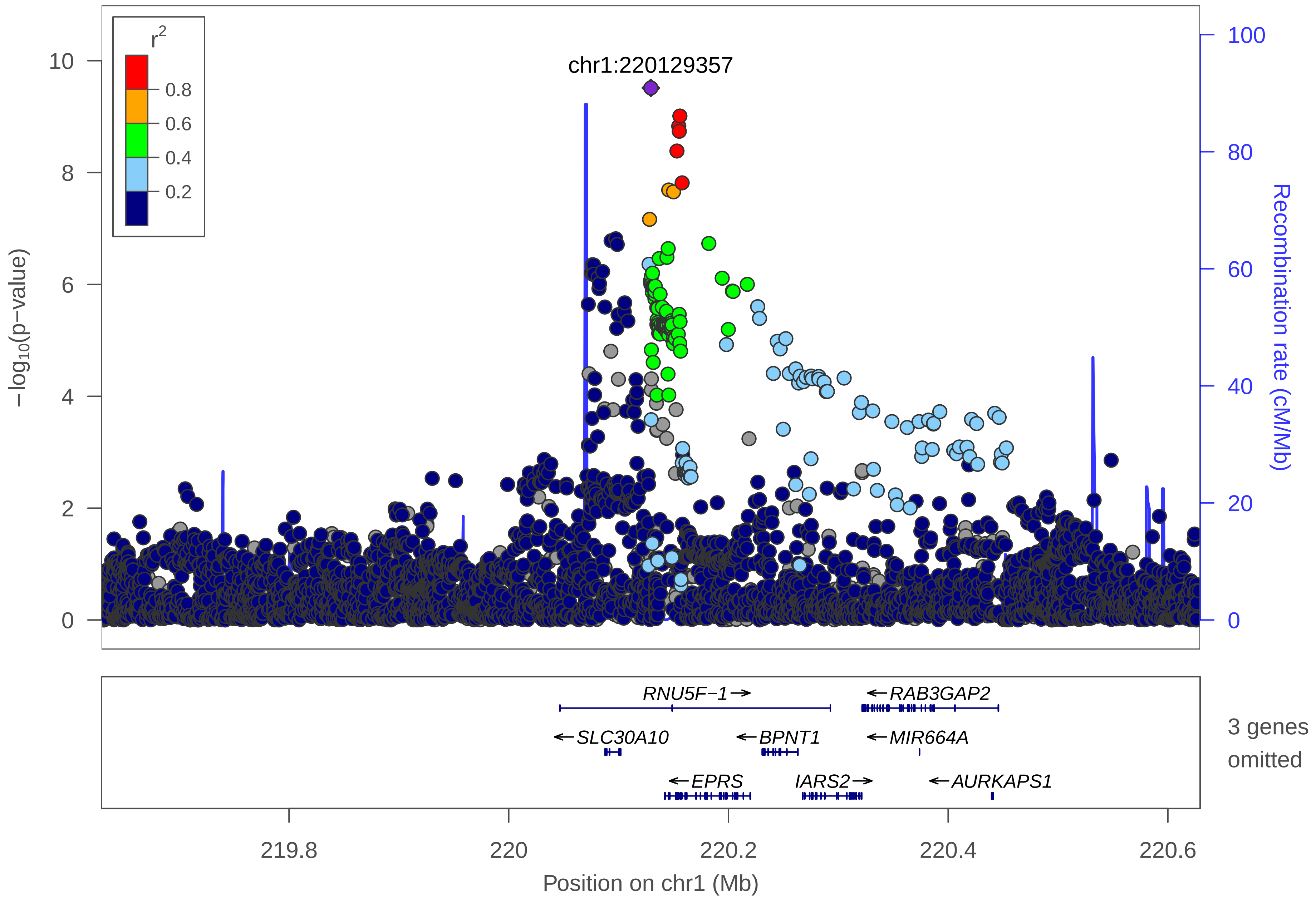

r2
0.8
0.6
0.4
0.2
chr1:220129357
−log10(p−value)
10
8
6
4
2
0
Recombination rate (cM/Mb)
100
80
60
40
20
0
RNU5F−1→
←RAB3GAP2
←SLC30A10
←BPNT1
←MIR664A
←EPRS
IARS2→
←AURKAPS1
3 genes omitted
219.8
220
220.2
220.4
220.6
Position on chr1 (Mb)

date:	Wed Aug 1 12:30:47 2018
build:	hg19
display range:	chr1:219629357−220629357 [219629357−220629357]
hilite range:	0 − 0 [0 − 0]
reference SNP:	chr1:220129357
number of SNPs plotted:	4217
min P−value:	3.06E−10 [chr1:220129357]
max P−value:	9.99E−1 [chr1:220494283]
omitted Genes:	MIR215, MIR194−1, SNORA36B

Make more plots at http://csg.sph.umich.edu/locuszoom/
