# Supplementary material for: Bone mineral density loci specific to the skull portray potential pleiotropic effects on craniosynostosis
Source: Commun Biol. 2023 Jul 4;6:691. doi: 10.1038/s42003-023-04869-0 (PMC10319806; doi:10.1038/s42003-023-04869-0)
Supplement: Supplementary file 6 — Supplementary Data 3 [file 42003_2023_4869_MOESM6_ESM.zip › loci/chr1_68134642-69134642.pdf]

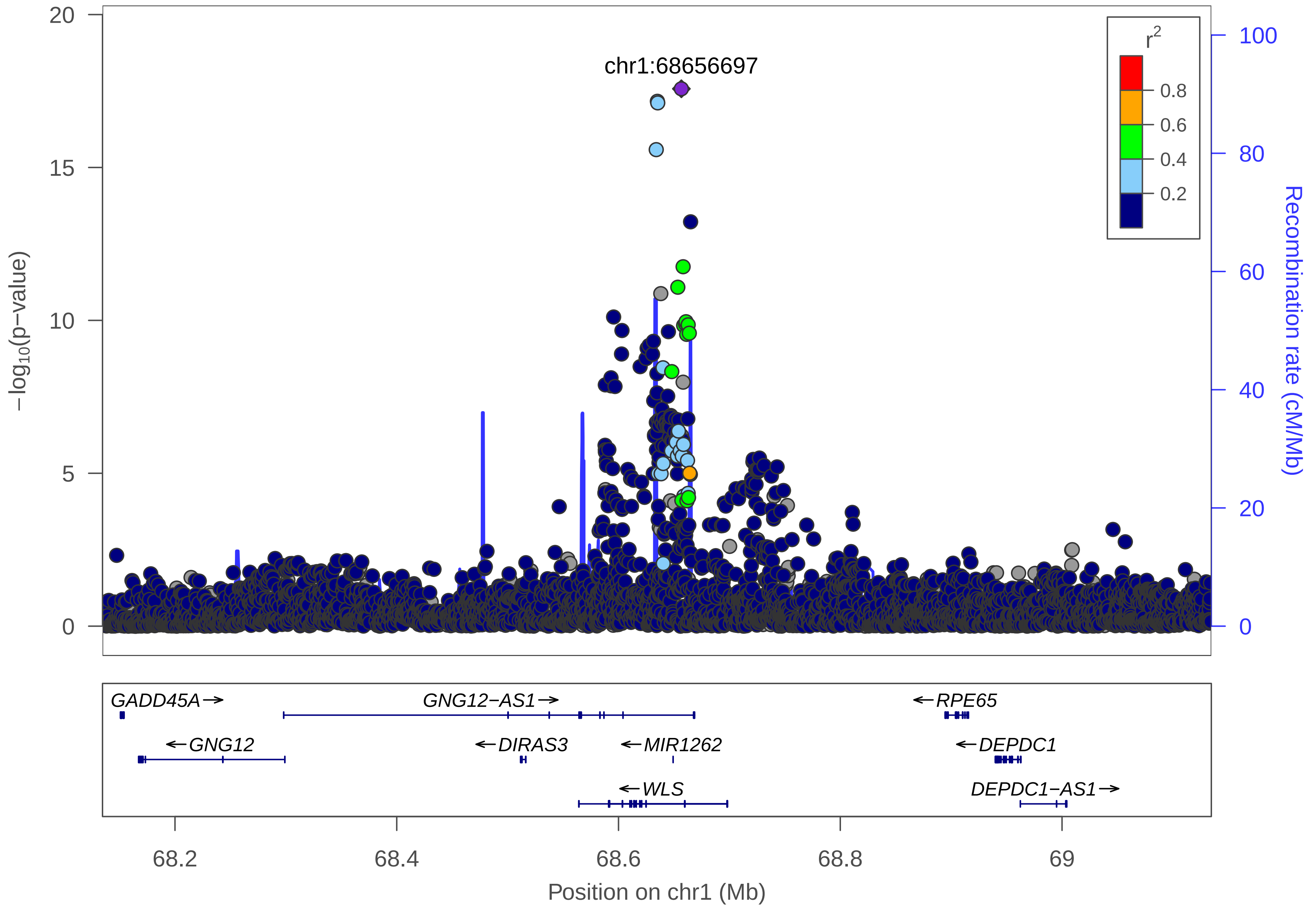

chr1:68656697
r2
0.8
0.6
0.4
0.2
−log10(p−value)
Recombination rate (cM/Mb)
0
5
10
15
20
0
20
40
60
80
100
GADD45A→
GNG12−AS1→
←RPE65
←GNG12
←DIRAS3
←MIR1262
←DEPDC1
←WLS
DEPDC1−AS1→
68.2
68.4
68.6
68.8
69
Position on chr1 (Mb)

date:	Wed Aug 1 12:27:20 2018
build:	hg19
display range:	chr1:68134642−69134642 [68134642−69134642]
hilite range:	0 − 0 [0 − 0]
reference SNP:	chr1:68656697
number of SNPs plotted:	4349
min P−value:	2.67E−18 [chr1:68656697]
max P−value:	10E−1 [chr1:68201556]

Make more plots at http://csg.sph.umich.edu/locuszoom/
