# Supplementary material for: Bone mineral density loci specific to the skull portray potential pleiotropic effects on craniosynostosis
Source: Commun Biol. 2023 Jul 4;6:691. doi: 10.1038/s42003-023-04869-0 (PMC10319806; doi:10.1038/s42003-023-04869-0)
Supplement: Supplementary file 6 — Supplementary Data 3 [file 42003_2023_4869_MOESM6_ESM.zip › loci/chr20_10140306-11140306.pdf]

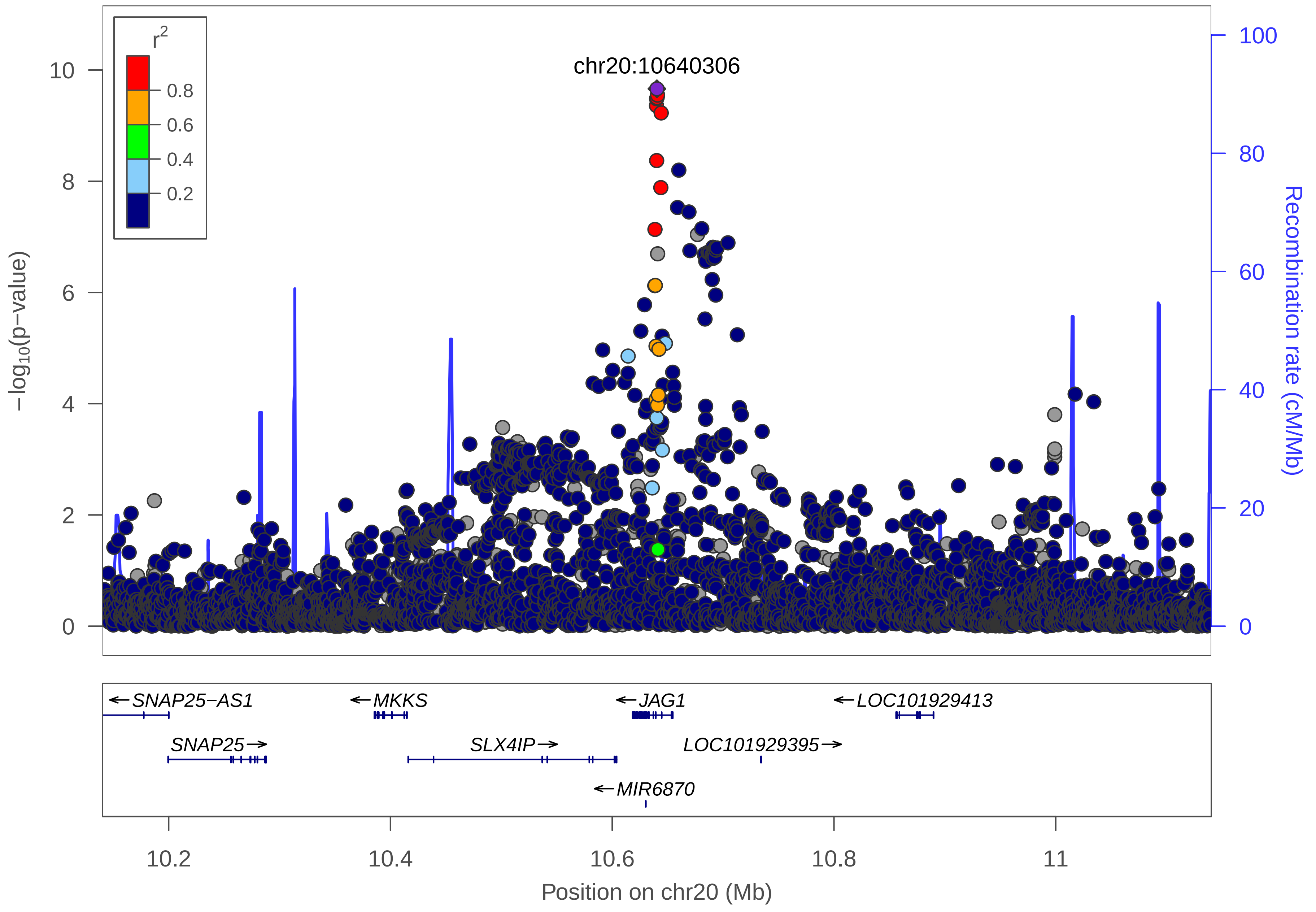

chr20:10640306
r2
0.8
0.6
0.4
0.2
10
8
6
4
2
0
−log10(p−value)
100
80
60
40
20
0
Recombination rate (cM/Mb)
←SNAP25−AS1
←MKKS
←JAG1
←LOC101929413
SNAP25→
SLX4IP→
LOC101929395→
←MIR6870
10.2
10.4
10.6
10.8
11
Position on chr20 (Mb)

date:	Wed Aug 1 13:10:22 2018
build:	hg19
display range:	chr20:10140306−11140306 [10140306−11140306]
hilite range:	0 − 0 [0 − 0]
reference SNP:	chr20:10640306
number of SNPs plotted:	4491
min P−value:	2.17E−10 [chr20:10640306]
max P−value:	10E−1 [chr20:10750883]

Make more plots at http://csg.sph.umich.edu/locuszoom/
