# Supplementary material for: Bone mineral density loci specific to the skull portray potential pleiotropic effects on craniosynostosis
Source: Commun Biol. 2023 Jul 4;6:691. doi: 10.1038/s42003-023-04869-0 (PMC10319806; doi:10.1038/s42003-023-04869-0)
Supplement: Supplementary file 6 — Supplementary Data 3 [file 42003_2023_4869_MOESM6_ESM.zip › loci/chr20_38588536-39588536.pdf]

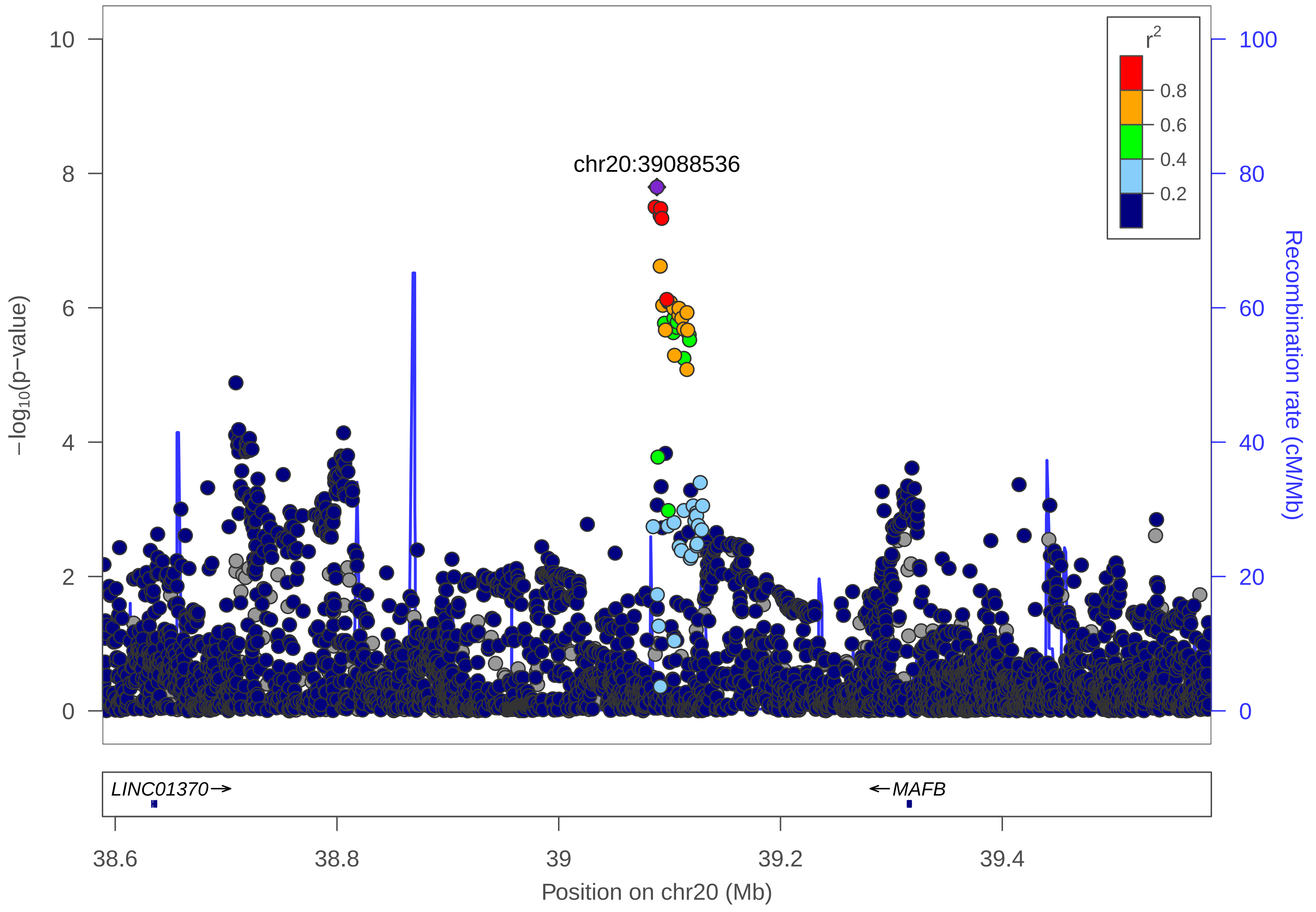

chr20:39088536
r²
0.8
0.6
0.4
0.2
−log10(p−value)
Recombination rate (cM/Mb)
0
2
4
6
8
10
0
20
40
60
80
100
LINC01370→
←MAFB
38.6
38.8
39
39.2
39.4
Position on chr20 (Mb)

date:	Wed Aug 1 13:10:59 2018
build:	hg19
display range:	chr20:38588536−39588536 [38588536−39588536]
hilite range:	0 − 0 [0 − 0]
reference SNP:	chr20:39088536
number of SNPs plotted:	4035
min P−value:	1.6E−8 [chr20:39088536]
max P−value:	10E−1 [chr20:38665715]

Make more plots at http://csg.sph.umich.edu/locuszoom/
