# Supplementary material for: Bone mineral density loci specific to the skull portray potential pleiotropic effects on craniosynostosis
Source: Commun Biol. 2023 Jul 4;6:691. doi: 10.1038/s42003-023-04869-0 (PMC10319806; doi:10.1038/s42003-023-04869-0)
Supplement: Supplementary file 6 — Supplementary Data 3 [file 42003_2023_4869_MOESM6_ESM.zip › loci/chr21_36470350-37470350.pdf]

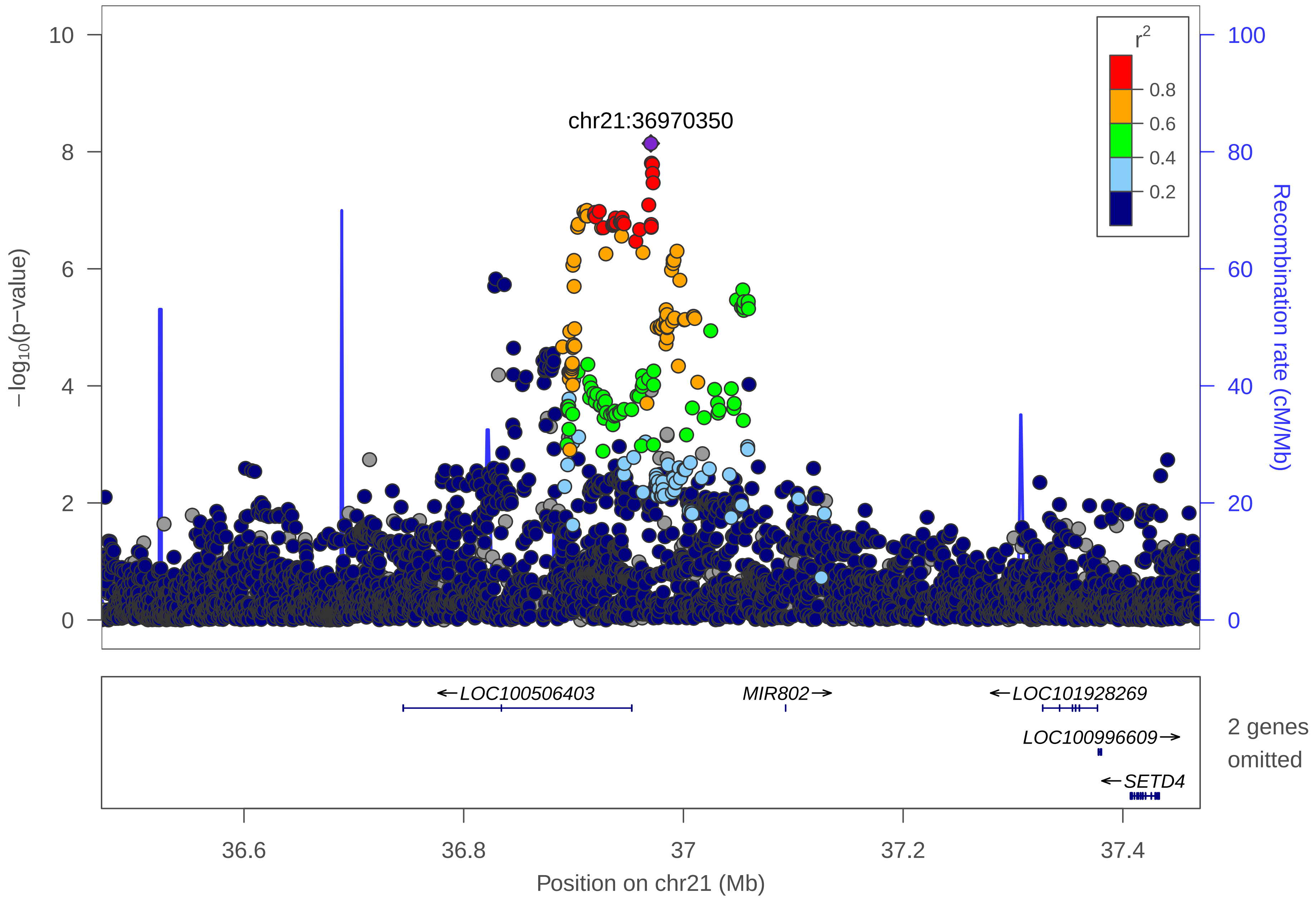

chr21:36970350
r²
0.8
0.6
0.4
0.2
−log10(p−value)
Recombination rate (cM/Mb)
10
8
6
4
2
0
100
80
60
40
20
0
←LOC100506403
MIR802→
←LOC101928269
LOC100996609→
←SETD4
2 genes omitted
36.6
36.8
37
37.2
37.4
Position on chr21 (Mb)

date:	Wed Aug 1 13:11:37 2018
build:	hg19
display range:	chr21:36470350−37470350 [36470350−37470350]
hilite range:	0 − 0 [0 − 0]
reference SNP:	chr21:36970350
number of SNPs plotted:	4675
min P−value:	7.21E−9 [chr21:36970350]
max P−value:	9.99E−1 [chr21:36678179]
omitted Genes:	LOC100133286, CBR1

Make more plots at http://csg.sph.umich.edu/locuszoom/
