# Supplementary material for: Bone mineral density loci specific to the skull portray potential pleiotropic effects on craniosynostosis
Source: Commun Biol. 2023 Jul 4;6:691. doi: 10.1038/s42003-023-04869-0 (PMC10319806; doi:10.1038/s42003-023-04869-0)
Supplement: Supplementary file 6 — Supplementary Data 3 [file 42003_2023_4869_MOESM6_ESM.zip › loci/chr2_118800000-119800000.pdf]

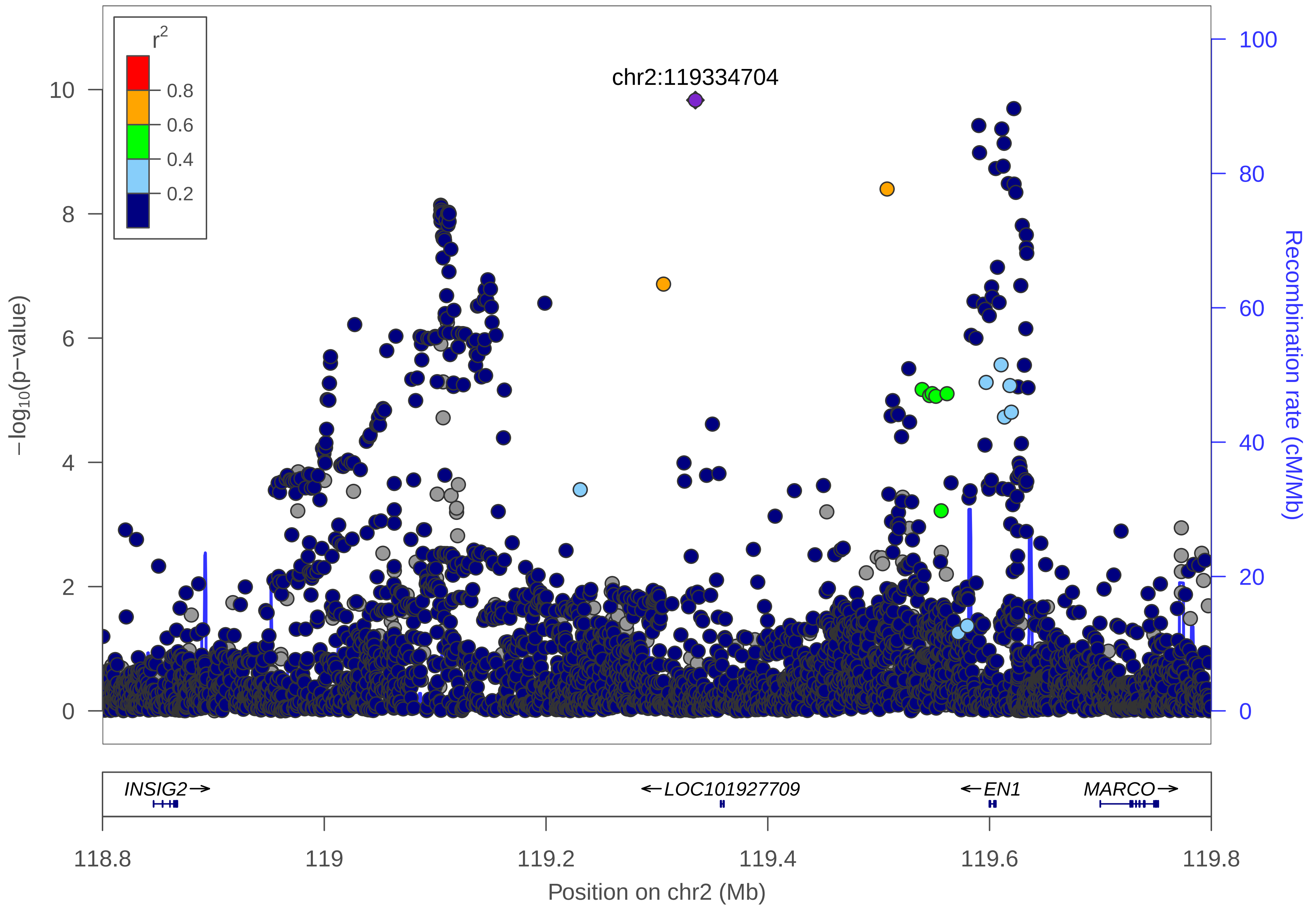

r2
0.8
0.6
0.4
0.2
chr2:119334704
−log10(p−value)
Recombination rate (cM/Mb)
0
2
4
6
8
10
0
20
40
60
80
100
INSIG2→
←LOC101927709
←EN1
MARCO→
118.8
119
119.2
119.4
119.6
119.8
Position on chr2 (Mb)

date:	Wed Aug 1 14:21:41 2018
build:	hg19
display range:	chr2:118800000−119800000 [118800000−119800000]
hilite range:	0 − 0 [0 − 0]
reference SNP:	chr2:119334704
number of SNPs plotted:	4088
min P−value:	1.48E−10 [chr2:119334704]
max P−value:	10E−1 [chr2:118960598]

Make more plots at http://csg.sph.umich.edu/locuszoom/
