# Supplementary material for: Bone mineral density loci specific to the skull portray potential pleiotropic effects on craniosynostosis
Source: Commun Biol. 2023 Jul 4;6:691. doi: 10.1038/s42003-023-04869-0 (PMC10319806; doi:10.1038/s42003-023-04869-0)
Supplement: Supplementary file 6 — Supplementary Data 3 [file 42003_2023_4869_MOESM6_ESM.zip › loci/chr2_40130678-41130678.pdf]

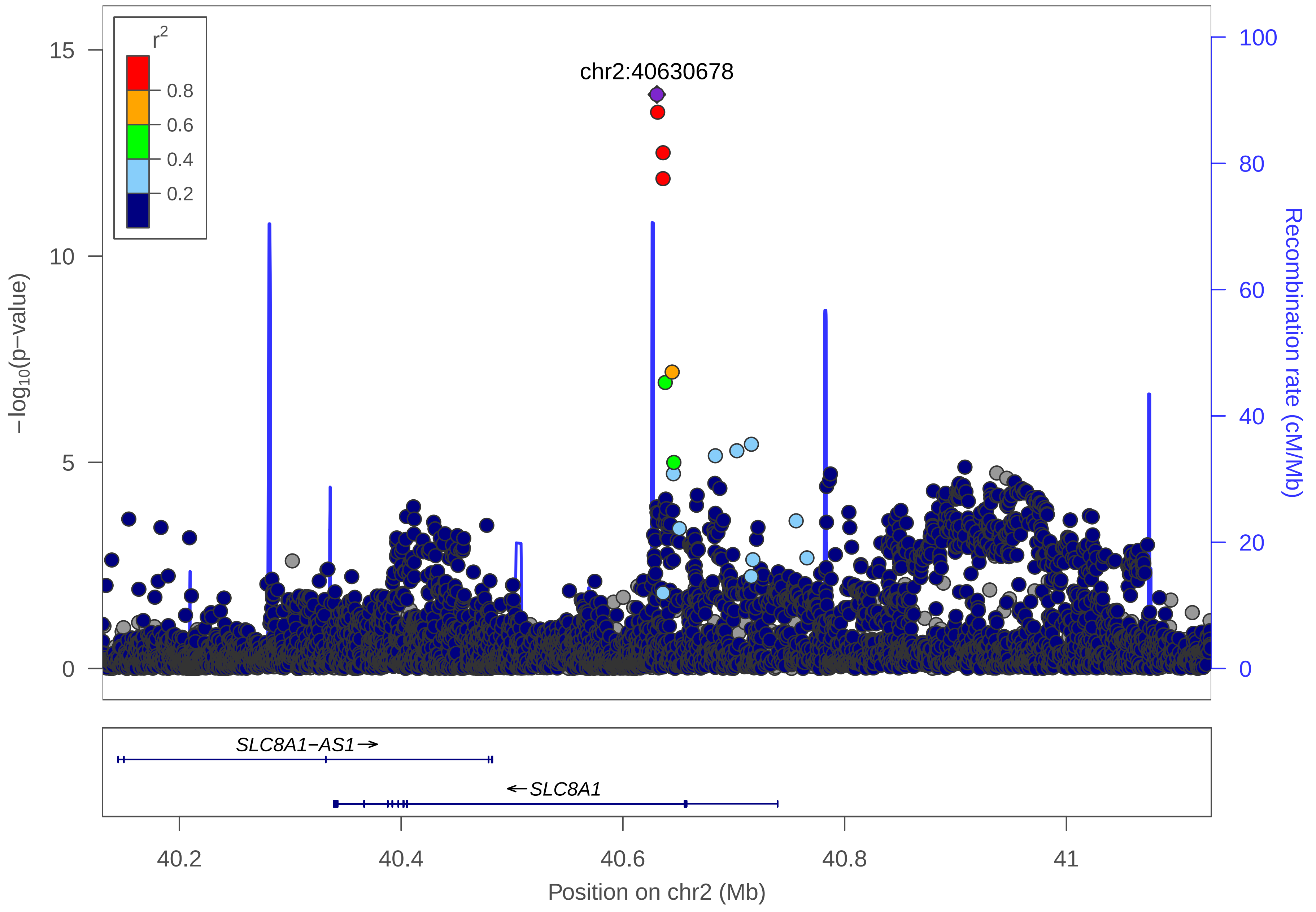

r2
0.8
0.6
0.4
0.2
chr2:40630678
15
10
5
0
−log10(p−value)
100
80
60
40
20
0
Recombination rate (cM/Mb)
SLC8A1−AS1→
←SLC8A1
40.2
40.4
40.6
40.8
41
Position on chr2 (Mb)

date:	Wed Aug 1 12:31:21 2018
build:	hg19
display range:	chr2:40130678−41130678 [40130678−41130678]
hilite range:	0 − 0 [0 − 0]
reference SNP:	chr2:40630678
number of SNPs plotted:	5322
min P−value:	1.2E−14 [chr2:40630678]
max P−value:	10E−1 [chr2:40207807]

Make more plots at http://csg.sph.umich.edu/locuszoom/
