# Supplementary material for: Bone mineral density loci specific to the skull portray potential pleiotropic effects on craniosynostosis
Source: Commun Biol. 2023 Jul 4;6:691. doi: 10.1038/s42003-023-04869-0 (PMC10319806; doi:10.1038/s42003-023-04869-0)
Supplement: Supplementary file 6 — Supplementary Data 3 [file 42003_2023_4869_MOESM6_ESM.zip › loci/chr2_84998783-85998783.pdf]

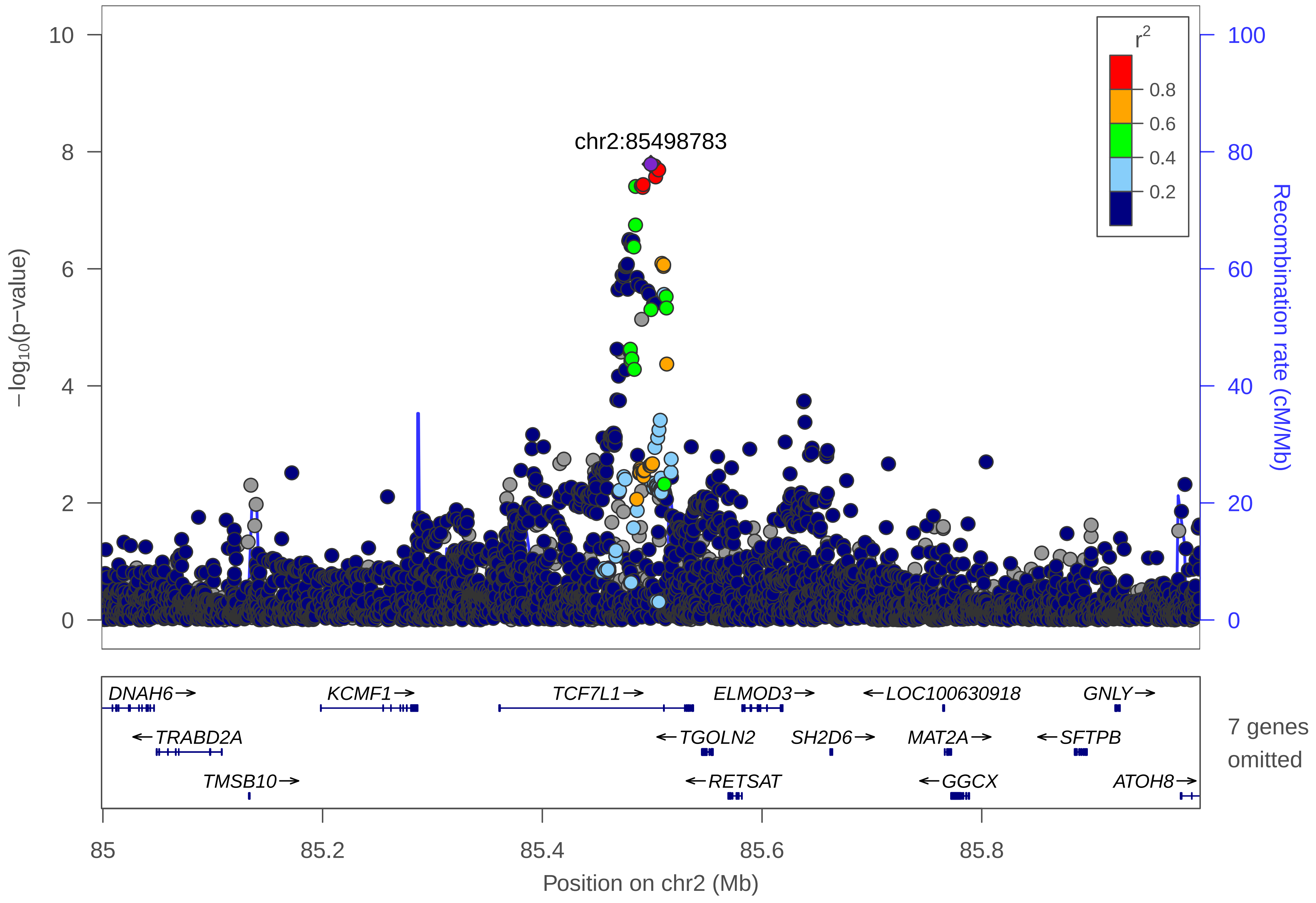

chr2:85498783
r2
0.8
0.6
0.4
0.2
−log10(p−value)
Recombination rate (cM/Mb)
0
2
4
6
8
10
0
20
40
60
80
100
DNAH6→
KCMF1→
TCF7L1→
ELMOD3→
←LOC100630918
GNLY→
←TRABD2A
←TGOLN2
SH2D6→
MAT2A→
←SFTPB
TMSB10→
←RETSAT
←GGCX
ATOH8→
7 genes omitted
85
85.2
85.4
85.6
85.8
Position on chr2 (Mb)

date:	Wed Aug 1 12:31:55 2018
build:	hg19
display range:	chr2:84998783−85998783 [84998783−85998783]
hilite range:	0 − 0 [0 − 0]
reference SNP:	chr2:85498783
number of SNPs plotted:	4237
min P−value:	1.63E−8 [chr2:85498783]
max P−value:	1E0 [chr2:85824039]
omitted Genes:	CAPG, VAMP8, VAMP5
omitted Genes:	RNF181, TMEM150A, USP39
omitted Genes:	C2orf68

Make more plots at http://csg.sph.umich.edu/locuszoom/
