# Supplementary material for: Bone mineral density loci specific to the skull portray potential pleiotropic effects on craniosynostosis
Source: Commun Biol. 2023 Jul 4;6:691. doi: 10.1038/s42003-023-04869-0 (PMC10319806; doi:10.1038/s42003-023-04869-0)
Supplement: Supplementary file 6 — Supplementary Data 3 [file 42003_2023_4869_MOESM6_ESM.zip › loci/chr3_146663978-147663978.pdf]

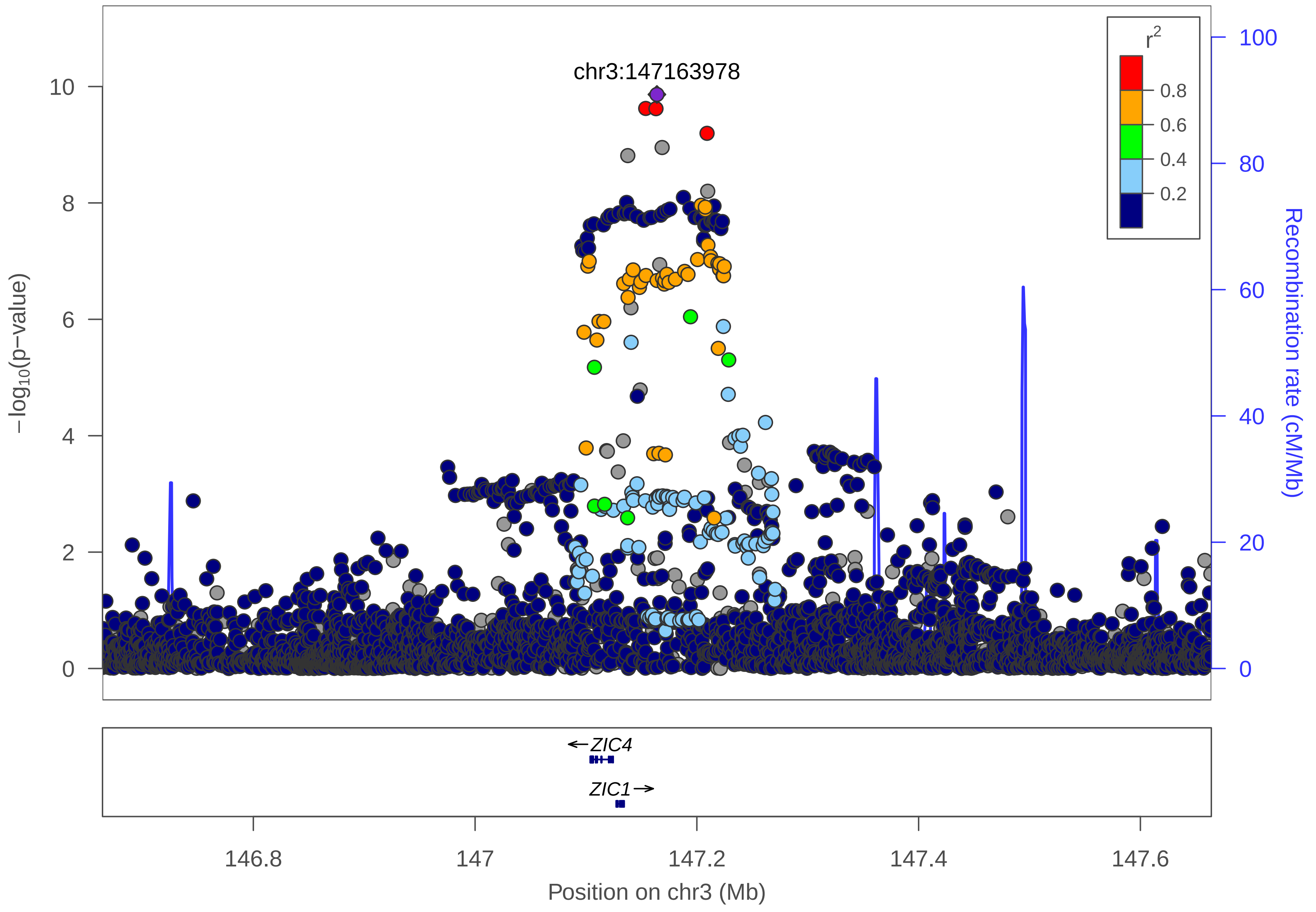

chr3:147163978
r2
0.8
0.6
0.4
0.2
−log10(p−value)
0
2
4
6
8
10
Recombination rate (cM/Mb)
0
20
40
60
80
100
ZIC4
ZIC1
146.8
147
147.2
147.4
147.6
Position on chr3 (Mb)

date:	Wed Aug 1 12:34:14 2018
build:	hg19
display range:	chr3:146663978−147663978 [146663978−147663978]
hilite range:	0 − 0 [0 − 0]
reference SNP:	chr3:147163978
number of SNPs plotted:	3855
min P−value:	1.37E−10 [chr3:147163978]
max P−value:	9.99E−1 [chr3:146777823]

Make more plots at http://csg.sph.umich.edu/locuszoom/
