# Supplementary material for: Bone mineral density loci specific to the skull portray potential pleiotropic effects on craniosynostosis
Source: Commun Biol. 2023 Jul 4;6:691. doi: 10.1038/s42003-023-04869-0 (PMC10319806; doi:10.1038/s42003-023-04869-0)
Supplement: Supplementary file 6 — Supplementary Data 3 [file 42003_2023_4869_MOESM6_ESM.zip › loci/chr3_156192207-157192207.pdf]

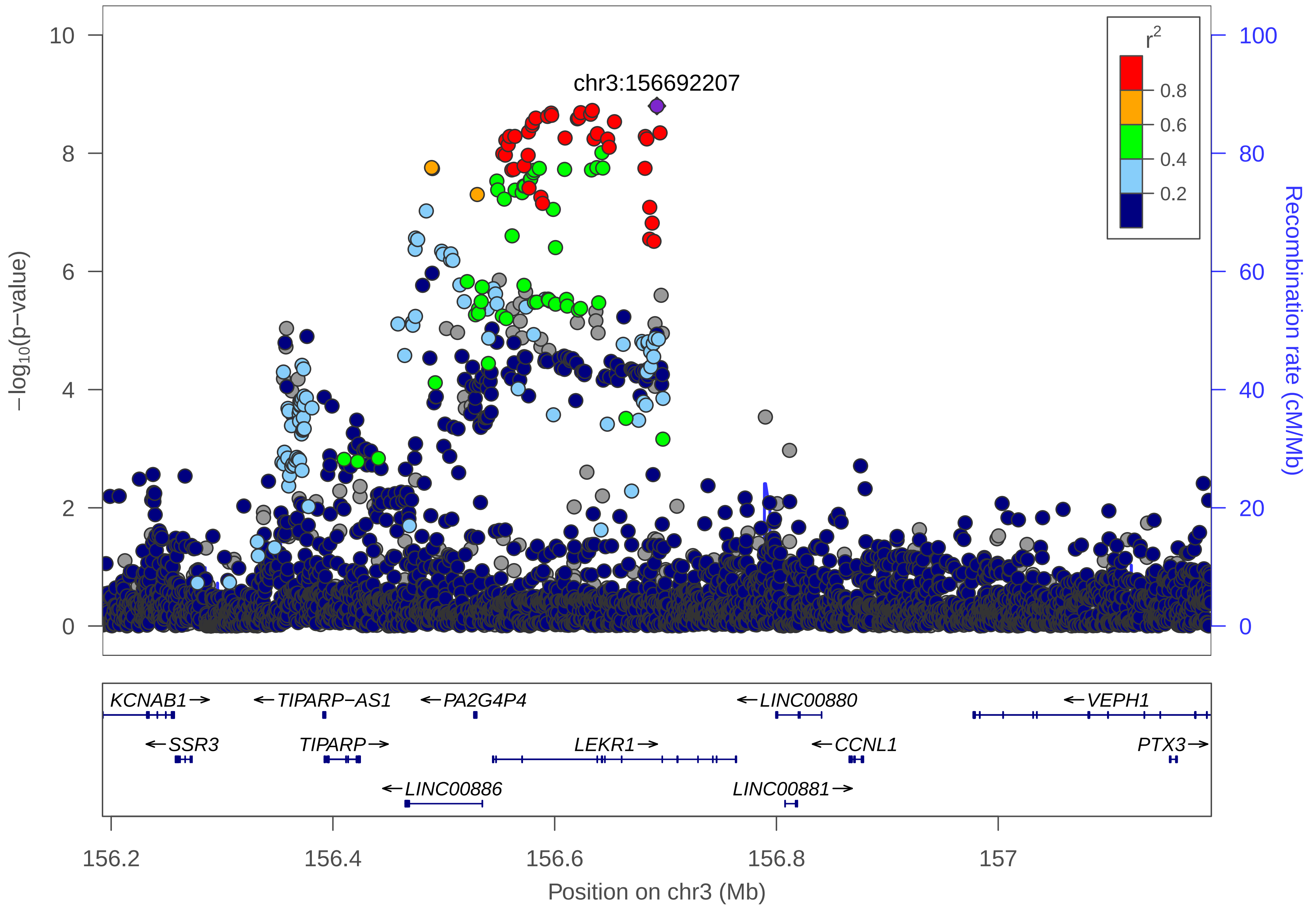
chr3:156692207
r2
0.8
0.6
0.4
0.2
10
8
6
4
2
0
100
80
60
40
20
0
−log10(p−value)
Recombination rate (cM/Mb)
KCNAB1→
←TIPARP−AS1
←PA2G4P4
←LINC00880
←VEPH1
←SSR3
TIPARP→
LEKR1→
←CCNL1
PTX3→
←LINC00886
LINC00881→
156.2
156.4
156.6
156.8
157
Position on chr3 (Mb)

date:	Wed Aug 1 12:34:49 2018
build:	hg19
display range:	chr3:156192207−157192207 [156192207−157192207]
hilite range:	0 − 0 [0 − 0]
reference SNP:	chr3:156692207
number of SNPs plotted:	4141
min P−value:	1.59E−9 [chr3:156692207]
max P−value:	10E−1 [chr3:156289843]

Make more plots at http://csg.sph.umich.edu/locuszoom/
