# Supplementary material for: Bone mineral density loci specific to the skull portray potential pleiotropic effects on craniosynostosis
Source: Commun Biol. 2023 Jul 4;6:691. doi: 10.1038/s42003-023-04869-0 (PMC10319806; doi:10.1038/s42003-023-04869-0)
Supplement: Supplementary file 6 — Supplementary Data 3 [file 42003_2023_4869_MOESM6_ESM.zip › loci/chr3_40638172-41638172.pdf]

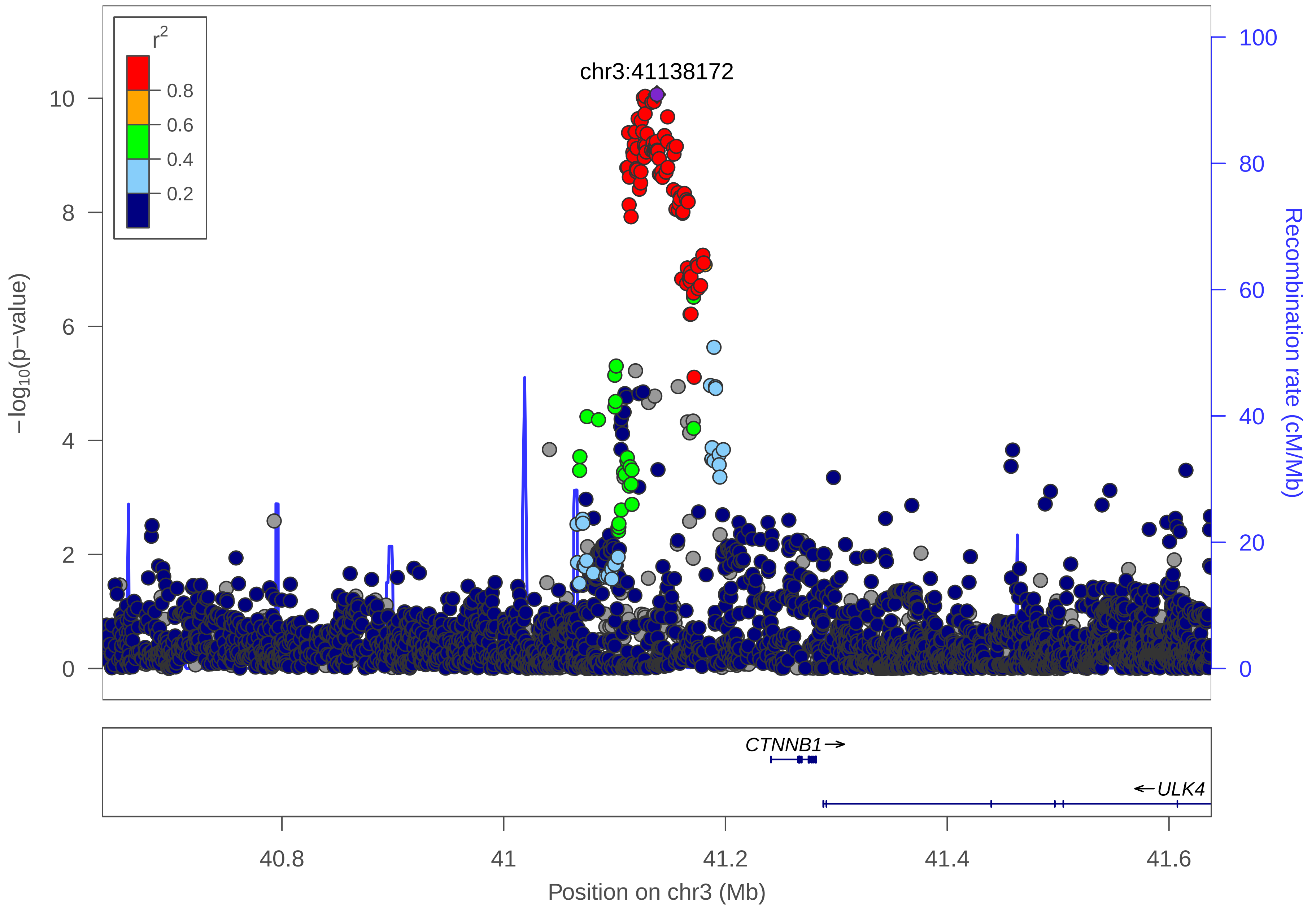

r2
0.8
0.6
0.4
0.2
chr3:41138172
10
8
6
4
2
0
−log10(p−value)
100
80
60
40
20
0
Recombination rate (cM/Mb)
CTNNB1
ULK4
40.8
41
41.2
41.4
41.6
Position on chr3 (Mb)

date:	Wed Aug 1 12:33:39 2018
build:	hg19
display range:	chr3:40638172−41638172 [40638172−41638172]
hilite range:	0 − 0 [0 − 0]
reference SNP:	chr3:41138172
number of SNPs plotted:	3665
min P−value:	8.56E−11 [chr3:41138172]
max P−value:	10E−1 [chr3:41485592]

Make more plots at http://csg.sph.umich.edu/locuszoom/
