# Supplementary material for: Bone mineral density loci specific to the skull portray potential pleiotropic effects on craniosynostosis
Source: Commun Biol. 2023 Jul 4;6:691. doi: 10.1038/s42003-023-04869-0 (PMC10319806; doi:10.1038/s42003-023-04869-0)
Supplement: Supplementary file 6 — Supplementary Data 3 [file 42003_2023_4869_MOESM6_ESM.zip › loci/chr4_494414-1494414.pdf]

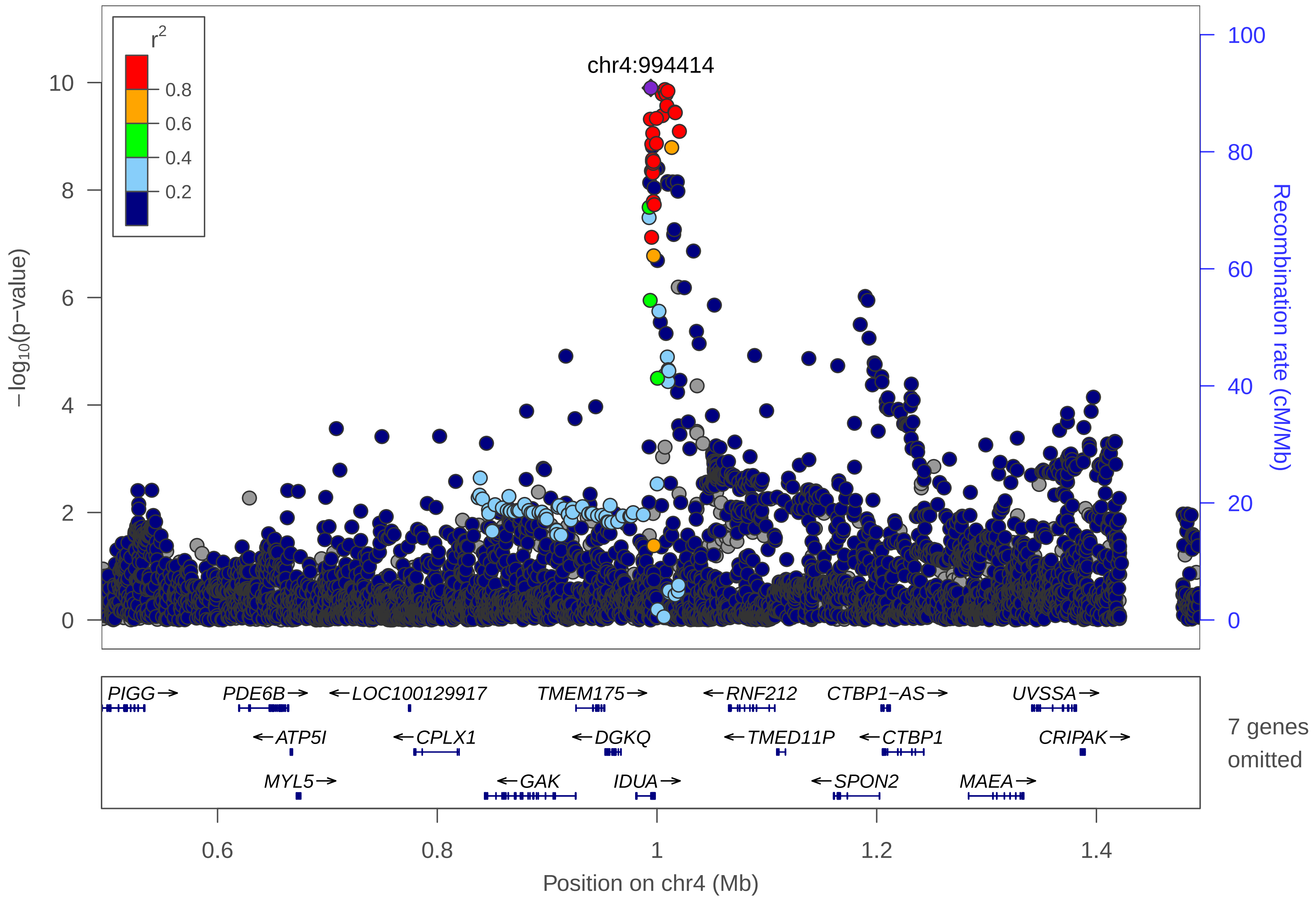

r2
0.8
0.6
0.4
0.2
chr4:994414
10
8
6
4
2
0
−log10(p−value)
100
80
60
40
20
0
Recombination rate (cM/Mb)
PIGG→
PDE6B→
←LOC100129917
TMEM175→
←RNF212
CTBP1−AS→
UVSSA→
←ATP5I
←CPLX1
←DGKQ
←TMED11P
←CTBP1
CRIPAK→
MYL5→
←GAK
IDUA→
←SPON2
MAEA→
7 genes omitted
0.6
0.8
1
1.2
1.4
Position on chr4 (Mb)

date:	Wed Aug 1 12:35:24 2018
build:	hg19
display range:	chr4:494414−1494414 [494414−1494414]
hilite range:	0 − 0 [0 − 0]
reference SNP:	chr4:994414
number of SNPs plotted:	5283
min P−value:	1.26E−10 [chr4:994414]
max P−value:	9.99E−1 [chr4:657857]
omitted Genes:	MFSD7, PCGF3, SLC26A1
omitted Genes:	FGFRL1, LOC100130872, CTBP1−AS2
omitted Genes:	NKX1−1

Make more plots at http://csg.sph.umich.edu/locuszoom/
