# Supplementary material for: Bone mineral density loci specific to the skull portray potential pleiotropic effects on craniosynostosis
Source: Commun Biol. 2023 Jul 4;6:691. doi: 10.1038/s42003-023-04869-0 (PMC10319806; doi:10.1038/s42003-023-04869-0)
Supplement: Supplementary file 6 — Supplementary Data 3 [file 42003_2023_4869_MOESM6_ESM.zip › loci/chr4_88331249-89331249.pdf]

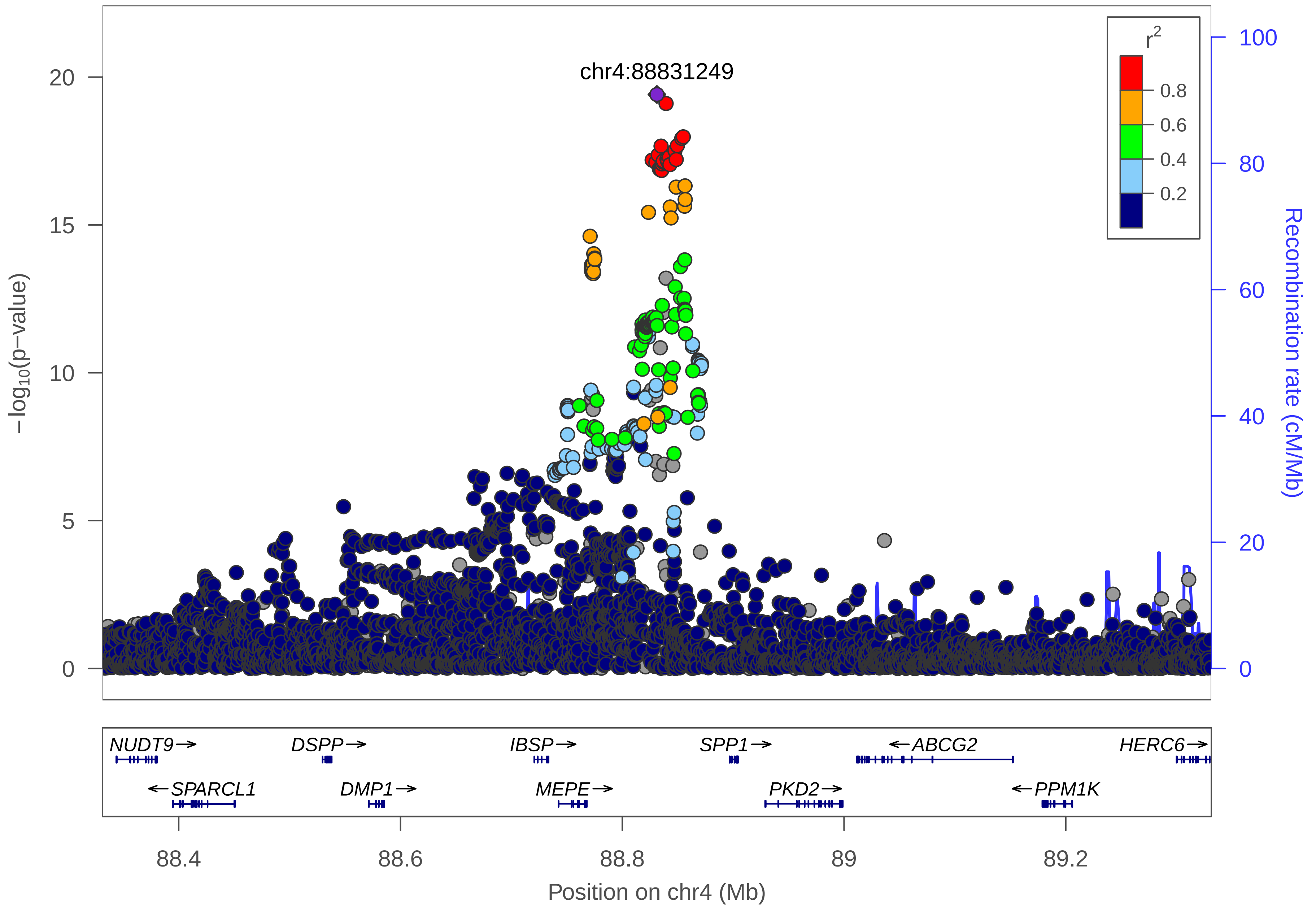

chr4:88831249
r2
0.8
0.6
0.4
0.2
−log10(p−value)
Recombination rate (cM/Mb)
NUDT9→
DSPP→
IBSP→
SPP1→
←ABCG2
HERC6→
←SPARCL1
DMP1→
MEPE→
PKD2→
←PPM1K
88.4
88.6
88.8
89
89.2
Position on chr4 (Mb)

date:	Wed Aug 1 12:35:59 2018
build:	hg19
display range:	chr4:88331249−89331249 [88331249−89331249]
hilite range:	0 − 0 [0 − 0]
reference SNP:	chr4:88831249
number of SNPs plotted:	4772
min P−value:	3.87E−20 [chr4:88831249]
max P−value:	10E−1 [chr4:88992813]

Make more plots at http://csg.sph.umich.edu/locuszoom/
