# Supplementary material for: Bone mineral density loci specific to the skull portray potential pleiotropic effects on craniosynostosis
Source: Commun Biol. 2023 Jul 4;6:691. doi: 10.1038/s42003-023-04869-0 (PMC10319806; doi:10.1038/s42003-023-04869-0)
Supplement: Supplementary file 6 — Supplementary Data 3 [file 42003_2023_4869_MOESM6_ESM.zip › loci/chr5_111688456-112688456.pdf]

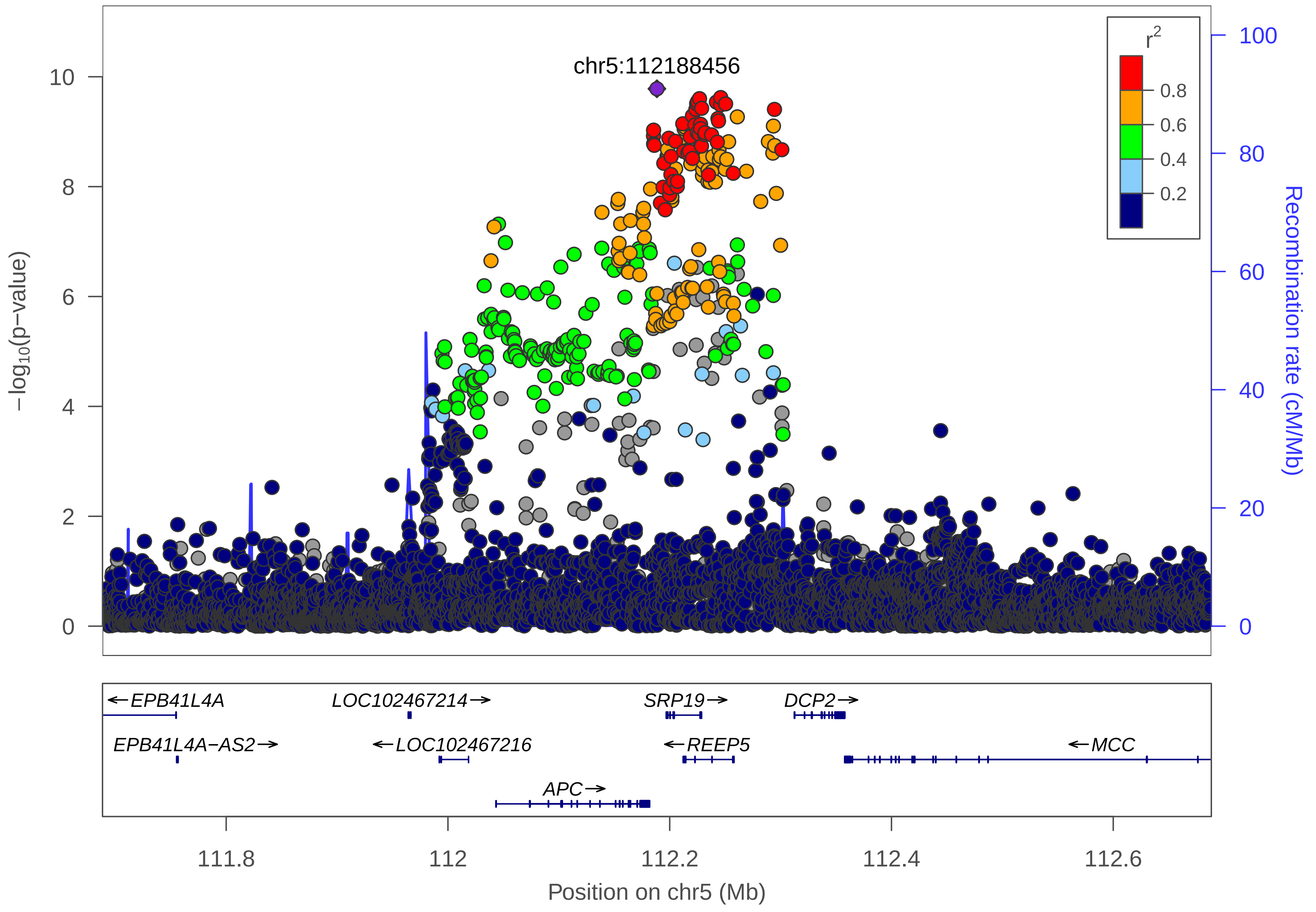

chr5:112188456
r2
0.8
0.6
0.4
0.2
10
8
6
4
2
0
−log10(p−value)
100
80
60
40
20
0
Recombination rate (cM/Mb)
←EPB41L4A
LOC102467214→
SRP19→
DCP2→
EPB41L4A−AS2→
←LOC102467216
←REEP5
←MCC
APC→
111.8
112
112.2
112.4
112.6
Position on chr5 (Mb)

date:	Wed Aug 1 12:37:08 2018
build:	hg19
display range:	chr5:111688456−112688456 [111688456−112688456]
hilite range:	0 − 0 [0 − 0]
reference SNP:	chr5:112188456
number of SNPs plotted:	5515
min P−value:	1.66E−10 [chr5:112188456]
max P−value:	1E0 [chr5:112519471]

Make more plots at http://csg.sph.umich.edu/locuszoom/
