# Supplementary material for: Bone mineral density loci specific to the skull portray potential pleiotropic effects on craniosynostosis
Source: Commun Biol. 2023 Jul 4;6:691. doi: 10.1038/s42003-023-04869-0 (PMC10319806; doi:10.1038/s42003-023-04869-0)
Supplement: Supplementary file 6 — Supplementary Data 3 [file 42003_2023_4869_MOESM6_ESM.zip › loci/chr5_122360170-123360170.pdf]

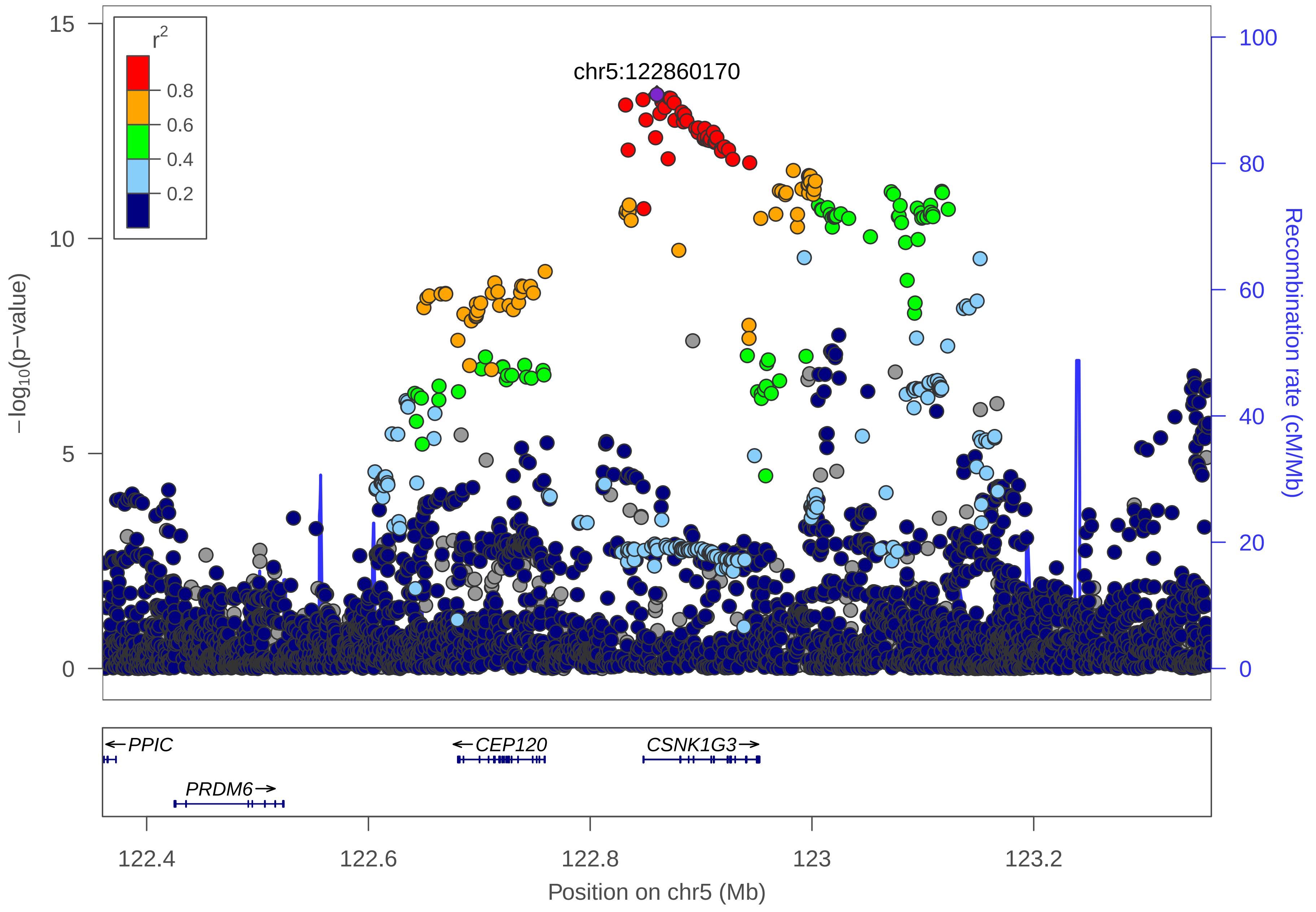

r2
0.8
0.6
0.4
0.2
chr5:122860170
15
10
5
0
−log10(p−value)
100
80
60
40
20
0
Recombination rate (cM/Mb)
PPIC
CEP120
CSNK1G3
PRDM6
122.4
122.6
122.8
123
123.2
Position on chr5 (Mb)

date:	Wed Aug 1 12:37:44 2018
build:	hg19
display range:	chr5:122360170−123360170 [122360170−123360170]
hilite range:	0 − 0 [0 − 0]
reference SNP:	chr5:122860170
number of SNPs plotted:	4211
min P−value:	4.47E−14 [chr5:122860170]
max P−value:	10E−1 [chr5:123147284]

Make more plots at http://csg.sph.umich.edu/locuszoom/
