# Supplementary material for: Bone mineral density loci specific to the skull portray potential pleiotropic effects on craniosynostosis
Source: Commun Biol. 2023 Jul 4;6:691. doi: 10.1038/s42003-023-04869-0 (PMC10319806; doi:10.1038/s42003-023-04869-0)
Supplement: Supplementary file 6 — Supplementary Data 3 [file 42003_2023_4869_MOESM6_ESM.zip › loci/chr5_72118265-73118265.pdf]

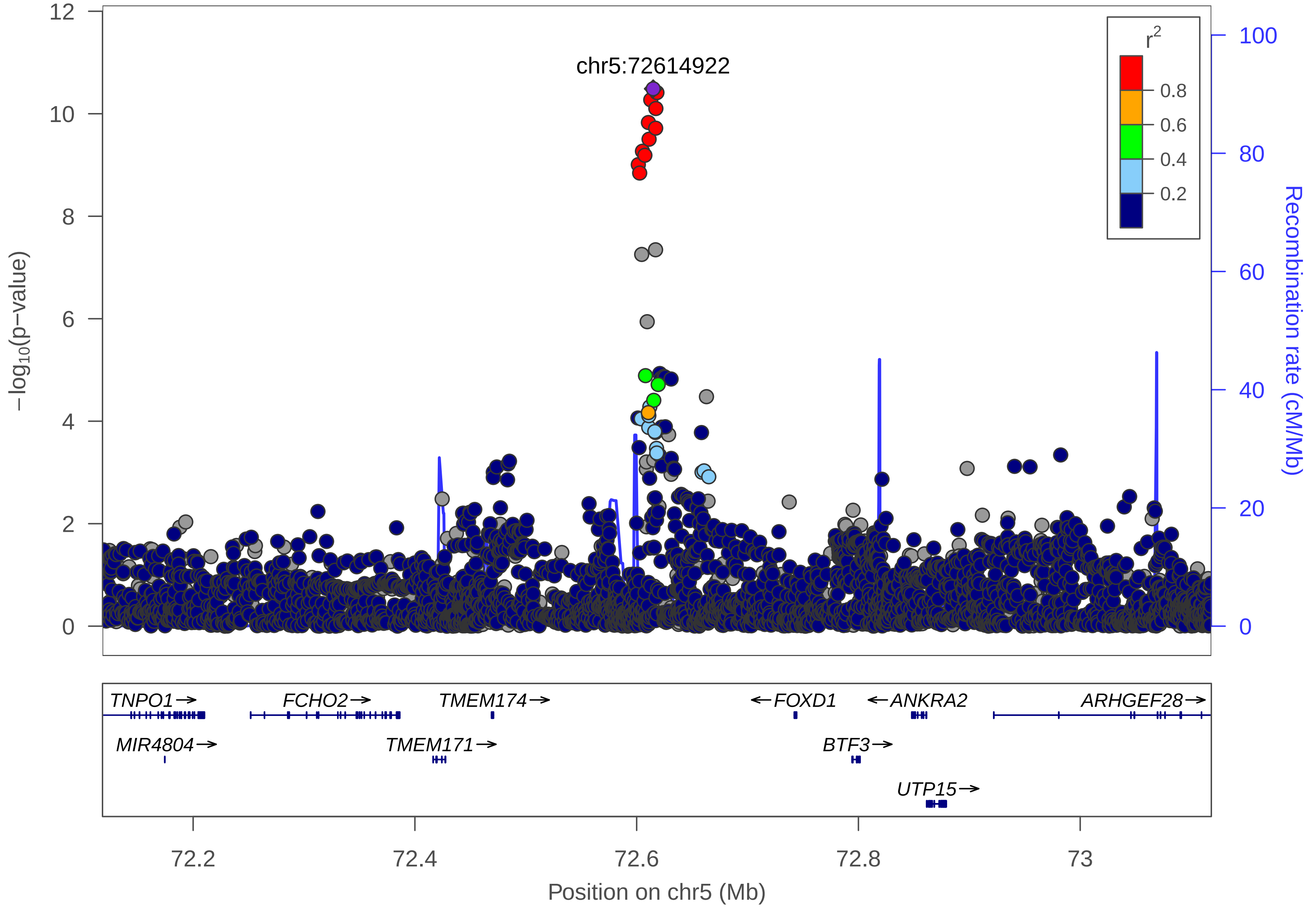

chr5:72614922
r2
0.8
0.6
0.4
0.2
−log10(p−value)
Recombination rate (cM/Mb)
0
2
4
6
8
10
12
0
20
40
60
80
100
TNPO1→
FCHO2→
TMEM174→
←FOXD1
←ANKRA2
ARHGEF28→
MIR4804→
TMEM171→
BTF3→
UTP15→
72.2
72.4
72.6
72.8
73
Position on chr5 (Mb)

date:	Wed Aug 1 12:36:33 2018
build:	hg19
display range:	chr5:72118265−73118265 [72118265−73118265]
hilite range:	0 − 0 [0 − 0]
reference SNP:	chr5:72614922
number of SNPs plotted:	3674
min P−value:	3.26E−11 [chr5:72614922]
max P−value:	10E−1 [chr5:72428831]

Make more plots at http://csg.sph.umich.edu/locuszoom/
