# Supplementary material for: Bone mineral density loci specific to the skull portray potential pleiotropic effects on craniosynostosis
Source: Commun Biol. 2023 Jul 4;6:691. doi: 10.1038/s42003-023-04869-0 (PMC10319806; doi:10.1038/s42003-023-04869-0)
Supplement: Supplementary file 6 — Supplementary Data 3 [file 42003_2023_4869_MOESM6_ESM.zip › loci/chr6_126425630-127725630.pdf]

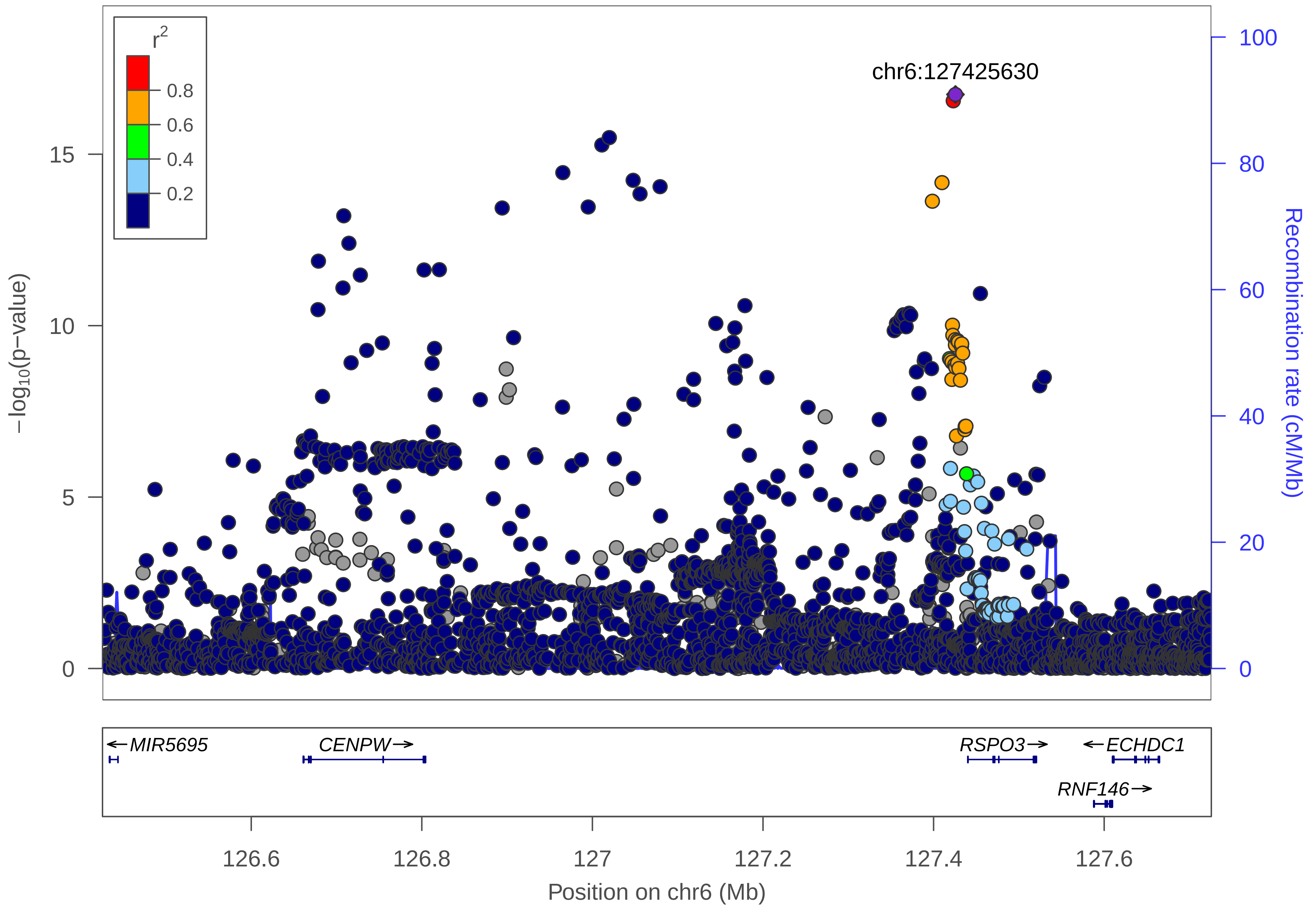

r2
0.8
0.6
0.4
0.2
chr6:127425630
15
10
5
0
−log10(p−value)
100
80
60
40
20
0
Recombination rate (cM/Mb)
← MIR5695
CENPW →
RSPO3 →
← ECHDC1
RNF146 →
126.6
126.8
127
127.2
127.4
127.6
Position on chr6 (Mb)

date:	Wed Aug 1 16:32:52 2018
build:	hg19
display range:	chr6:126425630−127725630 [126425630−127725630]
hilite range:	0 − 0 [0 − 0]
reference SNP:	chr6:127425630
number of SNPs plotted:	3274
min P−value:	1.81E−17 [chr6:127425630]
max P−value:	10E−1 [chr6:127096288]

Make more plots at http://csg.sph.umich.edu/locuszoom/
