# Supplementary material for: Bone mineral density loci specific to the skull portray potential pleiotropic effects on craniosynostosis
Source: Commun Biol. 2023 Jul 4;6:691. doi: 10.1038/s42003-023-04869-0 (PMC10319806; doi:10.1038/s42003-023-04869-0)
Supplement: Supplementary file 6 — Supplementary Data 3 [file 42003_2023_4869_MOESM6_ESM.zip › loci/chr6_129848257-130848257.pdf]

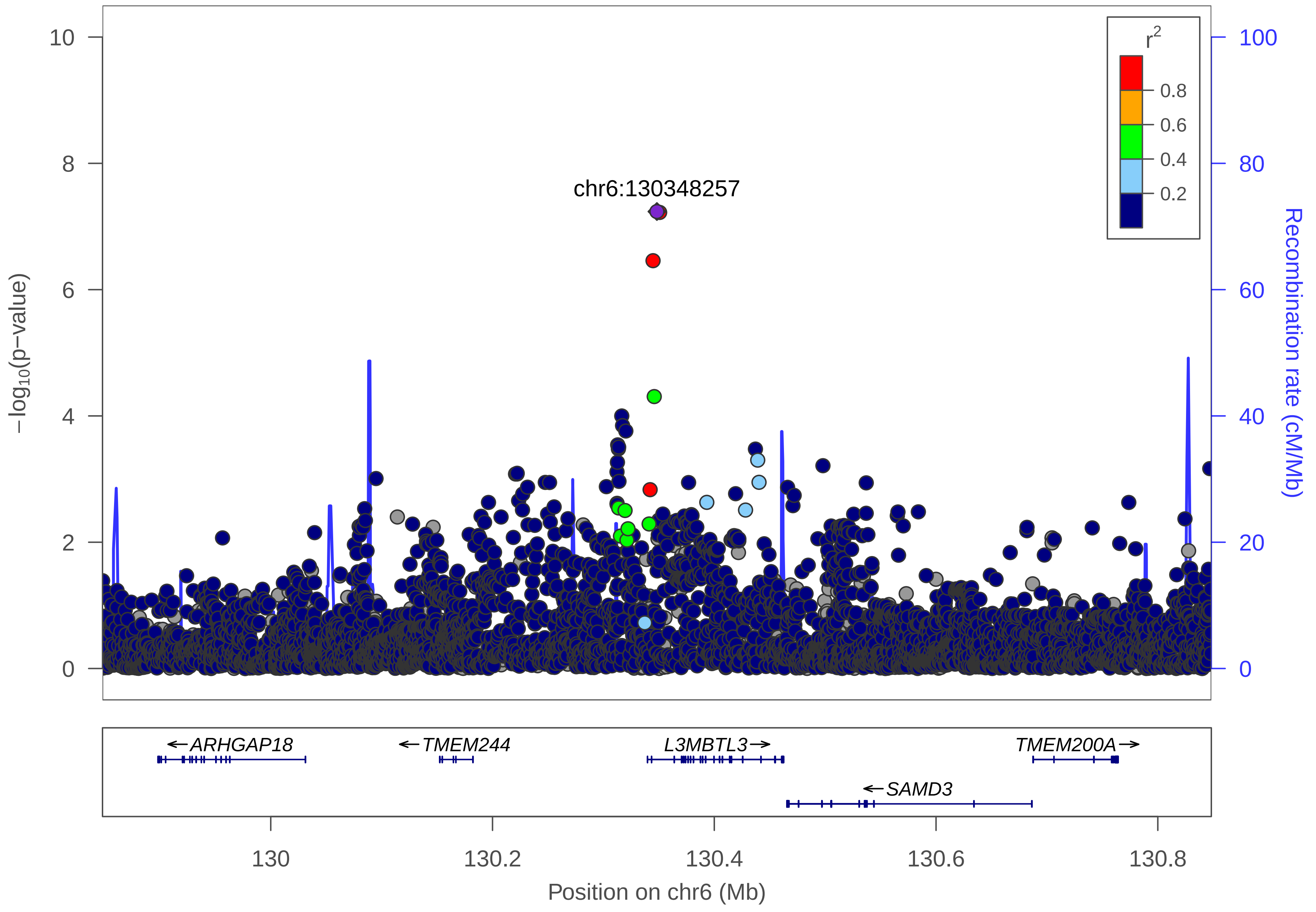

chr6:130348257
r2
0.8
0.6
0.4
0.2
−log10(p−value)
Recombination rate (cM/Mb)
0
2
4
6
8
10
0
20
40
60
80
100
ARHGAP18
TMEM244
L3MBTL3
TMEM200A
SAMD3
130
130.2
130.4
130.6
130.8
Position on chr6 (Mb)

date:	Wed Aug 1 12:42:29 2018
build:	hg19
display range:	chr6:129848257−130848257 [129848257−130848257]
hilite range:	0 − 0 [0 − 0]
reference SNP:	chr6:130348257
number of SNPs plotted:	5046
min P−value:	5.8E−8 [chr6:130348257]
max P−value:	10E−1 [chr6:129976794]

Make more plots at http://csg.sph.umich.edu/locuszoom/
