# Supplementary material for: Bone mineral density loci specific to the skull portray potential pleiotropic effects on craniosynostosis
Source: Commun Biol. 2023 Jul 4;6:691. doi: 10.1038/s42003-023-04869-0 (PMC10319806; doi:10.1038/s42003-023-04869-0)
Supplement: Supplementary file 6 — Supplementary Data 3 [file 42003_2023_4869_MOESM6_ESM.zip › loci/chr6_133025630-134025630.pdf]

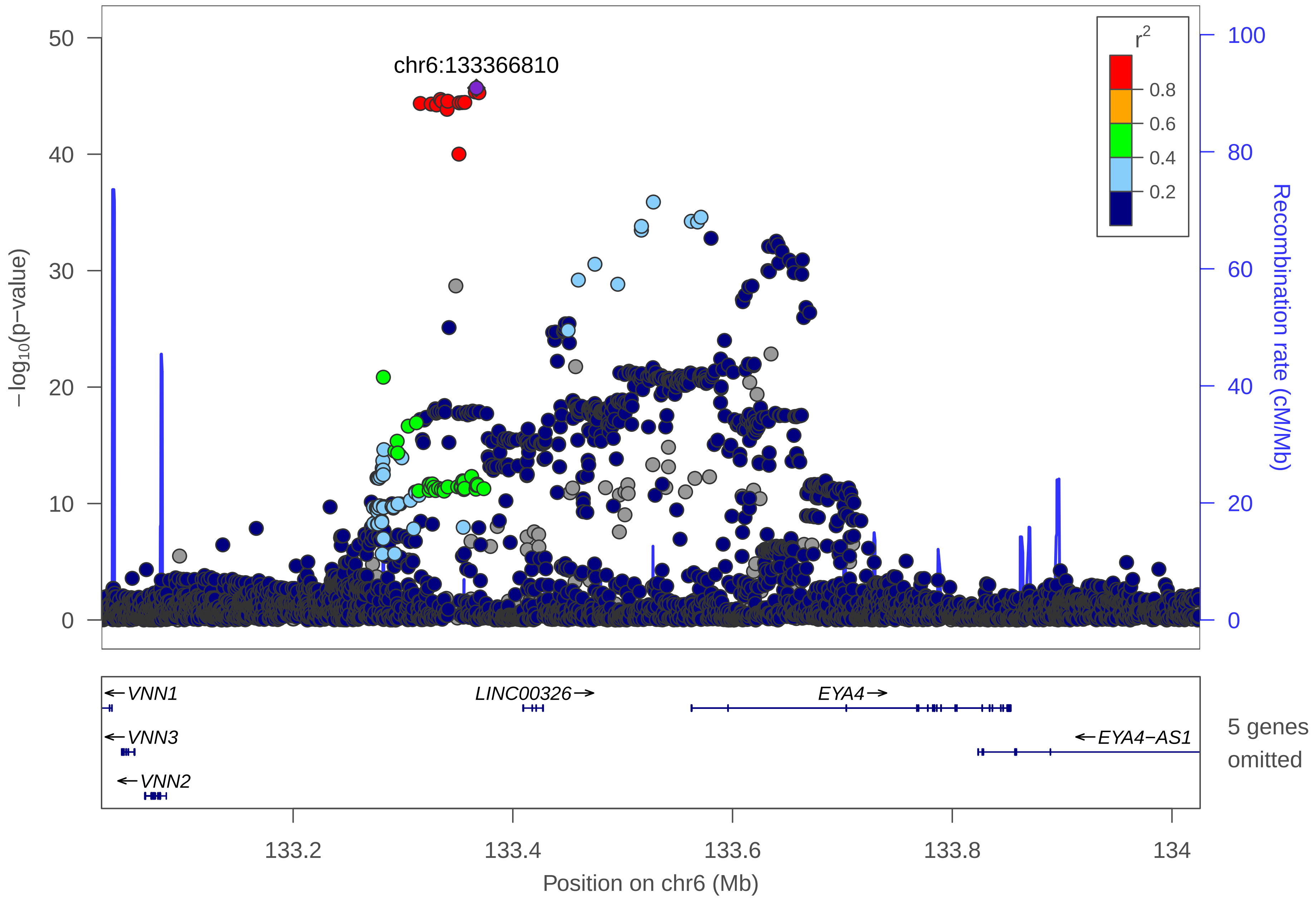

chr6:133366810
r2
0.8
0.6
0.4
0.2
−log10(p−value)
50
40
30
20
10
0
100
80
60
40
20
0
Recombination rate (cM/Mb)
←VNN1
LINC00326→
EYA4→
←VNN3
←EYA4−AS1
←VNN2
5 genes omitted
133.2
133.4
133.6
133.8
134
Position on chr6 (Mb)

date:	Wed Aug 1 16:37:47 2018
build:	hg19
display range:	chr6:133025630−134025630 [133025630−134025630]
hilite range:	0 − 0 [0 − 0]
reference SNP:	chr6:133366810
number of SNPs plotted:	4296
min P−value:	2.02E−46 [chr6:133366810]
max P−value:	10E−1 [chr6:133293326]
omitted Genes:	SLC18B1, RPS12, SNORD101
omitted Genes:	SNORD100, SNORA33

Make more plots at http://csg.sph.umich.edu/locuszoom/
