# Supplementary material for: Bone mineral density loci specific to the skull portray potential pleiotropic effects on craniosynostosis
Source: Commun Biol. 2023 Jul 4;6:691. doi: 10.1038/s42003-023-04869-0 (PMC10319806; doi:10.1038/s42003-023-04869-0)
Supplement: Supplementary file 6 — Supplementary Data 3 [file 42003_2023_4869_MOESM6_ESM.zip › loci/chr6_151374122-152374122.pdf]

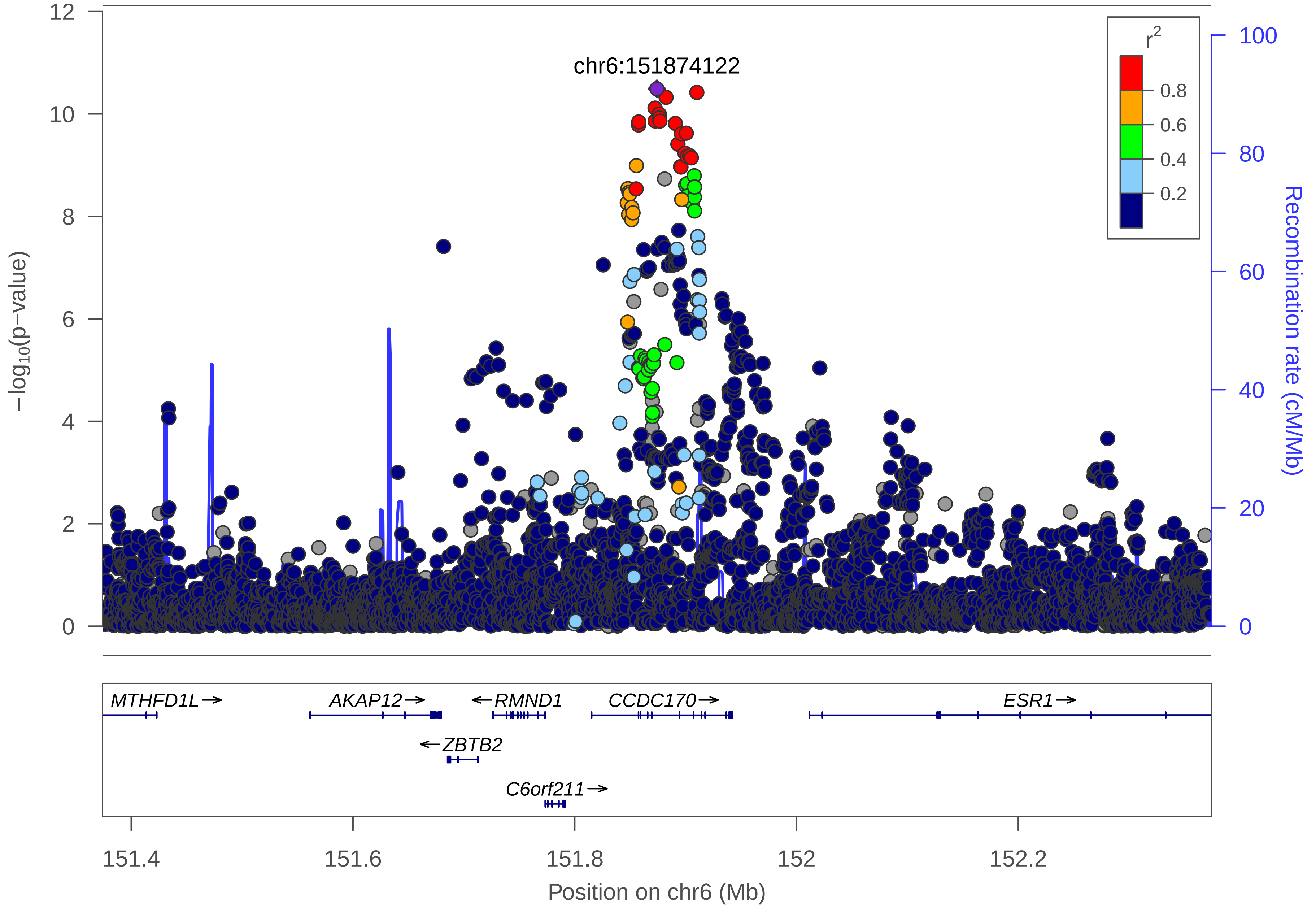
chr6:151874122
r2
0.8
0.6
0.4
0.2
12
10
8
6
4
2
0
−log10(p−value)
100
80
60
40
20
0
Recombination rate (cM/Mb)
MTHFD1L→
AKAP12→
←RMND1
CCDC170→
ESR1→
←ZBTB2
C6orf211→
151.4
151.6
151.8
152
152.2
Position on chr6 (Mb)

date:	Wed Aug 1 12:44:16 2018
build:	hg19
display range:	chr6:151374122−152374122 [151374122−152374122]
hilite range:	0 − 0 [0 − 0]
reference SNP:	chr6:151874122
number of SNPs plotted:	4820
min P−value:	3.23E−11 [chr6:151874122]
max P−value:	1E0 [chr6:151636959]

Make more plots at http://csg.sph.umich.edu/locuszoom/
