# Supplementary material for: Bone mineral density loci specific to the skull portray potential pleiotropic effects on craniosynostosis
Source: Commun Biol. 2023 Jul 4;6:691. doi: 10.1038/s42003-023-04869-0 (PMC10319806; doi:10.1038/s42003-023-04869-0)
Supplement: Supplementary file 6 — Supplementary Data 3 [file 42003_2023_4869_MOESM6_ESM.zip › loci/chr6_44443982-45543982.pdf]

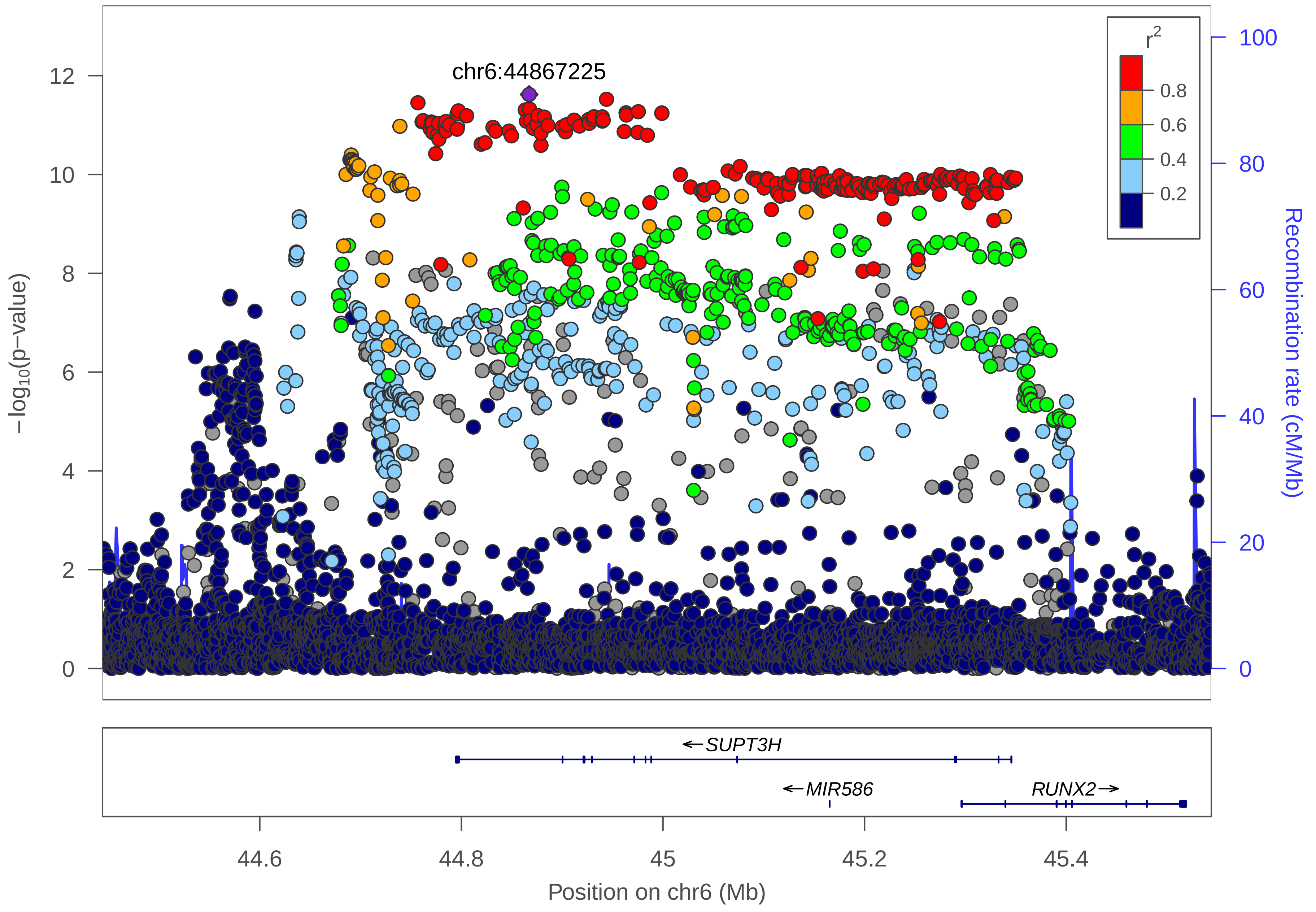

chr6:44867225
r2
0.8
0.6
0.4
0.2
−log10(p−value)
Recombination rate (cM/Mb)
12
10
8
6
4
2
0
100
80
60
40
20
0
SUPT3H
MIR586
RUNX2
44.6
44.8
45
45.2
45.4
Position on chr6 (Mb)

| | |
|---|---|
| date: | Wed Aug 1 14:44:37 2018 |
| build: | hg19 |
| display range: | chr6:44443982−45543982 [44443982−45543982] |
| hilite range: | 0 − 0 [ 0 − 0 ] |
| reference SNP: | chr6:44867225 |
| number of SNPs plotted: | 4765 |
| min P−value: | 2.4E−12 [chr6:44867225] |
| max P−value: | 9.98E−1 [chr6:44453194] |

Make more plots at http://csg.sph.umich.edu/locuszoom/
