# Supplementary material for: Bone mineral density loci specific to the skull portray potential pleiotropic effects on craniosynostosis
Source: Commun Biol. 2023 Jul 4;6:691. doi: 10.1038/s42003-023-04869-0 (PMC10319806; doi:10.1038/s42003-023-04869-0)
Supplement: Supplementary file 6 — Supplementary Data 3 [file 42003_2023_4869_MOESM6_ESM.zip › loci/chr6_53117914-54117914.pdf]

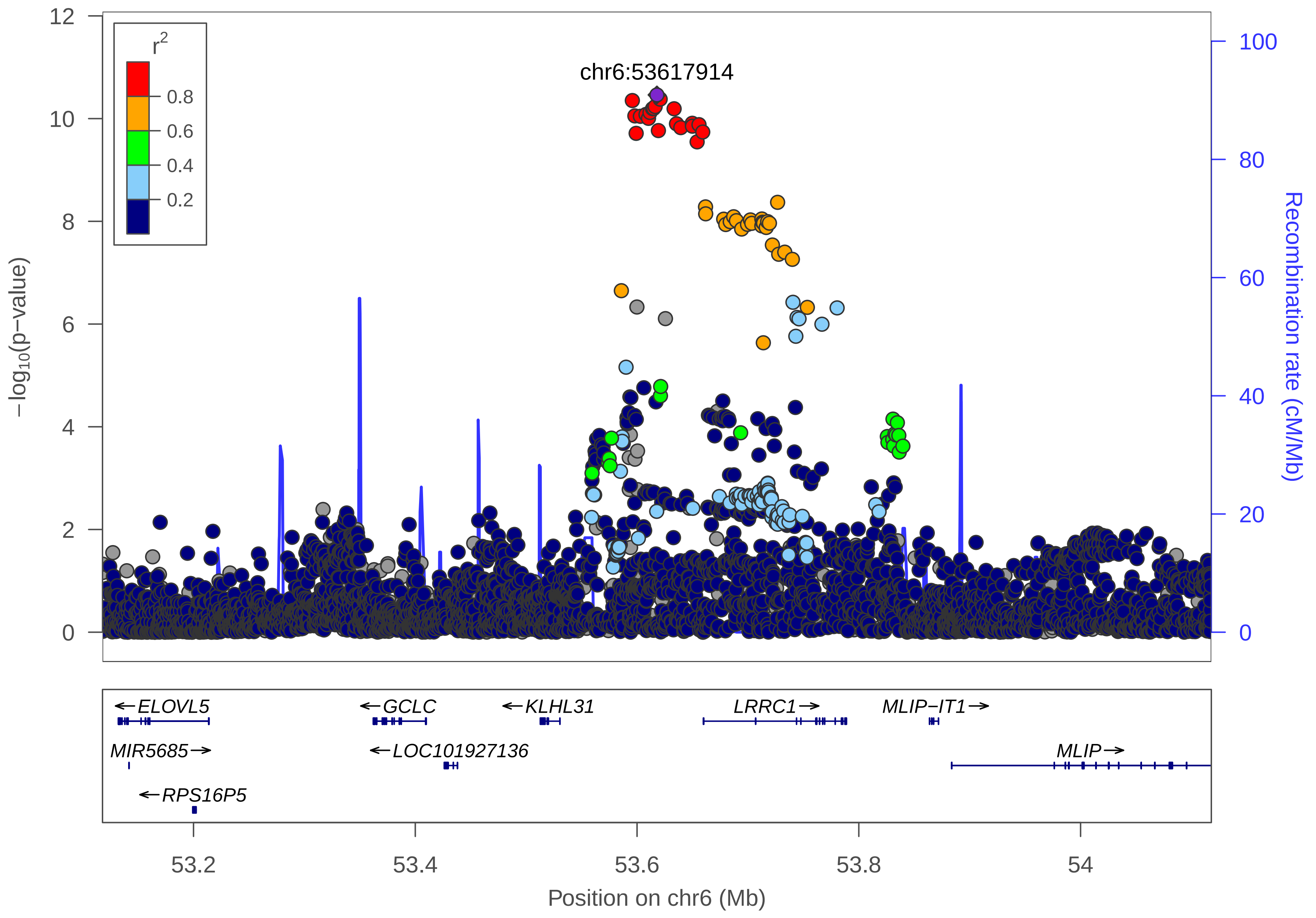

r2
0.8
0.6
0.4
0.2
chr6:53617914
12
10
8
6
4
2
0
−log10(p−value)
100
80
60
40
20
0
Recombination rate (cM/Mb)
←ELOVL5
←GCLC
←KLHL31
LRRC1→
MLIP−IT1→
MIR5685→
←LOC101927136
MLIP→
←RPS16P5
53.2
53.4
53.6
53.8
54
Position on chr6 (Mb)

date:	Wed Aug 1 12:39:30 2018
build:	hg19
display range:	chr6:53117914−54117914 [53117914−54117914]
hilite range:	0 − 0 [0 − 0]
reference SNP:	chr6:53617914
number of SNPs plotted:	4103
min P−value:	3.45E−11 [chr6:53617914]
max P−value:	10E−1 [chr6:53198685]

Make more plots at http://csg.sph.umich.edu/locuszoom/
