# Supplementary material for: Bone mineral density loci specific to the skull portray potential pleiotropic effects on craniosynostosis
Source: Commun Biol. 2023 Jul 4;6:691. doi: 10.1038/s42003-023-04869-0 (PMC10319806; doi:10.1038/s42003-023-04869-0)
Supplement: Supplementary file 6 — Supplementary Data 3 [file 42003_2023_4869_MOESM6_ESM.zip › loci/chr6_6439532-7439532.pdf]

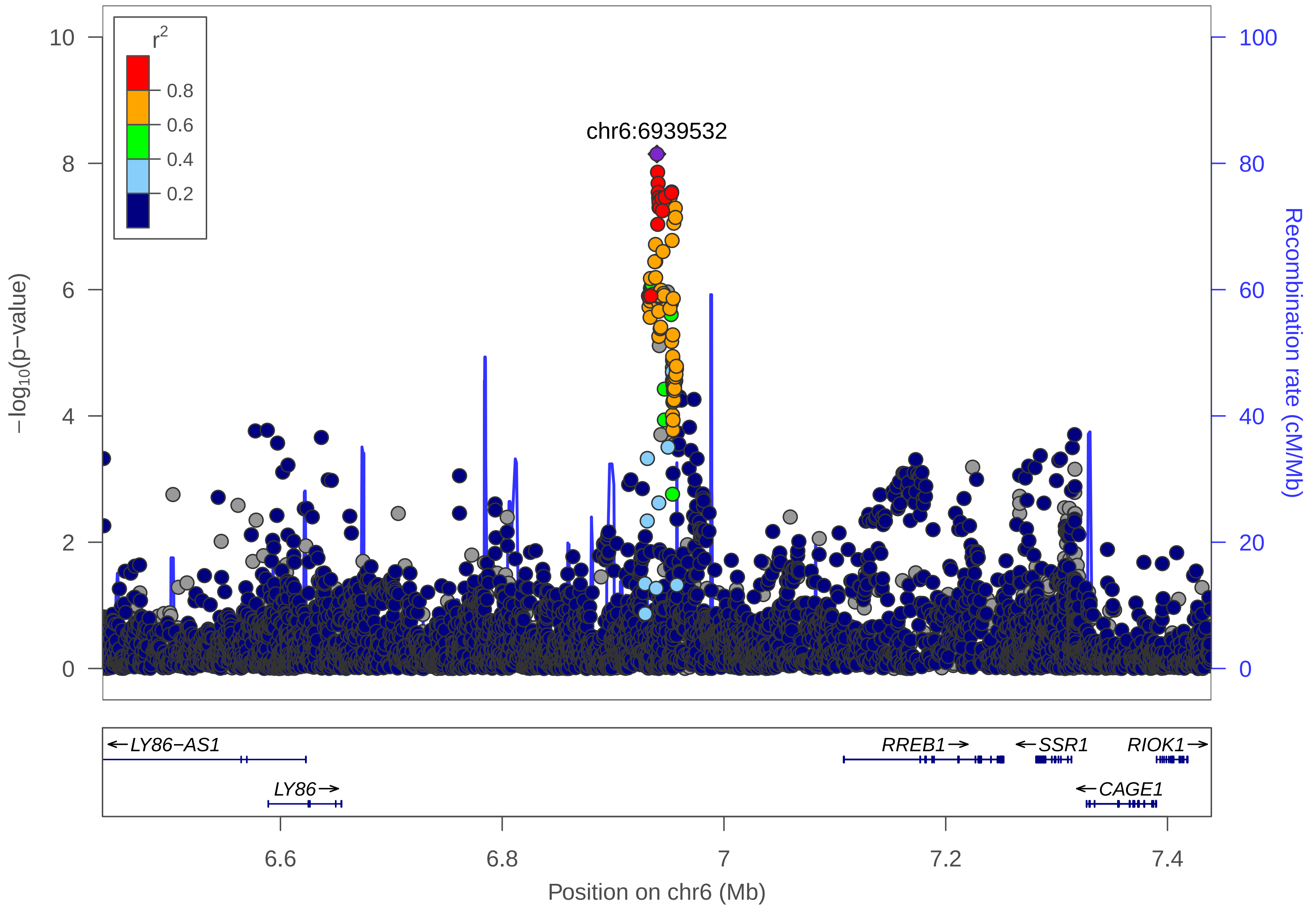

r2
0.8
0.6
0.4
0.2
chr6:6939532
10
8
6
4
2
0
−log10(p−value)
100
80
60
40
20
0
Recombination rate (cM/Mb)
←LY86−AS1
LY86→
RREB1→
←SSR1
RIOK1→
←CAGE1
6.6
6.8
7
7.2
7.4
Position on chr6 (Mb)

date:	Wed Aug 1 12:38:20 2018
build:	hg19
display range:	chr6:6439532−7439532 [6439532−7439532]
hilite range:	0 − 0 [0 − 0]
reference SNP:	chr6:6939532
number of SNPs plotted:	5623
min P−value:	7.12E−9 [chr6:6939532]
max P−value:	10E−1 [chr6:6718155]

Make more plots at http://csg.sph.umich.edu/locuszoom/
