# Supplementary material for: Bone mineral density loci specific to the skull portray potential pleiotropic effects on craniosynostosis
Source: Commun Biol. 2023 Jul 4;6:691. doi: 10.1038/s42003-023-04869-0 (PMC10319806; doi:10.1038/s42003-023-04869-0)
Supplement: Supplementary file 6 — Supplementary Data 3 [file 42003_2023_4869_MOESM6_ESM.zip › loci/chr6_73958737-74958737.pdf]

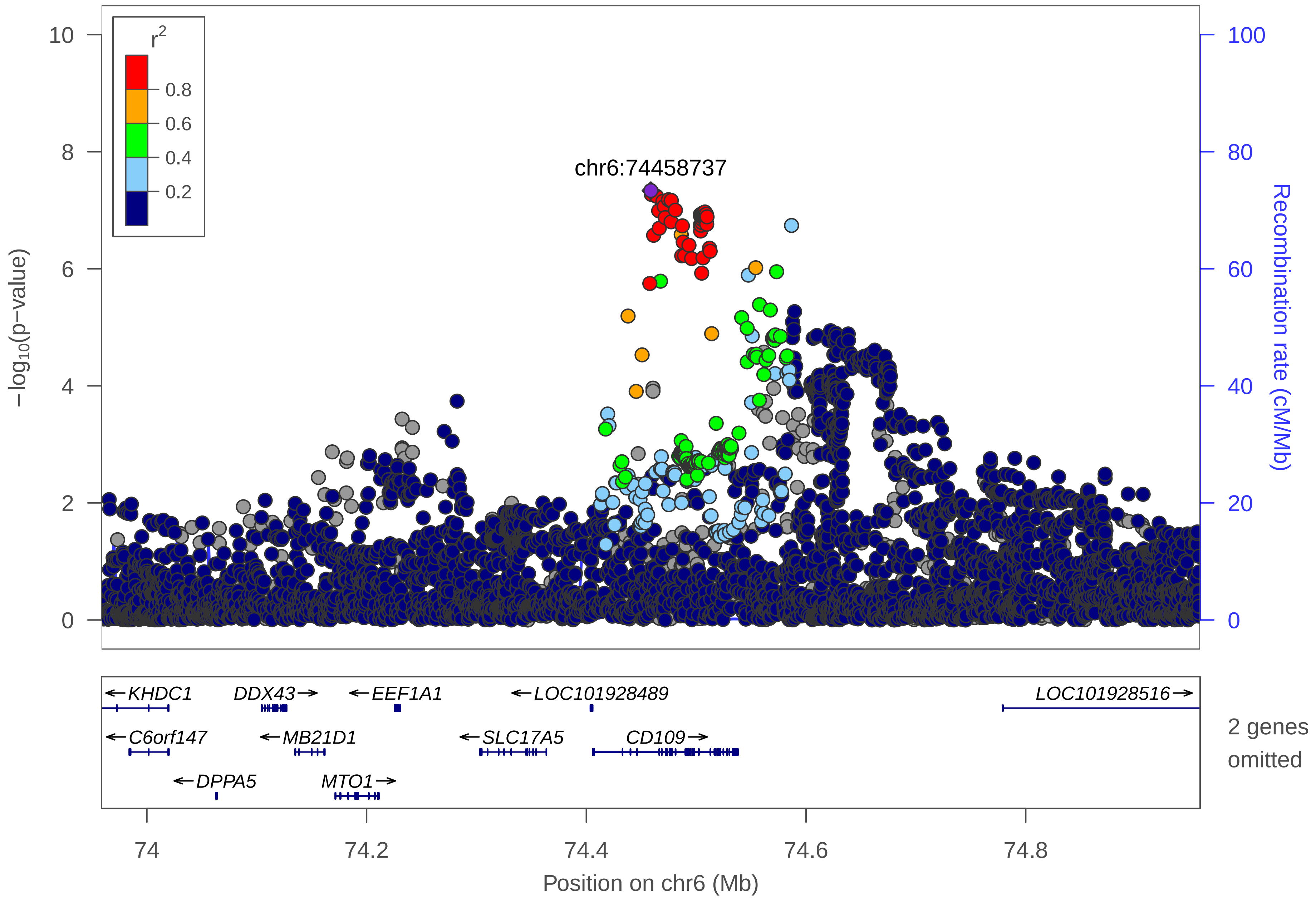

r2
0.8
0.6
0.4
0.2
chr6:74458737
−log10(p−value)
Recombination rate (cM/Mb)
0
2
4
6
8
10
0
20
40
60
80
100
KHDC1
DDX43
EEF1A1
LOC101928489
LOC101928516
C6orf147
MB21D1
SLC17A5
CD109
DPPA5
MTO1
2 genes omitted
74
74.2
74.4
74.6
74.8
Position on chr6 (Mb)

date:	Wed Aug 1 12:40:07 2018
build:	hg19
display range:	chr6:73958737−74958737 [73958737−74958737]
hilite range:	0 − 0 [0 − 0]
reference SNP:	chr6:74458737
number of SNPs plotted:	5212
min P−value:	4.61E−8 [chr6:74458737]
max P−value:	10E−1 [chr6:74346701]
omitted Genes:	KHDC3L, OOEP

Make more plots at http://csg.sph.umich.edu/locuszoom/
