# Supplementary material for: Bone mineral density loci specific to the skull portray potential pleiotropic effects on craniosynostosis
Source: Commun Biol. 2023 Jul 4;6:691. doi: 10.1038/s42003-023-04869-0 (PMC10319806; doi:10.1038/s42003-023-04869-0)
Supplement: Supplementary file 6 — Supplementary Data 3 [file 42003_2023_4869_MOESM6_ESM.zip › loci/chr7_120373438-121373438.pdf]

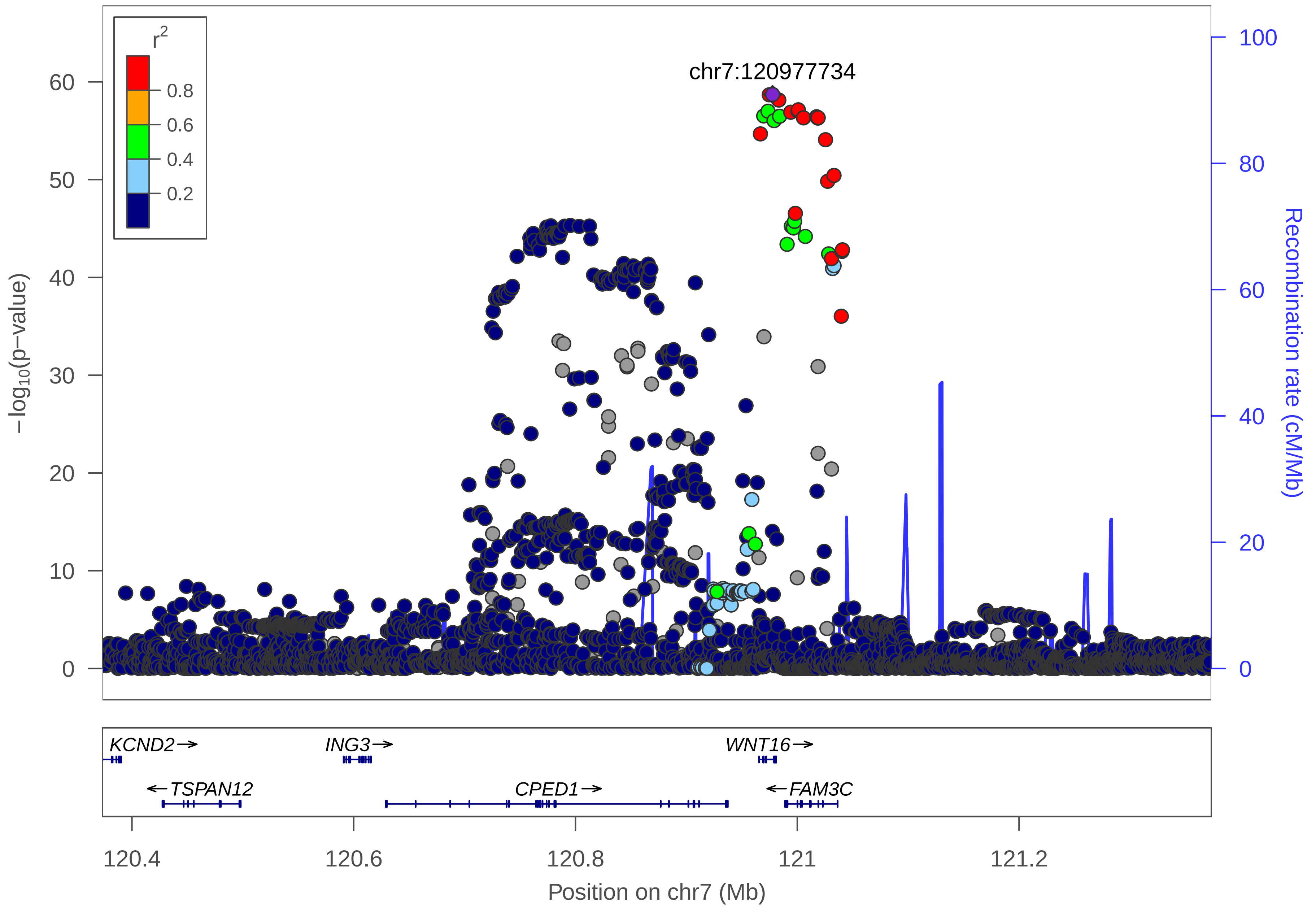

r2
0.8
0.6
0.4
0.2
chr7:120977734
−log10(p−value)
60
50
40
30
20
10
0
100
80
60
40
20
0
Recombination rate (cM/Mb)
KCND2→
ING3→
WNT16→
←TSPAN12
CPED1→
←FAM3C
120.4
120.6
120.8
121
121.2
Position on chr7 (Mb)

date:	Wed Aug 1 12:46:03 2018
build:	hg19
display range:	chr7:120373438−121373438 [120373438−121373438]
hilite range:	0 − 0 [0 − 0]
reference SNP:	chr7:120977734
number of SNPs plotted:	4031
min P−value:	1.95E−59 [chr7:120977734]
max P−value:	10E−1 [chr7:121012772]

Make more plots at http://csg.sph.umich.edu/locuszoom/
