# Supplementary material for: Bone mineral density loci specific to the skull portray potential pleiotropic effects on craniosynostosis
Source: Commun Biol. 2023 Jul 4;6:691. doi: 10.1038/s42003-023-04869-0 (PMC10319806; doi:10.1038/s42003-023-04869-0)
Supplement: Supplementary file 6 — Supplementary Data 3 [file 42003_2023_4869_MOESM6_ESM.zip › loci/chr7_95621636-96621636.pdf]

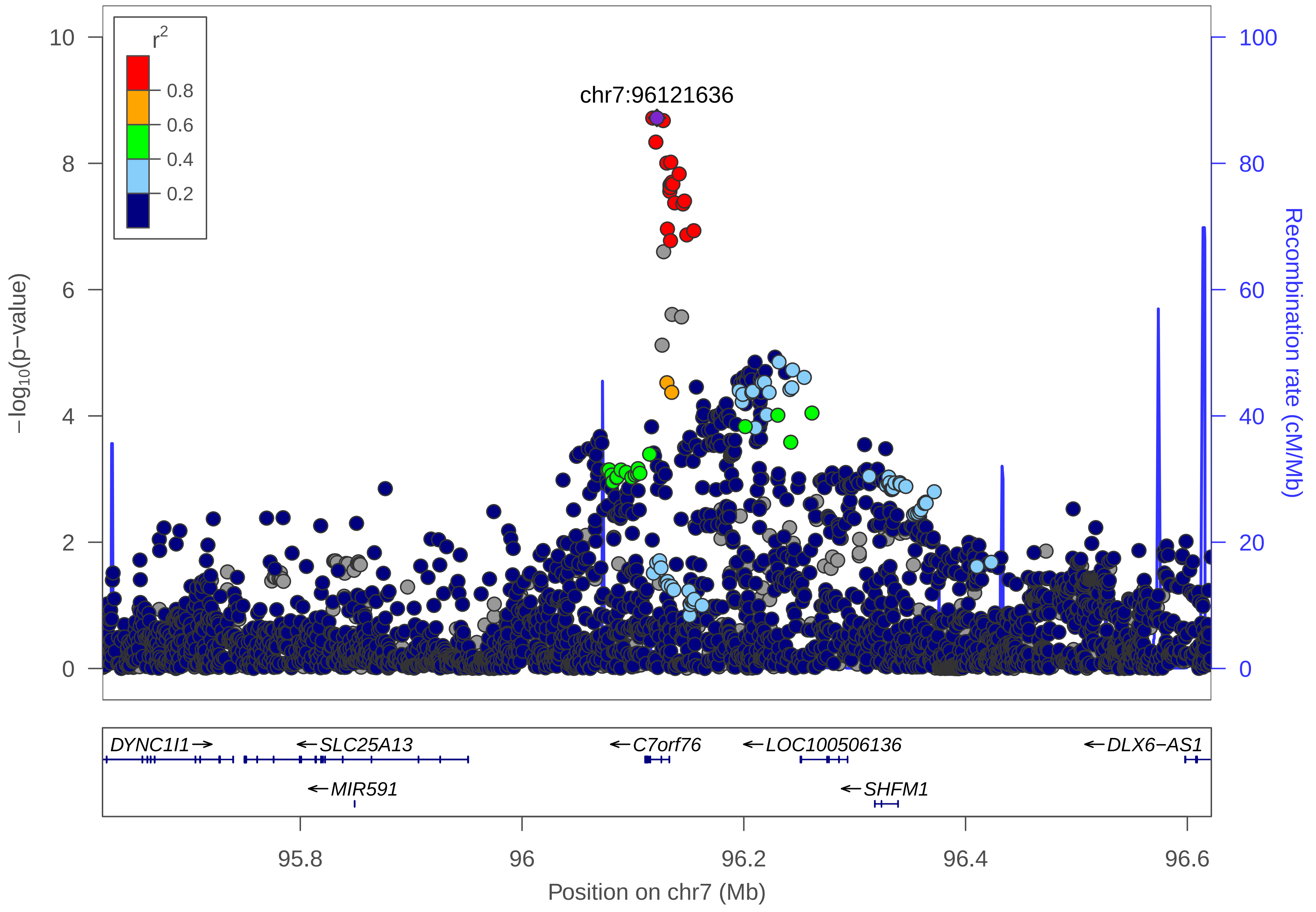

r2
0.8
0.6
0.4
0.2
chr7:96121636
10
8
6
4
2
0
−log10(p−value)
100
80
60
40
20
0
Recombination rate (cM/Mb)
DYNC1I1→
←SLC25A13
←MIR591
←C7orf76
←LOC100506136
←SHFM1
←DLX6−AS1
95.8
96
96.2
96.4
96.6
Position on chr7 (Mb)

date:	Wed Aug 1 12:44:51 2018
build:	hg19
display range:	chr7:95621636−96621636 [95621636−96621636]
hilite range:	0 − 0 [0 − 0]
reference SNP:	chr7:96121636
number of SNPs plotted:	3404
min P−value:	1.91E−9 [chr7:96121636]
max P−value:	1E0 [chr7:96393746]

Make more plots at http://csg.sph.umich.edu/locuszoom/
