# Supplementary material for: Bone mineral density loci specific to the skull portray potential pleiotropic effects on craniosynostosis
Source: Commun Biol. 2023 Jul 4;6:691. doi: 10.1038/s42003-023-04869-0 (PMC10319806; doi:10.1038/s42003-023-04869-0)
Supplement: Supplementary file 6 — Supplementary Data 3 [file 42003_2023_4869_MOESM6_ESM.zip › loci/chr8_103419090-104419090.pdf]

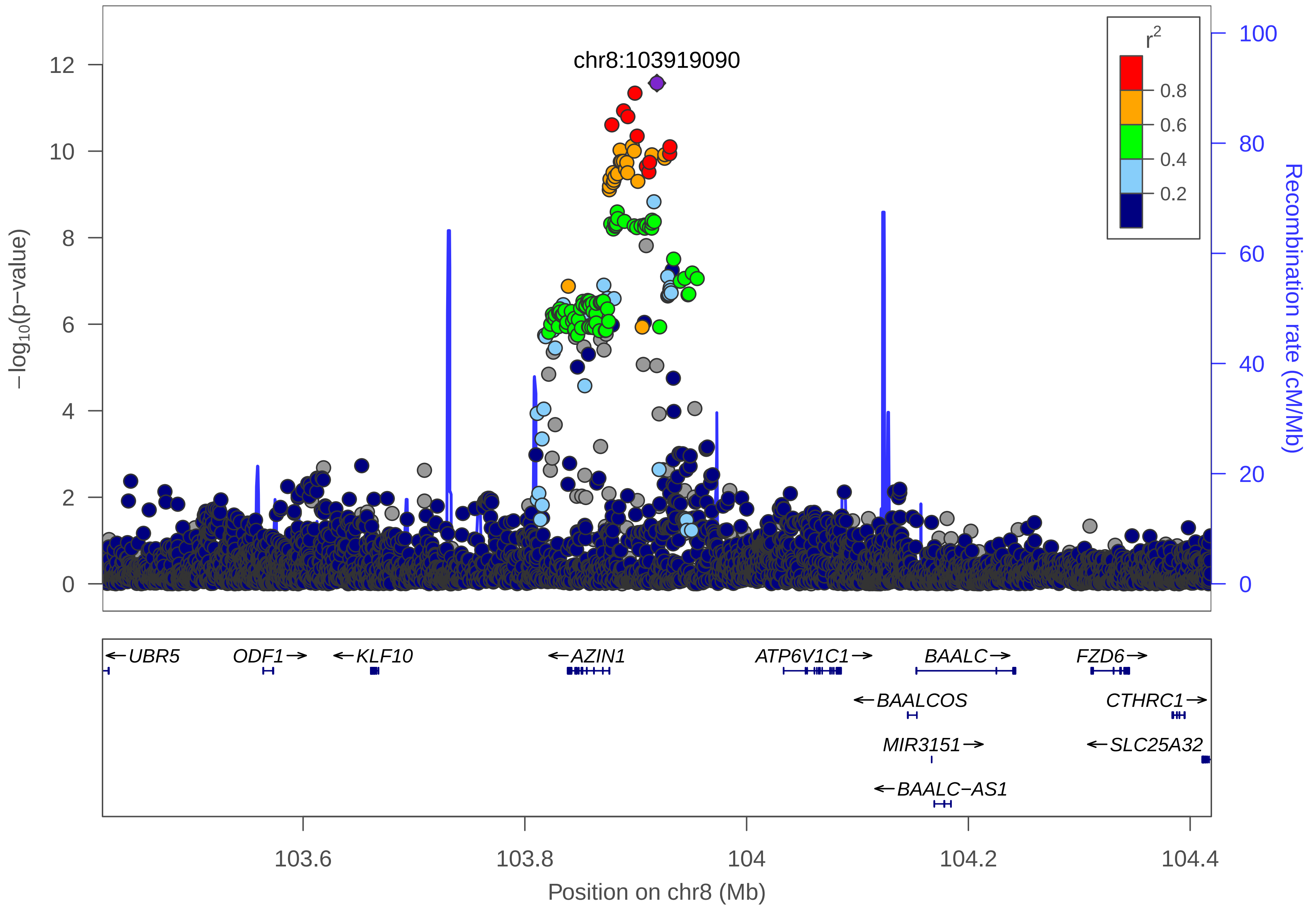
chr8:103919090
r2
0.8
0.6
0.4
0.2
−log10(p−value)
Recombination rate (cM/Mb)
0
2
4
6
8
10
12
0
20
40
60
80
100
UBR5
ODF1
KLF10
AZIN1
ATP6V1C1
BAALC
FZD6
BAALCOS
CTHRC1
MIR3151
SLC25A32
BAALC−AS1
103.6
103.8
104
104.2
104.4
Position on chr8 (Mb)

date:	Wed Aug 1 12:47:51 2018
build:	hg19
display range:	chr8:103419090−104419090 [103419090−104419090]
hilite range:	0 − 0 [0 − 0]
reference SNP:	chr8:103919090
number of SNPs plotted:	4665
min P−value:	2.67E−12 [chr8:103919090]
max P−value:	10E−1 [chr8:103732916]

Make more plots at http://csg.sph.umich.edu/locuszoom/
