# Supplementary material for: Bone mineral density loci specific to the skull portray potential pleiotropic effects on craniosynostosis
Source: Commun Biol. 2023 Jul 4;6:691. doi: 10.1038/s42003-023-04869-0 (PMC10319806; doi:10.1038/s42003-023-04869-0)
Supplement: Supplementary file 6 — Supplementary Data 3 [file 42003_2023_4869_MOESM6_ESM.zip › loci/chr8_108896911-109896911.pdf]

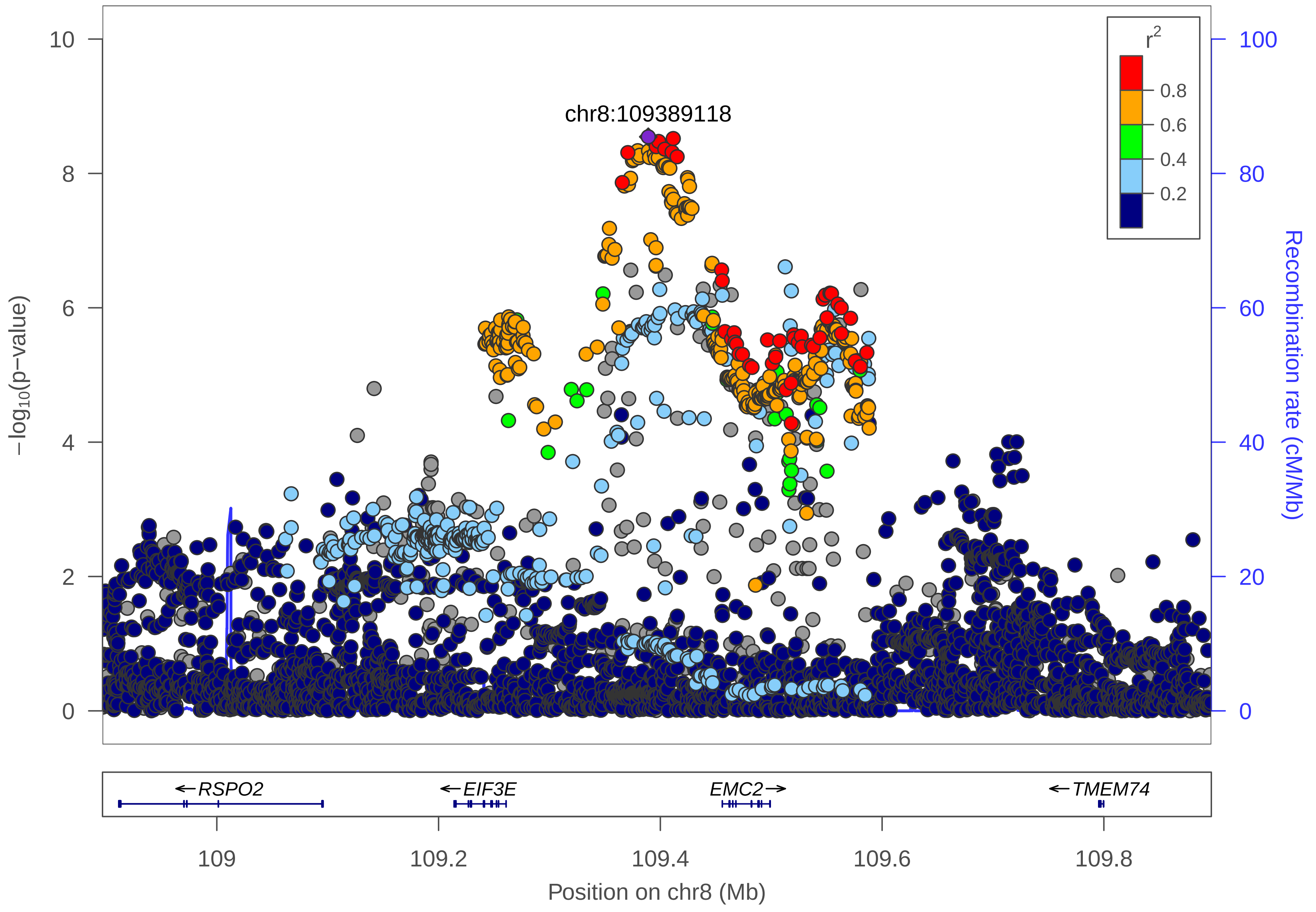

chr8:109389118
r^2
0.8
0.6
0.4
0.2
−log10(p−value)
Recombination rate (cM/Mb)
0
2
4
6
8
10
0
20
40
60
80
100
←RSPO2
←EIF3E
EMC2→
←TMEM74
109
109.2
109.4
109.6
109.8
Position on chr8 (Mb)

date:	Wed Aug 1 12:48:26 2018
build:	hg19
display range:	chr8:108896911−109896911 [108896911−109896911]
hilite range:	0 − 0 [0 − 0]
reference SNP:	chr8:109389118
number of SNPs plotted:	4088
min P−value:	2.85E−9 [chr8:109389118]
max P−value:	10E−1 [chr8:109839486]

Make more plots at http://csg.sph.umich.edu/locuszoom/
