# Supplementary material for: Bone mineral density loci specific to the skull portray potential pleiotropic effects on craniosynostosis
Source: Commun Biol. 2023 Jul 4;6:691. doi: 10.1038/s42003-023-04869-0 (PMC10319806; doi:10.1038/s42003-023-04869-0)
Supplement: Supplementary file 6 — Supplementary Data 3 [file 42003_2023_4869_MOESM6_ESM.zip › loci/chr8_119508587-120508587.pdf]

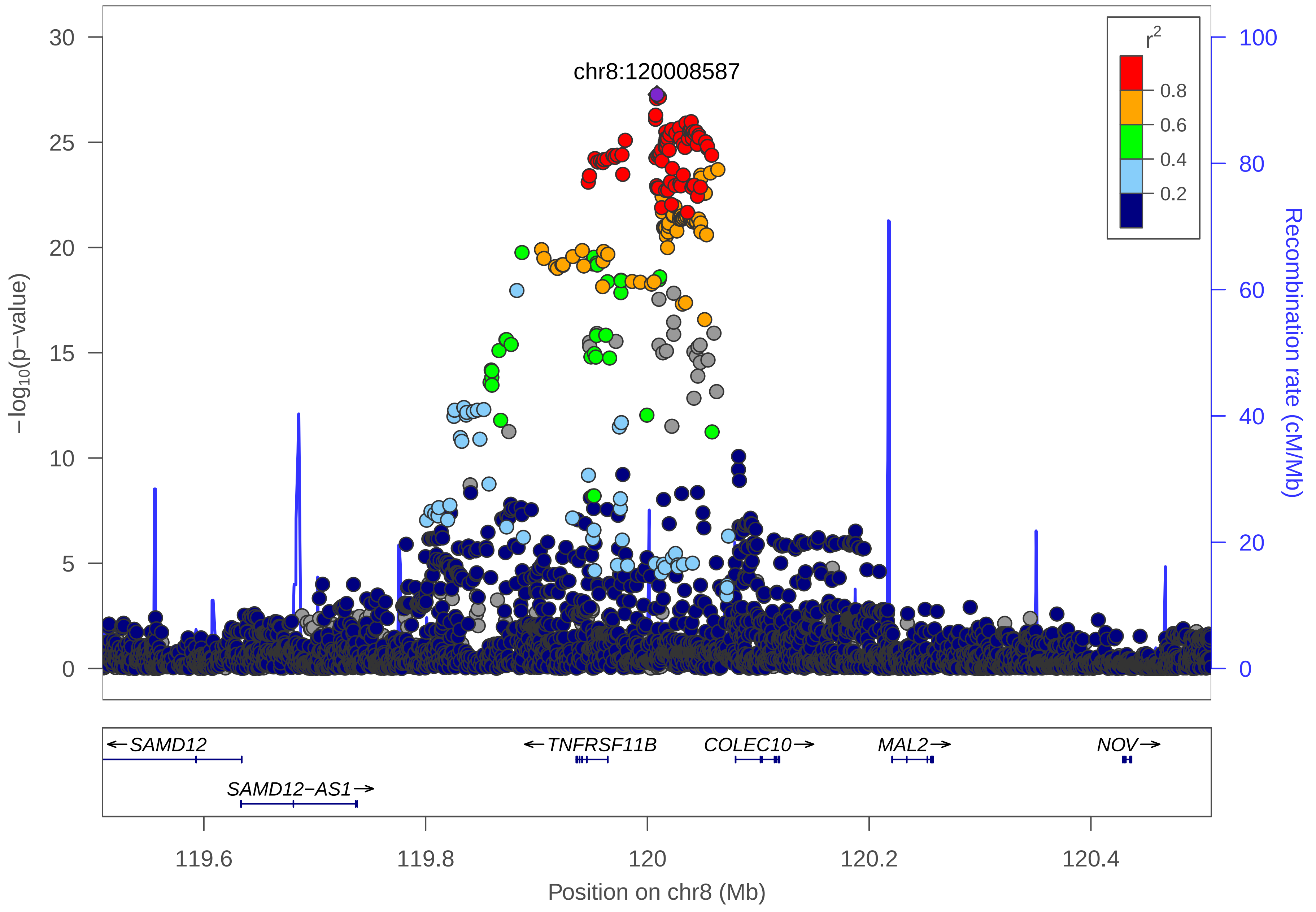

chr8:120008587
r2
0.8
0.6
0.4
0.2
30
25
20
15
10
5
0
−log10(p−value)
100
80
60
40
20
0
Recombination rate (cM/Mb)
SAMD12
SAMD12−AS1
TNFRSF11B
COLEC10
MAL2
NOV
119.6
119.8
120
120.2
120.4
Position on chr8 (Mb)

date:	Wed Aug 1 12:49:02 2018
build:	hg19
display range:	chr8:119508587−120508587 [119508587−120508587]
hilite range:	0 − 0 [0 − 0]
reference SNP:	chr8:120008587
number of SNPs plotted:	4271
min P−value:	5.36E−28 [chr8:120008587]
max P−value:	10E−1 [chr8:119537806]

Make more plots at http://csg.sph.umich.edu/locuszoom/
