# Supplementary figures and images for: Bone mineral density loci specific to the skull portray potential pleiotropic effects on craniosynostosis
Source: Commun Biol. 2023 Jul 4;6:691. doi: 10.1038/s42003-023-04869-0 (PMC10319806; doi:10.1038/s42003-023-04869-0)

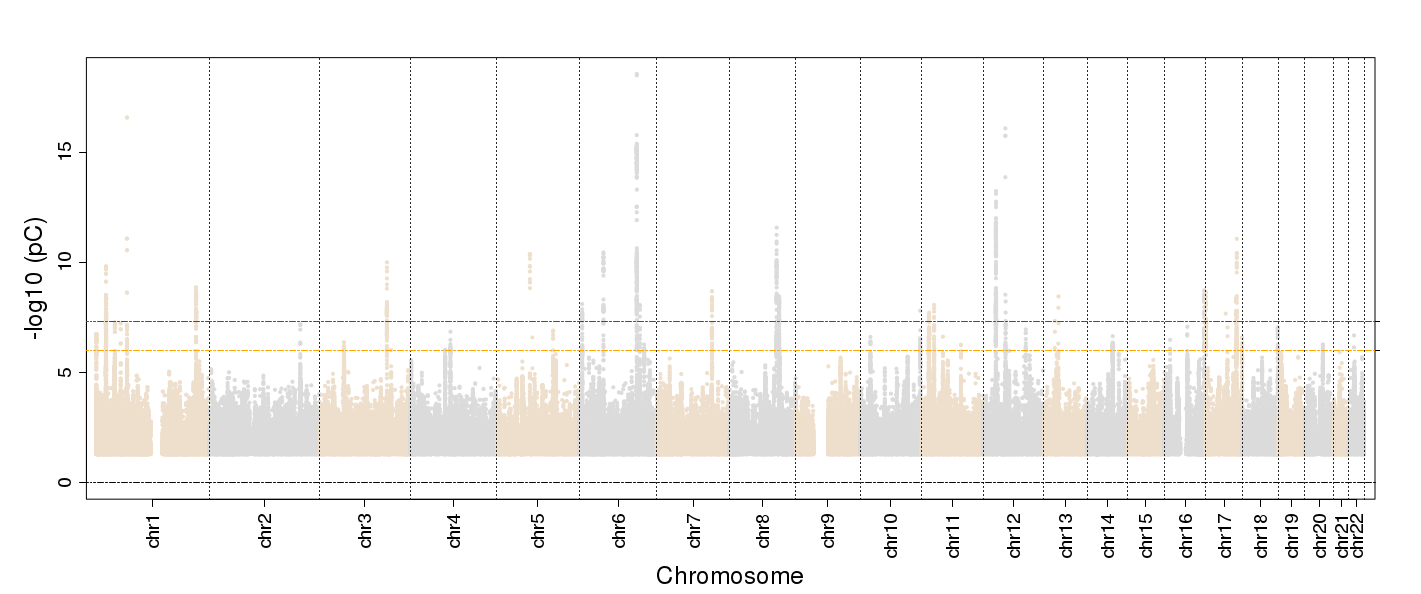

Supplement: Supplementary file 6 — Supplementary Data 3 [file 42003_2023_4869_MOESM6_ESM.zip › loci/condindependent.txt.mh.png]

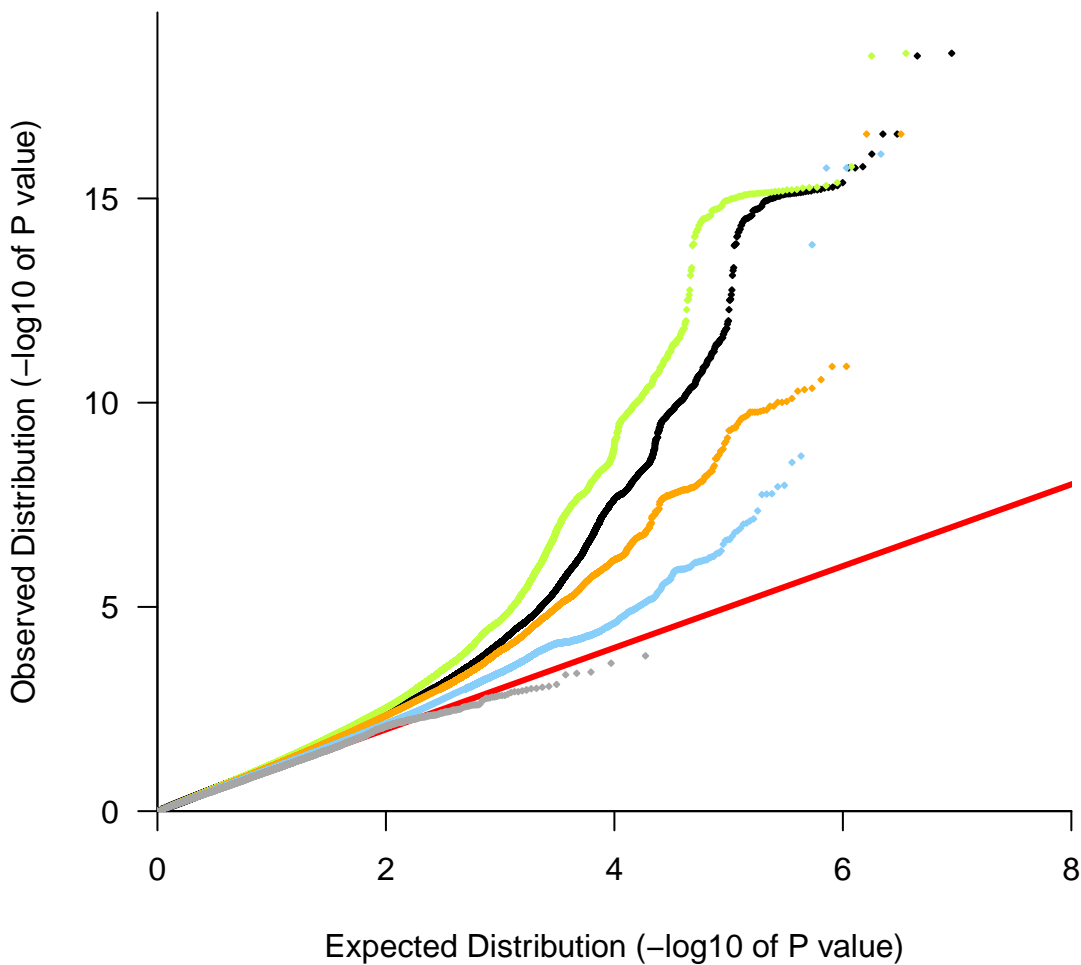

Supplement: Supplementary file 6 — Supplementary Data 3 [file 42003_2023_4869_MOESM6_ESM.zip › loci/META10002016new2cewqq.pdf]

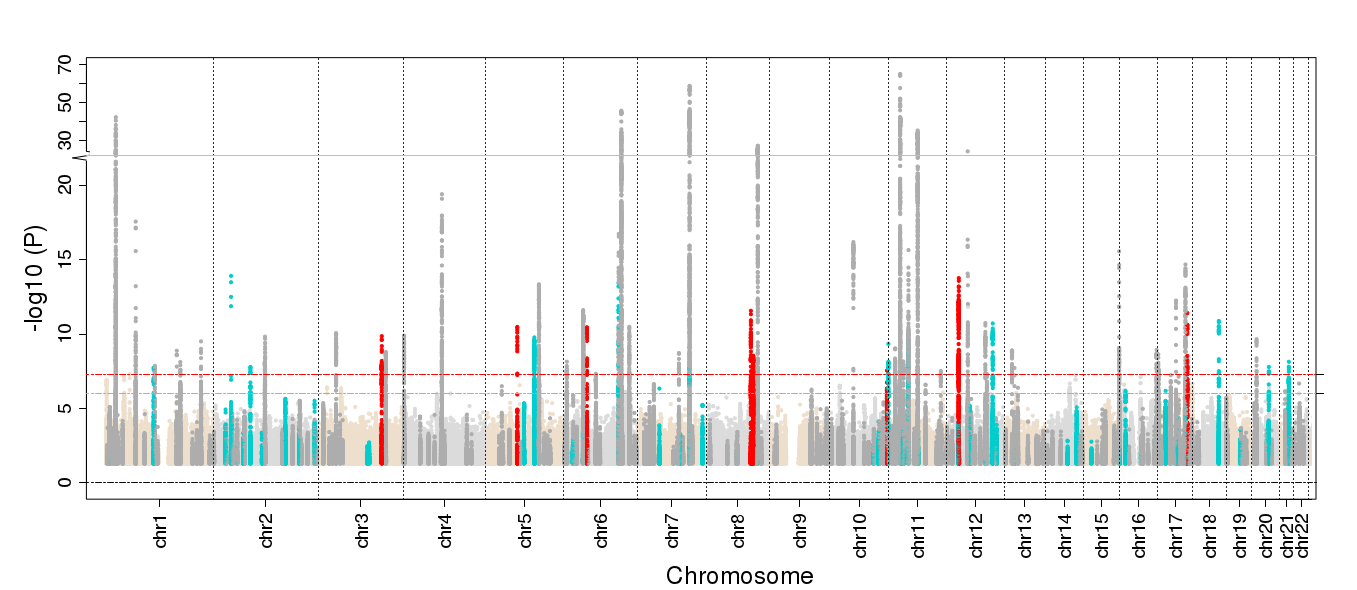

Supplement: Supplementary file 6 — Supplementary Data 3 [file 42003_2023_4869_MOESM6_ESM.zip › loci/try.txt.mh3.png]
