# Supplementary material for: Bone mineral density loci specific to the skull portray potential pleiotropic effects on craniosynostosis
Source: Commun Biol. 2023 Jul 4;6:691. doi: 10.1038/s42003-023-04869-0 (PMC10319806; doi:10.1038/s42003-023-04869-0)
Supplement: Supplementary file 11 — Reporting Summary [file 42003_2023_4869_MOESM11_ESM.pdf]

nature portfolio

Corresponding author(s): Carolina Medina-Gomez

Last updated by author(s): Mar 15, 2023

Reporting Summary

Nature Portfolio wishes to improve the reproducibility of the work that we publish. This form provides structure for consistency and transparency in reporting. For further information on Nature Portfolio policies, see our Editorial Policies and the Editorial Policy Checklist.

Statistics

For all statistical analyses, confirm that the following items are present in the figure legend, table legend, main text, or Methods section.

n/a	Confirmed	
☐	☒	The exact sample size (*n*) for each experimental group/condition, given as a discrete number and unit of measurement
☐	☒	A statement on whether measurements were taken from distinct samples or whether the same sample was measured repeatedly
☐	☒	The statistical test(s) used AND whether they are one- or two-sided<br>*Only common tests should be described solely by name; describe more complex techniques in the Methods section.*
☐	☒	A description of all covariates tested
☐	☒	A description of any assumptions or corrections, such as tests of normality and adjustment for multiple comparisons
☐	☒	A full description of the statistical parameters including central tendency (e.g. means) or other basic estimates (e.g. regression coefficient) AND variation (e.g. standard deviation) or associated estimates of uncertainty (e.g. confidence intervals)
☐	☒	For null hypothesis testing, the test statistic (e.g. *F*, *t*, *r*) with confidence intervals, effect sizes, degrees of freedom and *P* value noted<br>*Give P values as exact values whenever suitable.*
☒	☐	For Bayesian analysis, information on the choice of priors and Markov chain Monte Carlo settings
☒	☐	For hierarchical and complex designs, identification of the appropriate level for tests and full reporting of outcomes
☒	☐	Estimates of effect sizes (e.g. Cohen's *d*, Pearson's *r*), indicating how they were calculated

Our web collection on statistics for biologists contains articles on many of the points above.

Software and code

Policy information about availability of computer code

Data collection	Bone mineral Density was assessed by two types of DXA machines, namely Hologic and Lunar and analyzed by the developers software. Exact information on the machine and sowtware used can be found in the supplementary table 1
Data analysis	GWAS analysis was perform by different software chosen by each participating research center (see Supplementary table 1). Meta-analysis was run in METAL using and inverse-variance scheme. Software used in post-gwas analysis at every subsection of the manuscript has been fully described in the methods of the paper.

For manuscripts utilizing custom algorithms or software that are central to the research but not yet described in published literature, software must be made available to editors and reviewers. We strongly encourage code deposition in a community repository (e.g. GitHub). See the Nature Portfolio guidelines for submitting code & software for further information.

Data

Policy information about availability of data

All manuscripts must include a data availability statement. This statement should provide the following information, where applicable:

- Accession codes, unique identifiers, or web links for publicly available datasets
- A description of any restrictions on data availability
- For clinical datasets or third party data, please ensure that the statement adheres to our policy

Significant results of the GWAS meta-analysis generated during this study are included in the supplementary tables of this manuscript. Full-GWAS summary data is

available in the GEFOS website (www.gefos.org). In the same website readers will have access to the Osteoclast eQTL data. Data regarding MSC-derived osteoblasts can be accessed in ArrayExpress (https://www.ebi.ac.uk/biostudies/arrayexpress/studies) with the following accession numbers: Capture C: "E-MTAB-6862" and ATAC-seq: "E-MTAB-6834" (MSC-derived osteoblasts). T hrough the NCBI-GEO readers can have access to both, RNA expression of human mesenchymal stem cells data can be accessed (accession number GSE80614) and gene expression in calvarial osteoblasts from neonatal C57BL/6J (accession number GSE54461). The RNA-Seq data of the primary bone tissue are publicly available at SRA PRJNA764663

Human research participants

Policy information about studies involving human research participants and Sex and Gender in Research.

Reporting on sex and gender	Sex was used as a covariate in the GWAS-analyses but not sex-specific meta-analyses were carried out, also there was not inclusion of sex-chromosomes in the GWAS
Population characteristics	The description of participants of each of the participating cohorts can be found in Supplementary Table 2 of the manuscript
Recruitment	Description of the participant studies can be found in the Supplementary Information pages 2-8
Ethics oversight	The ethics statemnts of the participant cohorts can be found in the "Cohort short description" section of the Supplementary Information.

Note that full information on the approval of the study protocol must also be provided in the manuscript.

Field-specific reporting

Please select the one below that is the best fit for your research. If you are not sure, read the appropriate sections before making your selection.

☒ Life sciences ☐ Behavioural & social sciences ☐ Ecological, evolutionary & environmental sciences

For a reference copy of the document with all sections, see nature.com/documents/nr-reporting-summary-flat.pdf

Life sciences study design

All studies must disclose on these points even when the disclosure is negative.

Sample size	The total sample size of our meta-analysis is 43,800 individuals
Data exclusions	There were not specific criteria of exclusion of participants. However, given the complexity of GWAS analyses some research centers might have opted for exclusions based on health status, relatedness or ethnicity within their cohorts. List of exclusions can be found in the cohort short description and Supplementary Table 1.
Replication	Our meta-analysis intended to acquired the largest statistical power in the discovery phase, therefore the 21 participant studies were included at this phase.
Randomization	NA
Blinding	NA

Reporting for specific materials, systems and methods

We require information from authors about some types of materials, experimental systems and methods used in many studies. Here, indicate whether each material, system or method listed is relevant to your study. If you are not sure if a list item applies to your research, read the appropriate section before selecting a response.

Materials & experimental systems

n/a	Involved in the study	
☒	☐	Antibodies
☐	☒	Eukaryotic cell lines
☒	☐	Palaeontology and archaeology
☐	☒	Animals and other organisms
☒	☐	Clinical data
☒	☐	Dual use research of concern

Methods

n/a	Involved in the study	
☒	☐	ChIP-seq
☒	☐	Flow cytometry
☒	☐	MRI-based neuroimaging

Eukaryotic cell lines

Policy information about cell lines and Sex and Gender in Research

Cell line source(s)	human Mesenchymal Stromal Cells (hMSCs): Lonza Group Ltd., Basel, Switzerland from healthy male donors; Peripheral Blood Mononuclear Cells (PBMCs): Following the protocol detailed in ref. 65 from females.
Authentication	hMSCs: Cells were authenticated at Lonza Group Ltd. by FACS analyses for the presence of surface markers CD105, CD166, CD29 and CD44 and the absence of CD14, CD34 and CD45. PBMCs: using a CD14 antibody-conjugated magnetic bead system (Miltenyi Biotec, Bergisch Gladbach, Germany) and stained for tartrate-resistant acid phosphatase (TRAP).
Mycoplasma contamination	The hMSCs were tested negative for mycoplasma, both by the company and in-house during the culture experiments described in this manuscript. PBMC-derived osteoclasts, in-house during the culture experiments described in this manuscript
Commonly misidentified lines (See ICLAC register)	NA

Animals and other research organisms

Policy information about studies involving animals; ARRIVE guidelines recommended for reporting animal research, and Sex and Gender in Research

Laboratory animals	Danio rerio
Wild animals	*Provide details on animals observed in or captured in the field; report species and age where possible. Describe how animals were caught and transported and what happened to captive animals after the study (if killed, explain why and describe method; if released, say where and when) OR state that the study did not involve wild animals.*
Reporting on sex	Zebrafish were analysed before sexual maturation, therefore sex did not affect the results.
Field-collected samples	*For laboratory work with field-collected samples, describe all relevant parameters such as housing, maintenance, temperature, photoperiod and end-of-experiment protocol OR state that the study did not involve samples collected from the field.*
Ethics oversight	All experiments were performed at the University of Bristol and approved by the UK Home Office.

Note that full information on the approval of the study protocol must also be provided in the manuscript.
