# Supplementary material for: Bone mineral density loci specific to the skull portray potential pleiotropic effects on craniosynostosis
Source: Commun Biol. 2023 Jul 4;6:691. doi: 10.1038/s42003-023-04869-0 (PMC10319806; doi:10.1038/s42003-023-04869-0)
Supplement: Supplementary file 1 — Rivadeneira_Peer Review File [file 42003_2023_4869_MOESM1_ESM.pdf]

nature portfolio

Peer Review File

Bone Mineral Density Loci Specific to the Skull Portray Potential Pleiotropic Effects On Craniosynostosis

Open Access This file is licensed under a Creative Commons Attribution 4.0 International License, which permits use, sharing, adaptation, distribution and reproduction in any medium or format, as long as you give appropriate credit to the original author(s) and the source, provide a link to the Creative Commons license, and indicate if changes were made. In the cases where the authors are anonymous, such as is the case for the reports of anonymous peer reviewers, author attribution should be to 'Anonymous Referee' followed by a clear attribution to the source work. The images or other third party material in this file are included in the article's Creative Commons license, unless indicated otherwise in a credit line to the material. If material is not included in the article's Creative Commons license and your intended use is not permitted by statutory regulation or exceeds the permitted use, you will need to obtain permission directly from the copyright holder. To view a copy of this license, visit http://creativecommons.org/licenses/by/4.0/.

This manuscript has been previously reviewed at another Nature journal.

Reviewers' comments:

Reviewer #1 (Remarks to the Author):

In this study, Carolina Medina-Gomez et al. performed a skull bone mineral density (SK-BMD) genome-wide association meta-analysis and identified 59 loci. They then conducted a series of bioinformatical annotations. At last, the authors generated zebrafish gene knowndown models to evaluate bone related functions for the identified novel genes. Overall, the study is of interest with comprehensive analyses. However, I do have a couple of questions that need the authors to address.

Major comments:
1) Pleiotropic effect. As the authors stated in the title, the identified loci portray pleiotropic effect to both osteoporosis and craniosynostosis. In my opinion, pleiotropic effect means the same variants/loci being associated with both traits. However, throughout the study the authors solely performed a GWAS for SK-BMD, while no any population data or analyses were involved for craniosynostosis. The only involvment was the final zebrafish experiments in which the authors observed abnormal skull development. My suggestion is to either add some genetic data to prove that the identified loci were really associated with craniosynostosis, or to tone down the statement regarding pleiotropic effect in the title.
2) Evidence of the relation between lead variants and plausible causal genes is weak. It is well known that variants identified in GWAS are not necessarily causal, let alone their nearby genes. The authors need to convince the reader that the studied genes are really relevant to the identified loci/variants. For instance, 3q24 was indexed by rs12107945, but there is little evidence (such as eQTL, pQTL or other sources of information) linking rs12107945 to ZIC1 gene. The authors need to prioritize target genes with convincing evidence.
3) Collider bias. The authors corrected for the effect of covariates including age, weight and height. I don't understand why weight and height could have a significant effect on skell BMD. My concern was the potential collider bias caused by the latter two covariates. For the lead SNP rs12107945, it was also significant for sitting height (P=6.12x10-10) and standing height (P=2.56x10-5) in the UKBB cohort through the GeneATLAS web portal. I was wondering if your results changed or not without including weight and height into covariates.
4) Winner's curse effect. The total 12.5% phenotypic variance explained by the 59 loci seems a little bit high. I did not find how you estimated this value. If it was estimated from the same discovery sample, then the Winner's curse effect needs to be corrected.

Minor comments:
1) Title, "pleitropic" missing a letter "o"
2) Line 63, "and" is not necessary.
3) Lines 109-119, too many sub-sections.

Reviewer #2 (Remarks to the Author):

Summary: This is a well-designed and robust study, performing a meta-analysis of GWA studies and identifying 59 loci with skull bone density. Integrating such data with studies with eQTL and GARFIELD analysis, the authors are able to make inferences as to the functionality of a proportion of variants, demonstrating that some are likely functional SNPs found in open chromatin regions and likely regulate gene expression. While most loci had been previously associated with BMD (either whole body, or a specific site such as the hip or spine) the authors identify 4 novel loci previously not associated with BMD. Functional characterization of this loci in zebrafish demonstrate a role in skull development and BMD. This s a strong paper overall, and highlight the power of using skull BMD readings to find further loci that regulate BMD and skull development. Additional experiments could strengthen the functional (zebrafish) portion of the results. Also, the discussion is brief and could be expanded.

Major:

1) For their zebrafish analysis, given that crispants are used (as opposed to fish with a single mutation in all cells) the authors should show the mutation rate in a proportion of the animals analyzed. For example, for the zic1 crispants, only 22% of embryos showed abnormal skull growth and development. Is the mutation rate higher in these animals when compared to the 78% that did not display defects in bone development? For the microCT analysis, while a really nice aspect of the paper, it is not entirely convincing without knowing mutation rates. One would think that high mutation rate would be needed to get such a highly significant result. Overall, the zebrafish data would be more convincing if the mutation rate was calculated ore a subset of the experiments shown, or conversely, demonstration of the phenotype (lower BMD, suture defects) in stable mutants.

While the authors state in their methods that their CRISPR efficiency is 90%, I don't see any data to support this for any individual experiment where phenotyping occurred.

2) The discussion is very brief, and there is little mention of the much of the work that has been done. There is little mention of the gene expression studies, and their role in osteoblast differentiation, or the fact that some causative SNPs have been identified. The discussion ould be expanded to more accurately reflect all of the data presented in the paper.

Minor:

1) Table 1 is a little confusing. I don't not know what is meant by "notes refer to annotation based on the closest gene." Also, there is no description of what the asterisks (by rs61863293) is for, or the number sign (#) in the footnote.

2) Line 25- This sentence, referring to high heritability of SK-BMD, should be supported with a reference.

3) Line 31- Does skull BMD readings include the jaw? If so, then the phrase indicating that skull bone density can unravel gene and pathways important for intramembranous ossification should be reworded as most of the jaw is ossified through endochondral ossification.

4) Line 70-71- a nominally significant correlation was described between skull mineralization and infant head circumference- Is this data for the current study? If so, it should be included and the specific table/figure refereed to in the text.

5) Line 77-Conversely, a uniform pattern for functional signatures was observed in the other six cell lines tested (GM12878, H1HESC, HeLa-S3, HepG2, HUVEC, K562). Is this data shown in the paper?

6) Line 104- the following genes were prioritized……. Prioritized for what?

8)Line 123- ….”compartment. and”- Capital “A” or remove period?

9) Line 155- the authors indicate that one novel locus GLRX3 was not chosen for further study, as it is involved in iron metabolism, and there was little supporting evidence that it regulates bone mineralization. As there is literature demonstrating that iron intake and metabolism is associated with osteoporosis, this statement should be reworded.

10) Line 171- a p-value of 0.066 would generally not be considered significant. This statement should be reworded.

11) Line 183- “With relevant” should be “with relevance?”

12) For gene expression analysis in bone tissue, samples were taken from women with osteoarthritis. This should be discussed, as it is possible the expression of some genes may not occur in healthy tissue or may be sex specific.

Response to reviewers

We would like to thank the reviewers for their comments and remarks which
have been incorporated in this new version of our manuscript to the best of our abilities. In addition, this manuscript version now complies with the editorial policies of Communications Biology. Specific point-by-point responses and changes in our manuscript are described below.

Reviewer #1 (Remarks to the Author):

In this study, Carolina Medina-Gomez et al. performed a skull bone mineral density (SK-BMD) genome-wide association meta-analysis and identified 59 loci. They then conducted a series of bioinformatical annotations. At last, the authors generated zebrafish gene knockdown models to evaluate bone related functions for the identified novel genes. Overall, the study is of interest with comprehensive analyses. However, I do have a couple of questions that need the authors to address.

Major comments:

1) Pleiotropic effect. As the authors stated in the title, the identified loci portray pleiotropic effect to both osteoporosis and craniosynostosis. In my opinion, pleiotropic effect means the same variants/loci being associated with both traits. However, throughout the study the authors solely performed a GWAS for SK-BMD, while no any population data or analyses were involved for craniosynostosis. The only involvement was the final zebrafish experiments in which the authors observed abnormal skull development. My suggestion is to either add some genetic data to prove that the identified loci were really associated with craniosynostosis, or to tone down the statement regarding pleiotropic effect in the title.

As indicated by the reviewer, pleiotropy between skull BMD and craniosynostosis has not been formally tested. While we planned to perform colocalization analysis using the previously published craniosynostosis GWAS[1,2], the summary statistics are not publicly available nor we could obtain them after contacting the authors. Therefore, we have re-worded at every instance "pleiotropic effect" to "potential pleiotropic effects" throughout the manuscript.

2) Evidence of the relation between lead variants and plausible causal genes is weak. It is well known that variants identified in GWAS are not necessarily causal, let alone their nearby genes. The authors need to convince the reader that the studied genes are really relevant to the identified loci/variants. For instance, 3q24 was indexed by rs12107945, but there is little

evidence (such as eQTL, pQTL or other sources of information) linking rs12107945 to ZIC1 gene. The authors need to prioritize target genes with convincing evidence.

We agree with the reviewer that pinpointing the causal gene or variant underlying the GWAS signals is not straightforward. To assess this across the novel loci, we have scrutinized all datasets at hand, focusing on skeletal tissue and bone cells to help us build-up evidence of implication of the genes underlying the association signal. These included: expression profiles from primary bone tissue (fragments), expression profiles in murine and human bone cell lines, chromatin conformation in osteoblasts, eQTLs in osteoclasts and performing literature review for genes annotated in close proximity to the lead variant. Despite these comprehensive assessments, we did not find conclusive evidence of implication for any particular gene. While the use of animal models is a robust way to demonstrate functional implication, experiments take time and are costly, therefore, we limited the follow-up to one gene per locus (see Figure 1 below). Do note that our selection is backed-up by functional annotations. Two of the four novel GWAS signals mapped to genes involved in intramembranous ossification, neural crest development (*PRKAR1A*) and patterning (*ZIC1*). Moreover, *ATP6V1C1* on 8q22.3 was known to be essential in osteoclast-mediated bone resorption. For the last locus, on 10q26.3, the distance between the leading variant and the closest coding gene, *GLRX3,* was over ~2.7 Mb, and as such, without further evidence of implication, no gene in the locus was further investigated.

We have modified the manuscript to make this contention clear, reading in lines 133-136 of the results section Relevance of SK-BMD implicated genes in bone biology: "Notwithstanding the wide range of strategies to identify potential genes underlying the associations at the novel loci (i.e., gene expression, molecular pathways, ATAC-seq lookups, eQTL results, mutational evidence and genomic location), the overlap of the lines of evidence was not conclusive". To the discussion, in lines 233-246 we have added ". We attempted gene prioritization using lines of evidence from *in-silico* datasets (i.e., DEPICT, CADD scores), chromatin conformation in osteoblasts, eQTLs in osteoclasts, expression in murine and human bone cell lines and additional evidence from the literature. However, none of the strategies was effective in conclusively identifying potential genes underlying the association in the four novel loci, and in general, the overlap of these evidence lines was not overwhelming. Therefore, follow-up prioritization was based on the suggested function of the genes mapping to the four new loci. Our zebrafish experiments provided strong genetic evidence of the involvement of prioritized genes in the process of skull mineralization. Yet, we cannot guarantee that there are no additional genes underlying the association at specific loci. The evidence resulting from integrating our GWAS results with chromatin annotations and transcriptomic data was scattered across the distinct types of bone cells, overall showing heterogeneous results. Therefore, we cannot exclude that our GWS signals originate from processes stemming from either cells or tissues that play a role in mineralization, but were not integrated into this study."

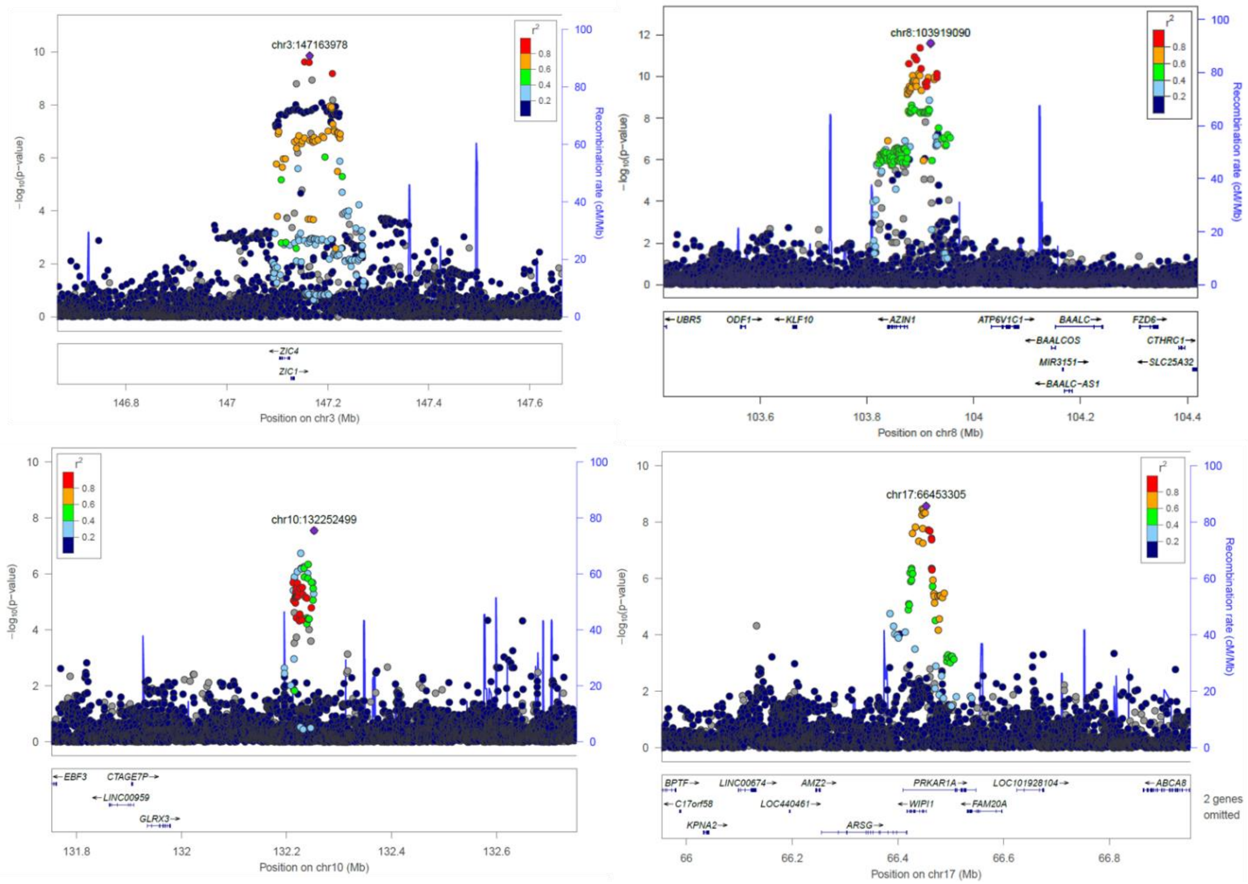

Figure 1. Regional plots for the four novel loci associated with SK-BMD ($P<5x10^{-8}$). Circles show GWAS meta-analysis P-values and position of SNPs for the overall meta-analysis (N=43,800). Different colors indicate varying degrees of pair-wise linkage disequilibrium with the top marker (1000 Genomes – CEU population)

3) Collider bias. The authors corrected for the effect of covariates including age, weight and height. I don't understand why weight and height could have a significant effect on skell BMD. My concern was the potential collider bias caused by the latter two covariates. For the lead SNP rs12107945, it was also significant for sitting height (P=6.12x10-10) and standing height (P=2.56x10-5) in the UKBB cohort through the GeneATLAS web portal. I was wondering if your results changed or not without including weight and height into covariates.

This project was generated hand by hand with the total body BMD project, published in 2018[3], and as such, we used exactly the same statistical model to assess both phenotypes across efforts, so that the approaches would be comparable. We indeed expect notorious less influence of body weight on skull BMD than on total body BMD. Correction for body height can be relevant considering differences in skull area and volume (third dimension artefact on the 2D areal assessment of skull BMD). Either way, we agree with the reviewer that the possibility of a collider effect needs to be considered. To do that, we used data from six participating cohorts (N= 15,080, representing ~35% of the original dataset), where we were able to rerun the skull BMD GWAS

without adjustments for weight and height within reasonable time. Briefly, we selected the data of the 78 independent SNPs identified in the COJO analysis from our manuscript, so both primary and secondary signals are reported, and compared the summary statistics of both models -with and without correction of height and weight- (**see Appendix 1**). Overall, taking the results for these SNPs, we did not observe hits ($P<5x10^{-8}$) appearing only in one model and not in the other, neither did we observe change in effect direction across SNPs after adjustment for height and weight. Yet, eleven out of these 78 SNPs showed a change in effect size larger than 10% between the unadjusted and the adjusted models. However, considering the confidence intervals of the effect sizes, differences were not significant (**see Figure 2**). Rs12107945, the SNP mentioned by the reviewer, showed a difference of only 2% in effect size after the adjustment for height and weight ($beta_{unadj}$=-0.0623, SE_{unadj}=0.0128; $beta_{adj}$=-0.0611, SE_{adj}=0.0128).

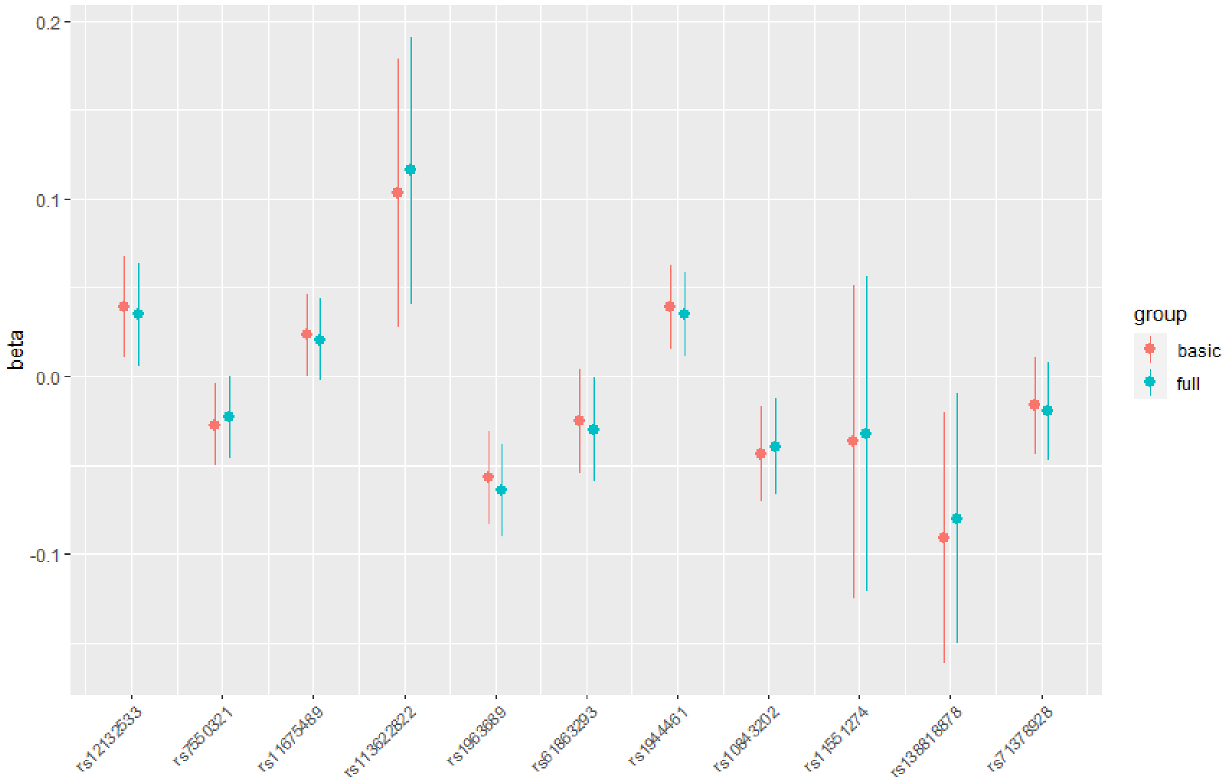

Figure 2. Comparison of effect sizes for SK-BMD GWAS using different modeling approaches. Effect sizes and 95% confidence interval for eleven SNPs with 10% change in effect size across models. Pink: SK-BMD GWAS results after adjustment for age, sex and principal components. Cyan: SK-BMD GWAS results after adjustment for age, sex, weight, height and principal components

To acknowledge the possibility of collider bias, we have added a paragraph regarding this possibility in the discussion section of our manuscript [lines 219-225]. “Traditionally, BMD-related GWAS[1,2,6,13] have adjusted for other heritable covariates such as weight, and height -in the case of pediatric cohorts- given the impact of these phenotypes either in bone mass or its

measurement. Yet, these adjustments could introduce collider bias[43] and risk of false positives. However, BMD loci unveiled by other GWAS[1,2,6,13] and by our current study have been robustly replicated in the ultrasound effort ran in the UKBB[4], which did not adjust for heritable covariates, making less likely the presence of false positives due to a potential collider effect."

4) Winner's curse effect. The total 12.5% phenotypic variance explained by the 59 loci seems a little bit high. I did not find how you estimated this value. If it was estimated from the same discovery sample, then the Winner's curse effect needs to be corrected.

We thank the reviewer for noticing our omission in describing the methodology to estimate the explained variance. As our betas are in SD units, we calculated the variance explained per SNP using the formula of the product of the effect size squared and the heterozygosity. Next, summed this variance for the 78 COJO independent SNPs

Var(y) =2*MAF*(1-MAF)[4]

This formula is commonly used in GWAS meta-analysis[3,5-11]. We have added the information for this calculation to the methods section (lines 327-331)

"The genetic variance explained by each SNP was calculated for each independently associated variant, using the formula $\sigma^2_{snp}=2\beta^2 x MAF x(1-MAF)$[54], where β represents the effect size per SNP in SD units and MAF is the minor allele frequency per variant. The total variance explained by GWS variants corresponds to the sum across all SNPs."

<u>Minor comments:</u>

1)Title,"pleitropic" missing a letter "o"

We have corrected this typo

2) Line 63, "and" is not necessary

We have corrected this typo

3) Lines 109-119, too many sub-sections.

We have modified this.

Reviewer #2 (Remarks to the Author):

Summary: This is a well-designed and robust study, performing a meta-analysis of GWA studies and identifying 59 loci with skull bone density. Integrating such data with studies with eQTL and GARFIELD analysis, the authors are able to make inferences as to the functionality of a proportion of variants, demonstrating that some are likely functional SNPs found in open chromatin regions and likely regulate gene expression. While most loci had been previously associated with BMD (either whole body, or a specific site such as the hip or spine) the authors identify 4 novel loci previously not associated with BMD. Functional characterization of this loci in zebrafish demonstrate a role in skull development and BMD. This s a strong paper overall, and highlight the power of using skull BMD readings to find further loci that regulate BMD and skull development. Additional experiments could strengthen the functional (zebrafish) portion of the results. Also, the discussion is brief and could be expanded.

Major comments:

1)For their zebrafish analysis, given that crispants are used (as opposed to fish with a single mutation in all cells) the authors should show the mutation rate in a proportion of the animals analyzed. For example, for the zic1 crispants, only 22% of embryos showed abnormal skull growth and development. Is the mutation rate higher in these animals when compared to the 78% that did not display defects in bone development? For the microCT analysis, while a really nice aspect of the paper, it is not entirely convincing without knowing mutation rates. One would think that high mutation rate would be needed to get such a highly significant result. Overall, the zebrafish data would be more convincing if the mutation rate was calculated ore a subset of the experiments shown, or conversely, demonstration of the phenotype (lower BMD, suture defects) in stable mutants.

While the authors state in their methods that their CRISPR efficiency is 90%, I don't see any data to support this for any individual experiment where phenotyping occurred.

We thank the reviewer for the appreciation of our work and constructive comments to improve the manuscript. We performed additional zebra fish experiments and phenotyping thereof. We added the relevant information to the methods (lines 469-478) and results sections (lines 184-195) of the manuscript. In addition, the results of the newly implemented analysis are displayed in Figures 7 and 8 of the supplementary material and also shown below, labeled as Figures 3 and 4.

Briefly, fragment length analysis was implemented to determine the CRISPR efficiency based on the CRISPR-STAT method (Somatic Tissue Activity Test)[4]. Our CRISPR system reached 90% indel efficiency (multiple peaks identified in at least 10/12 larvae analyzed). To determine the mutation rate, we used the average height of the *wt* peaks between uninjected and injected pools of fish[4].

Our experiments reached a minimum fold change of 5.6, indicating that somatic mutations are observed in ~6 times more cells than the *wt* allele.

As a proof-of-principle, we extended our experiments to generate *zic1* homozygous mutants. Our results show the association between abnormal suture and biallelic somatic mutations, recapitulating homozygous phenotype. Previous findings in Medaka, showed that Da homozygous mutants have double anal fin and asymmetrical caudal fin skeleton[12]. Medaka Da mutants have a transposon insertion in a regulatory region of *zic1* completely abolishing *zic1* expression. In our experiments with *zic1* crispants, we detected asymmetrical caudal fin phenotype in 63% (45/71) of injected fish analysed at 6 weeks. In other words, the biallelic mutation phenotype recapitulating complete loss of expression of *zic1* was present in 63% of the crispants. However, only a subset of fish with abnormal fins also showed the abnormal suture patterning phenotype. To make sure that the abnormal suture patterning phenotype was associated with biallelic mutations, we raised *zic1* crispants to adulthood and crossed them with *wt* to generate F1 (**Supplementary Figure 7**). From F1, we selected heterozygous mutants that were genotyped using Sanger sequencing followed by CRISPR-ID analyses[13]. One male and one female carrying the same type of mutation (5bp deletion) were crossed. Fifty-five F2 (2-month-old) were phenotypically assessed using live alizarin red staining. Asymmetrical caudal fin phenotype was detected in 13/55 (24%) of fish (**Supplementary Figure 8**). While a skull phenotype was not detected in any normal-fin zebrafish, all those with asymmetrical fins showed irregular cranial bone growth. Yet, ectopic sutures were detected in only 2 fish with abnormal fins. Therefore, our results from these *zic1* experiments showed that the suture phenotype is associated with a homozygous mutation of this known craniosynostosis gene; however, an ectopic suture is not always observed in homozygous fish. Our findings are similar to what has been shown for other mutants with an ectopic suture phenotype, such as in *sp7-/-*[14] whose ectopic sutures are extremely variable in number and position, and in *fgfr3-/-*[15] and *twist1* mutants[16] that showed ectopic sutures in only a small subset of mutants.

Because we performed uCT scanning on pre-selected crispants showing skull phenotype (ectopic suture or irregular bone growth), we believe our BMD results are robust.

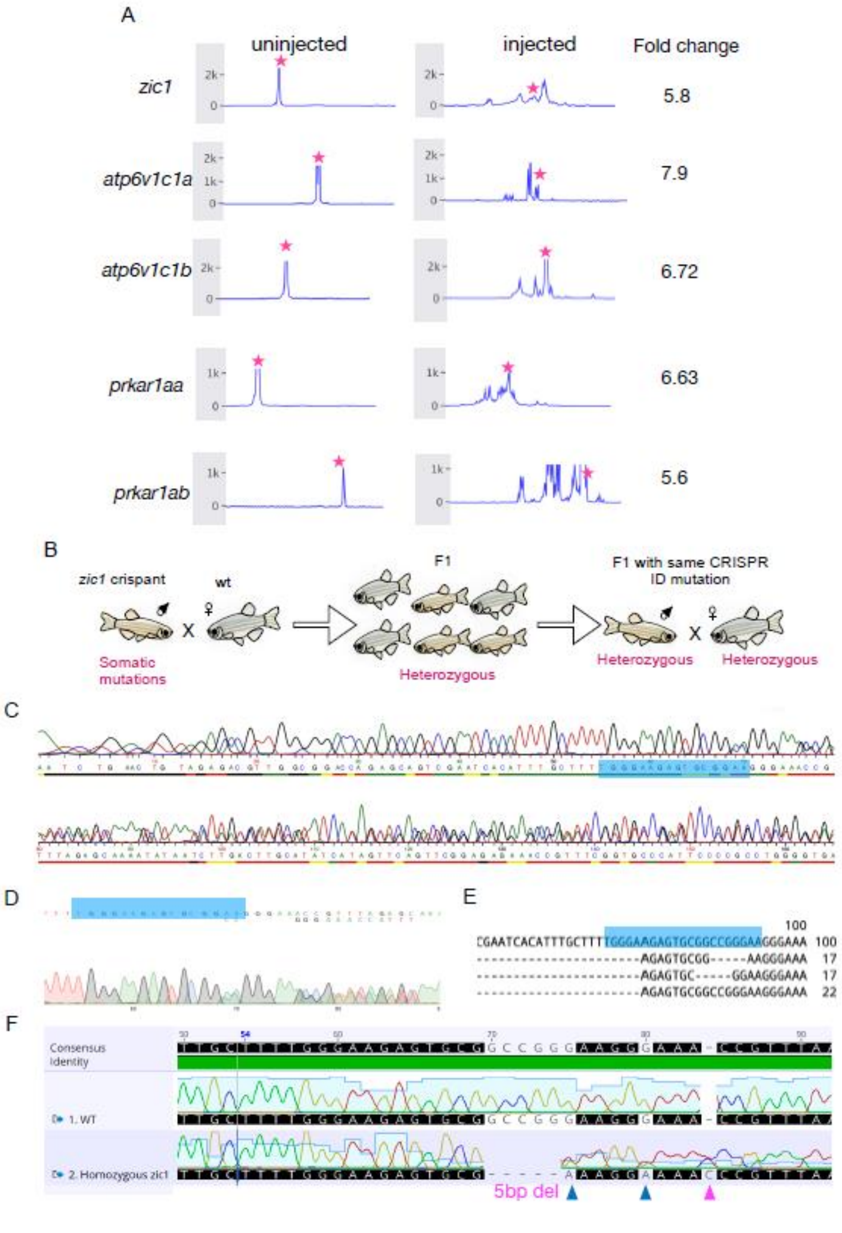

Figure 3. A) Evaluation of CRISPR efficiency. Amplification peak for uninjected and CRISPR injected pools (n=8) are shown for each of the genes. Red stars show the WT peak. Fold change was calculated dividing the WT peak between uninjected and injected pools. **B) Generation *zic1* homozygous mutants**. *zic1* crispants were crossed to wt to generate F1 (carrying different types of mutations in heterozygosity). F1 was genotyped using Sanger sequencing analysed using CRISPR-ID. A pair of F1 carrying the same mutation were crossed to generate F2. **C) Sanger sequencing of F1 fish**. Blue square highlights a region target by 2 gRNAS. **D and E) CRISPR ID analysis** showing the alignment between a reference control and F1. A pair of fish with the same mutation was crossed to generate *zic1* homozygous mutants (F2). **E) CRISPR-ID analysis of one F1 sequence** (fish with asymmetrical caudal fin), predicting 5bp deletions. F) Sequence alignment (Geneious) of a wt and a *zic1* homozygous mutant. Note the confirmation of a deletion of 5bp, two mismatches (blue arrowheads) and an insertion of a base pair (magenta arrowhead). These mutations lead to truncation of the *zic1* protein, reducing protein length from 442aa to 283aa.

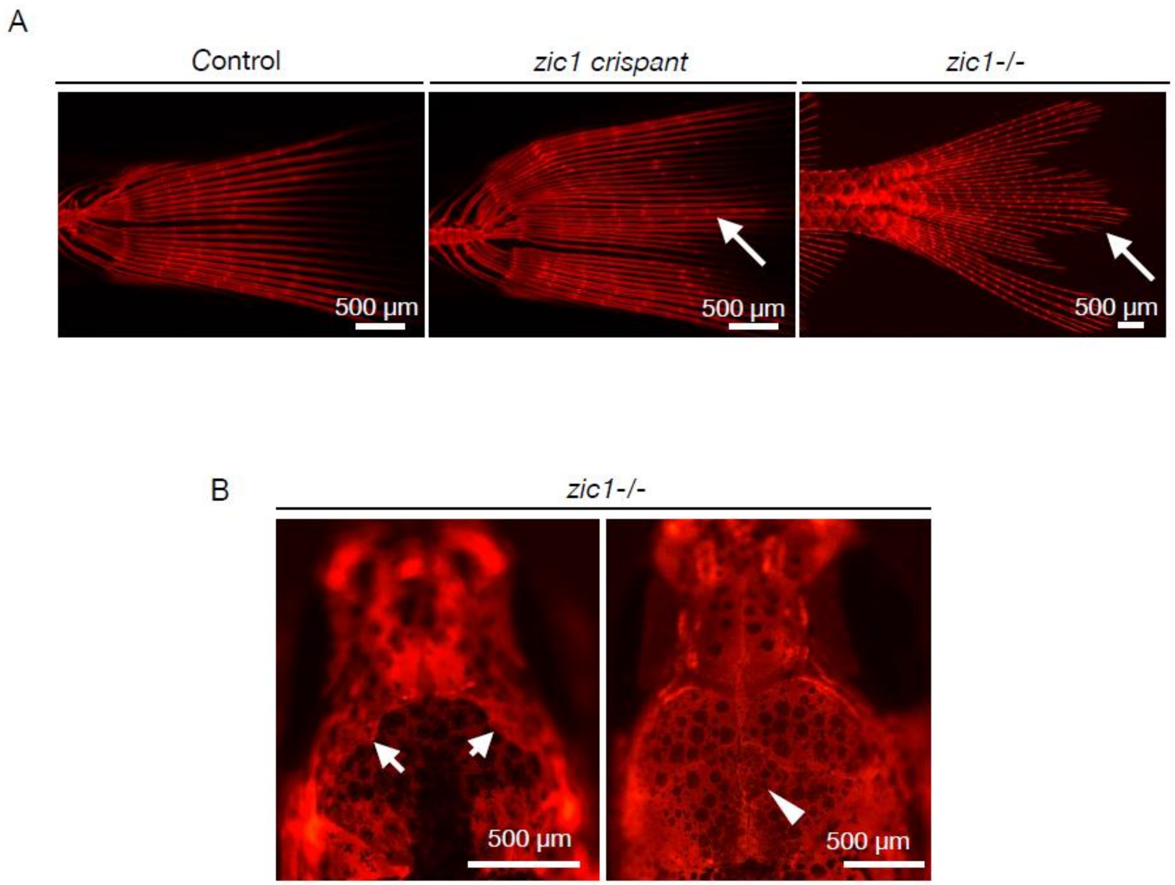

Figure 4. Zebrafish *zic1* homozygous mutants show similar phenotypes as crispants. A) Asymmetrical caudal fin phenotype was found among crispants and it is a persistent trait in homozygous mutants. Asymmetrical caudal fin phenotype was detected in 13/55 (24%) of zebrafish**. B) *zic1* homozygous mutant phenotype.** *Zic1* homozygous mutants showed a delay in skull formation, with uneven bone growth (arrows), that can culminate in ectopic suture (arrowhead), like in crispants. While skull phenotype was not detected in any zebrafish with normal fins, all zebrafish with abnormal caudal fins showed irregular cranial bone growth. Scale bars are indicated in each figure.

2)The discussion is very brief, and there is little mention of the much of the work that has been done. There is little mention of the gene expression studies, and their role in osteoblast differentiation, or the fact that some causative SNPs have been identified. The discussion ould be expanded to more accurately reflect all of the data presented in the paper.

Please note that the manuscript does not follow a typical IMRaD format following the journal style. Therefore, in a way, a considerable part of the discussion is already included in the results section aiding the interpretation of the findings. Following the reviewer’s suggestion, we have brought some of these points to the discussion section, next to extending the section in an attempt to more accurately reflect all the work presented in the manuscript.

Minor comments:

1)Table 1 is a little confusing. I don't not know what is meant by "notes refer to annotation based on the closest gene." Also, there is no description of what the asterisks (by rs61863293) is for, or the number sign (#) in the footnote.

We apologise for the confusing notation, we have modified the table hoping it is now clear.

2)Line 25- This sentence, referring to high heritability of SK-BMD, should be supported with a reference.

We have added a reference to this statement.

3)Line 31- Does skull BMD readings include the jaw? If so, then the phrase indicating that skull bone density can unravel gene and pathways important for intramembranous ossification should be reworded as most of the jaw is ossified through endochondral ossification.

We have followed the reviewer recommendation and clarified this further. Now lines 37-40 read "Intramembranous bones are predominant in the head, forming the cranial vault and face; however, the cranial base grows through means of endochondral ossification[9], and as such, investigating this skeletal site could capture molecular pathways from both processes."

4)Line 70-71- a nominally significant correlation was described between skull mineralization and infant head circumference- Is this data for the current study? If so, it should be included and the specific table/figure refereed to in the text.

Data from infant head circumference comes from a large GWAS meta-analysis published in 2012[17] as stated in Supplementary Table 7. We have added the correlation coefficient and p-value to the main text while adding a reference to infant head circumference in the text. Lines 77-79 "Nevertheless, a marginally significant correlation was observed between SK-BMD and infant head circumference[17] (ρ=-0.23; SE 0.07) (Supplementary Table 7)."

5) Line 77-Conversely, a uniform pattern for functional signatures was observed in the other six cell lines tested (GM12878, H1HESC, HeLa-S3, HepG2, HUVEC, K562). Is this data shown in the paper?

Thanks for calling our attention to this particular point. We indeed note that the statement you refer to did not reflect the results obtained, as although we see enrichment for chromatin states (Figure 5) and histone modifications (Figure 6) in other cell lines, they do not reflect the robust pattern we observe in osteoblast. In osteoblasts, variants associated at GWS threshold showed clear enrichment for enhancer, weak enhancer and transcribed regions. Concordantly,

trimethylation of the 27th lysine residue of the 3rd histone (H3K27me3) and trimethylation of the 9th lysine residue of the 3rd histone (H3K9me3) were not enriched, a result in line with lack of enrichment for repressed chromatin states. Simultaneously, variants were enriched for ATAC-Seq signatures in osteoblasts across all genome-wide p-value thresholds.

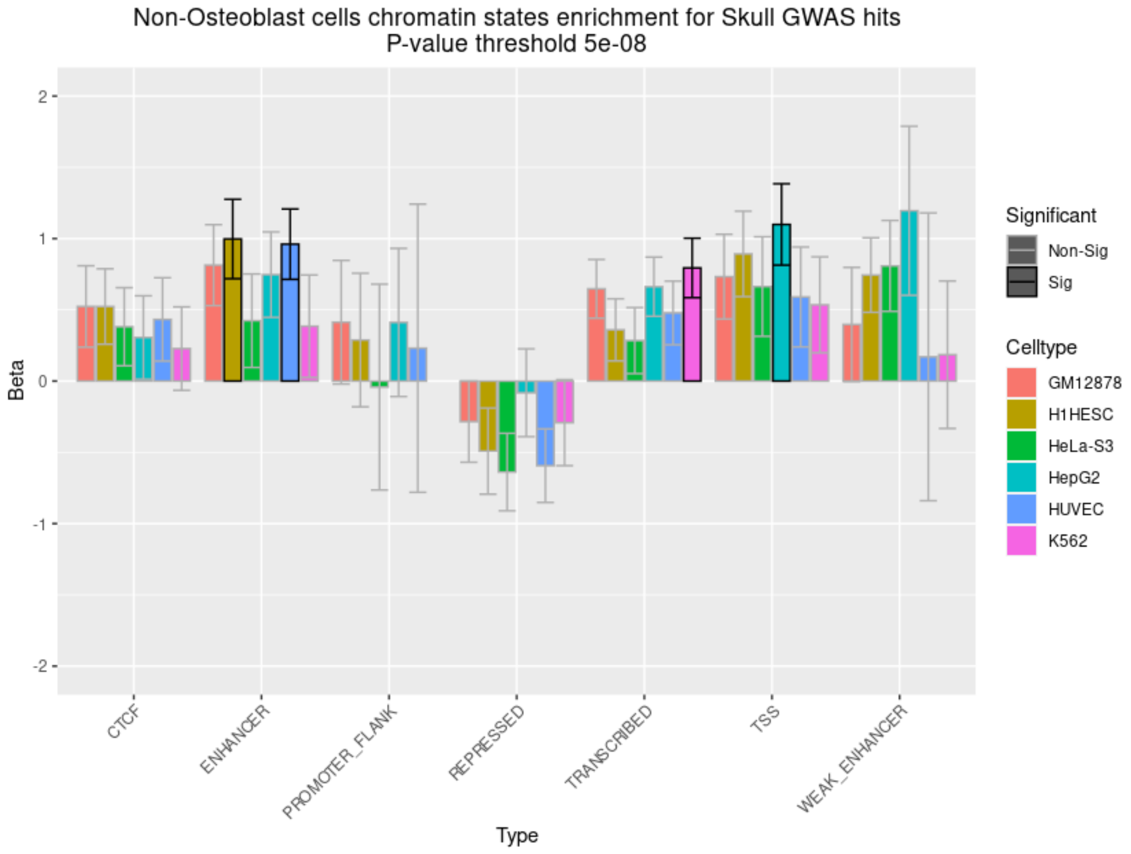

Figure 5. GARFIELD results for chromatin states enrichment analysis in non-osteoblastic cell lines. Enrichment significance was defined at P< $4.31x10^{-4}$, corrected by multiple-testing. Bar-plot fill colors represent the cell lines. Bar-plot outline colors represent significance of the enrichment.

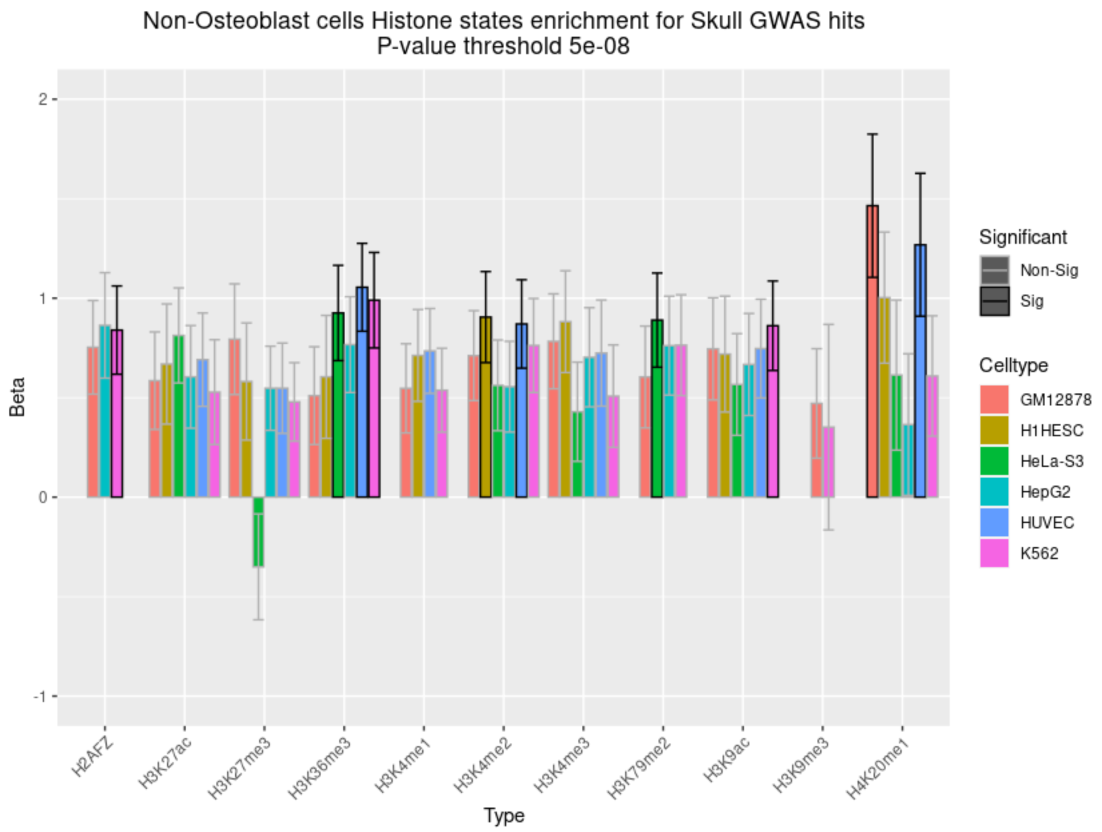

Figure 6. GARFIELD results for histone states enrichment analysis in non-osteoblastic cell lines. Enrichment significance was defined at P< 4.31×10^{-4}, corrected by multiple-testing. Bar-plot fill colors represent the cell lines. Bar-plot outline colors represent significance of the enrichment.

Therefore, we have rewritten the statements in our results (line 85) and discussion (lines 232-233) regarding this analysis. We also noted that we described the wrong number of participants (as not all the samples had both GWAS-DNA and expression data); so, in the methods section we have corrected this.

6) Line 104- the following genes were prioritized……. Prioritized for what?

We have rewritten this sentence to improve its readability. Lines 111-112 now read “After filtering out interactions between proxy SNPs (r^2>0.4) and promoters not in open chromatin, the following genes were implicated by this approach…”

8)Line 123- ….”compartment. and”- Capital “A” or remove period?

We have changed this.

9) Line 155- the authors indicate that one novel locus GLRX3 was not chosen for further study, as it is involved in iron metabolism, and there was little supporting evidence that it regulates bone mineralization. As there is literature demonstrating that iron intake and metabolism is associated with osteoporosis, this statement should be reworded.

Now, this statement reads. "The association signal in the remaining novel locus mapped ~2.7 Mb upstream of *GLRX3*, a gene involved in iron homeostasis[38] (**Figure 1**). In view of the long distance between the association signal and its closest gene, and the lack of evidence for a regulatory function of the lead variants, we did not select any gene at this locus for functional follow-up." [lines 167-170].

10) Line 171- a p-value of 0.066 would generally not be considered significant. This statement should be reworded.

Thanks for pointing us to this typo. As you can see in **Figure 4** of the manuscript, p=0.0066 rather than 0.066, we have modified this in the body of the manuscript.

11) Line 183- "With relevant" should be "with relevance?"

We have corrected this typo.

12) For gene expression analysis in bone tissue, samples were taken from women with osteoarthritis. This should be discussed, as it is possible the expression of some genes may not occur in healthy tissue or may be sex specific.

We agree in principle with the reviewer that expression of genes might be affected by disease or even hormonal status, which might vary with age and sex, amount of bone material, among others factors.

The dataset used in our analysis includes 71 biopsies from males and females with no known morbidities.

Sampling	Hip Fracture - Caput	Hip Osteoarthritis - Caput	Iliac
**Gender Female**	14	21	71
**Gender Male**	2	13	0

References

1. Justice, C.M. *et al.* A genome-wide association study identifies susceptibility loci for nonsyndromic sagittal craniosynostosis near BMP2 and within BBS9. *Nat Genet* **44**, 1360-4 (2012).
2. Justice, C.M. *et al.* A genome-wide association study implicates the BMP7 locus as a risk factor for nonsyndromic metopic craniosynostosis. *Hum Genet* **139**, 1077-1090 (2020).
3. Medina-Gomez, C. *et al.* Life-Course Genome-wide Association Study Meta-analysis of Total Body BMD and Assessment of Age-Specific Effects. *Am J Hum Genet* **102**, 88-102 (2018).
4. Carrington, B., Varshney, G.K., Burgess, S.M. & Sood, R. CRISPR-STAT: an easy and reliable PCR-based method to evaluate target-specific sgRNA activity. *Nucleic Acids Res* **43**, e157 (2015).
5. Marigorta, U.M., Rodriguez, J.A., Gibson, G. & Navarro, A. Replicability and Prediction: Lessons and Challenges from GWAS. *Trends Genet* **34**, 504-517 (2018).
6. Palmer, T.M. *et al.* Using multiple genetic variants as instrumental variables for modifiable risk factors. *Stat Methods Med Res* **21**, 223-42 (2012).
7. Park, J.H. *et al.* Distribution of allele frequencies and effect sizes and their interrelationships for common genetic susceptibility variants. *Proc Natl Acad Sci U S A* **108**, 18026-31 (2011).
8. Pasman, J.A. *et al.* GWAS of lifetime cannabis use reveals new risk loci, genetic overlap with psychiatric traits, and a causal influence of schizophrenia. *Nat Neurosci* **21**, 1161-1170 (2018).
9. Thorleifsson, G. *et al.* Genome-wide association yields new sequence variants at seven loci that associate with measures of obesity. *Nat Genet* **41**, 18-24 (2009).
10. Turcot, V. *et al.* Protein-altering variants associated with body mass index implicate pathways that control energy intake and expenditure in obesity. *Nat Genet* **50**, 26-41 (2018).
11. You, R. *et al.* High Level of Uromodulin Increases the Risk of Hypertension: A Mendelian Randomization Study. *Front Cardiovasc Med* **8**, 736001 (2021).
12. Moriyama, Y. *et al.* The medaka zic1/zic4 mutant provides molecular insights into teleost caudal fin evolution. *Curr Biol* **22**, 601-7 (2012).
13. Dehairs, J., Talebi, A., Cherifi, Y. & Swinnen, J.V. CRISP-ID: decoding CRISPR mediated indels by Sanger sequencing. *Sci Rep* **6**, 28973 (2016).
14. Kague, E. *et al.* Osterix/Sp7 limits cranial bone initiation sites and is required for formation of sutures. *Dev Biol* **413**, 160-72 (2016).
15. Dambroise, E. *et al.* Fgfr3 Is a Positive Regulator of Osteoblast Expansion and Differentiation During Zebrafish Skull Vault Development. *J Bone Miner Res* **35**, 1782-1797 (2020).
16. Teng, C.S. *et al.* Altered bone growth dynamics prefigure craniosynostosis in a zebrafish model of Saethre-Chotzen syndrome. *Elife* **7**(2018).
17. Taal, H.R. *et al.* Common variants at 12q15 and 12q24 are associated with infant head circumference. *Nat Genet* **44**, 532-538 (2012).

Appendix 1

	GENERAL INFO						NO WEIGHT NO HEIGHT			WEIGHT HEIGHT			DIF	
RSID	CHR	BP	EA	EAF	INFO	N	BETA	SE	P	BETA	SE	P	beta-dif	% change
rs12132533	1	110475552	A	0.799	1.00	37743	0.027	0.009	0.0015	0.031	0.009	0.00036	0.0034	0.1255
rs55652172	1	113147607	G	0.796	1.00	37743	-0.047	0.009	3.50E-08	-0.052	0.008	1.20E-09	0.0047	0.1008
rs7550321	1	163875519	C	0.538	1.00	37743	0.014	0.007	0.048	0.014	0.007	0.041	0.0004	0.0301
rs495590	1	172122809	G	0.465	0.99	37743	-0.026	0.007	0.00019	-0.022	0.007	0.0012	0.0035	0.1339
rs2795312	1	220129357	T	0.648	0.99	37743	0.037	0.007	3.70E-07	0.037	0.007	1.80E-07	0.0009	0.0238
rs7526484	1	22451845	T	0.238	1.00	37743	0.076	0.008	4.00E-21	0.074	0.008	1.80E-20	0.0014	0.0183
rs3971300	1	22484575	T	0.732	0.99	37743	0.107	0.008	5.40E-43	0.109	0.008	1.80E-44	0.0018	0.0168
rs12728589	1	22692315	G	0.822	1.00	37743	-0.076	0.009	2.80E-17	-0.081	0.009	1.80E-19	0.0050	0.0658
rs12132335	1	68634642	G	0.866	0.97	37743	0.047	0.010	4.70E-06	0.050	0.010	1.00E-06	0.0031	0.0657
rs2566752	1	68656697	T	0.624	0.99	37743	-0.049	0.007	3.50E-12	-0.047	0.007	2.60E-11	0.0021	0.0431
rs72962911	2	119104991	A	0.872	1.00	37743	-0.045	0.010	1.40E-05	-0.043	0.010	2.90E-05	0.0017	0.0383
rs11679232	2	119621910	C	0.702	1.00	37743	-0.033	0.008	8.90E-06	-0.033	0.007	8.70E-06	0.0000	0.0009
rs10490046	2	40630678	A	0.786	0.99	37743	0.075	0.008	6.50E-19	0.076	0.008	1.70E-19	0.0011	0.0151
rs11675489	2	85498783	A	0.544	0.99	37743	0.037	0.007	8.50E-08	0.039	0.007	2.70E-08	0.0013	0.0355
rs12107945	3	147163978	G	0.712	0.99	37743	0.047	0.008	4.70E-10	0.047	0.008	9.70E-10	0.0009	0.0196
rs74394007	3	156692207	A	0.870	0.98	37743	0.022	0.010	0.032	0.026	0.010	0.013	0.0035	0.1586
rs428510	3	41138172	G	0.437	1.00	37743	-0.069	0.007	5.00E-23	-0.067	0.007	6.20E-22	0.0019	0.0271
rs11934731	4	88831249	G	0.321	0.99	37743	0.087	0.007	2.50E-31	0.089	0.007	7.70E-33	0.0021	0.0236
rs3755955	4	994414	G	0.848	0.99	37743	0.086	0.010	6.20E-19	0.088	0.010	3.60E-20	0.0029	0.0336
rs454968	5	112188456	T	0.357	1.00	37743	0.036	0.007	6.30E-07	0.029	0.007	6.80E-05	0.0072	0.2014
rs9327301	5	122860170	G	0.739	1.00	37743	0.071	0.008	5.70E-20	0.074	0.008	1.50E-21	0.0029	0.0409
rs11948073	5	72618265	G	0.462	1.00	37743	0.038	0.007	5.00E-08	0.040	0.007	9.70E-09	0.0019	0.0510
rs1101560	6	127047776	C	0.750	0.99	37743	-0.046	0.008	6.90E-09	-0.051	0.008	1.40E-10	0.0049	0.1064
rs113622822	6	127178839	G	0.976	1.00	37743	-0.108	0.022	1.30E-06	-0.100	0.022	7.00E-06	0.0078	0.0722

rs1963689	6	127425630	T	0.754	0.99	37743	-0.063	0.008	5.40E-15	-0.061	0.008	2.80E-14	0.0017	0.0278
rs75191738	6	130348257	C	0.849	0.97	37743	0.051	0.010	1.40E-07	0.044	0.010	6.50E-06	0.0073	0.1435
rs13204469	6	133366810	A	0.676	0.99	37743	0.095	0.007	2.20E-37	0.097	0.007	4.20E-39	0.0022	0.0231
rs11759873	6	133641473	A	0.651	1.00	37743	0.080	0.007	3.10E-28	0.078	0.007	2.10E-27	0.0013	0.0167
rs9478217	6	151874122	A	0.403	1.00	37743	-0.056	0.007	1.30E-15	-0.060	0.007	6.80E-18	0.0043	0.0775
rs17423748	6	44943982	T	0.700	1.00	37743	0.064	0.008	1.90E-17	0.068	0.008	2.50E-19	0.0036	0.0569
rs10456165	6	53617914	C	0.786	0.99	37743	-0.048	0.008	1.40E-08	-0.051	0.008	2.20E-09	0.0026	0.0535
rs2490298	6	6939532	A	0.621	0.99	37743	0.043	0.007	2.10E-09	0.042	0.007	3.10E-09	0.0005	0.0117
rs9447004	6	74458737	A	0.486	1.00	37743	0.028	0.007	4.00E-05	0.028	0.007	6.20E-05	0.0007	0.0258
rs56335989	7	120790559	T	0.553	1.00	37743	-0.028	0.007	4.20E-05	-0.027	0.007	7.90E-05	0.0011	0.0377
rs6965195	7	120873438	G	0.615	1.00	37743	0.065	0.007	7.40E-20	0.063	0.007	8.50E-19	0.0020	0.0302
rs2908004	7	120969769	G	0.554	1.00	37743	-0.121	0.007	5.00E-69	-0.126	0.007	1.00E-74	0.0049	0.0404
rs55963900	7	120977734	G	0.719	1.00	37743	-0.149	0.008	9.60E-84	-0.153	0.008	8.70E-89	0.0044	0.0296
rs4383904	7	96121636	T	0.335	1.00	37743	-0.044	0.007	2.20E-09	-0.046	0.007	2.70E-10	0.0024	0.0549
rs11993347	8	103919090	T	0.771	1.00	37743	0.050	0.008	9.30E-10	0.049	0.008	2.10E-09	0.0011	0.0219
rs10108399	8	109396911	T	0.353	1.00	37743	0.030	0.007	2.90E-05	0.031	0.007	1.50E-05	0.0010	0.0324
rs7842942	8	120008587	T	0.431	1.00	37743	0.081	0.007	1.60E-31	0.085	0.007	7.40E-34	0.0031	0.0376
rs61863293	10	132252499	A	0.329	0.94	37743	-0.006	0.008	0.4	-0.004	0.008	0.58	0.0022	0.3444
rs1373004	10	54427825	T	0.106	0.99	37743	-0.071	0.011	2.20E-10	-0.073	0.011	8.30E-11	0.0016	0.0218
rs1944461	11	121917163	T	0.626	1.00	37743	0.041	0.007	9.00E-09	0.046	0.007	1.10E-10	0.0049	0.1204
rs1440705	11	15903307	G	0.732	1.00	37743	-0.038	0.008	7.70E-07	-0.040	0.008	3.20E-07	0.0012	0.0319
rs56257551	11	259575	C	0.895	1.00	37743	-0.025	0.011	0.029	-0.025	0.011	0.025	0.0006	0.0260
rs1351176	11	27280398	C	0.780	0.99	37743	0.061	0.008	2.00E-13	0.064	0.008	1.60E-14	0.0027	0.0443
rs10742173	11	27322158	G	0.380	1.00	37743	0.083	0.007	9.20E-32	0.088	0.007	2.30E-35	0.0047	0.0568
rs11029963	11	27367731	T	0.631	1.00	37743	-0.044	0.007	7.80E-10	-0.043	0.007	1.60E-09	0.0008	0.0191
rs11030009	11	27463564	A	0.484	0.99	37743	0.117	0.007	1.10E-63	0.118	0.007	1.50E-65	0.0016	0.0137
rs2553773	11	35083633	C	0.441	0.99	37743	-0.044	0.007	3.10E-10	-0.044	0.007	1.60E-10	0.0007	0.0156
rs4506602	11	46815633	C	0.481	0.99	37743	0.046	0.007	2.80E-11	0.046	0.007	4.10E-11	0.0004	0.0095
rs11228240	11	68218290	C	0.726	0.99	37743	0.106	0.008	4.20E-43	0.105	0.008	4.90E-42	0.0015	0.0141
rs7302653	12	107373522	G	0.489	1.00	37743	-0.021	0.007	0.0029	-0.025	0.007	0.00031	0.0043	0.2089
rs10843202	12	28716044	T	0.239	1.00	37743	-0.063	0.008	4.90E-15	-0.070	0.008	8.00E-18	0.0061	0.0969

rs11551274	12	49224108	G	0.970	0.99	37743	0.186	0.020	3.50E-20	0.178	0.020	1.40E-18	0.0083	0.0446
rs143348825	12	49248340	G	0.983	0.98	37743	0.038	0.027	0.15	0.032	0.027	0.23	0.0061	0.1591
rs77105837	12	49277680	A	0.943	0.98	37743	0.028	0.015	0.067	0.035	0.015	0.019	0.0077	0.2774
rs10875906	12	49385679	C	0.724	0.97	37743	-0.052	0.008	2.60E-11	-0.051	0.008	4.30E-11	0.0007	0.0129
rs4842705	12	90412755	A	0.581	1.00	37743	-0.051	0.007	4.40E-13	-0.056	0.007	6.10E-16	0.0058	0.1150
rs7977788	12	93982332	G	0.778	0.99	37743	-0.047	0.008	1.70E-08	-0.039	0.008	2.90E-06	0.0081	0.1719
rs493248	13	37473386	C	0.764	1.00	37743	-0.050	0.008	1.30E-09	-0.053	0.008	1.50E-10	0.0028	0.0555
rs138818878	13	43148546	C	0.970	0.94	37743	-0.113	0.021	8.60E-08	-0.111	0.021	1.20E-07	0.0015	0.0133
rs8047501	16	392318	A	0.498	0.98	37743	0.067	0.007	1.60E-21	0.067	0.007	1.10E-21	0.0002	0.0030
rs1984529	16	87182052	G	0.561	0.99	37743	0.045	0.007	1.90E-10	0.044	0.007	3.20E-10	0.0006	0.0136
rs4790881	17	2068932	C	0.302	1.00	37743	-0.042	0.008	1.90E-08	-0.043	0.008	7.40E-09	0.0011	0.0265
rs34814687	17	41862277	G	0.922	0.99	37743	-0.084	0.013	8.60E-11	-0.084	0.013	9.70E-11	0.0004	0.0043
rs12941447	17	63305939	A	0.112	0.98	37743	-0.075	0.011	1.40E-11	-0.073	0.011	2.90E-11	0.0013	0.0177
rs12601958	17	63820304	A	0.690	0.99	37743	-0.031	0.007	3.00E-05	-0.029	0.007	0.00012	0.0025	0.0798
rs71378928	17	66453305	C	0.216	0.99	37743	-0.044	0.008	1.50E-07	-0.043	0.008	3.90E-07	0.0015	0.0344
rs2214207	17	67672730	T	0.614	1.00	37743	0.028	0.007	7.90E-05	0.028	0.007	6.10E-05	0.0004	0.0143
rs72856781	17	68060451	A	0.653	0.99	37743	-0.059	0.007	9.20E-16	-0.058	0.007	2.70E-15	0.0011	0.0181
rs236529	17	68217231	A	0.405	0.98	37743	0.025	0.007	0.00044	0.024	0.007	0.00086	0.0013	0.0524
rs11650468	17	837381	T	0.591	0.97	37743	-0.050	0.007	1.40E-12	-0.050	0.007	1.80E-12	0.0003	0.0066
rs6567278	18	60059716	C	0.410	1.00	37743	-0.043	0.007	8.60E-10	-0.046	0.007	8.20E-11	0.0025	0.0586
rs6040061	20	10640306	C	0.525	0.99	37743	0.050	0.007	4.60E-13	0.051	0.007	2.40E-13	0.0006	0.0114
rs6029124	20	39088536	G	0.730	0.99	37743	-0.025	0.008	0.0012	-0.029	0.008	0.00015	0.0043	0.1714
rs9976876	21	36970350	G	0.520	0.99	37743	0.039	0.007	1.50E-08	0.043	0.007	7.50E-10	0.0034	0.0866

REVIEWERS' COMMENTS:

Reviewer #1 (Remarks to the Author):

Thank the authors for adding more details and analyses. I have only one comment, please do mention that the 12.5% phenotypic variance may be over-estimated due to the Winner's curse effect. After all, it was estimated from the same samples that discovered those loci.

Reviewer #2 (Remarks to the Author):

The authors have done an excellent job responding to the criticisms of this paper with new data and text. I have no major concerns and I enthusiastically support its publication in Nature Communications. One minor point- With respect to the significant association that was observed 2.7 MB upstream of GLRX3, the authors state that there is a lack of evidence to indicate it falls in a regulatory region. I'm not sure if I have missed the evidence for this, maybe it could be specifically stated (lines 167-70). Did the authors assess conservation of potential sites that may be epigenetically modified amongst species, which is a useful way of identifying conceived regulatory regions, or was this statement based on the lack of eQTLs associated with the SNP?

Response to reviewers

We would like to thank the reviewers for their concluding comments and remarks which have been incorporated in this new version of our manuscript.

Reviewer #1 (Remarks to the Author):

Thank the authors for adding more details and analyses. I have only one comment, please do mention that the 12.5% phenotypic variance may be over-estimated due to the Winner's curse effect. After all, it was estimated from the same samples that discovered those loci.

We have added this limitation to the results, where we report the variance explained . Lines 56-57 now read “As this estimate was obtained from the same samples used as discovery, it might have been over-estimated due to the winner's curse effect.”

Reviewer #2 (Remarks to the Author):

The authors have done an excellent job responding to the criticisms of this paper with new data and text. I have no major concerns and I enthusiastically support its publication in Nature Communications. One minor point- With respect to the significant association that was observed 2.7 MB upstream of GLRX3, the authors state that there is a lack of evidence to indicate it falls in a regulatory region. I'm not sure if I have missed the evidence for this, maybe it could be specifically stated (lines 167-70). Did the authors assess conservation of potential sites that may be epigenetically modified amongst species, which is a useful way of identifying conceived regulatory regions, or was this statement based on the lack of eQTLs associated with the SNP?

On pp. 169-170, we referred to the lack of any evidence from eQTLs in GTEx (v.8) either from our own datasets on chromatin conformation in osteoblasts and eQTLs in osteoclasts. However, we found the suggestion from the reviewer valuable and sought for evidence coming from epigenetic marks and conservation scores in the UCSC genome browser (Figure 1). Again, we found no evidence for this association peak to be involved in transcription regulation (no enriched H3K27Ac marks), and the conservation score is low (based on the 100 vertebrates base-wise conservation by PhyloP), In addition, there is no alignment of this DNA segment across vertebrates (e.g., fishes).

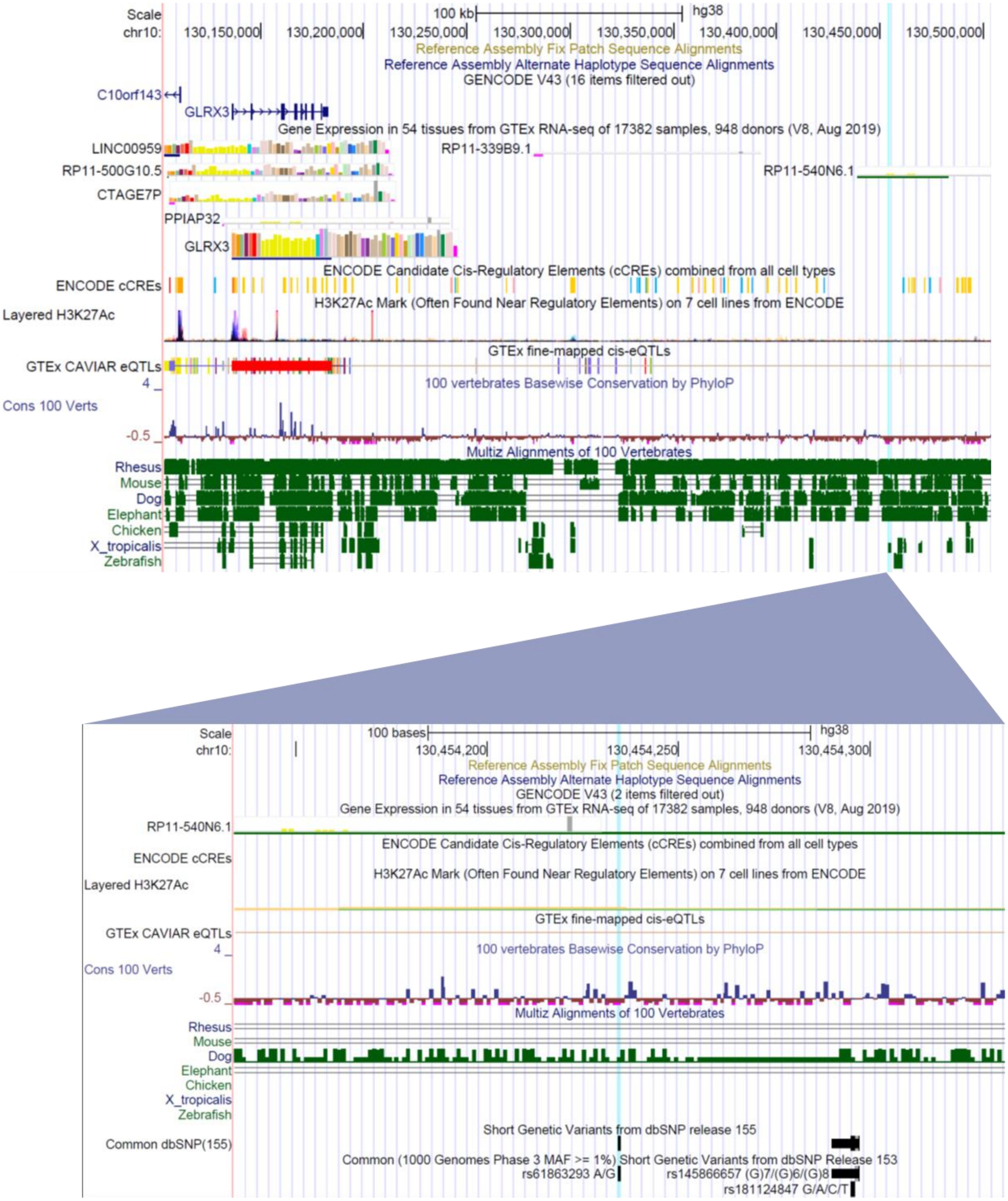

Figure 1. UCSC browser survey for evidence of the potential regulatory role of rs61863293. The upper panel, shows information on GTEx summarizing eQTL results, H3K27Ac marks and conservation scores for the region between *GLRX3* and rs61863293 (depicted by a cyan line). The lower panel, zooms into the region where rs61863293 seats.
